# 119th Annual Meeting Medical Library Association, Inc. Chicago, IL May 3–8, 2019

**DOI:** 10.5195/jmla.2020.897

**Published:** 2020-01-01

**Authors:** JJ Pionke, Ellen Aaronson

**Affiliations:** Proceedings Coeditor and Applied Health Sciences Librarian, University Library, University of Illinois at Urbana-Champaign, 1408 West Gregory, Urbana IL 61801, pionke@illinois.edu; Proceedings Coeditor and Librarian, Libraries, Mayo Clinic, 200 First Street SW, Rochester, MN 55905, aaronson.ellen@mayo.edu

## INTRODUCTION

The Medical Library Association (MLA) held its 119th annual meeting in Chicago, Illinois, May 3–8, 2019, at the Hyatt Regency Chicago. The meeting theme was “Elevate.” Total attendance for the meeting was 1,661, with 228 participating in continuing education courses. Additional meeting content—including the meeting program and various electronic presentations from the business meetings, plenary sessions, poster sessions, and program sessions—can be accessed by all meeting registrants via the MLA ‘19 website.

## WELCOME TO MLA ‘19

### Sunday, May 5, 2019

Executive Director Kevin Baliozian welcomed attendees and then introduced MLA President Beverly Murphy AHIP, FMLA. President Murphy welcomed attendees to the 2019 annual meeting.

President Murphy then introduced Merle Rosenzweig, who welcomed attendees on behalf of the Midwest Chapter.

Merle Rosenzweig: I want to thank Beverly again. And she has been practicing my last name for a long time, and she did it! So, to everyone here, all the MLA members, the Midwest Chapter of the Medical Library Association welcomes you to the 2019 annual meeting. The chapter was founded in 1950 and includes the great states of Illinois, Indiana, Iowa, Kentucky, Michigan, Minnesota, North Dakota, Ohio, and Wisconsin.

A little history of the chapter: During the World War II years, MLA could not have its meetings, and some of the regional groups continued to have their meetings. When Janet Doe was president, a committee was formed to set up guidelines for regional groups, and the committee was chaired by Margueriete Prime. And anyone who knows Margueriete Prime has been here longer than I have. And they recommended the establishment of regional groups and presented a set of guidelines to be observed.

So, with our annual meeting of the Midwest Chapter, we stimulate and foster an interest in health sciences libraries and librarianship, and acquaint persons interested in health sciences libraries and librarianship with MLA. All of the chapters are part of MLA, obviously, and we do promote MLA.

The Midwest Chapter hopes that you have a very productive meeting here in Chicago in 2019 and have the opportunity to see some of the sights, sounds, and food that Chicago has to offer.

So that is my short speech, and now it’s my pleasure to introduce a group of very creative people who made this great meeting here in Chicago possible. They will come on stage now. [*Applause.*]

James Dale Prince, AHIP: Good morning. I’m Dale Prince from the Louisiana State Health Sciences Library–New Orleans.

Mellanye J. Lackey, AHIP: Good morning. I’m Mellanye Lackey from the University of Nevada–Las Vegas Health Sciences Library. We’re so glad you’re here.

Dale Prince: On this first day of Ramadan—and *adayk ramadan almubarak.* I may have mispronounced that but have a blessed Ramadan. On behalf of the 2019 National Program Committee (NPC), your NPC, welcome to Chicago, the Windy City, the City of Big Shoulders, and home to the iconic L, Chicago’s elevated transit system, one of the seven wonders of Chicago. We don’t have a list of what the others are. [*Laughter.*]

Mellanye Lackey: But you can Google them.

Dale Prince: Librarians. So we’ve borrowed this for our theme, Elevate. It’s awe-inspiring. It’s about movement, just like libraries, librarianship, and librarians. And like the L, we hope the programming of this year’s meeting will elevate you and take you to places you want to be.

Mellanye Lackey: Some of the new things in this year’s meeting include an option to list your chosen pronouns during registration and have them printed on your badge; immersion sessions with surveys to collect immediate feedback; this year’s service project, Sit, Stay, Read, which brings human and dog volunteers into low-income Chicago public schools to advance children’s literacy. MLA members, you all have already contributed $500 to buy books for kids to read to dogs. Thank you for your generosity. Visit their booth today from 1:00 p.m. to 3:00 p.m. and tomorrow from noon to 2:00 p.m. at the escalator above the exhibit hall, right by registration, to see more of the adorable dogs and to learn more about their work.

Dale and I would also like to recognize members of the NPC who have put in countless hours of service to MLA to make this meeting a success. They have worked so hard to create an excellent program to inspire us and to elevate us. If the members of the NPC could please rise and be recognized. [*Applause.*]

Thank you. And now your Local Assistance Committee (LAC).

Rosie Hanneke, AHIP: Good morning. I’m Rosie Hanneke from the University of Illinois at Chicago.

Debra Werner: And I’m Deb Werner of the University of Chicago. We’re the cochairs of the LAC for MLA ‘19.

Rosie Hannneke: We’re so glad you made it to Chicago to elevate with us. We hope you will enjoy our beautiful and vibrant city while you’re here. From museums and shopping to dining to simply taking in the sights and sounds of the Loop, there are so many ways for you to spend your free time both here, downtown, and farther afield. And those of you who were lucky enough to get tickets to *Hamilton* last night, we hope you had a great time. But maybe play it down a bit for the rest of us. [*Laughter.*]

Deb Werner: Stop by the hospitality desk just outside the ballroom for more information about the Windy City. We’ve got directions, whether you’re going near or far, from maps of the pedway in this hotel to public transportation information if you’d like to explore one of Chicago’s seventy-seven unique neighborhoods. Be sure to pick up a bingo card courtesy of Sola Whitehead and Chris Shaffer, AHIP, and use it to meet new people.

This year, in partnership with many MLA chapters, we included dine-arounds on Friday and some on Monday as well, so if you attended one of these chapter dinners or happy hours, please let us know what you think of this addition.

Rosie Hanneke: We’d like to thank the LAC subcommittee chairs who worked behind the scenes to make this meeting a success. We’d also like to thank everyone else who volunteered their time to stuff meeting bags, host dine-arounds, staff the hospitality desk or career placement center, or help in any other way—all important and needed contributions. If you’re a subcommittee chair or volunteered in any capacity for the LAC, could you please stand so we can give you a hand. [Applause.]

Deb Werner: Thank you, everyone, and welcome, all of you, to Chicago, have a great meeting, and we’re so glad you’re here. Thank you. [*Applause.*]

Executive Director Baliozian returned to the podium to recognize and thank meeting planners and all the vendors who generously contributed to the meeting’s success. Each MLA ‘19 Gold sponsor gave a short one-minute speech. Gold sponsors were EBSCO Health, McGraw-Hill, and Wolters Kluwer.

Executive Director Baliozian then welcomed back President Murphy who introduced the In Memoriam video. President Murphy then acknowledged distinguished MLA members and introduced Cynthia Beeler, AHIP, to discuss the Academy of Health Information Professionals (AHIP) and recognize new members. President Murphy returned to the stage and introduced Past President Barbara A. Epstein, AHIP, FMLA, who joined President Murphy on the stage to acknowledge five new fellows to MLA (FMLA): Dolores Zegar Judkins, AHIP, FMLA; Michele Klein-Fedyshin, AHIP, FMLA; Amy Gische Lyons, AHIP, FMLA; Priscilla L. Stephenson, AHIP, FMLA; and Michele R. Tennant, AHIP, FMLA. President Murphy then awarded the 2010 Presidential Award to the Education Steering Committee. President Murphy then introduced Ana D. Cleveland AHIP, FMLA, the previous Marcia C. Noyes Award recipient, who then awarded M.J. Tooey, AHIP, FMLA, the 2019 Noyes Award.

## MLA PRESIDENTIAL ADDRESS: BEVERLY MURPHY, AHIP, FMLA (PLENARY SESSION 1)

Beverly Murphy, AHIP, FMLA: Good morning, again. Last year, during my inaugural address, I came out to “You Are the Sunshine of My Life,” and you are still the sunshine of my life. But this year, it’s “Love Like in Flight,” and that conveys my love for the profession in flight, because I’ve been in flight literally and figuratively. I know we’re all in flight. We’re on a journey. We should always be elevating, letting our profession and our roles take us up and away to the highest heights.

So, can you believe that it has been a year since we last had a conversation like this? In watching the clip that you saw earlier and thinking about what I wanted to talk to you about today, it occurred to me that a platform is not only a raised level surface on which people or things can stand, but in the tech world, it is the basic hardware and software on which applications can be run. Usually, they are updated with this version or that, but in our world, we’re not just going to update. It’s time to elevate. It’s time to raise the platform.

We have all moved from where we were last year to another space in time. So, what did I do in elevating my platform this past year? My presidential year actually started in May 2018 at the annual meeting in Atlanta—the home of Coke, grits, pie, and fried foods—when I was inaugurated as the first African-American president of our beloved association. [*Applause.*] We adapted, we transformed, and led, and it just got better from there.

With Cardiana in tow, I made waves at the Mid-Atlantic Chapter (MAC), my home chapter in Ocean City, Maryland—woo-hoo!—and I learned about artificial intelligence in systematic reviews. I had partners in progress and explored the possibilities of the Midcontinental Chapter, where I got a visual story, through infographics and data visualization, of partnership building, and I did it all virtually.

I shook, rattled, and rolled in Cleveland with the Midwest Chapter, I learned about work-life balance, and I had a fabulous time exploring the Rock & Roll Hall of Fame.

Since there are more chapters than I could possibly attend, I give a big thanks to my cohorts and partners. Past President Epstein presented at the Hawaii-Pacific Chapter, New York-New Jersey Chapter, and Pacific Northwest Chapter. President-Elect Julia Esparza, AHIP, presented at the North Atlantic Health Sciences Libraries (NAHSL) in New Hampshire and was a librarian on a mission at the South Central Chapter in San Antonio, Texas. Treasurer-Elect Shannon D. Jones, AHIP, experienced the magic in Orlando, Flortida, with the Southern Chapter. And Marie T. Ascher presented at the Philadelphia Regional Chapter and crossed borders and broke boundaries at Upstate New York and Ontario Chapter (UNYOC) in Niagara-on-the-Lake, Ontario, Canada. [*Applause.*]

In June, I will be part of a panel at the Northern California and Nevada Medical Library Group and the Medical Library Group of Southern California and Arizona joint meeting on critical health sciences librarianship examining our role in social justice. [*Applause.*]

In addition to attending chapter meetings, I participated in one way or another in about 200 meetings in person and virtually. I appointed some task forces; I wrote some articles, some letters, and some statements. I recently visited Capitol Hill with the Joint MLA/Association of Academic Health Sciences Libraries (AAHSL) Legislative Task Force, where we championed for funding for the National Library of Medicine, the National Institutes of Health, the Agency for Healthcare Research and Quality, the Institute for Museum and Library Services, and other initiatives.

The change of the MLA staff and headquarters management model to MCI is another initiative that I have been involved with along with the board. There are some new staff here today checking out our meeting for the first time, so please engage with them if you have the opportunity.

And along the way, I managed to get some ZZs in. For those who know me, they know that I’m a night owl. Enough said.

One of the largest initiatives and perhaps the most challenging last year and this year has been and is the restructuring of our changing communities. As a result of recommendations from the Communities Strategic Goal Task Force, we are officially in what I like to call “trends implementation mode.” This transition is grounded in the central objectives of diversity and inclusion and enhanced collaboration between our diverse member communities, strengthening of those communities, and improved experience and value for us as MLA members.

Our communities’ transition team—led by Stephanie Fulton, AHIP, Keith W. Cogdill, AHIP, and Kevin Baliozian—is in full swing, and they are doing a phenomenal job.

Our sections and special interest groups (SIGs), which leverage their skills in a very unique way, will all become caucuses. There will no longer be member fees associated with these communities, which should help to eliminate some of the inclusivity barriers. The caucuses are now deciding which of the seven domain hubs or practice areas they will be joining in order to connect to MLA-wide activities: programs like the annual meeting, continuing education, *MLAConnect,* and so on. Activity is brisk and engaging. Super Saturday was yesterday. There were lots of meetings and dialogue, and I’m really excited about what’s happening.

Now, on the governance side of the communities’ transition, members will elect the board and Nominating Committee, just as we have always done. Though the names will change, the process will be similar to what we have now, with a bit of expansion to be more inclusive. Section Council will become the Community Council, which will comprise caucus reps. The council will elect one board member and select six of eighteen members on the Nominating Committee slate.

As a reminder, Monday, from 10:30 a.m. to 11:55 a.m., the communities’ open forum will follow the education open forum. For the first time, you will be able to ask questions on the app, and they will be fielded as they are received. So, just a general reminder let us not forget our code of conduct as we participate in these sessions.

As I said last year, diversity makes us smarter because we grow from the ways in which we are different. As the Diversity and Inclusion Task Force, a.k.a. DITF, has coined, “Diversity, equity, and inclusion are the threads that strengthen the fabric of the Medical Library Association.” But when we talk about strengthening the fabric, we just can’t talk the talk; we have to walk the walk.

Sometimes a walk is uncomfortable, especially when we are engaging in critical conversations that we must have. As James Baldwin said, “Not everything that is faced can be changed, but nothing will be changed unless it is faced.” With that in mind, the task force members have been working hard since their appointment in 2017, and some of that work has been incorporated along the way. You may have noticed the new diversity and inclusion accessibility page this year on MLANET, which includes information about diversity and inclusion programming. They also have a web presence now with information about the work they’ve been doing and are continuing to do.

But the task force is not finished yet; they have work to complete. Plans are to conduct a survey of the membership in May or June to gather anonymous baseline data about member relations with the association and demographics. To allow them that time, the task force term has been extended until 2020; at which time, they will make their overall recommendations to the board. If you want to know more about what they’re doing, just look for a task force member and others who have a badge that looks like this, a little button, and engage in conversation.

Speaking of conversation, the DITF will sponsor a diversity dialogue roundtable discussion on diversity and inclusion issues as they pertain to MLA and health sciences librarianship. That’s tomorrow, Monday, May 5, from 6:00 p.m. to 7:30 p.m., in Regency Ballroom D. Come early or come in between, but come. They’d love to have you. I expect that the diversity, equity, and inclusion threads will become even more alive as our communities transform, and we will be stronger for it.

The Annual Meeting Innovation Task Force led by Lisa K. Traditi, AHIP, and Kevin Baliozian has been working hard since members were appointed in June 2018. Looking at the annual meeting from top to bottom and making recommendations for improvement has been their primary goal. Though the task force is for three years, their brainstorming and energy has been tremendous. As a result, it looks like they may be wrapping up their work shortly so that the implementation can continue in some areas and begin in others. So, stay tuned.

Sometime after this annual meeting, I will be spearheading a new campaign along with DITF member JJ Pionke, *MLAConnect* Editor Christine Willis, AHIP, and Kate Corcoran, MLA staff director for membership and communities. The fantabulous part about this is, every single member can play a part. All I will say for now is this: I am MLA. You are MLA. And we are MLA. So, get ready to share your stories.

As I have elevated in my life and career, I couldn’t end my presentation without putting a plug in for mentoring. Last year, I encouraged everyone to mentor at least one person during their career, and part of where I am today is because of mentoring. Mentoring spreads the seeds of diversity and inclusion and expands our connections. We should all have a symbiotic relationship, feeding off of each other, nurturing each other, and dropping knowledge seeds so someone can water them, another fertilize them, and they grow.

The names of the people who you’re seeing on the list now, those are my mentors, and they did just that, and I thank them all for that.

So, remember, over the next year, remain calm, remain open, remain flexible, and remain positive. Leverage what you know, recognize what you don’t know, and fill in the gaps with chocolate. [*Laughter; applause*.]

So, in addition to thanking my mentors, there are many others to thank, but I’d like to recognize a few now. My sugar, honey, darling, baby, sweetheart, Jerome Ballew. He is here today. Jerome, raise your hand. [*Applause.*] My posse—you all know who you are—the medical center library and archive staff, which is where I work. Thank you so much. MLA headquarters staff, we couldn’t do it without you. You’re just fantastic people to work with, and I’ve worked with you a very long time, and you just get better every year, so thank you.

And, all of you, thank you. I hope that along the way that I’ve inspired you with my optimism and energy and challenged you to be engaged and to embrace diversity and inclusion at the highest levels. Thank you all for the honor and opportunity to serve as your president. And you all, indeed, will always be the sunshine of my life.

Thank you so much.

This concludes the opening session. At 10:30 a.m., we will gather in this room for the eagerly anticipated John P. McGovern Award Lecture featuring four panelists of library power users from leading Chicago medical institutions, who will share their personal experiences about how, where, and on what devices they discover, access, and consume professional-level information. So be prepared to be enlightened by the fascinating information they will share with us.

Thank you. [*Applause.*]

## OTHER PLENARY SESSIONS

### Plenary Session 2. Sunday, May 5: John P. McGovern Award Lecture

Discovering New Pathways to Information: What Today’s Users Tell Us:

**Vineet Arora,** academic hospitalist; assistant dean, Scholarship and Discovery; and director, Graduate Medical Education Clinical Learning Environment and Innovation; University of Chicago Pritzker School of Medicine, Chicago, IL

**Margaret Danilovich,** assistant professor, Department of Physical Therapy and Human Movement Sciences, and assistant chair, Development and Communications, Feinberg School of Medicine, Northwestern University, Chicago, IL

**Allison Lale,** clinical associate, Center for Advanced Care at South Loop Family Medicine, University of Chicago Primary Care Network, Chicago, IL

**Janice M. Phillips,** director, Nursing Research and Health Equity, Rush University Medical Center, and associate professor, College of Nursing, Rush University, Chicago, IL

### Plenary Session 3. Monday, May 6: The Janet Doe Lecture

Introduction: Elaine R. Martin, FMLA, director, Library Services, Countway Library, Harvard University Medical School, Boston, MA

*The Activist Health Sciences Librarian*: **Gerald (Jerry) Perry, AHIP, FMLA,** associate dean, Libraries, and director, Health Sciences Library, University of Arizona–Tucson

### Tuesday, May 7: Get on Board: Hottest Ticket in Chi-Town Networking Event

The Get on Board: Hottest Ticket in Chi-Town Networking Event, was held on Tuesday, May 7, 2019, from 6:30 p.m.–10:00 p.m. The networking event featured a talent show that included a live band, singing, a sing-along, and poetry.

### Plenary Session 4. Wednesday, May 8: The Joseph Leiter NLM/MLA Lecture

Introduction: Sarah E. Katz, chair, Joseph Leiter NLM/MLA Lectureship Committee, and senior assistant librarian, Library, University of Delaware–Newark

**Nadya Okamoto,** social entrepreneur, activist, and founder and executive director, PERIOD: The Menstrual Movement

### Plenary Session 5. Wednesday, May 8

Introduction: Lisa A. Marks, AHIP, member, 2019 National Program Committee, and director, Libraries, Clinic Library, Mayo Clinic Scottsdale, Scottsdale, AZ

**Katherine (Katie) L. Watson,** associate professor, Medical Social Sciences, Medical Education, and Obstetrics and Gynecology, Feinberg School of Medicine, Northwestern University, Chicago, IL

### Wednesday, May 8: MLA ‘20 Invitation to Portland

The MLA ‘20 Invitation to Portland took place Wednesday, May 8,, 10:00 a.m.–10:15 a.m., and was hosted by Janna C. Lawrence, AHIP, chair, 2020 National Program Committee, and deputy director, Hardin Library for the Health Sciences, University of Iowa–Iowa City; Melissa De Santis, AHIP, cochair, 2020 National Program Committee, and director, Strauss Health Sciences Library, University of Colorado–Aurora; Stephanie C. Kerns, cochair, Local Assistance Committee, and director, Biomedical Libraries, Dartmouth College, Hanover, NH; and Tami Wilkerson, cochair, Local Assistance Committee, and librarian, StreamNet Regional Library, Columbia River Inter-Tribal Fish Commission, Portland, OR.

## BUSINESS MEETING

The Business Meeting was held on Tuesday, May 7, 2019, 9:00 a.m.–10:25 a.m. President Beverly Murphy, AHIP, FMLA, welcomed everyone to the meeting. President Murphy then called to order the Business Meeting of the 2019 MLA annual meeting and asked if a quorum of 200 voting members, required for transaction of business, was present. Sergeant-at-Arms Linné Girouard, AHIP, confirmed the quorum, and President Murphy called on Secretary Gurpreet Kaur Rana to move adoption of the Rules of Assembly. Secretary Rana explained that the Rules of Assembly include information on addressing the chair, presenting motions, debating, and voting. At the direction of the Board of Directors, she moved that the Rules of Assembly as they appear on MLANET be adopted. Voting paddles were raised, and there being a majority in the affirmative, the rules were adopted. Secretary Rana then announced that each meeting registrant had a printed copy of the *Official Program,* and that the agenda for the 2019 Business Meeting was on page 37. She moved that the agendas be adopted. The vote was affirmative, and the agendas were adopted.

President Murphy then asked Kevin Baliozian, executive director, to make introductions and announcements. Executive Director Baliozian presented the members of MLA’s 2018/19 Board of Directors: President Beverly Murphy, AHIP, FMLA; President-Elect Julia Esparza, AHIP; Immediate Past President Barbara Epstein, AHIP, FMLA; Treasurer Amy Blevins; Secretary Gurpreet Rana; Chapter Council Chair Melissa Ratajeski, AHIP; Section Council Chair Elizabeth Lorbeer, AHIP; Directors Marie T. Ascher, Keith W. Cogdill, AHIP, Stephanie Fulton, AHIP, Shannon D. Jones, AHIP, and Sandra Irene Martin, AHIP.

President Murphy then recognized and thanked retiring MLA Board Members and presented them with certificates as a token of respect and gratitude for work well done. She also expressed her gratitude to Past President Epstein, MLA president during the 2017/18 association year. Highlighting some of Past President Epstein’s initiatives, President Murphy presented a crystal gavel to her for a job well done.

President Murphy then called on Treasurer Amy Blevins to present the treasurer’s report.

Amy Blevins: I am going to talk about the communities a little bit, I’m going to talk about the annual meeting a little bit, and I’m going to talk about continuing education, since I’ve been reading Twitter and seeing some of the comments and concerns that people have. But first, I’m going to go through a little bit of a review of our finances.

What you can see on the screen here are the 2017 actual figures. And if you remember from last year, the parentheses mean a negative or a deficit. So, last year, we finished up around $27,894 not in the budget, but that was fine, because it was really an imaginary figure that had to do with the MLA InSight Initiative money going into the 2018 budget instead of 2017.

So, last year, we anticipated a revenue of $1,837, but we actually finished with $15,315. That, I’m told, is a 12% increase in actual revenue; $14,000 above budget. [*Applause*.] Thanks, everyone.

This is showing you our areas of growth. So this is in dollar amounts. And you can see that we’ve grown in revenue with our annual meeting; InSight Initiative; Institute of Museum and Library Services (IMLS) grant, which is part of what funds the Research Training Institute (RTI) membership; and all other. You might notice that the all publication, or the publications, is dipping. That’s because we’ve made the decision to have the *Journal of the Medical Library Association (JMLA)* as an open access journal. There has been a decrease in revenue from advertising, which is consistent with all online journals. And there has also been a decrease in revenue as people have switched to the online-only version instead of paper. But that’s fine. Don’t kill trees for your journals.

This is showing the percentages, or a pie chart, of our actual revenues from 2018, and you can see here that, as I mentioned last year—how many of you were here for last year’s report? Oh, good. I’m so glad you’re engaged. Fifty-two percent of our revenues came from the annual meeting, followed by 20% for membership and 9% from continuing education. I’m not going to read all the other ones to you, but you can kind of see where the money is coming in for the association.

I wanted to talk about the meeting a little bit more. So those of you on MEDLIB-L may have seen an email that I sent out kind of explaining why the meeting costs as much as it does. I am not saying this because I don’t empathize with the amount of money that the meeting costs, and I appreciate and value all of the people who can afford to come, while also sympathizing with those who cannot.

At this time, the annual meeting brings in most of the money for our association: 46% of our 52% comes from registration, and the other 54% is coming from our vendors. I hope everyone had the time to chat with our vendors yesterday afternoon.

Meetings are expensive, Chicago is expensive, having food at the hotel is expensive. But we do have an Annual Meeting Innovation Task Force headed up by Kevin Baliozian and Lisa K. Traditi, AHIP, and we are looking at ways that we can balance the value the meeting brings while also keeping costs lower. So, look forward to a survey and some focus groups in the future, where you can tell us what’s important about the meeting for you versus not.

In this chart, you see our revenues versus expenses. So on the left-hand side, these are the things that are generating money. On the right-hand side are things that are costing money. You’ll notice things like advocacy. So I know that Barbara Epstein and Beverly Murphy and other MLA presidents actually go up to Capitol Hill and lobby on behalf of our profession for the things that we value. Those things cost money rather than bring in money.

This last November, we made the decision to move continuing education (CE) from a cost-neutral to a revenue-generating cost center. And I know some of you may be thinking, well, continuing education is important and we can’t afford to spend money on it, so why would MLA make that decision? Well, in part, it’s because we need to be able to generate money from something other than just the annual meeting, because right now, if we have a bad annual meeting, we’re kind of in bad shape because that’s where we’re getting most of our revenue.

That being said, there are ways that we can maximize CE for everyone, while still balancing the need for it to make money. And I think we’ll be seeing more opportunities for free or low-cost continuing education from our communities, while we still have to understand that we’re paying for instructional designers and we’re paying for our members’ valuable time in delivering some of the CE opportunities that we see coming from our Education Steering Committee and from all the hard work that the curriculum committees are doing.

If you have more questions, I’m more than happy to talk to you. They told me I had five minutes. I told Beverly I might take more.

So, right here, you can see the 2018 actual budget versus the 2019 forecasted budget. Don’t panic. I know it says that we have a $126,914 deficit, but that is because of our move to the association management company, MCI, from this last November, when we approved to do that. They started in January, so you might have seen some of our new colleagues from MCI at the meeting this year.

The $126,914 is really a one-time cost that we’re using to invest in our future. We would have spent $163,000 for a new headquarters either way because our lease was expiring, and we had too much space. Now we only spent $95,000. And also, this is a one-time cost.

We have rainy-day funds in the form of $3.3 million. Half of it is endowment; half of it is reserve. And we use that to both invest in our future and to offset any years when we’re not doing as well as we had hoped—like maybe you remember 2009–2010.

So, change is constant and also difficult, but we have a lot of exciting and fun changes coming as well. This is my David Bowie caricature from a good friend of mine. Someone said people don’t know who David Bowie is, so just Google him if you don’t.

So we have upcoming MLA activities, community activities, and growth. You’ll see that in our 2020 budget. We’re working on new educational offerings, so hopefully you had an opportunity to attend the open forum yesterday. There was great information coming from there. And I don’t know if you were aware, but the 125-year anniversary for MLA is coming up in 2023, and so a lot of work will be going into raising an MLA scholarship fund to help offset the cost of the annual meeting for those who cannot afford to attend.

I’m not too concerned about all the changes that are coming, because MLA is all of you and all the people who aren’t in the room today. And I know there are a lot of really intelligent, wonderful, and dedicated people who are devoted to making our association the best that it can be.

Communities: I put this slide in here because I can’t really tell you what’s going to happen with finances and the treasuries for our sections as we move into communities, and I would be more scared, but I know that Shannon Jones is taking over as treasurer, and she’s working on the communities transitions teams with a bunch of other intelligent, invested people.

And really, it’s all about communication. If you have good ideas for doing this, share this with people. Share your ideas, share your concerns. A membership that questions its leadership is a membership that’s engaged, so I hope that you all reach out and participate. And then give everyone a little bit of grace, empathize. I know it’s difficult, but we’re going to get through it together, and it’s going to be better than ever.

So, that’s the end of my treasurer’s report. [*Applause*.] I’ll leave you guys with an image of Cujo ordering things from Amazon, and Cooper just living his best life. I hope that Shannon Jones includes lots of pictures of Cooper in the future in our slides. Thank you, everyone. [*Applause*.]

Next, President Murphy called on Executive Director Kevin Baliozian to give the executive director’s report.

Kevin Baliozian: Thank you very much, Beverly Murphy. Shannon Jones and I already have one major connection. I have a dog called Cooper, so it’s very exciting. [*Laughter.*]

So just a few quick photos of where we are at. Individual membership, or membership in general: We’ve been going down 4.5% for years. The good news is, this has stopped, or slowed, or to a fraction of things, and that’s great. So, we expect 2,650 members and 257 institutional members, which also is stable from prior years. Our retention rates floats around 84%, 83% to 85% depending on the year, which is above the standards for associations, so we’re quite happy about that.

What is very exciting is, we have a constant flow of new members. Many of them are here. And we should have 400, or perhaps even exceed that this year, so that’s great. So, generally, better news than it has been, and I think that’s excellent.

Annual meeting attendance this year is at 1,050 librarians, or at least members or people in the field, so that’s not counting staff; that’s not counting exhibitors. We had 500 exhibitor attendees on a hundred exhibits, which is impressive, that has actually been quite stable over the last few years. So, good news as well.

Now, every time you see the boxes go up, I’d like you to say, “Elevate,” because this is the next few slides. Member engagement: there will be a few slides on this. This is a really exciting story here. We have 627 of you members participating in MLA committees and juries. That does not count section roles, special interest group (SIG) roles, chapter roles. That’s 24% of the association just in committees, task forces, and juries. That’s phenomenal. [*Applause*.]

And the graph below is the number of people applying to be volunteers, and look at this: from 167 to 272. So, yes, our membership has gone down during that time, and the number of you engaged has gone up. This is phenomenal, and you are a big part of it. [*To Beverly Murphy:*] You said so many times, if you don’t apply, you can’t get on a committee. And how many times did you say this?

Beverly Murphy: Lots.

Kevin Baliozian: So, elevate!

Beverly and audience: Elevate!

Kevin Baliozian: Okay. So, SIGS and sections: Even with all the changes and some saying this is doom and everything coming, we still have 74% of people today, members who have joined at least 1 SIG or section, so that’s great. And for an individual who joins a SIG or section, you typically join 2.9; that’s hard to do; maybe 3. Let’s round up. Three sections or SIGs. So that’s great. And hopefully now, with less dues, you’d actually be participating for more. That is also spectacular, so, well done.

And then the next part down below is the number of members of the Academy of Health Information Professionals, which is also elevating.

Beverly and audience: Elevate!

Kevin Baliozian: From 899 to 1,070 today. That’s a 19% increase over the last 3 years, and 40% of members. That’s spectacular. So, thank you.

Scholarships, grants, other support: You can see how MLA, through its various programs, has significantly increased the amount of money that it provides to support programs for members. So, from $99,000 to an estimate of $170,000 this year, and the year’s not over yet. And that includes programs like scholarship grants and some of the funds that sections provide. That’s about $15,000 of that number. And then things like the IMLS grant and attending the RTI or the InSight Initiative Summits, which are essentially fully funded by MLA. So, that’s very impressive, and the number of individuals we’re touching is increasing. And this year, we got $20,000 in new grants for annual meeting travel in addition to all of the others, so that’s great.

That’s it. [*Applause*.] Thank you very much.

President Murphy then moved on to the annual report. In the interest of time, annual reports were received in a block. The informational reports of the appointed officials, councils, committees, task forces, representatives, sections, and chapters are found in the 2018/19 Annual Report of the Medical Library Association. These reports are available on MLANET and will remain there throughout the year. They are also available in paper copy from the executive director’s office by request. There being no corrections or objections from the members, the reports were filed as presented. President Murphy next introduced 2019/20 MLA President Julia Esparza, AHIP, who delivered her inaugural address.

## INAUGURAL ADDRESS

Julia Esparza, AHIP: Thank you, Beverly. I appreciate that wonderful introduction. Today, I would like to talk with you about how MLA is in the process of elevating our vision. First of all, who can tell me what this is? [*Several audience replies.*] I am sure almost everyone has at some point seen a roll of pennies. I want you to think of this roll of pennies as MLA. When we open this roll, we know what is inside: fifty pennies. Now, these individual pennies are the valuable members who make up MLA. They are not all identical, but they are all important parts of the whole.

You can take these fifty pennies and arrange them in all sorts of different ways. You can make ten groups of five, some larger groups, and some smaller groups, in all sorts of different stacks. But no matter how these individuals are arranged, they still make up the whole.

Medical libraries, we are all familiar with the structure of MLA as it is now. The pennies of MLA are arranged in many different ways, and these groups are not mutually exclusive. Members can join sections and SIGs, volunteer to serve on committees and juries, be nominated for and elected to leadership positions, and participate locally in our own regional chapters.

Sections and SIGs have been part of MLA for a long time. We are comfortable with these groups, but these groups are no longer meeting the needs of all of our members. You probably heard a lot of discussion about the communities transition during this annual meeting. Even now, you may still have questions: What will happen to my SIG or section? What the heck is a domain hub? Why does everything have to change?

There are important reasons that MLA is undergoing these transitions. It costs members extra money to join a section. Our members have cited these section dues as a barrier to participation. It can be difficult to find candidates who are willing to take on leadership positions in sections and SIGs with the leadership structure we now have.

Sections and SIGs tend to be siloed from each other, working on projects and research by themselves because we don’t have official avenues for collaboration beyond annual meeting programming. Right now, SIGs and sections are not equal. Sections get to send nominees to the MLA Nominating Committee and send representatives to the Section Council. SIGs don’t have this equal representation.

Section membership numbers are declining, and yes, this is—because these are data from before Kevin Baliozian presented his today, so don’t hold it against me on Twitter. [*Laughter*.] Hey, I know these people. [*Laughter*.] And section treasuries are depleting. Sections are spending beyond what the dues bring in.

The changes that MLA is undergoing are a reflection of what is happening in our profession. Two weeks ago, on April 23, the *2019 Horizon Report on Higher Education* was released. I highly recommend everyone read through this document, even if you think it may only apply to academic institutions, because many of the topics of the report can be applied to teaching and learning in clinical settings.

Today, I want to share with you the parts I found to be relevant to our discussion. Key trends: “Accelerating Technology Adoption” and “Higher Education.” The report identifies long-term, mid-term, and short-term trends. One of the long-term goals is rethinking how institutions work. This section of the report discusses how, quote, “because of economic and political pressures, institutions of higher education are actively developing new strategies to rethink how they fulfill their mission.” Does that not sound a little familiar?

For mid-term trends, the need for a growing focus on measuring learning highlights that institutions need to identify new ways to analyze data related to their students and that institutions have a great need to demonstrate process and achievements by their students to their administrations and outside accrediting agencies. The metrics that used to be a value may no longer be relevant. As libraries, information centers, or special libraries, we also struggle with identifying measures that represent our exceptional value to our administrators.

Finally, short-term trends highlight the growing need for redesigning learning spaces and blended learning designs.

The *Horizon Report* reflects the changes that are happening in our institutions. Change in our industries—be it academic, hospital, or special library—is not something new. We have all felt the evolution of librarianship, and we will all continue to adapt to new changes in the future. These themes of change are reflected in our environments. Our titles and our roles are changing. Some of us are still called librarians; some are called informationists, information specialists, or even data consultants.

Facilities are changing. Libraries are losing and sometimes gaining space in universities and hospitals. Have you repurposed space that once housed parts of your print collection to simulation centers, information commons, or maker spaces?

Teaching methods are changing in universities and in the health professions, and we, as librarians are having to grow and adapt to these changes. Has your institution developed innovative teaching techniques such as flipped classrooms or interprofessional education? Has your library been asked to aid in the development of these programs?

Our collections are changing, too. More and more resources are becoming electronic only. Some institutions are greatly reducing their print holdings or doing away with them altogether. Is your institution promoting open educational resources and asking the library to assist with the research or technology implementation of those resources?

With all these challenges, we do have to remember, some things haven’t changed. Research shows that physicians can have up to five clinical questions for each patient encounter. Research also shows that physicians don’t pursue all the answers to their questions. Their reasons for not pursuing an answer are that they didn’t believe an answer exists—that’s sort of scary—they would rather just use a curbside consult or consultation with a specialist, which opens them to legalities that we won’t discuss today; lack of time, which we heard about during the McGovern panel on Sunday; and finally, they didn’t know where to look. When physicians DID attempt to find answers, they failed almost 30% of the time.

The *Harvard Business Review* describes the nature of the type of smart employees who will be in demand in the future. They highlight that these individuals must be able to use higher order, critical, creative, and innovative thinking. Listening, relating, collaborating, and learning will also be crucial in these future employees.

Our education, our valuable skills in teaching information literacy, our critical thinking, our rich opportunities for continuing education through our association and other areas, and our understanding of the digital landscape make us the people to lead and educate on these issues. Because we facilitate the development of these skills in the people we serve, we are valuable to our organizations. Just like we continue to evolve as librarians to meet the needs of our patrons, MLA is evolving to meet the needs of its members.

The new community structure of MLA will reduce barriers to member participation by eliminating the division between sections and SIGs. All communities will be caucuses, and there will be no additional membership fees to be part of these growing communities. All communities will be on equal footing as caucuses, and each caucus will be represented in the new community counsel.

Domain hubs will create overarching conceptual communities that caucuses can be a part of, which will foster collaboration across groups that may not have worked together before.

And all of this together will help MLA to become a more nimble organization responsive to the needs of its members. In order to do this, we had to break our barriers. [*Breaks something to demonstrate the idea of breaking.*] While this may seem messy and noisy, this is part of growth.

Caucuses will have the flexibility to align with the domains that are relevant to the needs of their members. Caucuses within domains can work together. While this has been an established part of annual meeting planning, now this collaboration can go beyond annual meetings to projects and initiatives that may not have easily happened in the old organization. The organization may change and is meant to change as members express their ideas as to what is important to them. But the important thing to remember is that no matter how caucuses align, we will still add up to the whole. [*Applause*.]

Our differences, our basic professional needs, will make this whole more vibrant. This is why it’s so important for each of you to participate in the communities transition process. Throughout this annual meeting, we have heard from multiple groups that we need more constructive feedback from you as members. One example cited yesterday during the open forum on education was the low participation in the MLA competencies self-assessment. When your colleagues request your input, provide constructive comments. The communities transition team has been and will continue reaching out to your section or SIG for feedback, so don’t hesitate to speak up.

I really doubt that most of us will—given us—but I want to make sure that you all know. In the future, when you get a survey from a group studying the membership, such as the Diversity and Inclusion Task Force, then you need to participate in that anonymous survey. Domain hubs and the new community structure will only be as worthwhile as we make them together.

As Winston Churchill said, “To improve is to change, so to be perfect is to have changed often.” The exciting part of our new communities framework is that caucuses can continually reevaluate the needs of their members and align with domains as those collaboration centers change their focus in the years going forward; nothing will be static. Only with your participation can we develop a clear vision for MLA. As we move forward, our vision will become sharper with the ideas that you contribute.

I thank you, MLA, for giving me this opportunity to serve as your president. I know that the future isn’t always clear; however, I know that together, we will do everything we can to make the future of MLA fantastic. Thank you. [*Applause*.] Thank you, everyone.

Get on board and join us for the hottest ticket at the Chi-Town networking event tonight, where you will enjoy a great dinner and be entertained by your colleagues at the open mic night.

This session is now closed.

## IMMERSION SESSIONS, LIGHTNING TALKS, AND PAPER SESSIONS

Breakout sessions for immersion sessions, lightning talks, and paper sessions were presented in 6 time slots: Sunday, May 5, 2:00 p.m.–3:25 p.m. and 4:30 p.m.–5:55 p.m.; Monday, May 6, 2:00 p.m.–3:25 p.m. and 4:30 p.m.–5:55 p.m.; and Tuesday, May 7, 2:00 p.m.–3:25 p.m. and 4:30 p.m.–5:55 p.m. Abstracts of papers that were scheduled to be presented are available on the MLA ‘19 website. The final version of the abstracts reflecting only those presented at the meeting is included as online-only supplemental file to the January 2020 issue of the *Journal of the Medical Library Association.*

## POSTER SESSIONS

Poster sessions were presented in 3 time slots: Sunday, May 5, 3:30 p.m.–4:25 p.m.; Monday, May 6, 3:30 p.m.–4:25 p.m.; and Tuesday, May 7, 3:30–4:25 p.m. Abstracts of posters that were scheduled to be presented are available on the MLA ‘19 meeting website. The final version of the abstracts reflecting only those posters presented at the meeting is included as an online-only supplemental file to the January 2020 issue of the *Journal of the Medical Library Association.* The actual posters are available online in the MLA ‘19 meeting website.

## OTHER MEETINGS AND EVENTS

### Pre-meeting activities

The MLA Board of Directors met on Thursday, May 2, and Friday, May 3. The Credentialing Committee met on Friday, May 3. On Saturday, May 4, the following MLA units met: 2020 National Program Committee, Chapter Council, Department of Veteran Affairs Librarians SIG Business Meeting, Eugene Garfield Research Fellowship Jury, Joint Section Council/Chapter Council, Leaders’ Recognition Reception, and Section Council.

### Sunday, May 5

On Sunday, May 5, the following MLA units met: Ad Hoc Committee to Review Core Clinical Journals, African American Medical Library Alliance SIG Business Meeting #1, chapter treasurers orientation, Data Catalog Collaboration Project information session, education committee chairs joint meeting, Fellows of MLA, History of the Health Sciences Section Business Meeting, International Cooperation Section Business Meeting, *Journal of the Medical Library Association (JMLA)* Editorial Board Meeting, LGBTQ Health Sciences Librarians SIG Business Meeting, Medical Humanities SIG Business Meeting, Medical Informatics Section Business Meeting, Medical Library Education Section (MLES) Business Meeting, Medical Library Group of Southern California and Arizona Business Meeting, MLA Book Discussion Group for *Blindspot,* New York-New Jersey Chapter Board Meeting, Nursing and Allied Health Resources Section (NAHRS) Business Meeting and Executive Board Meeting, Public Health/Health Administration Section Business Meeting, Research and Evidence-Based Practice Curriculum Committee Meeting, Research Section Research Award Judging Meeting, Resource Sharing SIG Business Meeting, Rising Stars Program Meeting, Social Justice Section Business Meeting, and Southern Chapter Executive Meeting.

### Monday, May 6

On Monday, May 6, the following MLA units met: 2021 National Program Committee Meeting, African American Medical Library Alliance SIG Business Meeting #2, Awards Committee Meeting, Books Panel Meeting, Cancer Librarians Section Business Meeting, Collection Development Section Business Meeting, Complementary and Alternative Medicine SIG Business Meeting, Consumer and Patient Health Information Section (CAPHIS) Executive Committee Meeting, Data SIG Business Meeting, Dental Section Business Meeting, Educational Media and Technologies Section (EMTS) Business Meeting, Federal Libraries Section Business Meeting, Governmental Relations Committee Meeting, Health Association and Corporate Libraries Section (HACLS) Business Meeting, Health Information Professionalism Curriculum Committee Meeting, Hospital Libraries Section (HLS) Executive Board Meeting and Business Meeting, Information Literacy in Medical Education (ILME) SIG Business Meeting, Information Services Curriculum Committee Meeting, Instruction and Instructional Design Committee Meeting, Librarians without Borders® Committee Meeting, Libraries in Curriculum SIG Business Meeting, Molecular Biology and Genomics SIG Business Meeting, Northern California and Nevada Medical Library Group Business Meeting, Osteopathic Libraries SIG Business Meeting, Pacific Northwest Chapter Business Meeting, Pediatric Librarians SIG Business Meeting, Pharmacy and Drug Information (PDI) Section Business Meeting, Professional Recruitment and Retention Committee Meeting, Public Services Section Business Meeting, Scholarly Communications Committee Meeting, Solo Librarians SIG Business Meeting/Chat, Systematic Reviews SIG Business Meeting, Technical Services Section Business Meeting, Translational Sciences Collaboration SIG Business Meeting, Veterinary Medical Libraries Section Informal Meeting, and Vision Science SIG Business Meeting.

### Tuesday, May 7

On Tuesday, May 7, the following MLA units met: Bylaws Committee Meeting, Clinical Librarians and Evidence-Based Healthcare SIG Business Meeting, Consumer and Patient Health Information Section (CAPHIS) Business Meeting, Diversity and Inclusion Task Force Meeting, Education Steering Committee Meeting, Information Management Curriculum Committee Meeting, Institutional Animal Care and Use SIG Business Meeting, Interprofessional Education SIG Business Meeting, *Journal of the Medical Library Association (JMLA)* data sharing policy: author discussion and preparation, Latino SIG Business Meeting, Leadership and Management Section Business Meeting, Leadership & Management Curriculum Committee Meeting, Joseph Leiter NLM/MLA Lectureship Committee Meeting, Membership Committee Meeting, MLA community managers and webmasters, *MLAConnect* Editorial Board Meeting, Research Section Business Meeting, Rising Stars presentations, section treasurers orientation, and Systematic Reviews SIG Informal Meeting.

### Wednesday, May 8

On Wednesday, May 8, the following MLA units met: Education Annual Programming Committee (EAPC) Meeting, Grants and Scholarships Committee Meeting, and Oral History Committee Meeting.

## OPEN FORUM

The MLA Open Forum was held on Monday, May 6, 10:30 a.m.–11:55 a.m.

## NATIONAL LIBRARY OF MEDICINE UPDATE

The National Library of Medicine (NLM) Update took place on Tuesday, May 7, 11:00 a.m.–11:55 a.m.

## LEGISLATIVE UPDATE

The Legislative Update was held on Tuesday, May 7, from 2:00 p.m.–3:25 p.m. Moderated by Cristina Pope, this update provided an overview of health funding, information issues, and policy.

## OTHER SPECIAL EVENTS, RECEPTIONS, AND AFFILIATE MEETINGS

### Friday, May 3, 2019

- National Network of Libraries of Medicine (NNLM) Steering Committee Annual Meeting (invitation only), 2:00 p.m.–4:00 p.m.

### Saturday, May 4, 2019

- Welcome Reception and Opening of the Hall of Exhibits, 5:00 p.m.–7:30 p.m.

### Sunday, May 5, 2019

- MLA New Members/First-Time Attendees Program and Breakfast, 7:00 a.m.–8:55 a.m.
- Yoga Class, 7:30 a.m.–8:30 a.m.
- Covidence Library Champions (invitation only), 8:00 a.m.–8:55 a.m.
- DOCLINE Users Group, noon–12:55 p.m.
- Librarians without Borders®/Elsevier Foundation Research4Life Grants: Round Table Discussion, noon–12:55 p.m.
- Elevate Your Practice with Research: Research Training Institute (RTI) Fellows and Faculty Share Their Experience Designing and Conducting Research, 1:00 p.m.–1:55 p.m.
- Association of Academic Health Sciences Libraries (AAHSL) Future Leadership Committee Meeting (invitation only), 1:00 p.m.–1:55 p.m.
- PubMed Update, 1:00 p.m.–1:55 p.m.
- International Visitors Reception, 7:00 p.m.–8:00 p.m.
- Schmooze with Science (invitation only), 7:30 p.m.–9:00 p.m.

### Monday, May 6, 2019

- AAHSL/NLM Fellowship Program Information Session, noon–12:55 p.m.
- Covidence Advisory Group Meeting (invitation only), noon–12:55 p.m.
- Lunch & Learn: Best Practices: Implementation and Rollout of Ovid Discovery (invitation only), noon–1:00 p.m.
- Lunch & Learn: One-Click Access to PDFs from PubMed: Using the New LibKey Service from Third Iron, noon–1:00 p.m.
- Academy of Health Information Professionals Q&A Session, 1:00 p.m.–1:55 p.m.
- Diversity Dialogues with the Diversity and Inclusion Task Force, 6:00 p.m.–7:30 p.m.

### Tuesday, May 7, 2019

- Lunch & Learn: Elsevier Luncheon for Medical Librarians (invitation only), noon–1:55 p.m.
- MLA Book Authors and Prospective Authors Gathering, 3:30 p.m.–4:25 p.m.
- Get on Board: Hottest Ticket in Chi-Town Networking Event, 6:30 p.m.–10:00 p.m.

### Wednesday, May 8, 2019

- Invitation to MLA ‘20 in Portland, 10:00 a.m.–10:15 a.m.
- Book Sales and Signings, noon–12:30 p.m.

## SUNRISE SEMINARS

Exhibitors held Sunrise Seminars to provide information and to introduce new products and services. The following seminars were held.

### Sunday, May 5, 2019

- EBSCO Health: Beyond Evidence on Fast Healthcare Interoperability Resources (FHIR)
- Elsevier: Defining a “Preprint”
- Springer Nature: Copyright Compliance for Commercial Use: Identify, Educate, and License
- Wolters Kluwer: Information Overload: An Opportunity for Library Service Development (invitation only)

### Monday, May 6, 2019

- American Psychological Association (APA) Sunrise Seminar
- EBSCO Health: A Total Transformation: How One Medical Library Took Charge of Their Physical and Digital Space
- VisualDx: Connecting Symptoms to Construct a Diagnosis

## TECHNOLOGY SHOWCASES

Six Technology Showcases were held throughout Sunday, Monday, and Tuesday.

- EndNote: More than Just a Reference Manager
- Implement a Systematic Review Management System with Covidence
- More than Just Reference Management: How DistillerSR and CuratorCR Can Make Your Job Easier
- OpenAthens: The Authentication Landscape
- The R2 Digital Library: A Health Sciences E-Book Database
- Standing Out from the Crowd: A New, Multidimensional Approach to Researcher Profiles

## CONTINUING EDUCATION COURSES

The 2018/19 Continuing Education Committee offered the following courses to 228 attendees on May 3 and May 4, 2019.

### Friday, May 3, 2019

**CE100** Advanced Searching Techniques and Advanced Strategy Design, **Instructors:** Julie Glanville, MCLIP, associate director, York Health Economics Consortium, University of York, York, United Kingdom, and Carol Lefebvre, HonFCLIP, independent information consultant, Lefebvre Associates, Oxford, United Kingdom

**CE101** Fields, Filters, and Fun: Incorporating Creativity and Craft into Database Literature Searches, **Instructors:** David Kaunelis, methods specialist, and Kelly Farrah, AHIP, research information specialist, Research Information Services, Canadian Agency for Drugs and Technologies in Health (CADTH), Ottawa, ON, Canada

**CE102** Health Services Research: Sources and Strategies for Effective Information Searching, **Instructors:** Judith E. Smith, informationist, Taubman Health Sciences Library, University of Michigan–Ann Arbor, and Abraham Wheeler, AHIP, librarian, College of Osteopathic Medicine, Flint Research, and Department of Epidemiology and Biostatistics, Michigan State University–East Lansing

**CE300** Developing Library Data Visualization Services from Scratch, **Instructor:** Fred Willie Zametkin LaPolla, research and data librarian, Health Sciences Library, New York University (NYU) Langone–New York

**CE301** Applying the ACRL Information Literacy Framework to Your Teaching, **Instructors:** Xan Goodman, AHIP, health sciences librarian and associate professor, and Samantha Godbey, education and psychology librarian and associate professor, University Libraries, University of Nevada–Las Vegas

### Saturday, May 4, 2019

**CE103** Effectiveness and Efficiency in Exhaustive Searches, **Instructors:** Wichor M. Bramer, biomedical information specialist, Medical Library, Erasmus University Medical Center, Rotterdam, The Netherlands, and Melissa L. Rethlefsen, AHIP, associate dean, George A. Smathers Libraries, and Fackler Director, Health Science Center Libraries, University of Florida–Gainesville

**CE104** Which Review Is Right for You? Matching Questions to Review Type and Teaching the Process to Others, **Instructor:** Margaret J. Foster, systematic reviews and research coordinator, Medical Sciences Library, Texas A&M University–College Station

**CE105** We’re Way Past Peas: Uses of Genetic Information to Understand Human Health and Guide Health Care Decision Making, **Instructors:** Diana Nelson Louden, biomedical and translational sciences librarian, Health Sciences Library, University of Washington–Seattle, and Carolyn Martin, AHIP, consumer health coordinator, National Network of Libraries of Medicine, Pacific Northwest Region, Seattle, WA

**CE106** Trials without Tribulations: Identifying Clinical Trials for Systematic Reviews and Other Clinical and Research Questions, **Instructors:** Julie Glanville, MCLIP, associate director, York Health Economics Consortium, University of York, York, United Kingdom, and Carol Lefebvre, HonFCLIP, independent information consultant, Lefebvre Associates, Oxford, United Kingdom

**CE107** Going for the Grey: Finding Grey Literature for Complex Reviews, **Instructors:** Sarah Bonato, reference/research librarian, Centre for Addiction and Mental Health (CAMH), Toronto, ON, Canada; and Kelly Farrah, AHIP, research information specialist, and Monika Mierzwinski-Urban, research information specialist, Research Information Services, Canadian Agency for Drugs and Technologies in Health (CADTH), Ottawa, ON, Canada

**CE302** Teaching Critical Appraisal Skills, **Instructor:** Laura Menard, assistant director, Medical Education and Access Services, Ruth Lilly Medical Library, Indiana University–Indianapolis

**CE400** Implicit Bias Training for Information Professionals, **Instructors:** Shannon D. Jones, AHIP, director, Libraries, and associate professor, Medical University of South Carolina–Charleston; and Kelsa Bartley, manager, Library Services, Reference and Education Department, Louis Calder Memorial Library, and Kimberly L. Reynolds, assistant professor, Clinical Pediatrics, University of Miami Miller School of Medicine, Miami, FL

**CE501** Beyond Pyramids of Evidence: Evaluating Research in the Health Sciences Literature, **Instructors:** Abraham Wheeler, AHIP, librarian, College of Osteopathic Medicine, Flint Research, and Department of Epidemiology and Biostatistics; Chana Kraus-Friedberg, AHIP, liaison, Program in Public Health, Department of Pharmacology and Toxicology; and Carin Graves, liaison librarian, Sociology, Social Work, Criminal Justice, and Human Development and Family Studies; Michigan State University–East Lansing

**CE502** Advancing Health Equity through Evidence-Based Public Health: How to Find the Evidence, **Instructors:** Elaina Vitale, academic coordinator, National Network of Libraries of Medicine, Middle Atlantic Region, Pittsburgh, PA, and Derek Johnson, health professionals outreach specialist, National Network of Libraries of Medicine, Greater Midwest Region, Hardin Library for the Health Sciences, University of Iowa–Iowa City

**CE601** Institutional Review Boards (IRBs): Integrating Information Professionals into the Process, **Instructors:** Taneya Y. Koonce, associate director, Research; Sheila V. Kusnoor, senior research information scientist; Zachary E. Fox, associate director, Information Services; Annette M. Williams, senior information scientist; and Mallory N. Blasingame, information scientist; Center for Knowledge Management, Vanderbilt University Medical Center, Nashville, TN

**CE602** Goal: Success at Being a Solo Librarian!, **Instructors:** Helen-Ann Brown Epstein, AHIP, FMLA, informationist, Health Sciences Library, Virtua Health, Mount Laurel, NJ, and Louise McLaughlin, information specialist, Health Sciences Library, Woman’s Hospital, Baton Rouge, LA

### Saturday, May 4, Symposium

**CE800** Managing from the Middle: Learning to Lead from Where You Are, **Panelists:** Joan Gallos, professor of leadership emerita, Wheelock College, Boston, MA; Rikke Sarah Ogawa, AHIP, director, Louise M. Darling Biomedical Library and Science and Engineering Library, University of California–Los Angeles; Shalu Gillum, AHIP, head, Public Services, Harriet F. Ginsburg Health Sciences Library, University of Central Florida College of Medicine–Orlando; Shannon D. Jones, AHIP, director, Libraries, and associate professor, Medical University of South Carolina–Charleston; Erinn Aspinall, AHIP, strategic initiatives librarian and communications coordinator, Health Sciences Library, University of Minnesota–Minneapolis; and Christine Willis, AHIP, director, Knowledge Management & Learning Resources, Noble Learning Resource Center, Shepherd Center, Atlanta, GA

## RESOURCES AND SERVICES

The online itinerary planner, sponsored by Wolters Kluwer, allowed attendees to peruse programs and events online. Complimentary WiFi was available throughout the Hyatt Regency Chicago, excluding the exhibit hall, courtesy of the JAMA Network. Live streaming was available on Twitter using the hashtag #MLANET19, and volunteer bloggers, the Local Assistance Committee, and the 2019 National Program Committee contributed to the official meeting blog with meeting tips, announcements, and more. For those seeking new jobs and prospective employers, the Job Placement Center was open from Saturday through Tuesday, and the MLA Resume Clinic was available Saturday through Monday. The Hall of Exhibits was open Saturday through Monday.

## SUPPLEMENTAL FILES

Appendix MLA ‘19 Immersion Session, Lightning Talk, and Paper Abstracts

Appendix MLA ‘19 Poster Session Abstracts

## 

**JJ Pionke, MA, MSI,** pionke@illinois.edu, Proceedings Coeditor and Applied Health Sciences Librarian, University Library, University of Illinois at Urbana-Champaign, 1408 West Gregory, Urbana IL 61801

**Ellen Aaronson, MLS, AHIP,** aaronson.ellen@mayo.edu, Proceedings Coeditor and Librarian, Libraries, Mayo Clinic, 200 First Street SW, Rochester, MN 55905

