# Supplementary material for: 119th Annual Meeting Medical Library Association, Inc. Chicago, IL May 3–8, 2019
Source: J Med Libr Assoc. 2020 Jan 1;108(1):E1–E17. doi: 10.5195/jmla.2020.897 (PMC6920001; doi:10.5195/jmla.2020.897)
Supplement: Appendix [file jmla-108-E1-s001.pdf]

Medical Library Association

MLA '19 Immersion Session, Lightning Talk, and Paper Abstracts

Any unsolicited abstracts for the annual meeting undergo a process of blind peer review. Abstracts of papers intended for presentation are reviewed by members of a panel of reviewers sponsoring the programs. The final decision on program speakers rests with the program planners.

Sunday, May 5, 2:00 p.m.–3:25 p.m.

Room: Columbus IJ (East Tower, Ballroom/Gold Level)

Innovation & Research Practice Immersion Session 1

Moderator: Margaret A. Hoogland, AHIP

My Favorite Tool

Margaret A. Hoogland, AHIP, Clinical Medical Librarian, Mulford Health Sciences Library - The University of Toledo, Temperance, Michigan

Karen Newmeyer, AHIP, Medical Librarian, Rocky Mountain University of Health Professions, Provo, Utah

David A. Nolfi, AHIP, Head, Research Engagement, Health Sciences/STEM Initiatives & Assessment, Duquesne University, Gumberg Library, Pittsburgh, Pennsylvania

Tamara M. Nelson, Instruction & Research Librarian, Rowland Medical Library, Jackson, Mississippi

Suzanne Fricke, AHIP, Animal Health Sciences Librarian, Animal Health Library, Pullman, Washington

Emily Vardell, Assistant Professor, Emporia State University School of Library and Information Management, Olathe, Kansas

Ariel Deardorff, Data Services Librarian, UCSF Library, San Francisco, California

Margaret Emily Ansell, AHIP, Nursing & Consumer Health Liaison Librarian, Health Science Center Libraries, University of Florida, Gainesville, Florida

Electra Enslow, AHIP, Head of Library Research and Instruction, Spokane Academic Library/Washington State University, Spokane, Washington

Denise Rumschlag, AHIP, Jovy O'Grady, Scholarly Communications Librarian, New York Medical College Health Sciences Library, Valhalla, New York

Cecelia Vetter, NLM Associate Fellow, U.S. National Library of Medicine, Washington, District of Columbia

Fred King, Medical Librarian, MedStar Washington Hospital Center Washington, DC 20010, Washington, District of Columbia

Marilia Antunez, Life & Allied Health Sciences Librarian, The University of Akron, Akron, Ohio

Stephanie Shippey, AHIP, Associate Managing Director, Texas Tech University Health Sciences Center Library, Lubbock, Texas

Laura Menard, Assistant Director for Medical Education and Access Services, Indiana University, Indianapolis, Indiana

Margaret A. Hoogland, AHIP, Clinical Medical Librarian, Mulford Health Sciences Library - The University of Toledo, Temperance, MichiganJoin the Educational Media and Technologies Section for a fast-paced, informative, and most of all, unconventional and fun session! We guarantee it will be unlike any other MLA event you have ever attended. You will discover new tools for teaching, managing time, analyzing data, searching, and other tasks you or your patrons may want to accomplish with ease and style. You will also see how you can explain the benefits of specific tools to patrons who may be reluctant to try something new. We encourage you to arrive early or on time, claim a spot with your friends or colleagues, get your device ready, and be an active participant by voting for each contestant.

Sunday, May 5, 2:00 p.m.–3:25 p.m.
Room: Columbus EF (East Tower, Ballroom/Gold Level)

Education Session 1

Moderator: Debra R. Berlanstein, AHIP

Health Sciences Librarians' Perceptions of Interprofessional Education and Collaboration Research

Rachel Hinrichs, AHIP, Health Sciences Librarian, IUPUI, Indianapolis, Indiana

Kristine M. Alpi, AHIP, University Librarian, Oregon Health & Science University, Portland, Oregon

Tara Brigham, Assistant Professor of Medical Education & Medical Librarian, Mayo Clinic, Jacksonville, Florida

Caitlin Bakker, AHIP, Research Services Liaison Librarian, University of Minnesota, Minneapolis, Minnesota

Gregg A. Stevens, AHIP, Health Sciences Librarian, Stony Brook University, Stony Brook, New York

Amy Gische Lyons, AHIP, Associate Librarian, Liaison to the School of Nursing, SUNY at Buffalo, Health Sciences Library, Buffalo, New York

Objective
Librarians have a unique perspective on the value of working with other health professions. We sought to learn more about health sciences librarians' experiences with interprofessional activities and to assess their perceptions of interprofessional education (IPE) using a standard measure, the Interprofessional Education Perception Scale (IEPS).
Methods
We used a cross-sectional survey design to assess librarians' perceptions toward IPE, and to gather information on librarian participation in interprofessional activities. The survey consisted of a demographics section; the IEPS, an instrument developed to assess perceived attitudes about interdisciplinary collaboration for one's own profession; and questions about the librarian's prior and current experiences with IPE. It was sent via email lists to the MLA Interprofessional Education Special Interest Group (IPE-SIG), and the Research Section (RS), as a comparison group. After overlap between groups was addressed, mean IEPS scores between populations were compared to explore differences in attitudes and perceptions. Other variables of interest included years of experience as a librarian, previous career as a health professional, and experience teaching or supporting interprofessional education. We also compared librarians' IEPS scores with those of health professional students published previously (Hawk, 2002).
Results

Librarians' scores on the IEPS indicated highly positive perceptions towards IPE. There were no statistically significant differences between the IPE-SIG and RS groups (p=0.59), years of experience as a librarian (p=0.82), previous career as a health professional (p=0.91), or experience supporting IPE (p=0.16). Librarians' mean IEPS score (262.9) was slightly less than the mean score of all health profession students (265.9) published previously (Hawk, 2002), but was not statistically significant (p=0.43). Themes identified from two open-ended questions included librarian involvement in teaching and facilitating required learning activities for health professions students, committee involvement, and non-curricular activities such as Grand Rounds and book clubs. Five respondents addressed impact of their activities. Less frequent themes included perceiving respect for librarians as part of IPE, feeling undervalued, and desiring more involvement.

Conclusion

This study provides the first data for the IEPS with health sciences librarians. Health sciences librarians have highly positive attitudes towards IPE, in line with the majority of other health professionals studied previously. Years of experience, previous health professional careers, and experience supporting IPE as a librarian had little bearing on the responses to the survey. This suggests that health sciences librarians have positive attitudes towards IPE, regardless of whether they directly support IPE programs.

Preparing Health Sciences Students for Real-World Information Gathering Using Teams, Cases, and Critical Pedagogy Research

Margaret Henderson, AHIP, Health Sciences Librarian, San Diego State University Library, Ramona, California

Objectives: Teaching in health sciences is moving to Team-Based and Case-Based Learning (TBL, CBL), focusing on health inequalities and disparities to better mirror real world conditions. Will TBL and CBL, using cases based on healthcare disparities, enhance learning in one-shot curriculum-based information literacy sessions in the health sciences and improve student literature review skills?

Methods: Classes are assigned to TBL/CBL or lecture classes, based on course instructor preference, the resources the instructor wants covered, room availability, and class size. The librarian reviewed cases for information needs and developed mini-cases for class use. After a review of search methods, teams were asked to find peer-reviewed, and in some classes grey literature, to help with their case. Teams then presented their search strategies and results to the whole class. Surveys will be sent out 3 or 4 weeks after each class, but within the semester, to see how well students met class objectives and how well they feel they completed the information collection part of their assignment. TBL/CBL class responses will be compared to lecture-based class responses about the usefulness of the class, as well as retention of search methods.

Results: Preliminary results show that students who participate in a TBL/CBL class feel better prepared to conduct a literature review than those in a regular lecture class.

Elevating Library Learning: Implementing "Self-Directed Learning" as an Educational Framework

Stevo Roksandic, AHIP, Regional Director Library Services, Mount Carmel Health System, Columbus, Ohio

Kenneth Wright, Health Sciences Librarian, Mount Carmel Health System, Columbus, Ohio

Background: Academic and health sciences librarians create and use a variety of teaching tools and methods to meet the educational needs and preferences of its users. From traditional brown bag 'Lunch & Learn' informational sessions, group lectures and demonstrations, PowerPoint presentations, video tutorials and diverse learning guides, we decided to enhance the library's pedagogical practices by creating and implementing Self-Directed Learning (SDL) tutorials, by exploring and practicing simultaneously theoretical learning and practical experiences. This transformative educational framework is essential for the library to remain relevant and an integral part of the education for our organization's health sciences lifelong learners.
Description: After an extensive literature review and comprehensive library assessment (i.e. learner demographics, educational needs and learning preferences, interest for current educational tutorials, financial and human resources, collaborative opportunities), two SDL software applications were evaluated. Evaluations were based upon ease of use, compatibility of software with organizational IR system, cost, technical support, installation, maintenance, authoring and structure of tutorials, and creation of tutorial evaluations. The first two SDL tutorials were developed through collaboration between librarians and medical and academic staff.
The SDL tutorials are designed with split-screen technology, enabling users to perform live interactive exercises without having to leave tutorial screens. Moving through tutorials they are required to take brief quizzes called "knowledge checks" which increase learner comprehension. SDL tutorials were implemented by making them accessible through the library website and certificates of completion are given after filling out an evaluation.
Conclusion: After having eight SDL tutorials available, learner evaluations of any completed SDL tutorial will be used as outcome measures of the library's newly implemented pedagogical practice, including: accessibility, usefulness, effectiveness, enhancement of personal and/or professional development, and overall comments.

Exploring Best Practices for Librarian Integration into Case-Based Small Group Learning

Gail Kouame, Assistant Director for Research & Education Services, Augusta University, Augusta, Georgia

Julie K. Gaines, AHIP, Head, AU/UGA Medical Partnership Library, Augusta Univ/Univ of GA Medical Partnership, Athens, Georgia

Background: Health sciences librarians and medical educators performed a study about undergraduate medical students’ use of resources in small group case-based learning experiences. Librarian activities included collecting students’ presentations, critiquing resources cited, and providing feedback. Librarians also performed in-person observations of student presentations. The librarians discovered gaps in information seeking skills on the part of both the students and faculty small group facilitators. Facilitators acknowledged their own need for training about effective searching. The goal of this project is to explore best practices for ongoing integration of health sciences librarians in case-based learning in undergraduate medical education to facilitate lifelong learning.

Description: As an outcome of the original study, one of the librarians participated in a teaching fellowship focusing on increasing librarian participation in small group case-based learning. She was invited to teach an orientation session on searching and critical appraisal skills to faculty small group facilitators at the beginning of the 2018-2019 academic year. Another librarian is taking part more regularly in small groups at her campus. A needs assessment is being developed for the small group facilitators to assess their needs for improving information seeking skills, how to integrate review of resources into student learning summary presentations, facilitator comfort level in providing feedback to students on resource use, and best ways to leverage librarian expertise. Librarians are also exploring ways to reinforce information seeking skills throughout the academic year through more intensive educational interventions, such as adding graded criteria.
Conclusion: The presence of the librarians in the small group case-based learning sessions prompts both students and faculty facilitators to consider the quality of cited information resources which are important for clinical reasoning skills for future clinical work. Due to increased participation by librarians, faculty facilitators are more open to having librarians attend small group case-based learning sessions. The librarians are also gaining increased recognition among small group facilitators. A needs assessment of faculty small group facilitators will help shape future librarian involvement, along with possible addition of graded criteria for student presentations.

Using Comics to Discuss the Geriatric Clerkship Experience

Ariel Pomputius, Assistant University Librarian, University of Florida Health Science Center Libraries, GAINESVILLE, Florida

Mallory Otto

Background: Graphic medicine—the intersection of comics and healthcare—offers many examples of memoirs that explore aging and palliative care. Roz Chast's "Can't We Talk About Something More Pleasant?" offers a deeply personal, biographical account of one's aging parents at the end of life. Reading and discussing a comic about the caregiver perspective of elder care gives medical students a space to discuss their own experiences working with geriatric patients. A clinician and a librarian collaborated to develop a two-hour book club for the end of the geriatric clerkship that medical students participate in during their fourth year.
Description: The Geriatrics Clerkship Program Director had previous experience with medical humanities; the liaison librarian to the Geriatrics department had recently taught the same graphic memoir to honors undergraduate students. They compiled an introduction to graphic medicine and a series of discussion questions and began leading a book club at the end of the geriatrics clerkship. The book club helps introduce students to healthcare concepts including discussion of costs of care with aging, caregiver burnout, hospital quality and customer service, dementia care, and palliative care. This presentation will discuss ways to introduce students to unfamiliar reading formats such as comic books, exercises and questions used to inspire students to share their experiences, the challenges and opportunities found in developing a medical humanities interlude built around a graphic novel at the end of the geriatric clerkship, and student responses to the exercise.
Conclusion: While the initial responses from the students to the program has been positive, evaluation forms will allow for deeper analysis of whether students feel the book club discussions and graphic novel format of the materials is useful as a means for addressing difficult topics and sharing their own experiences in the geriatric clerkship.

Sunday, May 5, 2:00 p.m.–3:25 p.m.
Room: Columbus GH (East Tower, Ballroom/Gold Level)

Global Health & Health Equity Immersion Session 1

Moderator: Caitlin Ann Pike, AHIP

Elevating the "Open" Conversation: Access to Health Information as a Social Justice Concern

Caitlin Ann Pike, AHIP, Health Sciences Librarian, IUPUI University Library, Indianapolis, Indiana

Caitlin Ann Pike, AHIP, Health Sciences Librarian, IUPUI University Library, Indianapolis, Indiana

Jere Odell, Scholarly Communication Librarian, IUPUI, Indianapolis, Indiana

Beth St. Jean, Associate Professor, University of Maryland College of Information Studies, College Park, Maryland

Bharat Mehra, Professor and EBSCO Endowed Chair in Social Justice, School of Library and Information Studies University of Alabama, Tuscaloosa, Alabama

Caitlin Ann Pike, AHIP, Health Sciences Librarian, IUPUI University Library, Indianapolis, IndianaSocial justice, including equitable access to information and bridging the digital divide, are concepts familiar to many librarians. As a result, these ideas create a natural intersection for advocacy as health information professionals. As a brief background before the panel, we will review the literature on open access and social justice to provide context for the topic, and discuss survey results from undergraduate student opinions regarding open access. Panelists will then speak to the topic from their individual perspectives, and the audience will have an opportunity to engage and ask questions.

Sunday, May 5, 2:00 p.m.–3:25 p.m.
Room: Columbus CD (East Tower, Ballroom/Gold Level)

Information Management Session 1

Moderator: Janice Marie Young

Building a Health System Institutional Repository: Setting Yourself up for Success from the Start

Heather J. Martin, AHIP, Director, System Library Services, Providence St. Joseph Health, Portland, Oregon

Barbara (Basia) Delawska-Elliott, AHIP, Medical Librarian, Providence St. Joseph Health, Portland, Oregon

Daina Dickman, AHIP, Digital Asset Librarian, Providence St. Joseph Health, Portland, Oregon

Background: While more common in university settings, institutional repositories (IR) have a place within hospitals and healthcare systems too, though the challenges in creating them may be different. This paper looks at the development of a Digital Commons institutional repository at Providence St. Joseph Health. The authors present the necessary steps for a successful initiative beginning with the planning process and building from there. Highlighted are some of the different challenges faced in non-academic settings; considerations when selecting a platform and designing and structure; and recommendations for doing outreach and promotion to unique user groups.
Description: Library staff proactively followed trends in IRs and prepared for future state. Identifying technology requirements and platform options, exploring other health system IRs, and building a search algorithm to capture the affiliations within a newly merged enterprise meant that when approached by senior leadership about publication tracking the library was well positioned for success.
Library staff selected a platform, determined the repository's scope, and identified key stakeholders. An implementation plan included securing funding and executive support, hiring a librarian, and developing a framework for year 1 of the project.
A complex organization presented unique challenges when designing a metadata and taxonomy structure. Getting buy-in from clinicians required a strong elevator pitch, and a direct tie to the organization's strategic goals. Anticipating organizational needs meant a platform able to accommodate datasets, visual collections, Magnet documentation, and locally-published peer-reviewed journals
Conclusion: Digital Commons launched in July 2018, and within 3 months was populated with 700+ publications and included profiles from several world-renowned researchers. A successful Year 1 is on track and will be measured by hitting a publication target of 1000 and successfully importing all 2018 affiliated publications from PubMed. Library staff is now focused on marketing and outreach, with the intention to rapidly grow the Expert Gallery to include individuals from a diverse representation of disciplines and health system markets, increase submission of non-periodical scholarly activity, and to form collaborative partnerships for growth into new areas like datasets, and video.

Leading University Researchers in Research Data Management Planning and Curation

Jennifer Putnam Davis, Scholarship and Data Librarian, Augusta University, Grovetown, Georgia

Kathy Davies, Associate Director of Research, Augusta University, Augusta, Georgia

Stephanie Hendren, AHIP, Clinical Information Librarian, Robert B. Greenblatt, M.D. Library, Augusta, Georgia

Brenda L. Seago, Professor, Augusta University, Evans, Georgia

Julie Zadinsky, Assistant Dean for Research, Augusta University, Augusta, Georgia

Background: Robert B. Greenblatt, M.D. Library at Augusta University conducted a survey to determine the research data management needs of the University as a foundation for a collaborative data services model. Establishing this service would allow the institution to address skills, gaps, and existing services offered to support research data management. The initial step was providing professional development opportunities to embedded librarians assigned to the research community. This approach allowed library faculty to learn data management best practices and effectively plan for the implementation of future services and instruction programs.
Description: A Scholarly Communications position was restructured as Scholarship and Data Librarian to encompass data management responsibilities. The Associate Director of Research completed the National Library of Medicine (NLM) Research Data Management training course and began planning for library research data support. Greenblatt Library received funding from the National Network Libraries of Medicine Southeast/Atlantic (NNLM/SEA) region to host a campus-wide research data management symposium. The funding proposal was a collaboration with College of Nursing, Office of Research Support, and the Georgia Cancer Center. The symposium is intended to connect researchers and resources across all disciplines and provide professional development for researchers and librarians. The one-day symposium will be held March 2019 and feature national and local speakers, a panel discussion, poster session, and data resources exhibits. The library will incorporate data management in instructional programming such as webinars and expanded LibGuides. The symposium will be assessed by pre and post surveys and the instruction sessions will be evaluated by attendees.
Conclusion: Librarians have existing skills such as teaching, organizing, analyzing, and providing access to information sources that transfer readily to the research data management life cycle. The training symposium will increase campus awareness of library services for data management and facilitate new research collaborations. Training provided by the symposium will increase will increase library faculty capabilities to engage in the critical processes necessary for data sharing, scholarship, and research reproducibility. This symposium is the first stage in developing a research data services model to incorporate embedded librarians. The symposium survey results will be used to identify training needs for the university's research community.

Elevating Institutional Knowledge by Capturing, Preserving, and Repurposing: A New Type of Library Repository

Rebecca Harrington, AHIP, Assistant Professor, Research Services Librarian, University of Tennessee Graduate School of Medicine, Knoxville, Tennessee

David W. Petersen, AHIP, Assistant Professor, Research & Learning Services Librarian, Preston Medical Library, University of Tennessee Graduate School of Medicine, Knoxville, Tennessee

Background: Many hospitals and medical schools are still sharing institutional best practices through one-time lectures, grand rounds, and workshops – benefiting only those who attend in person. Recordings and PowerPoint slides are often locked in LMS systems or buried on department-specific websites in unorganized folders. Topics such as leadership, wellness, ethics, and effective teaching skills can benefit the entire organization. Instead of watching this wealth of experience and knowledge disappear into silos, or entirely, the librarians began to mine and capture presentations and their associated resources to create a shared knowledge repository.
Description: The library, being a shared resource between the hospital and the graduate school of medicine, is the logical choice to host best practices for interdisciplinary teaching and learning. The librarians approached stakeholders to support the creation of a LibGuides group to disseminate shared institutional knowledge, going beyond simply housing theses and dissertations to actively promoting the wisdom each organization has brought in. With subject listings and search features, the new system increases the content's value by broadening access to these materials across the two organizations. Next steps include using this shared institutional knowledge base to create interactive learning modules for asynchronous learning, removing attendance barriers so often found in the clinical setting.
Conclusion: Effectively implementing knowledge management initiatives requires building partnerships with the people in the organization who will have to invest in it, and eventually benefit from it. While building the repository, librarians will be reaching out to department leaders and presenters to expand the collection. Success will be measured when these stakeholders begin to submit their best practices and presentations to the repository. In addition, not only will the repository's usage statistics be measured, but also the number of places the repository is included (LMS pages, each organization's intranet and internet sites), and the requests/collaborations for learning modules.

Increasing Visibility of Research in an Institutional Repository through NCBI LinkOut

Erin D. Foster, Data Services Librarian, Indiana University School of Medicine, Indianapolis, Indiana

Hima Varsha Anne, Scholarworks Student Assistant, IUPUI University Library, Indianapolis, Indiana

Jere Odell, Scholarly Communication Librarian, IUPUI, Indianapolis, Indiana

David E. Polley, Social Sciences & Digital Publishing Librarian, IUPUI, Indianapolis, Indiana

Background: To increase the visibility and access to an academic university's institutional repository content by participating in the National Center for Biotechnology Information (NCBI) "Institutional Repository LinkOut" program.
Description: The authors used R, an open source programming language, and an R package called 'rentrez' to a) identify those articles in the university's repository that were in PubMed and b) determine of those, which ones did not already have full-text available via PubMed Central. Identifying articles in

PubMed that are not in PubMed Central is required by NCBI in order to participate in the "Institutional Repository LinkOut" program. Using the R package, a set of 4,400 open access items from the repository were processed, 557 eligible records were identified, and were sent to NCBI. In June 2018, the R scripts were revised to further streamline the process--at the beginning of July 2018 a total of 2,129 repository items were processed and 434 eligible records were identified for inclusion in the LinkOut program.
Conclusion: The university's institutional repository experienced a significant increase in visibility due to its participation in the NCBI's "Institutional Repository LinkOut" program. In its first implementation (July 2017), this automated solution was estimated to save over 30 hours of manual work on the part of the library staff. The LinkOut program has resulted in a 9% annual increase in web traffic to the repository and PubMed is now the third most frequent referral site to the repository. The R script and implementation process are publicly available, via GitHub, to help other institutions reduce the barriers for participating in the LinkOut program.

Organizing Your Organization: Taxonomy and Organizational Schemes in a Health System Institutional Repository

Daina Dickman, AHIP, Digital Asset Librarian, Providence St. Joseph Health, Portland, Oregon

Heather J. Martin, AHIP, Director, System Library Services, Providence St. Joseph Health, Portland, Oregon

Background: In the development of a new institutional repository, library staff at a large health system were faced with building an organizational structure that would be meaningful to both internal and external users. The health system context meant unique challenges designing a taxonomy that supported end users as well as institutional goals and branding. Health systems have unique user needs for specialized collections and organizational schemes. With a comparatively large volume of items being added there are also concerns with the backend structures to accommodate the institutions' large research output.
Description: This large, recently merged health system did not already have a predefined structure or taxonomy by which to organize or make collections browsable. Complex institutions without clearly set "Departments" will be interested to hear how library staff created and implemented an organizational structure and taxonomy for their institutional repository. By considering existing classification schemes, internal naming practices, and consulting small stakeholder focus groups publications are now easily browsable by both internal and potential external users.
Additional organizational structures were created to house collections in support of institutional goals. One of the debut specialized collections focuses on nursing evidence-based practice, and was leveraged as documentation during a recent Magnet recertification.
Tactics for creating backend organizational structures that support large volumes of items is also an area being explored in the first year of their institutional repository.
Conclusion: The institutional repository launched in 2018 with a taxonomy and organizational structure developed for the health system. The library continues to solicit feedback from stakeholders for improvements and ideas to serve internal and external users. Currently, different backend organizational structures are being tested in support of the large volume of items entered on a monthly basis in the institutional repository.

Sunday, May 5, 2:00 p.m.–3:25 p.m.
Room: Grand Ballroom A (East Tower, Ballroom/Gold Level)

Information Services Immersion Session 1

Moderator: Erik Davis Fausak

Megan G. Van Noord

Developing a Systematic Review Service: Blueprints for Success

Megan G. Van Noord, Health Sciences Librarian, University of California, Davis, Davis, California

Erik Davis Fausak, Health Sciences Librarian, Carlson Health Sciences Library - UC Davis Library, Davis, California

Megan G. Van Noord, Health Sciences Librarian, University of California, Davis, Davis, California

Erin RB Eldermire, Head, Flower-Sprecher Veterinary Library, Cornell University Library, Ithaca, New York

Margaret J. Foster, AHIP, Associate Professor (Systematic Reviews Coordinator), Texas A&M University, College Station, Texas

Leila Ledbetter, AHIP, Research and Education Librarian, Duke University Medical Center Library & Archives, Durham, North Carolina

Erik Davis Fausak, Health Sciences Librarian, Carlson Health Sciences Library - UC Davis Library, Davis, California

Megan G. Van Noord, Health Sciences Librarian, University of California, Davis, Davis, California

The body of biomedical literature has experienced an exponential growth in the number of systematic reviews published each year, which require the expertise of a librarian or other information specialist. To meet mounting institutional demands for librarian collaboration, many libraries have developed formal systematic review literature services. This session aims to bring librarians with established systematic review literature search service models, at different phases of implementation, to the table for a collaborative discussion and peer sharing of experiences, lessons learned, as well as guidance for librarians seeking to make informed decisions in how to structure similar services at their institutions.

Sunday, May 5, 2:00 p.m.–3:25 p.m.
Room: Columbus KL (East Tower, Ballroom/Gold Level)

Lightning Talks 1

Moderator: Wynn Tranfield

Self-Directed Evidence-Based Medicine (EBM) Medical School Curriculum: A Case-Based Approach

Deborah A. Crooke, Associate Director, New York Medical College, Health Sciences Library, Valhalla, New York

Marie T. Ascher, Lillian Hetrick Huber Endowed Director, New York Medical College, Valhalla, New York

Background: Evidence-based medicine (EBM) is part of the medical school curriculum. Librarians seek to provide EBM training in a practical way. As future physicians, medical students need to develop skills to find and evaluate evidence. Accreditors mandate that self-directed learning – opportunities for identification, analysis and appraisal of information – be included in the curriculum. A successful, novel case-based program was completed.
Objectives

- Provide hands-on experience finding, appraising and applying evidence
- Integrate learning with concurrent curriculum
- Provide opportunity to present in journal club style
- Partner with clinical faculty and fourth-year Learning-to-Teach students
- Employ a scoring rubric

Description: Librarians worked with academic administration to develop the second-year EBM curriculum using four atrial fibrillation cases. Cases coincided with the cardiology block to augment concurrent clinical knowledge. An EBM lecture modelled a journal club presentation of best evidence – a compulsory skill. Groups of 3-5 students were assigned one of four cases. Each student found and appraised a pertinent research article. Group members chose one article, representing best evidence, to present.

Librarians, clinical faculty and fourth-year students acted as group facilitators for 52 final presentations. In a development session, each facilitator received a librarian-created case evidence summary and critical appraisal of each chosen article. Classrooms of four groups, representing the four cases, were led by 1-2 facilitators. A six-part scoring rubric was developed to grade the presentations. Facilitators provided positive program feedback. Student course evaluations will provide further program assessment.

Conclusion: Partnering with academic administration and clinical faculty provided librarians with the opportunity to integrate a case-based, self-directed EBM program into the curriculum. Second-year medical students supplemented their first-year introductory EBM knowledge with practical activities that augmented their core curriculum. Fourth-year students welcomed an opportunity to lead a class as a facilitator. Clinical faculty and fourth-year students developed their own evidence and critical appraisal

skills during a development session. Students scored an average of 85% on their presentations. Facilitators reported the program as beneficial and personally rewarding. Administration deemed the program a success. Student course evaluations will be completed in May.

An edX Course on Advanced Literature Searching in the Health Sciences

Mark MacEachern, Informationist, University of Michigan, Ann Arbor, Michigan

Patricia F. Anderson, Emerging Technologies Informationist, University of Michigan-Ann Arbor, Ann Arbor, Michigan

Emily C. Ginier, Informationist, University of MI, Ann Arbor, Michigan

Tyler Nix, Informationist, University of Michigan, Ann Arbor, Michigan

Judith Smith, Informationist, Ann Arbor, Michigan

Jean Song, AHIP, Assistant Director, Academic and Clinical Engagement, University of Michigan, Taubman Health Sciences Library, Ann Arbor, Michigan

Whitney A. Townsend, Informationist, University of Michigan, Ann Arbor, Michigan

Background: A team of informationists at an academic institution partnered with local educational experts to create an edX course on advanced literature searching. The course spans the search process, from finding the right resources to reporting the process well. Through a series of educational methods (lectures, interviews) and activities, the instructors share their collected experience and expertise with the learners, who approach the course with a general interest in the process or with a specific project in mind. To our knowledge there is no other massive open online course (MOOC) that covers the search process as comprehensively as this one does.

Description: A planning team convened in 2016 to develop the course. Its initial work, spanning several months, resulted in a syllabus that consisted of eight core lessons. Each lesson included content and activity ideas, with objectives mapping directly to the overarching course objectives. This work fed the proposal that was submitted to the unit that coordinates campus MOOC initiatives. The proposal was accepted, ultimately providing the team with access to resources, technologies, and educational experts that otherwise would have been inaccessible.

The course maintains a weekly lesson structure; however, all content becomes immediately available to learners upon registration. edX provides instructors with assorted learner data, including completion rates and demographics. A survey integrated into the course provides additional data about effectiveness and applicability of content. Registration has exceeded 1000 in the three months since launch.

Conclusion: The course is ongoing, with new learners registering daily. The team hopes that each learner finds the information relevant to their search interests or needs, regardless of whether they sequentially work through all lessons or choose material more selectively. Ideally, the material will help improve the quality of advanced searches, as well as the discourse that surrounds the search process. As an open

course, it has potential for broad appeal, to both librarians and non-librarians, and especially to those who do not have easy access to search experts and professional development courses.

Crash Courses for Medical Students: Digital Research and Systematic Reviews

Rachel Vukas, Asst. Dir. of Research and Learning, Dykes Library, University of Kansas Medical Center, Kansas City, Kansas

Background: Our newly adopted medical school curriculum is designed around 9-week blocks with one week devoted to Scholarship, Enrichment, or Remediation (SER) activities. The SER week is designed to provide the medical students a break from normal studies and the opportunity to explore special interests in-depth. It also provides librarians the opportunity to provide instruction on information literacy topics not offered in the regular curriculum This presentation describes two SER week classes taught by a librarian, one on digital information research and the other on systematic reviews.
Description: The SER "week" is a three and a half-day course providing an intensive learning experience that is restricted to in-class hours. Instruction is based on active learning techniques using digital learning objects, group exercises, discussion, and software programs. The Digital Health Information Research course focuses on an in-depth understanding of research methods based partially on modules developed from the ACRL's Framework for Information Literacy for Higher Education. Internet research and resource evaluation are included as topics as well as the use of research-quality databases and grey literature. Students become adept at navigating health information in a digital environment. In the Mini-Systematic Review class, students learn about the systematic review process by participating in many of the steps in a limited manner. They are required to write a research protocol which may later be developed into a systematic review for publication.
Conclusion: Students are not graded or awarded points for the SER weeks, but are required to complete all assigned elements and keep a daily journal. Both the instructor and the students enter evaluations into the School of Medicine grading system. Emerging data from the students' journals and class evaluations suggest the content is perceived as engaging and useful by the students.

How to Elevate Your Game by Launching a Board Game Collection

Andrew Hamilton, Assistant Professor, OHSU Library, Portland, Oregon

David Forero

Background: Objective: Adding a circulating collection of board games to the holdings of a Health Sciences University Library to promote critical thinking, inter-professional interaction and collaboration skills, as well as supporting leisure activities, for university students and personnel. To acquire appropriate material for our Health Science Library collection, games featuring Social/Cooperative mechanics and Medical/Scientific themes such as Pandemic, Cytosis, Antidote, and Healthy Heart Hospital were identified to serve as the core of the collection. We partnered with other groups on campus to solicit financial support to minimize the amount of money that the Library would spend to launch the collection.

Description: The games that were identified as the core of the collection were acquired. To reduce costs, we had arranged with a local game store to receive a 25% discount for any games ordered through them. A procedure for preparing games for circulation was developed which included improving the durability of the games by adding plastic sleeves and bags for game materials, laminating instructions, and using book tape to reinforce boxes. Repacking instructions for each game were created and taped inside the lid. New circulation procedures and policies were developed and staff was trained on how to handle the new games as part of the collection. A small scale was acquired and the weight of each processed game was recorded. This weight is used to quickly determine if any element of the game is missing from the box upon check-in.
Conclusion: Our new Library Board Game Collection currently features six circulating games and a LibGuide describing the collection. We are in the process of expanding the collection to twenty five games thanks to a generous donation from our Student Life Center. Future expansion of the program will include marketing & promotion of the game collection to the our community, including hosting Game Nights at the Library; and securing financial support to purchase additional games. User Assessment will also take place to collect feedback from staff and users to improve the way the collection is viewed and utilized at our institution.

Collaborating for Classes: Training Initiatives for the Mutual Benefit of Libraries and Their Institutional Partners

Carrie L. Iwema, AHIP, Coordinator of Basic Science Services, University of Pittsburgh, Pittsburgh, Pennsylvania

Melissa Ratajeski, AHIP, Coordinator of Data Management Services, University of Pittsburgh, Pittsburgh, Pennsylvania

Ansuman Chattopadhyay, Asst Director Mol Bio Information Service, University of Pittsburgh, Pittsburgh

Background: Meeting the educational needs of researchers requires a wide variety of classes on software and specialized tools. Limitations with budget, librarian expertise, time, and staffing may result in missed opportunities for assisting patrons. A large academic health sciences library tackled this issue by collaborating with institutional partners to provide novel training opportunities for biomedical researchers. This lightning talk will briefly highlight our strategy and encourage attendees to create their own mutually beneficial partnerships.
Description: We formed three collaborations with diverse institutional partners to provide training on specialized topics for our research community. (1) An Immunology Department researcher took advantage of flexible library space as well as class registration and promotion infrastructure to teach a semester-long lecture and hands-on workshop series introducing computational flow cytometry. (2) We created a series focusing on open access software developed by institutional health sciences researchers. Invited presenters provided a brief overview on the tool development and use cases followed by hands-on practice. (3) Faculty from the Center for Research Computing taught hands-on workshops in the library on Python and R programming languages. Librarians helped tailor examples for a biomedical audience and also attended sessions to assist with basic questions.
Conclusion: All three of these initiatives promote the expertise of our collaborators and reinforce the concept of the library as a resource for free, authoritative training opportunities. Based on high attendance levels and positive feedback, these successful workshops will continue to be offered while

we seek out new class topics and additional partnerships. This strategy supports attendees by providing classes that would not otherwise be available, and is also mutually beneficial for the collaborators in terms of opportunities to promote their services and resources. Such an approach is feasible for all health sciences libraries interested in expanding their own educational offerings.

Starting a Book Club at an Interprofessional Health Sciences Campus

Allison Piazza, AHIP, Health Sciences Librarian, Seton Hall University, New York, New York

Background: The purpose of this presentation is to describe the creation of a book club at an Interprofessional health sciences campus.
Description: A book club was created to foster interprofessional discussion between three health/medical schools situated on one campus. Through discussion of health/medical texts (both non-fiction and fiction), we hope to mimic an interprofessional approach to healthcare (the coordinated care of patients by a collaborative team of health care providers). The book club encourages participation from students, faculty, and staff representing all three schools. We utilized various channels (MailChimp email campaigns, social media, electronic signs, posters) to promote the event.
Conclusion: On November 12, 2018 our Library launched the book club with a discussion of "Dopesick: Dealers, Doctors, and The Drug Company That Addicted America" by Beth Macy. Nine people attended this initial meeting and there was great conversation and positive feedback, although only one of the schools was represented. Since then, three book club discussions have taken place. As we continue, we hope to observe attendance from each of the three schools with representation from students, faculty and staff.

Piloting the Emerging Technologies Internship at a Health Sciences Library

Tariq Rahaman, Emerging Technologies & Collection Development Librarian, Martin & Gail Press Health Professions Division Library, Fort Lauderdale, Florida

Background: The Emerging Technologies Internship program aims to provide 12th grade high school students an opportunity to explore, use and develop unique applications of 3D printing/scanning, virtual reality, and digital devices. The interns work closely with library staff on student and faculty projects, but are also challenged to develop individual projects that can positively impact health sciences and medical education.
Description: The internship was developed in collaboration with a local high school and launched in September 2018. The program components include 1) goals and logistics; 2) marketing; 3) a Learning Lunch presentation for interested students; 4) application & interview phase; 5) scheduling system and modes of communication; and 6) evaluation. The interns use Trello, a web-based project management application, to view projects and keep track of scheduled tasks.
Conclusion: Our first group of interns finishes in June 2019. Students have been logging their progress twice a month and will present their projects and experiences to library staff at the end of the internship. Students have also been working on a project for their senior capstone and will present their projects as part of their graduation requirements. A final measured outcome will be timely completion of tasks and projects which will be reflected in Trello.

All Aboard! A Collaborative Orientation

Bridget Jivanelli, Medical Librarian, Hospital for Special Surgery, New York, New York

Rie Goto, Medical Librarian, The Hospital for Special Surgery, New York, New York

Christopher DeFrancesco

Karla J. Felix, Director, GME Curriculum and Evaluation, Hospital for Special Surgery, New York, New York

Peter Fabricant

Background: Due to increasing demands on surgical skill development, all incoming orthopaedic residents participate in a surgical skills boot camp which began June 2014. While this program is successful at providing introductory orthopaedic surgery skills, the length of other orientation programs was shortened, specifically, the medical library. Therefore, to increase the knowledge base to the medical library's offerings, a personalized re-orientation session for residents will be introduced 8 to 10 months following the initial orientation. This allows the resident to seek for relevant and deeper questions as they begin to formulate research ideas, develop content for teaching and prepare for cases.
Description: When discussing new resource marketing strategies, the Library Task Force presented a follow up orientation for PGY1 residents. A resident task force member proposed the structure for the re-orientation session. This session is a collaborative effort with a librarian and a resident presenting. It including a pre-test on locating resources, an interactive lecture on services, academic resources, scholarly communication, tips from an experienced resident and a post-test. During this session, IT personnel will be on hand to troubleshoot problems, make sure the resident's laptop is up to date, and facilitate requests.
Library staff worked with a resident task force member, a faculty member and the GME Curriculum and Evaluation Director to determine the best time to revisit library resources. Re-orientation sessions will take place for individual residents during a rotation halfway through their first year allowing time to get acclimated.
Conclusion: This second orientation will allow time to foster a closer relationship with the new residents.
We plan to implement this program over the coming months and will present findings at the conference.

Elevating Health Literacy Education with a Medical School Selective

Cynthia Beeler, AHIP, Academic Medical Librarian, Mayo Clinic Libraries, Rochester, Minnesota

Tara Brigham, Assistant Professor of Medical Education & Medical Librarian, Mayo Clinic, Jacksonville, Florida

Lisa A. Marks, AHIP, Director of Libraries, Mayo Clinic Libraries, Arizona, Scottsdale, Arizona

Background: A new librarian in the medical school library had previously taught a health literacy selective for fourth year medical students. Upon beginning work in a new setting, it became clear that a health literacy selective for first and second year students would be beneficial. In cooperation with librarians from the other campuses, the content and format was modified to fit the selective requirements and provide a comprehensive overview of health literacy for medical students.
Description: Once the decision was made to move forward in offering a one-week health literacy selective through the libraries, the librarians virtually met to discuss objectives, structure, and requirements. The proposal was submitted to the selectives committee, and they needed more information. After a second proposal was questioned, one librarian attended a committee meeting and described the selective in detail. It was then approved by the committee. A LibGuide was prepared to inform students of the requirements and how to sign up. Students were able to sign up beginning on December 1. As of March 15, four students have completed the selective, and three more are signed up for a week in March. Each student meets with the librarian at their location after the selective, and comments were noted about what worked best and what will need tweaking in the future.
Conclusion: The selective requires students to submit two essays. One is a reflection on how the health literacy knowledge gained will impact their future practice. The other is a reflection on interviews conducted with family or friends about when they didn’t understand a health care professional. They also have assigned readings and videos. Students said the interviews were eye-opening and two said they were their favorite part of the selective. Students enjoyed the videos, and one disliked the readings. In the future, we may offer the students options for more videos or more readings while keeping the core content the same.

The Boot Camp: An Effective Measure to Teach Information Literacy to Graduate Students

Mirko von Elstermann, Biomedical Sciences Librarian, University of Iowa, Iowa City, Iowa

Janna C. Lawrence, AHIP, Deputy Director, Hardin Library for the Health Sciences, University of Iowa, Iowa City, Iowa

Background: Teaching information literary (IL) to graduate students poses a challenge for libraries. Notably, science graduates are under strong time constraints, among them lab work, attending classes, and writing reports or publications. On the other hand, IL competencies, except for basic knowledge of statistics, are usually not part of the curriculum of graduate programs. In this lightning talk, we give a snapshot of a comprehensive instruction format that promotes graduate students’ IL competencies and fosters library outreach to this patron group.
Description: We set up a series of IL workshops packed into one day (a format called Boot Camp) within an academic health sciences library that serves colleges of medicine, dentistry, pharmacy, and nursing, among other health professions. The target group of the workshops were graduate students of all colleges, in particular approximately 350 PhD students within biomedical departments of a college of medicine. Our idea was that the workshops should teach essential IL competencies from the start to the end of a PhD project.
Conclusion: When we offered the Boot Camp the first time, we did not understand that it would be necessary to be in frequent communication with the students who signed up for the event, so few registrants actually attended. In addition to better communication, the second time the Boot Camp was offered we also emphasized the ‘logical’ order of the workshops—searching the literature, reference

and data management, scientific writing, publishing (with an emphasis on Open Access), and requirements for thesis submission. The liaison librarian introduced the event himself when we offered it last time, which may have increased attendance and also attracted graduates from 'difficult' areas (e.g., biochemistry). In sum, our Boot Camp contributed to closing the gap between the library and the graduates at our institution.

Data Needs during Infectious Disease Outbreaks

Sarah C. Clarke, AHIP, Associate Fellow, National Library of Medicine, Bethesda, Maryland

Stacey J. Arnesen, Chief, Disaster Information Management Research Center, National Library of Medicine, Bethesda, Maryland

Siobhan Champ-Blackwell, Librarian, National Library of Medicine/Disaster Information Management Research Center, Bethesda, Maryland

Objectives: Biomedical libraries can play a vital role in managing health information during public health emergencies. Helping organizations be prepared is critical for response efforts on the part of health care providers and emergency responders. A literature review and interviews with federal and organizational partners will shed light on data needs and barriers during infectious disease outbreaks and address how librarians may provide assistance.
Methods: A search of the literature regarding data needs during infectious disease outbreaks was performed in Disaster Lit, PubMed, and Web of Science, with supplemental searching in Google; citations were managed in EndNote. Barriers to rapid data sharing were identified in the literature and used to curate interview questions. Additional questions include the exploration of librarian involvement to assist in the management of disaster data and discoverability. The Disaster Information Management Research Center (DIMRC) team provided a list of key agencies and staff to interview. Eleven interviews were conducted over the phone.
Results: Reoccurring themes surrounding data barriers were exposed. Awareness: work is being duplicated across organizations due to a lack of knowledge and coordination. Policy: data sharing policies and data use agreements take time to implement. Quality and timeliness: quality of data is a concern because standards are not widely adopted. Data shared may not be peer-reviewed due to a push to rapidly share, therefor this data should address limitations. Recognition: there is hesitancy to share data prior to publishing findings due to a fear of scooping. Additionally, many who reuse shared data fail to provide proper credit. Furthermore, there are often no promotional or tenure incentives to share data.
Conclusions: There are ways librarians can immediately assist with data sharing needs during outbreaks. First, libraries could offer research data management services. Secondly, libraries can provide toolkits for responders and researchers; these toolkits could include links to grey literature in the Disaster Lit® database, a summary of response efforts across organizations, tips for relationship building, data use agreement guidelines, available datasets and databases on the web, and instructions for proper data citation. Finally, librarians should remain up to date on current outbreaks and new trends; ProMED-mail, HealthMap, and the Disaster Outreach Lib listserv are useful resources. Having services and resources in place prior to an infectious disease outbreak will assist in decreasing barriers in data sharing and better allow researchers and responders a more rapid and efficient response.

Elevate Liaison-ship: Uncommon Relationships with the Library

Ayaba Logan, Research and Education Informationist, Medical University of South Carolina, Charleston, South Carolina

VeLonda Dantzler

Background: As Librarians, we want to support as many people, departments, and areas on our campus. Yet we tend to write about only the common or main areas like the schools or college of Health/Medical Sciences. Human Resources is a seemingly uncommon department to support, especially for an Academic Health Science Center. The purpose of this abstract is to describe the forging of a new relationship and liaison area with the Human Resources department and how we plan to evaluate this relationship.
Description: Human Resources department on any campus, organization, or institution is extremely important, like the library. Without either department, academic organizations cannot function well or properly. And similarly, sometimes we are overlooked when decisions are made. At this Academic Health Science Center, we have joined forces to support the professional development of the enterprise, not just the clinical staff. HR felt like we as an institution needs to do more to support the career development of all its personnel. Out of that passion and hunting for a solution, she discovered UC Berkely's "Career Counseling Library". Thus the beginning of a beautiful relationship was born. I as the newly minted HR Liaison was concerned; I was not sure if we had resources to support career development for everyone. I was wrong, we had tons of career development resources.
Conclusion: One result of the relationship was to develop a LibGuide that can be updated regularly and display usage statistics. The LibGuide went live January 14, 2019. It has already been publicized in the HR newsletter and viewed 130 times to-date. The next step is to discuss a larger evaluation of the LibGuide and this uncommon relationship.

The Goldilocks Method: Demonstrating Your Value in Small, Medium, and Large Bites

Heather J. Martin, AHIP, Director, System Library Services, Providence St. Joseph Health, Portland, Oregon

Background: In an era of financial constraint hospital libraries are facing closer scrutiny and must prove their value in order to survive. Leadership at a large health system library found the traditional written annual report lacked the impact needed to capture the attention and the imagination of administration. Taking different approaches over the years, this library has found a solution that is “just right”, creating three very different documents – small, medium, and large – to demonstrate its worth.
Description: Library leadership moved away from the traditional written annual report toward a one-page infographic that depicted the library’s scope, scale, and impact. This visual representation of library statistics captured the attention of administration and patrons in a new, compelling way. As it didn’t allow for telling deeper stories of special projects and partnerships, the library later added back in a written supplement and a “Selected Thanks” section that directly quoted satisfied library patrons. But, when the library’s very existence was at stake, the need for a much more comprehensive value-case was necessary, and a 20-page white paper tied to the organization’s strategic plan and key initiatives was

created. Providing an annual report in 3 different formats – small, medium, and large, allows the library to tell just the right story, to the right person, in the right place.
Conclusion: Reporting on library usage statistics, initiatives, and successes in different formats has allowed the health system library to tell its story in just the right way at the right time. The posted infographic provides a lasting snapshot that draws people in. As such, it has been viewed by far more people than reports in the past. The written supplement tells stories from our patrons, and is delivered to key leadership. The value-case effectively tied ALL the work the library does directly to the institution's strategic goals, and this past year effectively convinced senior leadership to stave off cuts.

Sunday, May 5, 2:00 p.m.–3:25 p.m.
Room: Grand Ballroom B (East Tower, Ballroom/Gold Level)

Professionalism & Leadership Immersion Session 1

Moderator: Nisha Mody

Clueless at MLA: New Member Immersion Session

Nisha Mody, Health & Life Sciences Librarian, UCLA, Los Angeles, California

Keith Engwall, AHIP, Web & Emerging Technologies Librarian, Oakland University William Beaumont School of Medicine, Rochester, Michigan

Nisha Mody, Health & Life Sciences Librarian, UCLA, Los Angeles, California

Erin E. Reardon, Reference Librarian, LSU Health Shreveport, Shreveport, Louisiana

Kelsa Bartley, Manager, Library Services: Learning, Research & Clinical Information Services, Louis Calder Memorial Library, University of Miami Miller School of Medicine, Miami, Florida

Jahala Simuel, Medical Librarian and Head of Access Services, Louis Stokes Health Sciences Library, Washington, District of Columbia

Alexandria Leigh Brackett, AHIP, Clinical Librarian, Cushing/Whitney Medical Library, Yale University, New Haven, Connecticut

Alice Jean Jaggers, Outreach Coordinator, UAMS, Little Rock, Arkansas

Siobhan Champ-Blackwell, Librarian, National Library of Medicine/Disaster Information Management Research Center, Bethesda, Maryland

Jolene M. Miller, AHIP, Director, University of Toledo, Mulford Health Science Library, Toledo, Ohio

Nisha Mody, Health & Life Sciences Librarian, UCLA, Los Angeles, California

Stacey Arnesen, Chief, Disaster Information Management Research Center, Specialized Information Services, Bethesda, Maryland

As a SIG, we have observed that newer members are overwhelmed with information about MLA as an organization and the many types of events during the MLA Annual Meeting. We would like to address

these concerns proactively with a welcoming Immersion Session. We plan to split this session into two parts.
The first part of the session will consist of a 20-minute presentation about MLA Basics: (1) brief look at MLA organizational structure (2) conference events and format, (3) getting involved during conference, (4) self-care during the conference
The second part of the session will be a 70-minute block of roundtable discussions where members can (but are not required to) rotate through five stations for 20 minutes each (with transition buffers).
Members: (1) early career questions (2) mentorship (3) diversity & inclusion (4) AHIP (5) Specializations (Consumer Health Information and Disaster Information).

Sunday, May 5, 2:00 p.m.–3:25 p.m.
Room: Columbus AB (East Tower, Ballroom/Gold Level)

Professionalism & Leadership Immersion Session 2

Moderator: Angela Spencer, AHIP

Searching Clinically: How to Search, Find, and Assess Information at the Point of Care, Part 1 Research

Angela Spencer, AHIP, Manager, Medical Library, St. Lukes Hospital, Chesterfield, Missouri

Helen-Ann Brown Epstein, AHIP, FMLA ,Informationist, Health Sciences Library, Mt Laurel, New Jersey

Sarah L. Carnes, AHIP, Clinical Librarian, Veterans Health Administration, Bedford, Massachusetts

Timothy Kenny ,Clinical Library Manager / Senior Librarian, UNT Health Science Center / JPS Health Network, Arlington, Texas

Hannah Rutledge, AHIP, Head of Clinical Informationist Services, Emory University, Atlanta, Georgia

Shawn Steidinger, AHIP, Assistant Librarian for Clinical Services, University of Utah, Salt Lake City, Utah

Angela Spencer, AHIP, Manager, Medical Library, St. Lukes Hospital, Chesterfield, Missouri
Working with health care teams at the point of care requires clinical awareness and the ability to quickly locate and assess case-relevant information. Through presentations, demonstrations, group activities, and discussion, you will learn about and practice how to quickly meet clinician information needs. This exceptional, double session is for librarians who work in a clinical setting, participate in rounds, or provide instruction at point of care.
Part 1 focuses on identifying questions and key elements in case discussions, conducting “rapid reference” interviews, searching at the point of care and with urgency, quickly assessing articles, developing clinically embedded librarian roles in rounds and case conferences, and contributing to electronic health records.

Sunday, May 5, 4:30 p.m.–5:55 p.m.
Room: Columbus CD (East Tower, Ballroom/Gold Level)

Clinical Support Session 1

Moderator: Margaret Henderson, AHIP

Connecting Consumer Health Information with Clinical Care

Hannah F. Norton, AHIP, Reference & Liaison Librarian, University of Florida, Gainesville, Florida

Margaret Emily Ansell, AHIP, Nursing & Consumer Health Liaison Librarian, Health Science Center Libraries, University of Florida, Gainesville, Florida

Matthew Daley

Mary Edwards, Reference & Liaison Librarian, University of Florida, Gainesville, Florida

Jane Morgan-Daniel, AHIP, Community Engagement and Health Literacy Liaison Librarian, University of Florida, Gainesville, Florida

Background: Accessing, navigating, evaluating, and using health information are essential skills for all healthcare consumers. To improve information-seeking behaviors and knowledge of consumer health information resources among patients at a large academic health system, a team of librarians and IT staff are creating and disseminating brief educational videos for distribution through Emmi, an existing patient education video resource. Videos focus on specific consumer health information resources including MedlinePlus, skills such as evaluating online health information, and local resources and services available through the health science and public libraries.
Description: The project consists of five work phases: drafting videos; expert panel review; revising, recording, and uploading videos; promoting videos to healthcare providers; and assessment. Using a successful model from previous outreach projects, an expert panel will review draft content before videos are recorded to ensure their accuracy, clarity, and effectiveness; the panel consists of a physician, professor of education and pediatrics, nurse educator, clinical social worker, and homecare staff. Once finalized, videos will be uploaded to Emmi, where they can be assigned directly to patients by their healthcare providers. The team will promote these videos through presentations to a variety of clinicians across the health system, initially focusing on hospitalists and large primary care services. Assessment metrics include the number of times videos are assigned to and viewed by patients and patients' responses to Emmi's standard post-video satisfaction survey.
Results and Conclusions: It is anticipated that using embedded videos assigned to patients at the point of healthcare need will be an effective method for disseminating patient education about finding, evaluating, and using health information. Measures of success will include reach of the videos (number assigned and number watched) and patient satisfaction with the videos. Longer-term impacts, such as patient engagement and questions for healthcare providers, may be assessed after project completion through interviews with healthcare providers who assigned the videos to patients.

Connecting Systemic Diseases and the Oral Health of Children: Helping Families Understand the Relationship

Julie H. Schiavo, AHIP, Assistant Librarian / Assistant Director for Dental Library Services, Louisiana State University Health Sciences Center - New Orleans, New Orleans, Louisiana

Wesley Lucas, Reference Librarian, Louisiana State University Health Sciences Center - New Orleans

Priyanshi Ritwik

Background: This paper describes the collaboration between Louisiana State University Health Sciences Center, New Orleans librarians and the pediatric dentistry department to develop a website containing accurate health information in a user-friendly format on the relationship between systemic diseases and the oral health of children in the hope that having this information will educate and encourage parents to seek dental care for their children with systemic diseases. Information on the oral aspects of systemic diseases in children is often very difficult for a consumer to find. What little is available is usually written for health professionals in scientific journals and is neither accessible nor comprehensible to the average consumer.
Description: This project was funded by an Express Outreach Award from the National Network of Libraries of Medicine / South Central Region (NNLM/SCR). The LSUHSC-NO Pediatric Dentistry and library faculty choose ten health conditions based on needs assessments of their patients and a 2015 community needs assessment to become the basis of a mobile friendly accessible website. The content of each topic is based on the best evidence and written at an easy to read manner and a printable version of each topic is available. Materials will be tested for usability and edited based on feedback from patients' parents or guardians and health professionals. The site will be submitted to several consumer health websites and agencies for possible inclusion.
Conclusion: Health professionals and consumers may not be aware of the impact of a disease on oral health or the effect of poor oral health on systemic conditions. This project will create and make available such information allowing for health professionals and consumers to better address the oral-systemic health connections of the patient. Health care providers will be able to use this as a resource for anticipatory guidance for oral care specifically pertinent to the systemic condition of their patient. Parents who are aware of the connection will also be more likely to seek dental care for their children.

Elevating Patient Education through State-Wide Instruction of Nurses and Patient Educators on Consumer Health Resources

Lindsay E. Blake, Clinical Services Librarian, University of Arkansas for the Medical Sciences, Little Rock, Arkansas

Alice Jean Jaggers, Outreach Coordinator, UAMS, Little Rock, Arkansas

Background: Patient Education is consistently cited by the Joint Commission as needing work at many institutions. However, with a limited number of patient educators and high nurse to patient ratios it is

hard to find the time to adequately teach patients all they may need to know. The goal of this project was to create a educational course for nurses and patient educators covering authoritative, freely available online resources and an online guide of these resources that could be accessed by health care educators and/or given to patients to explore on their own.
Description: The Clinical Services Librarian and Outreach Coordinator at an academic health sciences center proposed a plan to educate the nurses and patient educators in the hospital on reliable consumer health resources. Partnerships were formed within the institution with patient educators, nurses, patient advocates, and patient- and family-centered care. Librarians created a course presenting general consumer health resources from the National Library of Medicine as well as other reliable sites. The course also looked at patient education resources for drugs and supplements, alternative and complementary health, genetics, and website evaluation. The course was granted nursing, dietitian, and certified health education specialist (CHES) continuing education credit. Parallel with the course, an online resource was built using LibGuides software. The guide contains information on both general and specific health issues, based on local needs. During building of the guide a section was also added for all resources available in Spanish, as well as En Espanol links added to all resources with Spanish language links available. The course walks participants through the guide and was piloted in local hospitals with nurses, dietitians, and patient educators before classes were arranged around the state.
Conclusion: The online guide was completed in July 2018. As topics were suggested at courses, they were added to the guide, including sections on women's health, and children, and veterans. The course and guide were well received. Results from pre- to post-tests showed an average increase of 20%. Evaluations of the course and instructors were consistently ranked average or above average including: having a better understanding of subject materials and ability to identify, navigate, and analyze resources. Classes were taught in the spring with hospitals and organizations both local and from around the state.

Criteria for Evaluating Deception, Disinformation, and Controversy in the Evolving Digital Consumer Health Information Universe Research

Alla Keselman, Senior Social Science Analyst, US National Library of Medicine, Bethesda, Maryland

Catherine Arnott Smith, Professor, University of Wisconsin-Madison, Madison, Wisconsin

David R. Kaufman, Associate Professor, Arizona State University, Scottsdale, Arizona

Objectives: With the emergence of new Web platforms and social media, critically evaluating online health information has taken on a new urgency. Unfortunately, existing information quality criteria address do not address content, target information creators rather than users, or do not address novel technologies. This study develops a methodology for analyzing health-related webpages and applies it to a set of webpages.
Methods: This qualitative study analyzes twenty-five type 2 diabetes pages derived from the results of a Google search for “diabetes, reversal, natural.” The coding scheme, developed via a combination of theory-driven and data-driven approaches, includes five categories from existing established guidelines (resource type, information authority, validity of background information sources, objectivity, currency) and seven novel categories (treatment/reversal method, promises and certainty, criticisms of establishment, emotional appeal, vocabulary, rhetoric and presentation, use of science in argumentation). The coding involves both categorical judgement and in-depth narrative

characterization. Upon establishing satisfactory level of agreement on the narrative coding, the team coded the complete dataset of twenty-five pages.
Results: Treatments proposed by the pages include a mixture of conventional evidence-based treatments (e.g., healthy balanced diet) and unconventional treatments (e.g., dietary supplements). Most pages either promise or strongly imply high likelihood of complete recovery. Pages vary greatly with respect to the authors' stated background and credentials and the information sources they reference. The majority includes criticisms of the traditional healthcare establishment. Many sell commercial products. A significant number makes positive mentions of the word "cure" and includes references to nature as a positive healing force. Most pages present biological explanations of their proposed treatments of varying levels of complexity.
Conclusions: Both traditional and data-driven categories of codes used in this work yield insights about the resources and highlight challenges faced by their users. This exploratory study underscores the challenges of consumer health information seeking and the importance of assisting the public with seeking, evaluating, and analyzing consumer health information in the changing digital ecosystem.

The Information Sources and Preferences of Patients Diagnosed with Rare Cancers Research

Dana L. Ladd, AHIP, Community Health Education Center Librarian, Tompkins-McCaw Library for the Health Sciences, Lanexa, Virginia

Objectives: Patients diagnosed with rare cancers face many challenges including difficulty finding reliable consumer health information. Little research exists regarding the information sources used and source preference of patients with rare cancers. The research questions are:
What sources do patients use to find information about rare cancers?
What sources do patients prefer when seeking information about their rare cancers?
Methods: This cross-sectional design study used validated measures to assess the information sources and preferences of patients diagnosed with rare cancers (n=113). Patients were contacted via mailed survey following the Tailored Design Method. Eligible patients were those who were 18 years and older and had been diagnosed with rare cancers of any type or stage being treated at a large National Cancer Institute (NCI) designated cancer center in the past 12 months. Survey questions were taken from the Health Information and National Trends Survey and assessed patients' use and and patients' source preferences of books; brochures and pamphlets; cancer organizations; family, friends, and coworkers; doctors and healthcare providers; Internet; library; magazines; newspapers; telephone information number; complementary, alternative, or unconventional practitioner; and other sources.
Results: A total of 199 patients were identified as having a rare cancer and were sent a mailed survey. Of those, 113 surveys were returned resulting in a 56.7% overall response rate. Nearly all (93.8%) reported searching for health information from any source. Participants reported preferring information from their health care provider (80.5%); however, participants reported using the Internet (80.9%) over all other sources of health information.
Conclusions: This study fills an important gap in the rare cancer information sources literature. Identification of source use and preference is important because patients are reporting that they are not receiving information from their preferred source of information but are turning to the Internet to meet their information needs. Healthcare providers and information professionals need to be able to direct patients to reliable sources consumer-oriented websites for information about their rare cancers.

Sunday, May 5, 4:30 p.m.–5:55 p.m.
Room: Grand Ballroom A (East Tower, Ballroom/Gold Level)

Education Immersion Session 2

Moderator: Kathryn Houk, AHIP

Activate Your Evidence-Based Practice Instruction!

Kathryn Houk, AHIP, Medical Education Librarian, University of Nevada Las Vegas, Health Sciences Library, Las Vegas, Nevada

Stephanie M. Swanberg, AHIP, Associate Professor, Information Literacy & eLearning Librarian, Oakland University William Beaumont School of Medicine, Rochester, Michigan

Hanna L. Schmillen, AHIP, Health Sciences & Professions Librarian, Ohio University, Athens, Ohio

Laura Menard, Assistant Director for Medical Education and Access Services, Indiana University, Indianapolis, Indiana

Kathryn Houk, AHIP, Medical Education Librarian, University of Nevada Las Vegas, Health Sciences Library, Las Vegas, Nevada

Kathryn Houk, AHIP, Medical Education Librarian, University of Nevada Las Vegas, Health Sciences Library, Las Vegas, Nevada

This immersion session will share pedagogical approaches for creating active learning activities for evidence-based practice (EBP) instruction. Participants will learn from experienced panelists about different instruction modalities and then have the opportunity to discuss and practice designing EBP activities in small groups. Activities developed during the breakout session can be applied at participants' own institutions or used to help reimagine instructional approaches for EBP concepts. Panelists will have 40 minutes for brief presentations and Q&A, followed by a 5-minute break, and then participants will have 45-50 minutes for guided practice and discussion. Session participants are strongly encouraged to bring an example of a current teaching approach and a laptop or iPad for the interactive portion. If you prefer pen and paper, please bring a hard surface to write on.

Sunday, May 5, 4:30 p.m.–5:55 p.m.
Room: Columbus GH (East Tower, Ballroom/Gold Level)

Information Management Session 2

Moderator: Jessica Koos, AHIP

Using an Electronic Lab Notebook System to Promote Data Management Plans at an Academic Medical Center

Erin D. Foster, Data Services Librarian, Indiana University School of Medicine, Indianapolis, Indiana

Elizabeth C. Whipple, AHIP, Assistant Director for Research and Translational Sciences, Indiana University School of Medicine, Indianapolis, Indiana

Heather L. Coates, Digital Scholarship & Data Management Librarian, IUPUI, Indianapolis, Indiana

Background: At an academic medical center, the rollout of an electronic lab notebook (ELN) system includes tailored support to develop functional data management plans for participating research labs. The office of research is sponsoring the adoption of the ELN and, while administrative drivers include improving lab efficiency and reducing institutional liability, the pilot primarily focuses on the benefits of ELN use. In particular, improving the collection of and access to experimental information for research projects. The creation of data management plans, in conjunction with the ELN adoption, allows participants to engage more holistically with the research processes of their laboratories.
Description: Nine research labs are identified as “early adopters” for the 4-month pilot of the selected electronic lab notebook system. As participants in the pilot, these labs are expected to a) attend training on the ELN system and b) evaluate their current information and data management practices. To ensure these expectations are met, the institution's ELN implementation team meets with each lab prior to conducting the ELN training to discuss how they manage (i.e., organize, store, and share) research data. These consults result in a draft data management plan that reflect the labs’ current research data management processes and provides suggestions for potential areas of improvement/streamlining. The training on the functionality of the ELN follows this to encourage participating labs to develop concrete strategies for integrating the ELN into the research data/information management processes of the lab overall.
Conclusion: As of this submission, the authors have worked directly with four of the “early adopter” labs (each with 6-10 members) to review their information and data management practices while onboarding them to the ELN. These labs receive an individualized data management plan in addition to training on the ELN. A primary outcome is to complete this process with the remaining five “early adopter” labs while gathering feedback on the ELN system itself and monitoring the adoption success of the ELN in these labs. A secondary outcome is to “scale up” this program for full rollout to the institution in 2019.

Developing Workflows to Facilitate the Sharing of Research Datasets from Electronic Health Records

Nicole Contaxis, Data Catalog Coordinator, NYU Health Sciences Library, New York, New York

Michael Cantor

Alexander Bragat

Kevin Read, Lead, Data Discovery and Data Services Librarian, NYU Health Sciences Library, New York, New York

Background: Electronic Health Record (EHR) systems provide opportunities to leverage clinical data for research purposes. Yet, at an academic medical center, researcher awareness of centralized resources to access clinical data is limited. Librarians and a clinical data management team (CDMT) collaborated to increase the awareness of institutional systems and the transparency of research practices by indexing datasets and access procedures in an institutional catalog. Librarians and the clinical data team worked to integrate workflows, share administrative information, prevent redundant efforts, increase the discoverability of EHR data for research purposes, and convince leadership to update institutional policy to support the ongoing collaboration.
Description: Librarians and the CDMT began the project by establishing workflows to share information about EHR data requests. The librarians initiated a pilot phase of the project, which included connecting with researchers who had pulled EHR data and cataloging that data for other researchers to be able to re-request. Thirteen new dataset records were created through the pilot. Librarians presented the pilot to research leadership with the intent to argue for a policy that requires all EHR data requests processed by the CDMT to be indexed in the institutional data catalog. Research leadership approved the proposal and added the policy to the CDMT data request form. Librarians then indexed all past EHR data requests, making this valuable research data discoverable across the institution. The library and the CDMT continue to collaborate to index future data requests.
Conclusion: At the time of writing, there are 165 EHR datasets indexed in the institutional catalog. Furthermore, workflows have been completely integrated between the library and the CDMT. Key team members have permission to access digital records from both groups and are able to ensure that effective communication and collaboration continues. Additional outcomes to be measured moving forward include: (1) access statistics for EHR datasets in the institutional catalog; (2) the number of new EHR datasets added to the catalog; (3) the number of publications resulting from EHR datasets in the catalog; and (4) an evaluation of eliminated redundancies.

Creating Institution-Specific Resources on Data Transfer and Data Sharing

Nicole Contaxis, Data Catalog Coordinator, NYU Health Sciences Library, New York, New York

Lee Taylor

Alisa Surkis, Assistant Director, Research Data and Metrics/Vice Chair for Research, NYU Health Sciences Library, New York, New York

Kevin Read, Lead, Data Discovery and Data Services Librarian, NYU Health Sciences Library, New York, New York

Background: Librarians identified researcher confusion about institutional data sharing policies and supported data transfer technologies through regular library consultations at an academic medical center. Researchers voiced concerns about de-identification, the need for data sharing agreements, data ownership, and tools to transfer both protected and non-protected data. To address a lack of transparency about institutional data policies, a librarian partnered with General Counsel and Research IT to develop resources and classes to render institutional data sharing policies and technology more discoverable. The process of building these resources coincided with collaborative efforts to update institutional data transfer policies and practices.
Description: Librarians initiated a collaboration with General Counsel to address a lack of researcher awareness about data sharing and transfer policies. Librarians and General Counsel took several approaches to solve the issue: (1) convene a working group, (2) develop and co-teach a class on data transfer at the institution; (3) create a guide about institution specific policies and supported tools. The working group included staff and leadership from Research IT, the Office of Science and Research, and Basic Science Operations. The class on data transfer included lectures on policies and tools as well as case studies from the institution's history. The guide includes an interactive tutorial to guide researchers through institutional procedures for sharing their research data as well as information on data de-identification, repositories suitable for data sharing, and how to make their data discoverable via the institution’s data catalog.
Conclusion: Evaluation forms were collected at the end of the data transfer class. All attendees said that they would use the class material in their work, and all attendees said that they would recommend the class to others. Furthermore, all attendees said that they would be interested in classes on related advanced topics. Additional outcomes that will be measured include usage statistics for the online guide on institution specific policies, integration of the online guide into institutional workflows, and ongoing class evaluation data.

From Conception to Action: Elevating Library Projects through Collaboration Between Librarians and Developers

Ian Lamb, Solutions Developer, NYU Health Sciences Library, New York, New York

Joel Marchewka, Web Applications Developer, Health Sciences Library System / Digital Library Services, Pittsburgh, Pennsylvania

Jean-Paul Courneya, Bioinformationist, Health Sciences and Human Services Library, Baltimore, Maryland

Kevin Read, Lead, Data Discovery and Data Services Librarian, NYU Health Sciences Library, New York, New York

Background: The Data Catalog Collaboration Project (DCCP) is a multi-institutional venture aiming to unlock the true value of research data by making datasets easily findable and reusable. The project has a technical component consisting of an open-source search engine and user interface for metadata curation, as well as a human component: the collaboration both within and between institutions. Librarians and developers work on an equal footing to suggest new directions and innovations to continually improve the catalog. The DCCP leverages several strategies and technologies to improve dataset visibility, and many fruitful innovations have arisen from the collaboration between developers and librarians.
Description: Librarians and developers each bring their own expertise to the table, and the fusion of these viewpoints has been valuable. Initially, librarians suggested the use of Linked Open Data, and developers advocated for using the Schema.org vocabulary; this combination has enabled our metadata to be harvested by Google's new Dataset Search tool, which now prominently displays datasets from our catalogs. A new application programming interface (API) allows librarians to provide developers with a CSV file of curated metadata which can be ingested in bulk, allowing rapid growth of the catalog. Librarians suggest tweaks to the metadata schema to accommodate new use cases, which are then implemented by developers. And developers suggested a novel way for librarians to provide a temporary link to researchers so they can approve the way their dataset appears in the catalog, before it goes live.
Conclusion: Librarians and developers established a streamlined workflow for metadata curation which, combined with using the API to batch ingest dataset records, enabled DCCP institutions to increase efficiency when adding records. The creation of temporary links allows librarians to distribute pre-published records to researchers so they can view their datasets in context before approval. Finally, records from DCCP catalogs now appear in Google Dataset Search, which has drastically increased the number of daily visitors and pageviews. This project demonstrates that collaboration between librarians and developers where both parties work on equal footing can provide high value outcomes for specific library projects.

Design of a Data Catalog to Support Discovery, Acquisition, and Use of Data Sets in a Secure Research Environment

Michael Eliot Bales, Research Impact and Evaluation Informationist, Weill Cornell Medicine, New York, New York

Peter Robert Oxley, Associate Director of Research Services, Weill Cornell Medicine, New York, New York

Terrie R. Wheeler, Director, Weill Cornell Medicine, New York, New York

Background: Since 2015, our medical school's library has supported a secure computational environment, known as Data Core, which allows researchers to work collaboratively on projects involving sensitive research data. As of October 2018 the Data Core supports 20 researchers spanning 38 active projects and a total of 41 datasets governed by data use agreements (DUAs) with external agencies, including the Centers for Medicare and Medicaid Services. Data Core users have recently expressed interest in a searchable online system that would allow for discoverability of relevant research data sets and provide information relevant to data governance, acquisition, and use.

Description: In response to these conversations we have begun developing a data catalog with online search functionality. To create a data dictionary for the catalog we first selected applicable terms from the Dublin Core Metadata Initiative (DCMI) and the Data Catalog Vocabulary (DCAT), an RDF vocabulary designed to facilitate interoperability between data catalogs published on the Web. We supplemented this list with data elements reflecting institution-specific data governance considerations – for example, whether an additional attestation must be submitted for each new user, and the names of other principal investigators who have previously used a given data use agreement (DUA).
Conclusion: We are in the process of populating the Data Catalog with metadata on existing internal and external data sets and DUAs, and are developing a structured plan to make the Data Catalog searchable via searches of indexed fields as well as via a full-text search of metadata.

Sunday, May 5, 4:30 p.m.–5:55 p.m.
Room: Columbus AB (East Tower, Ballroom/Gold Level)

Information Services Session 2

Moderator: Elizabeth Laera, AHIP

Reinvigorating the Open Access Movement at Johns Hopkins University and Medicine

Caitlin A. Carter, Scholarly Communication and Open Access Policy Fellow, Johns Hopkins Medicine, Baltimore, Maryland

Robin Sinn, Coordinator, Office of Scholarly Communication, Johns Hopkins University, Baltimore, Maryland

Claire J. Twose, Associate Director, Johns Hopkins University, Baltimore, Maryland

Anne K. Seymour, Director, Welch Medical Library, Baltimore, Maryland

Background: In July 2018, the new institutional Open Access Policy went into effect. In support of the new policy the libraries also launched a new submission system for faculty to deposit research into the institutional repository and PubMed Central simultaneously. By aligning these workflows, librarians can more easily promote the policy to faculty, who can more efficiently comply with the institution's policy and the NIH Public Access policy. We believe raising awareness about the policy and new submission system reinvigorates the Open Access movement, can foster greater health equity, and promote more sustainable community-based infrastructure throughout the academic health research landscape.
Description: Developing
A year-long discussion around an Open Access policy made clear that campus stakeholders were united in requesting that no extra burden be added to their workflows.
Implementing
The libraries developed two initiatives to support faculty and administrators in their compliance with the new policy. These initiatives reduced burden on faculty while also encouraging greater access to the institution's health and medical research. The libraries developed a new submission system that allows researchers to comply with the institution's policy and NIH Public Access requirements via a single deposit. The libraries also added two positions to provide education and support around the policy.
Evaluating
Librarians record basic statistics, including outreach efforts, presentations, questions answered. Submission of files in compliance are tracked. Several different tools will be used to assess the uptake across faculty and compared with other universities.
Conclusion: Medical libraries can use policies and community-based workflow tools to inspire faculty, staff, and students to open their research to the world, thus supporting health equity and patient advocacy. These conversations will also educate researchers about trends in publishing and research

evaluation, opening up systems that are inherently closed. Librarians are core to this initiative and need to use their trusted position to move openness forward.

Bibliometric Benchmarking of Pediatric Cardiovascular Centers

Cathy Sarli, AHIP, Senior Librarian, Evaluation Services, Washington University, Saint Louis, Missouri

Evan Sprague, Research and Publishing Support Librarian, Bernard Becker Medical Library, Washington University in St Louis, St Louis, Missouri

Pirooz Eghtesady, Chief, Section of Pediatric Cardiothoracic Surgery, Washington University/St. Louis Children's Hospital, St Louis, Missouri

James Zerkel, Library Assistant, Washington University School of Medicine, St. Louis, Missouri

Angie Rosengarten

Amy Suiter, Scholarly Publishing Librarian, Bernard Becker Medical Library, St. Louis, Missouri

Background: A specialized pediatric cardiovascular center on campus approached the library to gain insight as to how their scholarly productivity and impact compared to similar centers from other institutions. The objectives of this benchmarking project were to: identify metrics to normalize for group size, year of publication, and document type to allow for bibliometric comparison among centers of various sizes; determine the feasibility of repeating the benchmarking project at regular intervals; and create a report format suitable for summary review.
Description: Twelve metrics from Elsevier Scopus and SciVal were selected for the benchmarking project to normalize for group size, year of publication, and document type. Six pediatric cardiovascular centers were identified by the center to use for benchmarking against their center. A five year period, 2013-2017, was determined to be appropriate. Author lists for the centers were provided by the center. Five library staff members were involved with the benchmarking project with a workflow established to replicate the project at two year intervals. Each author was searched in Scopus using the Author search feature to capture publication data. All publication types were included. The publication data from Scopus was imported to SciVal for further analysis. A concise summary report was created to present the findings of the project and included metric definitions and limitations.
Conclusion: Benchmarking of specialized medical centers can be challenging. Limitations include use of a single source for publication and benchmarking data; author name ambiguities; and that publication data alone does not provide a full overview of research performance. Despite these limitations, this type of benchmarking analysis can help centers better understand their impact.
By the end of the session participants will be able to:
1. undertake a similar project at their institution.
2. identify various metrics available for bibliometric comparison of groups to normalize for group size, year of publication and document type.

Optimizing ORCID Membership: A University-Wide Outreach Plan

Jennifer Putnam Davis, Scholarship and Data Librarian, Augusta University, Grovetown, Georgia

Sandra L. Bandy, AHIP, Chair, Content Management, Augusta University, Evans, Georgia

Background: Open Researcher and Contributor Identifier (ORCID) is globally recognized by publishers, research organizations, and funders to distinguish researchers' identity with a 16-digit unique identifier. By recently joining the ORCID US Community consortium member organization, the University has been able to integrate ORCID with two current information research systems, Elsevier Pure and Digital Measures. This allows the University to streamline researchers' scholarship activities. The Library is leading this effort by educating faculty to register for an ORCID iD and adopt its interoperability features. This project is a collaboration between the Library and University stakeholders, such as the Division of Institutional Effectiveness.
Description: The Library's Chair of Content Management and the Scholarship and Data Librarian created an outreach plan to reach faculty researchers about obtaining ORCID iDs and granting permissions for syncing with Pure and Digital Measures. This plan involved a tiered approach, starting with presenting an overview of the ORCID integration project and its benefits to University executive administration, then reaching faculty at a divisional level. The project leaders implemented a pilot program with the Cancer Center. Several drop-in sessions throughout the University's campuses occurred, as well. In general, the Library continuously markets and promotes the ORCID integration through University channels, such as the newsletter, as well as by collaborating with other University entities, such as Human Resources' new faculty onboarding program. Success of this outreach initiative is measured by the number of faculty reached for implementing ORCID with these systems.
Conclusion: ORCID integration will allow Pure to be a more robust tool for connecting researcher's with similar profiles. Digital Measures integration with ORCID will be used for the purpose of syncing research activities for performance evaluation data.

Elevating Researchers' Impact: Turning Posters into Peer-Reviewed Publications Research

Terry Kit Selfe, AHIP, University of Florida, Gainesville, Florida

Objectives: Novice, unpublished researchers might Increase research impact by disseminating study findings in scholarly journals, if they were shown concrete steps to follow, plus research study reporting guidelines to use as a blueprint. Our objective was to determine whether novice researchers benefitted from a librarian-led training on converting locally presented poster content into a manuscript suitable for a peer-reviewed journal.
Methods: Targeting recent CTSI Clinical Research Day poster presenters, we piloted a workshop designed to encourage them to turn their content into a manuscript publishable in a scholarly journal, and thus broaden the dissemination and increase the potential impact of their research. We covered choosing a journal, finding the journal's requirements, locating the reporting standards for the study type (e.g., CONSORT), and structuring the manuscript appropriately per International Committee of Medical Journal Editors recommendations (e.g., Introduction, Methods, Results, and Discussion). At the end of the workshop, each attendee was given a brief course evaluation survey to complete anonymously and place in an opaque envelope. Data were entered into an Excel spreadsheet and response counts were tallied. The study protocol, including the survey instrument, was submitted to the University of Florida Institutional Review Board which determined it qualified for exempt status.

Results: Of the 14 attendees completing surveys, 13 (92.9%) found the course to be helpful (extremely (n=1), very (n=8), and somewhat (n=4) helpful). The numbers were exactly the same regarding satisfaction with the course content (extremely (n=1), very (n=8), and somewhat (n=4) satisfied). Only one attendee (0.07%) reported finding the course not very helpful, and being not very satisfied with the content. Twelve respondents (85.7%) said they felt more (n=10) or much more (n=2) confident in their ability to write a publishable article, with 10 (71.4%) feeling it enhanced their likelihood of getting an article published in a peer-reviewed journal.
Conclusions: This librarian-led training on converting recently presented poster content into a manuscript suitable for a scholarly journal was found to be beneficial to novice researchers. Based on these findings, the CTSI is eager to have this training offered on an annual basis following their Clinical Research Day Poster Session. Poster presenters at this year's event proved to be an excellent target audience. This training is broadly applicable and can easily be adapted to follow a variety of research poster events. Future plans are to offer sessions following the Undergraduate Research Symposium, and the College of Dentistry's research poster event.

Open Science in Biomedical Research in Taiwan: An Exploratory Study

Kathy Kwan, Health Information Specialist, Independent, POTOMAC, Maryland

Chen-jung Huang, Librarian., National Taiwan University Medical Library, Taipei, Taipei, Taiwan (Republic of China)

Objectives: Open science, defined as free access to scholarly articles and its supporting data resulting from publicly funded research, is agreed to be important in Europe and US. However, its state in the biomedical research community in Taiwan is relatively unknown. This exploratory study investigated researchers' attitude towards open science in Taiwan and explored possible ways for its promotion.
Methods: The study was done through a series of guided interviews and an online survey, conducted from October to December 2017. The focus was on researchers' acceptance of open science, the obstacles in practicing open science, and the ways that open science could be promoted in Taiwan. Sixteen interviews were conducted with over 50 stakeholders of open science, including researchers, clinicians, health professionals and librarians, at the National Taiwan University College of Medicine, National Taiwan University Hospital and Academia Sinica.
In order to get a wider perspective and confirm the impression gotten from the interviews, an online survey, administered with Google Form, was conducted after the interviews were concluded. The survey targeted biomedical researchers in organizations served by members of the Taiwan Medical Library Association. A total of 57 valid responses were received.
Results: A majority (>90%) of the targets were supportive of openness in their research output and >75% had published open access articles. However, journal impact factor remained the key factor in deciding where to publish.
No requirement from funders to share data; fear of data scooping; privacy or intellectual property issues; were identified as obstacles in sharing research data.
Two third of the targets felt that the keys to promote open science were:
1. a national policy to mandate the sharing of articles and associated data, and
2. their openly available articles and data could be counted in their performance assessment.
Conclusions: Based on the study result, the following strategies to promote open science in biomedical research in Taiwan were identified:

1. A policy from the Ministry of Science and Technology, the largest founder of science research in Taiwan, that mandates the sharing of research publications and the associated data.
2. The support of evaluation bodies in research institutions, by rewarding the sharing of research data by their researchers; and putting less emphasis on journal impact factor and seeking alternative measures to evaluate their researchers.
Further study and planning will be needed to put these strategies into actions.

Sunday, May 5, 4:30 p.m.–5:55 p.m.
Room: Columbus IJ (East Tower, Ballroom/Gold Level)

Innovation & Research Practice Session 2

Moderator: Sarah C. Clarke, AHIP

Using Framework Analysis for Qualitative Research Data: Pros and Pitfalls

Tara Malone, Assistant Professor/Reference & Instructional Services Librarian, Robert M. Bird Library, University of Oklahoma Health Sciences Center

Shari Clifton, AHIP, Associate Director & Head, Reference, University of Oklahoma HSC, Oklahoma City, Oklahoma

Background: Originally developed as a practical methodology for applied policy research, framework analysis has become popular for qualitative data analysis in health care fields. Librarians at an academic health sciences center sought to explore the feasibility of using the framework analysis methodology to analyze qualitative focus groups data from a five-year public-health sciences library collaboration. The goal was to determine if this methodology provided rigorous and reproducible results from qualitative data, while maintaining enough flexibility for contextual analysis.
Description: Focus groups with participants from the collaboration yielded approximately 13 hours of audio recordings. Investigators then employed a seven-step process of framework analysis to interpret qualitative data: transcription, familiarization, coding, building the analytical framework, applying the analytical framework, data charting, and data interpretation. Benefits of using framework analysis for focus group transcripts included allowing investigators to manage a large amount of data, providing enough structure for reproducibility, ease of synthesis, and allowing a flexible mix of inductive and deductive approaches to the data. Challenges primarily focused on the time-consuming nature of checking transcription, coding, and the charting data phase of analysis.
Conclusion: Large amounts of qualitative data can be overwhelming and difficult to systematically analyze in a reproducible manner. The framework analysis methodology provides structure and reproducibility with enough flexibility to honor the unique nature of qualitative research.

Elevating Health Sciences Librarians’ Research Capacity through an Innovative Research Training Institute

Jodi L. Philbrick, AHIP, Senior Lecturer, University of North Texas, Denton, Texas

Lorie A. Kloda, AHIP, Associate University Librarian, Planning & Community Relations, Concordia University, Montreal, Quebec, Canada

Susan Lessick, AHIP, FMLA, Librarian Emerita/Project Director, RTI, University of California, Irvine, Anaheim, California

Background: The MLA Research Training Institute (RTI) is a three-year program funded by the Institute of Museum and Library Services (IMLS) to equip practicing health sciences librarians with key research competencies related to scholarly research, inquiry, and publishing. The centerpiece of the RTI is a five-day immersive research workshop, where librarians develop the skills necessary to conduct a research study during the ensuing year. RTI fellows participate in an online community of practice, which reinforces learning goals and provides ongoing support. A secondary objective of the RTI is to spur health information research to advance evidence-based practice in health sciences librarianship.
Description: Planning for the RTI began in 2015 and grew out of the work of the MLA Research Imperative Task Force. Intensive planning and implementation of RTI activities occurred during the first year of the grant. Five faculty developed a curriculum to teach the cohort of 20 fellows about the process of conducting research from start to finish. During the workshop at the University of Illinois-Chicago campus, faculty engaged participants with discussion about research methods and analysis, hands-on activities, and peer-to-peer/peer-to-faculty mentoring. Fellows, with faculty mentoring, are participating in a yearlong experience of conducting a research project at their home institutions. Pre- and post-test surveys were conducted to measure the fellows' learning of the research concepts. Data was gathered from participants regarding their research progress and outputs via quarterly reports. Informal feedback and other evidence of fellows' research activities were collected.
Conclusion: Developing the MLA RTI has already had a positive impact on the first cohort practicing health sciences librarians' ability to conduct research. The authors will examine the impact of the research training and support program on participants' research and practice. Fellows' pre- and post-test results that measure changes in research confidence and capability to design and conduct research will be discussed. Research progress and output of cohort members will be shared. Other types of research activities resulting from fellows' participation in the RTI program will be described.

Trend and Transformation of Research Measurement Tools in the Field of Health Literacy: An Exploratory and Descriptive Analysis of the Instruments in Chinese Society Research

Peggy Liu, Student, Dept. of Healthcare Administration, Asia University, Taipei, Taipei, Taiwan (Republic of China)

Ling-Ling Yeh

Objectives: The development of the Health Literacy Measurement Tools in Chinese society has nearly started a decade ago. The tools developed by government or research institutions are China's Health Literacy 66 and Taiwan's MHLS. In order to further explore the trend and development of health literacy in Chinese society, Literature associated with these two main scales has been investigated.
Methods: Literature analysis is adopted to inspect eight databases, including Ovid Medline, CNKI, WANFANG DATA, CQVIP, Airiti Library, Law Data, NDLTD in Taiwan, and PerioPath Index to Taiwan Periodical Literature System, and the Chinese government's statistical yearbooks, totaling 185 journal articles, and 28 master's theses. From these literatures, data of health literacy scale of these two regions were organized and analyzed from the following perspectives: validity and reliability, health literacy

domain measured, specific context, modern approach for tool development, maximum number of items, approximate administration time and sample size in validation study, age validation, and measure availability.
Results: China's "Healthy Literacy 66" contains three major parts: basic health knowledge and concepts, healthy lifestyle and behaviors, and basic skills. The overall level increased from 6.48% in 2008 to 14.18% in 2017. Taiwan's MHLS contains three-stage, five-level healthcare concepts and disease treatment development process, and designs four different question pools: the reading and understanding for patient education information, dialogue simulation for physician-patient interaction, user-guide of medicine bag and the comprehension and signing of medical documents . The survey started in 2012 and has successfully helped to increase the budget for preventive medicine, and to publicize in the international communities.
Conclusions: China's health literacy scale is implemented by government without participation of scholars and experts. Due to the complexities and difficulties of the scale implementation, mobile technology, especially the penetration of mobile phone functioning as the major payment tools in China, are be an alternative of point to overcome the barriers. In addition, more stakeholders, such as scholars and experts, to be involved in the government projects in the future. Moreover aligning the value of mobile technology in promotion and monitoring, the ultimate objective is to improved people's health literacy.

Building a Conflict of Interest Disclosure Tool

Alisa Surkis, Assistant Director, Research Data and Metrics/Vice Chair for Research, NYU Health Sciences Library, New York, New York

Brian Han

Ian Lamb, Solutions Developer, NYU Health Sciences Library, New York, New York

Stuart Spore

Background: News reports about a prominent cancer researcher's failure to disclose conflicts of interest (COI) in journal articles led to increased scrutiny of this issue from leadership at our academic medical center. Leadership reached out to the compliance office for information on whether institutional researchers were making appropriate COI disclosures in articles. The compliance office had access to financial relationship information through the OpenPayments database, but was faced with the task of manually checking articles from all researchers with potential COIs. Compliance office personnel reached out to the library for assistance with this task.
Description: The library maintains a database of faculty publications, so was able to provide a quick response to the request from the compliance office. We extracted PMIDs from our database and, using the NCBI E-utilities programs, queried PubMed for COI statements from articles. Since March 2017, PubMed has been including COI statements in article records, when those statements are supplied by the publisher. After meeting with members of the compliance office, the library undertook a project to expand the search and provide a more user-friendly interface. The E-utilities were used to query the PubMed Central (PMC) Open Access subset and search for the COI statements within the full-text XML of the articles. The results of these searches were integrated into an existing library tool for displaying

faculty publication metrics, with initial access restricted to the library, compliance office, and senior leadership.
Conclusion: The library's queried PubMed for articles published in 2017-2018. Of those 7.3% were returned with a COI statement. COI statements for an additional 10.5% of articles were located within the XML of the PMC Open Access subset. The library was therefore able to provide the compliance office with extracted COI statements from 17.8% of articles queried. In order to improve upon this rate, we next plan to use the PMC FTP service to download full-text for the PMC Author Manuscript subset (~10% of PMC articles), and extract COI statements from these articles.

Grant-Funded Research Activities in Academic Health Sciences Libraries 2012-2017 and Opportunities for Career Preparation
Research

Nandita S. Mani, AHIP, Associate University Librarian & Director, Health Sciences Library, University of North Carolina, Chapel Hill, Chapel Hill, North Carolina

Lauren Tomola

Fei Yu, Health Informatics Librarian, Health Sciences Library at the University of North Carolina at Chapel Hill, Cary, North Carolina

Objectives: To keep pace with the expanding scope and technological complexity facing the research enterprise, Librarians face challenging questions about the skills and knowledge necessary to participate in grant-funded research (GFR). Since limited data about these questions exist, UNC Health Sciences Library (UNC HSL) designed two surveys to investigate librarian engagement in GFR and the skills needed to meet future opportunities.
Methods: The UNC HSL developed two survey questionnaires on the skills and knowledge librarians require for GFR participation (i.e., UNC IRB#18-1642), with a survey population that included all librarians and administrators working at health sciences libraries or medical libraries in North America. In August 2018, one survey was sent to the library directors who are members of the Association of Academic Health Sciences Libraries (AAHSL) and another survey was administered to librarians who are members of the Medical Library Association (MLA). Both surveys were active for four weeks to collect data.
Results: The two complementary surveys received 129 total responses, with 50 from AAHSL and 79 from MLA. Results illustrated that 74% of the responding AAHSL library directors reported that their libraries had participated in GFR projects in the past five years, and 35% of the responding MLA librarians indicated that they had opportunities to participate in GFR. In the GFR projects, two significant external collaborators were School of Medicine and Clinical and Translational Science Award (CTSA) Program Hubs. The skills that librarians identified as critical for future GFR projects included grant searching/writing, data management/analytics, and systematic review expertise.
Conclusions: Our study reveals that majority of health science librarians are interested in participating in GFR activities. However, their opportunities to become involved have been limited due to lack of time, limited opportunities to gain necessary skills, limited staffing, knowledge of how to engage with grant opportunities, and lack of awareness by potential collaborators about the breadth of librarian expertise.

In addition, this study identified core skills that librarians will need to develop during the next five years in order to capitalize on and participate in GFR activities.

Sunday, May 5, 4:30 p.m.–5:55 p.m.
Room: Columbus KL (East Tower, Ballroom/Gold Level)

Lightning Talks 2

Moderator: David Duggar, AHIP

Informatics Consult Services: A Partnership Between Librarians and Informaticists

Karen Heskett, Instruction Librarian, UC San Diego, La Jolla, California

Korey G. Brunetti, Assistant Director, Academic Liaison Program & Health Sciences Librarian, University of California, San Diego, La Jolla, California

Mary Linn Bergstrom, Liaison Librarian, UC San Diego Library, La Jolla, California

Background: As more health care facilities use electronic health records (EHR) to track, store and retrieve patient health data, new opportunities arise for information professionals in the clinical environment. These professionals can work with and support health professionals looking to mine patient data and use evidence-based resources for patient care decisions. This presentation will raise awareness of unique partnerships librarians can forge in this era of big health data and the EHR.
Description: The Informatics Consult Service pilot project aims to improve patient care by leveraging informatics with data specialists' and librarians' collective expertise. Pairing details from millions of patient health records along with librarian-conducted systematized searches of published literature, this service is a protocol for providing evidence based responses to physicians' clinical care questions. Librarians provide rapid paced, systemized searches of published literature. When the literature does not satisfactorily address a question, electronic health record data is queried to identify cohorts that might address the question. This lighting talk will summarize successes, challenges and plans for the future.
Conclusion: This is a lightning talk proposal. We are not expecting an opportunity to measure outcomes.

Survey of Credit-Bearing Library Courses for Health Sciences Students

Emily Brennan, Research and Education Informationist, Medical University of South Carolina (MUSC), Charleston, South Carolina

Emily P. Jones, AHIP, Research and Education Informationist, Medical University of South Carolina, Charleston, South Carolina

Christine Andresen, Research & Education Informationist, Medical University of South Carolina, Charleston, South Carolina

Irene Lubker, AHIP, Research and Education Informationist, Medical University of South Carolina, Charleston, South Carolina

Objectives: This research project aims to present an overview of credit-bearing courses offered by librarians at Association of Academic Health Sciences Libraries (AAHSL) member institutions. This research project is funded by a Medical Library Association (MLA) Research, Development, and Demonstration Project grant.
Methods: The grant team developed and distributed a national survey administered via REDCap to health science libraries that are AAHSL member institutions in the United States (U.S.). This cross-sectional survey aims to investigate prevalence, content covered, credits offered, barriers, successes, and other details about credit-bearing library courses offered by librarians at AAHSL institutions. Additional data will be collected using semi-structured interviews of institutions that offered credit-bearing library courses during the 2017-2018 academic year. Semi-structured interviews will be thematically analyzed using qualitative methods. The team pilot tested the survey with academic librarians not included in the study population to confirm the validity and reliability of the survey.
Results: This survey was completed by 40 of 150 U.S. AAHSL institutions, resulting in a 27% survey completion rate. Preliminary results indicate that during the 2017-2018 academic year, 17 institutions (42.5% of survey respondents) stated that they offered library-sponsored courses to students for academic credit, 8 institutions (20%) stated that they did not offer such courses but planned to in the future, and 15 institutions (37.5%) stated that they did not offer such courses and do not plan to. The greatest barrier in planning and teaching such courses is lack of time. Topics taught in library-sponsored courses include literature searching (26%), evidence-based practice (20%), citation management (18%), followed by a variety of other topics. 79% of courses were taught in-person, followed by 18% asynchronous online, and 3% synchronous online.
Conclusions: The grant team will conduct semi-structured interviews of institutions that indicated that they offered credit-bearing library courses during the 2017-2018 academic year in order to confirm that these courses are indeed credit-bearing library-sponsored and not offered through a specific college's curriculum. More detailed data pertaining to content covered, barriers, successes and other information will be obtained through these interviews. The grant team will use these results to evaluate the utility of credit-bearing library courses.

Make It a Game! Engaging Students in Searching the Literature

Carol Shannon, Informationist, Academic & Clinical Engagement, Taubman Health Sciences Library, Ann Arbor, Michigan

Background: In 2015, I revised the library curriculum for the College of Pharmacy, working with faculty to increase the number of sessions from two to three, scaffolding the instruction, and introducing a flipped classroom model, with active learning and assessments throughout. In one session, students were not learning as much as I had hoped. In part, this was due to the setting - an auditorium - which made it hard for students to work in small groups. I wanted to rethink the session. Was there a way to revise the active learning portion to make it more effective?
Description: Working with colleagues, I decided to make a "serious" game for the active learning portion of the session. In this way, I could guide the students' work, encourage them to work more as a group (they were told that only a limited number of logins was allowed), and give them positive feedback along the way, so even if they got an answer wrong, they would still see the correct one. It was

challenging to translate the group activity into a game: one colleague had to learn to use a new program, some activities that worked well in person didn't work as well in a game, and the game had to be accessible.
Conclusion: I have now completed a 2nd year using this game in class. The data from assessments is still mixed, but the trend is upward. I hope that with a few more years of assessments, I will be able to tell with more certainty the game is. The students have been more animated in class and seem to enjoy the game, with the possibility of reward, as well.

Call Numbers as Data-Driven Collection Management Tools

M Wynn Tranfield, Physical and Basic Sciences Librarian, Louise M. Darling Biomedical Library, Los Angeles, California

Background: The physical collections of large academic institutions are constantly in flux. Increasing demands for study and active learning spaces are shrinking available shelf space, and collections decisions are increasingly data-driven. For new librarians that lack experience working with large print collections, especially in the health sciences, making informed collection management decisions about legacy collections can be intimidating.
Description: A recent case study examined a legacy collection for continued relevance to future research and instruction needs. Circulation statistics were incomplete, so in order to determine action areas of the collection, librarians consulted cataloguing records. Voyager records are returned to librarians in Excel spreadsheet format, but require cleaning in Open Refine to isolate LOC Classification Subclasses. The clean data can then be uploaded to Tableau visualization software, which projects record attributes into a single view. Librarians and stakeholders can see a snapshot of the shelf on one screen.
Conclusion: The ability to view and compare collection component ratios highlighted the strengths and weaknesses of the collection. The framework permits adding existing circulation data if available, consortial data, and shelf area. This added context provided librarians with an improved rationale for large-scale decision-making.

Facilitating Open Access through Collaboration with Clinical Departments

Jessica Petrey, Assistant Professor/Clinical Librarian, Kornhauser Health Sciences Library, Louisville, Kentucky

Background: In order to reduce economic barriers to health sciences information, a group of physicians consulted their clinical librarian about the university libraries' capabilities in supporting their goal of creating and publishing two open access journals.The faculty's desire was to have these journals not only be free to read, but also free of publication costs to potential authors. A working group including division faculty and staff members, clinical librarian, and library faculty and staff from archives and special collections was created in July of 2016 to explore the potential project.
Description: The work group spent 6 months on negotiations and project planning, including deciding on design aspects of the site, calling for editors, authors, and reviewers, and drafting a memorandum of understanding detailing labor responsibilities between the University Libraries' and the clinical

department. Instead of subscription or submission fees, the project was funded by a combination of grants, departmental support and existing library services. The journals are housed in the University Libraries' institutional repository platform, but the editorial board and technical support are staffed by clinical faculty and employees.
Conclusion: Since its launch in January 2017, the Journal of Respiratory Infections has published 52 articles which have been downloaded a total of 7890 times in 127 different countries. The 14 articles published in the Journal of Refugee and Global Health, which launched in August 2017, have been downloaded 1342 times in 86 countries. By the end of the session, participants will be able to thoughtfully examine opportunities that may exist within their own institution and service model to facilitate equitable dissemination of information through the support of open access journals.

Practicing What We Preach: Making Our Own Research Data Open Access

Melissa Ratajeski, AHIP, Coordinator of Data Management Services, University of Pittsburgh, Pittsburgh, Pennsylvania

Carrie L. Iwema, AHIP, Coordinator of Basic Science Services, University of Pittsburgh, Pittsburgh, Pennsylvania

Background: Data services librarians at a large academic health sciences institution deposited three datasets from their own research into figshare. The goals of this strategic decision were to: understand and document the workflow in order to assist researchers with their own deposits; facilitate requests to share the data; make unpublished results discoverable; track usage of the data; and model best practices to researchers and librarian colleagues. Given the new data sharing policy for the Journal of the Medical Library Association that will go into effect October 2019, we believe this last goal is of particular importance.
Description: The deposited datasets are from three separate projects relating to assessment of internal library services and the services offered by peer libraries. Two of the projects were approved and granted exempt status from the University's Institutional Review Board (IRB). In order to deposit the datasets, make them reusable, and increase discoverability, a number of steps were taken including: detailing why and how the data was collected, uploading supplemental materials such as survey instruments and codebooks, ensuring de-identification of the data, assigning keywords and Medical Subject Headings to the dataset record, including the dataset citation on our curriculum vitae, and linking to the dataset from our institution's data catalog.
Conclusion: Our first dataset was deposited in 2017 and since that time we have met all of our defined goals including knowledgeably answering several researcher questions about deposits into figshare and illustrating our own impact as researchers on annual faculty appraisals through tracking of growing dataset metrics (views, downloads, and citations). In this short presentation we will share our experiences and provide practical guidance for librarians who may have to deposit their own datasets in the near future.

Developing an Interactive Tool to Assist in Complying with the National Institutes of Health (NIH) Public Access Policy

Merle Rosenzweig, Informationist, University of Michigan, Ann Arbor, Michigan

Tyler Nix, Informationist, University of Michigan, Ann Arbor, Michigan

Chase Masters, Enabling Technologies Informationist, University of Michigan, Ann Arbor, Michigan

Background: On April 7, 2008, the National Institutes of Health instituted the NIH Public Access Policy. In order to apply for and to continue receiving NIH funding, a researcher needs to comply with the Policy. There are many resources that address compliance with the National Institutes of Health Public Access Policy but most are text heavy. Our goal was to create a tool that would simplify this complex process. We broke down the myriad tasks involved and represented them in an interactive flow chart. By accomplishing each individual task, the NIH Grantee can achieve compliance with the Policy.
Description: The interactive tool was created in two iterations. The first focused on compliance based on the policy's four available manuscript submission methods. Microsoft Visio was used to develop the simplified PDF process map in which interactive links provided the user with additional information by linking to external documentation. Due to its success, a second, more detailed process map was later developed by the team using the Lucidchart software. It covers the entire submission process and how to track compliance status following submission. Each box on the process map links to a page within the PDF with additional compliance information, and linear navigation is available at the bottom of each page to walk users step-by-step through the process.
Conclusion: Since the National Institutes of Health Health's Public Access Policy was launched, librarians and informationists have provided support and instruction to those needing to comply with it. Complying with the Policy can be a daunting task that can be time consuming and frustrating. The interactive tool we have developed can help in navigating the complex process required in complying with the Policy. Our talk will outline the software and workflows that the team used to create the interactive process map with the goal of raising awareness for librarians interested in developing similar learning objects to meet their specific training needs.

How Big Is Too Big: A Case Study Using Keyword Text Analysis and Unsupervised Machine Learning to Manage Large Search Results

Jennifer S. Walker, Cancer Information Librarian / Liaison to the School of Dentistry, University of North Carolina at Chapel Hill, Chapel Hill, North Carolina

Barbara Rochen Renner, Library Services Evaluation Specialist/Allied Health Sciences Liaison and Adjunct Professor, Allied Health Sciences, University of North Carolina at Chapel Hill Health Sciences Library and School of Medicine, Department of Allied Health Sciences, Chapel Hill, North Carolina

Michelle Cawley, Head of Clinical, Academic, and Research Engagement, UNC Chapel Hill Health Sciences Library, Chapel Hill, North Carolina

Objectives: To employ the use of a data-driven technique to refine a complicated search strategy by analyzing contributions of individual keywords. To prioritize results from a comprehensive, complex search on a broad topic using machine learning.

Methods: We developed a comprehensive search strategy on a diffuse public health subject that resulted in more than 50,000 results from PubMed. To refine the search strategy, we conducted keyword analysis that indicated the relative contribution by individual keywords and informed our decision-making around terms to exclude from the final search strategy. Using this method we revised the search strategy by eliminating several keywords. After executing the final search, we used unsupervised machine learning or clustering to prioritize a subset of studies for manual review. Specifically, we used a clustering algorithm that groups like references based on text similarities in titles and abstracts. For each cluster, the software produces a set of keywords or a topic "signature". We used these topic signatures to select clusters to prioritize for manual review.
Results: Our initial search in PubMed yielded approximately 50,000 results. Keyword analysis informed our decisions to eliminate several terms that were causing excessive "noise". The revised search was reduced to approximately 15,000 records. These records were clustered using unsupervised machine learning and we prioritized approximately 1,400 studies for manual review.
Conclusions: Keyword analysis informs searchers of potentially irrelevant terminology in their search strategies and is useful in shining a light on a set of results at any stage of developing a search strategy. Unsupervised machine learning or clustering assists research groups in prioritizing a subset of literature from a large set of results and is particularly useful for broad, comprehensive literature reviews. Our initial results from manual screening indicate a higher degree of precision than would be typically expected from such a large set of search results.

Yes, It Really Is Necessary to Use Controlled Vocabulary in Systematic Searches

Kate Nyhan, Research and education librarian, Yale University, New Haven, Connecticut

Objectives: Do researchers ever ask you, "Is it truly necessary to use controlled vocabulary in a systematic search?" How about, "If a keyword search is sufficient in Scopus, why isn't a keyword search sufficient in MEDLINE?" Expert searchers know that searching with both keywords and subject headings, when possible, is a best practice. So let's find some real life examples!
Methods: Using five high-quality, fully reported systematic reviews published in prominent journals (JAMA, Lancet, BMJ) as a source of search strategies and inclusion decisions, I will investigate how many of the included articles are not retrieved by a keyword-only version of the documented search.
Results: For each of the five included studies, I will report these data points:
- number of included papers
- number of included papers found by the documented search strategy in MEDLINE or PubMed
- number of included papers found by a keyword-only version of the documented search strategy in MEDLINE or PubMed
- number of included papers that could have been missed, had the authors not used controlled vocabulary in the MEDLINE or PubMed search
Conclusions: I anticipate that this small-scale study will demonstrate what expert searchers already know: that a search strategy with controlled vocabulary and keywords is better than a search strategy with keywords alone. Because this small-scale study draws on examples of real publications from prominent journals (instead of hypothetical examples created by librarians), I anticipate that its anecdotal findings will be useful in systematic review planning consultations.

Quality Assurance in an Academic Ask-A-Librarian Service

Nita K. Mailander, AHIP, Director of Library Services, Grand Canyon University, Phoenix, Arizona

Background: In 2010, the University Library, which supports nursing and health sciences programs from pre-licensure to the doctoral level, established a new model of centralized Ask-A-Librarian services, to answer all questions regardless of subject and origin, providing virtual and face-to-face, on-demand individualized research assistance to students, staff, and faculty. In 2012, phone queue software was implemented to provide efficient call flow and maximize customer service. Six years later in 2018, a phone quality assurance (QA) initiative was launched to include the evaluation of library inbound and outbound phone calls.
Description: The phone QA initiative measures eight areas of staff competency including, call opening, knowledge and understanding, communication, advising and problem solving, ownership, branding, and closing. Two inbound calls and one outbound call are evaluated each month for those staff who answer calls for the Ask-A-Librarian service. The evaluation results are accessible via a staff personal dashboard and are reviewed by department supervisors. Staff members reaching and maintaining one hundred percent compliance are recognized quarterly at departmental staff meetings and become part of the University's QA 100 Club. Since implementing the phone QA initiative in September 2018 over 8,288 inbound and outbound calls were handled by library staff and 305 evaluations were completed by April 15th, 2019. The library evaluation scores resulted in an average departmental score of ninety-seven percent.
Conclusion: The implementation of assessment to the Library's virtual phone service strengthens the Library's continuous improvement processes, enhancing the Library's quality and overall effectiveness in providing academic research and learning support to students, faculty, and staff. Future QA initiatives will include the implementation of a call library with model examples of excellent customer service interactions and an expansion of the QA model to the library's chat service.

How Are Researchers Documenting Their Day-to-Day Activities?

Merle Rosenzweig, Informationist, University of Michigan, Ann Arbor, Michigan

Marci Brandenburg, Bioinformationist, University of Michigan Taubman Health Sciences Library, Ann Arbor, Michigan

Scott Martin, Biological Sciences Librarian, University of Michigan, Ann Arbor, Michigan

Leena Lalwani, Engineering Librarian, University of Michigan, Ann Arbor, Michigan

Mark MacEachern, Informationist, University of Michigan, Ann Arbor, Michigan

Yulia Sevryugina, Chemistry Librarian, University of Michigan

Background: We undertook a pilot project to evaluate the feasibility of launching a full-scale research project to investigate best practices of researchers at our institution for keeping lab notebooks. Our objectives are to learn about practices already present on campus through a series of faculty interviews,

and to analyze those practices based on their similarities or differences, their compliance with modern technology, and ease of use and access, as well as to suggest other practices based on our review of tools reported in the literature.
Description: We focused on a narrowly defined group of researches to obtain a more useful set of conclusions upon which to launch a full-scale project. After obtaining IRB approval (HUM00147763), we emailed a biology department at our institution, our sample population, to explaining the project and asking for volunteers. We interviewed six-research faculty from that department about their recordkeeping practices: including print vs. electronic notebook use, data and protocol sharing within the lab, and standard policies. Interviews were recorded and transcribed, and the transcripts were coded and analyzed.
Conclusion: The results provide a snapshot of current disciplinary practice, which will be useful as a benchmark for longitudinal studies of a wider community as campus-supported electronic tools for research documentation are expected to become more widely available in the coming years. They also suggest points of need where the library might productively offer services within the current paradigm to improve access to and preservation of day-to-day research documentation.

Advocate, Innovate, Elevate: Developing Strategies to Market Your Library Services and Align with Organizational Strategic Goals

Carol F. Colasacco, AHIP, Medical Librarian Specialist, College of American Pathologists, Pleasant Prairie, Wisconsin

Brooke Billman, AHIP, Medical Librarian, College of American Pathologists, Northfield, Illinois

Background: Librarians in non-traditional or small libraries face numerous challenges in today's environment -- Many have limited visibility and face financial constraints that may seriously impact their operational capacity. Recognizing a library's (or librarian's) value to an organization may not be intuitive for those responsible for making difficult financial decisions about limited resources. The objective of this presentation is to encourage librarians in niche environments or small libraries to explore opportunities to elevate the awareness of the benefits they provide to the organization and advocate for themselves by developing marketing strategies and innovative programs that align with organizational strategic goals.
Description: The College of American Pathologists (CAP) has well defined strategic goals, including several particularly pertinent for residents, fellows, and new-in-practice pathologists. After reviewing the strategic goals and identifying a potential area of need, we developed a proposal focused on pathology residents and fellows for the CAP annual meeting. We worked with CAP staff and the Residents Forum Executive Committee to determine topics of interest and designed two small group presentations and individual consultations scheduled on two separate days during the CAP annual meeting. Information about the sessions was included in CAP meeting publications, and resident/fellow members were emailed about the opportunity to attend these librarian-led sessions. Attendees registered in advance and submitted materials via email if requesting librarian review. Walk-in participants were also encouraged at the meeting. Follow-up materials and a survey were emailed to all participants after the meeting.
Conclusion: Feedback was positive, and survey results were shared with CAP staff and oversight committees. A debrief meeting was held to help inform future meeting planning efforts. Future planning

efforts are ongoing in collaboration with CAP staff and committees representing pathology residents and fellows.

Sunday, May 5, 4:30 p.m.–5:55 p.m.
Room: Columbus EF (East Tower, Ballroom/Gold Level)

Professionalism & Leadership Immersion Session 3

Moderator: Jennifer A. Lyon, AHIP

Searching Clinically: How to Search, Find, and Assess Information at the Point of Care, Part 2 Research

Angela Spencer, AHIP, Manager, Medical Library, St. Lukes Hospital, Chesterfield, Missouri

Ellen Aaronson, AHIP, Librarian, Mayo Clinic, Rochester, Minnesota

Kerry Dhakal, Assistant Professor/Research and Education Librarian, The Ohio State University, Columbus, Ohio

Rebecca C. McCall, AHIP, Clinical Librarian, University of North Carolina-Chapel Hill, Chapel Hill, North Carolina

Jennifer A. Lyon, AHIP, Librarian, Children's Mercy Kansas City, Kansas City, Missouri

Jennifer A. Lyon, AHIP, Librarian, Children's Mercy Kansas City, Kansas City, MissouriWorking with health care teams at the point of care requires clinical awareness and the ability to quickly locate and assess case-relevant information. Through presentations, demonstrations, group activities, and discussion, you will learn about and practice how to quickly meet clinician information needs. This exceptional, double session is for librarians who work in a clinical setting, participate in rounds, or provide instruction at point of care.
Part 2 focuses on searching techniques that rapidly locate high-quality, case-relevant information, particularly for challenging clinical questions, using PubMed and other specialized point-of-care or evidence-based medicine resources, quickly judging quality and relevance of located information, and identifying the best methods of communication and information delivery.

Sunday, May 5, 4:30 p.m.–5:55 p.m.
Room: Grand Ballroom B (East Tower, Ballroom/Gold Level)

Professionalism & Leadership Immersion Session 4

Moderator: Catherine Soehner

Tips for New Leaders: Advice from the Trenches

Catherine Soehner, Interim Executive Director, University of Utah, Salt Lake City, Utah

Catherine Soehner, Interim Executive Director, University of Utah, Salt Lake City, Utah

Melissa L. Rethlefsen, AHIP, Associate Dean and Fackler Director, University of Florida, Gainesville, Florida

Nicole Capdarest-Arest, AHIP, Head, Blaisdell Medical Library, University of California, Davis, Sacramento, California

James Dale Prince, AHIP, Director of Libraries, LSU Health Sciences Center, New Orleans, Louisiana

Stephanie C. Kerns, Director of Biomedical Libraries, Dartmouth College, Geisel School of Medicine, Hanover, New Hampshire

Jeff D. Williams, AHIP, Chair/Director, NYU Health Sciences Library, NYU Health Sciences Library/NYU Langone Health, New York, New York

Catherine Soehner, Interim Executive Director, University of Utah, Salt Lake City, Utah

Join in a discussion with 6 new leaders of health sciences libraries to learn about their first year of leadership. An initial introduction by the panelists will give you their top advice for starting out a leadership position. Participation in the Q&A will assist you with your new leadership role or determine if leadership is for you. The panelists will also discuss how library staff can help prepare for a change in leadership most effectively.

Monday, May 6, 2:00 p.m.–3:25 p.m.
Room: Columbus AB (East Tower, Ballroom/Gold Level)

Clinical Support Session 2

Moderator: Merle Rosenzweig

Elevating Evidence-Based Practice (EBP): A Multi-Site Workshop Pilot for Nurses

Lisa A. Marks, AHIP, Director of Libraries, Mayo Clinic Libraries, Arizona, Scottsdale, Arizona

Diana Almader-Douglas, AHIP, Librarian, Mayo Clinic, Phoenix, Arizona

Tara Brigham, Assistant Professor of Medical Education & Medical Librarian, Mayo Clinic, Jacksonville, Florida

Heather Jett, Librarian, Mayo Clinic Libraries, La Crosse, Wisconsin

Background: Objectives include:
-Expanding the reach of an established EBP (Evidence-Based Practice) workshop for nurses from one site of our organization to our other sites where there is no workshop in place.
-Evaluating this method (video-conferencing with local library support in attendance) for future multi-site library instruction.
-Increasing the visibility of the librarian and library services across the organization.
Description: Leveraging an established EBP (Evidence-Based Practice) workshop, the Manager of Nursing Research asked local library staff to discuss the research process and to demonstrate for the attendees how to use literature databases to find material to support their research projects and EBP practices on their units. The EBP workshop was video-conferenced from the originating location to two remote locations within the organization. Each remote site had a librarian in attendance to support the librarian at the originating site. This allowed the librarians at each site to guide and assist patrons and establish a face-to-face connection with attendees. Two post-session evaluation surveys were conducted, one immediately after the event and one two months later.
Conclusion: Based on the course and evaluative surveys, one immediately after the event (43% response rate) and one two months later (55% response rate), this multi-site course was an overall success. Participants suggested that this course should continue to be offered, and emphasized that the quality of the instruction was excellent. Several participants recognized the impact of the librarian and library services in this course. One respondent indicated a plan to “use library resources more.” Other comments included “I am able to refine my literature and library search skills,” and “working with the librarian was very helpful.”

Revising Journal Club to Upgrade Pediatric Residents' Understanding of Evidence-Based Practice

Stephanie Hendren, AHIP, Clinical Information Librarian, Robert B. Greenblatt, M.D. Library, Augusta, Georgia

Peter C. Shipman, Dental Medicine and Cancer Librarian, Augusta University, Augusta, Georgia

Gail Kouame, Assistant Director for Research & Education Services, Augusta University, Augusta, Georgia

Darra R. Balance, Director, Retention Programming & Technology, Georgia Statewide AHEC Network, Augusta, Georgia

Rebecca Yang

Background: The pediatric department at a large academic medical center decided to revise their existing journal club model due to informal feedback from the residents that the current model was not meeting their needs. The first, second, and third year residents each had a specific role in presenting an article in a monthly journal club. The pediatric department requested that the library collaborate on a new approach to the assignment for the third year residents to improve evidence based practice and include more involvement with the librarians.
Description: The first cohort of 13 residents began in June 2017. The librarians rounded with each third year resident twice, on average. Librarians worked with the residents in developing answerable research questions, searching the databases, and providing methods to analyze results. At the end of each rotation, the resident presented the patient and findings to the other residents and attending physicians at a designated morning report. Residents also discussed how the literature did or did not apply to their particular patient scenario, and whether the standard hospital procedure was in line with the published evidence, followed by a group discussion. Afterwards, a librarian evaluated each resident on certain EBM competencies. A second cohort began in July 2018 and offered an opportunity to update the evaluation form to cover 10 specific competencies.
Conclusion: Third year residents were able to engage with librarians beyond the traditional journal club. Residents gained hands-on experience of searching the literature for a specific patient problem and had a platform to share their knowledge with their peers. Librarians utilized the one-on-one interactions to provide tailored literature search instruction based upon the resident's research topic and results found.

Factors Affecting Clinical Referrals to the Medical Library Research

Liz Kellermeyer, Biomedical Research Librarian, National Jewish Health, Denver, Colorado

Matthew Strand

Objectives: The objective of this study is to understand why and when clinical care teams refer patients to the medical library. It provides information about how clinical care teams currently utilize and recommend medical libraries as a patient resource.
Methods: A census of clinical care teams at a research hospital was taken from October to December 2018. The census population was 482; 95 responses were collected. We administered an anonymous online survey using REDCap, featuring multi-choice questions and Likert-type scales to measure awareness of library services available to patients, facilitators and barriers to referral, and likelihood of future referral. Demographic variables included gender, age, professional role, and years employed at the hospital. Spearman correlations were used to determine the strength of relationships between familiarity with the services and how often respondents referred those services (rs). Referral rate distributions were compared between job type groups using the Kruskal-Wallis test.
Results: Overall, self-reported referral rates were low. There was a marginally significant relationship between referral rate and job type, with providers having lower referral rates (p=0.01). There was a positive correlation between familiarity with the services and service referral frequency (rs=0.78 for combined data) and between current referral rates and likelihood of future referral (rs=0.43 for combined data). Among respondents who had never referred patients, top reasons were “Not aware library offered has service” and “Don’t know how to make a referral to library;” least selected reasons were “Clinic wants to control information to patient” and “Concerned about quality of information provide by library.” Top referral reasons were “Complements or enhances the patient’s access to health information” and “Adds value to the patient’s visit.”
Conclusions: The results suggest there is evidence that lack of knowledge, rather than lack of interest and support, results in lower clinic referrals to the library. The correlation between knowledge of services and likelihood of referral demonstrates that when providers are aware of the library, they are referring patients. Similarly, those currently referring are likely to make future referrals, suggesting that the library services are considered valuable. The qualitative responses show agreement, linking the lack of referrals to marketing and procedural insufficiencies (rather than distrust or dislike of the services), which indicates potential for increasing referrals by addressing these deficits. A streamlined patient referral system from clinic to library could be beneficial.

Connecting with Rural Health Care Providers: What Information Do They Need and How Do They Search for It? Results of a Mixed Methods, Multiphase Assessment Project Research

Marcia Francis, AHIP, Southwest Clinical Campus Librarian, University of North Dakota, Bismarck, North Dakota

Michael Skinner, Northwest Clinical Campus Librarian, University of North Dakota School of Medicine and Health Sciences, Library Resources, Minot, North Dakota

Dawn E. Hackman, AHIP, Medical School Liaison, University of Minnesota Bio-Medical Library, Minneapolis, Minnesota

Erika M. Johnson, Clinical Campus Librarian, University of North Dakota, Fargo, North Dakota

Kelly Thormodson, Director, University of North Dakota, Grand Forks, North Dakota

Objectives: Outreach librarians at a small academic health sciences library conducted a mixed methods, multiphase information needs assessment of unaffiliated healthcare providers within a predominantly rural state. The assessment's purpose was to determine the information-seeking behaviors, unmet information needs, and training preferences of the target population to inform future outreach planning.
Methods: Outreach librarians conducted a preliminary literature review that found recent, relevant studies describing the health information needs of unaffiliated rural healthcare providers were lacking. Librarians then designed a descriptive survey to assess information-seeking behaviors and unmet information needs of the target population. State healthcare organizations were recruited to encourage their members to complete the survey. In Phase II, librarians interviewed Phase I volunteers and other health professionals individually to collect additional and more in-depth data, including training preferences. Unmet information needs identified in Phases I and II were mapped to freely accessible online information tools in anticipation of future trainings. Librarians used qualitative data analysis software to glean additional insight into unmet information needs of the target population during Phase III of this project. All research activities were approved by the university's Institutional Review Board.
Results: A total of 282 health professionals working in a variety of roles and settings responded to the Phase I survey distributed in 2017. Respondents reported seeking patient care/management information most often with various resources consulted, free online resources and journals being considered most useful. In Phase II, four librarians interviewed a total of 21 individuals to further explore information-seeking behavior, perceived barriers, and utilization of information resources. Phase II and III qualitative data analysis indicated interviewees primarily rely upon online information resources and colleagues; the most frequently discussed barriers to information-seeking were time and money.
Conclusions: Many unaffiliated health professionals have unmet information needs and are unaware freely available, quality information resources are available to meet some of those needs. These professionals also want access to subscription-based information products and are unfamiliar with product licensing and budget restrictions libraries face. Selected health professionals recognize the value of library resources and librarians' expertise in efficiently locating relevant information to meet their needs. With the wealth of online resources available, some believe currently available information is sufficient to meet their information needs.

Monday, May 6, 2:00 p.m.–3:25 p.m.
Room: Columbus CD (East Tower, Ballroom/Gold Level)

Education Session 3

Moderator: Ayaba Logan

More Instruction Time for Evidence-Based Dentistry: How I Learned to Love Dental Curriculum Change

Peter C. Shipman, Dental Medicine and Cancer Librarian, Augusta University, Augusta, Georgia

Background: Restructuring of the dental school curriculum doubled instruction time for the librarian to teach Evidence-Based Dentistry (EBD) principles and PubMed searching to first-year predoctoral students. The library instruction in the old curriculum occurred in the Research Design and Critical Thinking course and consisted of a single session of 90 minutes with 25 minutes of pre-recorded flipped instruction. The new curriculum created an Evidence Based Dentistry course and the librarian-led instruction expanded to two sessions of 120 minutes each. Neither course allowed for testing and grading is pass/fail.
Description: The second session and more contact time allowed more choices about the type and sequencing of active learning activities. The formative assessment Jeopardy-style game, remained unchanged. Successful activities from the single session (PubMed searching, formatting of clinical questions, student teach-back of PubMed search strategies, and librarian feedback of teaching strategy) were retained and expanded to include more in-class practice and reinforcement of skills. The recorded flipped instruction was discontinued and a critique of a systematic review was added. The expanded time also provided the opportunity to emphasize the importance of EBD to lifelong learning and the value of databases other than PubMed.
Conclusion: The librarian was able to build upon the success of its original librarian led evidence based instruction in the dental college when the college restructured its curriculum. The students received more reinforcement of evidence based practice skills,had more time to complete in-class exercises, and allowed more review of content. All desired active learning activities could be included with less risk of rushing through content. The students are primed to a mindset of lifelong learning. Testing in a pass/fail course was not allowed, so effectiveness is determined by informal observation by the librarian and from the course evaluations of the students.

Integrating Evidence-Based Medicine Skills into a Medical School Curriculum: A Longitudinal Approach

Amy Blevins, Associate Director for Public Services, Indiana University School of Medicine, Indianapolis, Indiana

Laura Menard, Assistant Director for Medical Education and Access Services, Indiana University, Indianapolis, Indiana

Kenneth Lazarus, Senior Academic Content Specialist, Indiana University School of Medicine, Indianapolis, Indiana

Background: This project took place at a large medical school in the Midwest. The school is served by a dedicated library on the main campus with nine liaison librarians available to teach. In 2016, efforts began to integrate EBM throughout the all four years of the undergraduate medical school curriculum, a shift away from the previous model, which consisted of a two month EBM course in the second year of the curriculum and lectures during clerkships. The purpose of this shift was to better prepare the students for their clerkships and residencies, as well as increase EBM scores on standardized testing.
Description: Development: An associate director at the medical library, along with a committee of medical school faculty, oversaw the design and implementation of the new EBM "thread."
This librarian also took on the task of mapping and assisting with planning EBM instruction throughout the undergraduate medical curriculum. Implementation: Many EBM sessions in the new "thread" are designed at the library by a curriculum committee, which then trains liaisons to deliver content. These sessions are a mix of didactic lecture, small group activity, and formative assessment activities. Sessions are delivered in-person, via video conferencing, and using a flipped classroom model depending on time constraints and curricular needs.
Assessment: Every session has a formative assessment, review of "muddiest points", and a summative assessment when the session concludes. Additionally, student EBM scores on course exams and standardized tests are evaluated to determine impact.
Conclusion: The EBM thread has increased students' exposure to EBM throughout their undergraduate medical education. Additionally, librarians have become trusted partners for delivering EBM instruction. Where students in the previous curriculum had isolated EBM lectures during the second and third year, current students receive longitudinal curriculum-integrated sessions covering EBM topics with progressively more clinical applicability as they prepare to enter their clerkships with reinforcing assignments for each required clerkship. As the curriculum team receives the results of standardized testing, we intend to compare the EBM scores of the students who received the new curriculum to those of the legacy curriculum students.

Planes, Trains, and Automobiles? Engaging Medical Students with Interactive, Skills-Based, Multimodal Evidence-Based Practice Instruction

Amy Christine Studer, AHIP, Health & Life Sciences Librarian, University of California, Davis, Sacramento, CA, California

Nicole Capdarest-Arest, AHIP, Head, Blaisdell Medical Library, University of California, Davis, Sacramento, California

Background: The UC Davis School of Medicine was proposing curriculum change that would have resulted in a reduction in curricular time for course content delivered by UC Davis Library (the "Library"). Since the Library continually monitors the curriculum, we seized this opportunity to connect with

education leaders and re-envision Library content on evidence-based practice ("EBP") at an even more developmentally appropriate place, earlier in the curriculum. Resultantly, we collaborated on a teaching session focusing on specialized information resources and the "Ask" and "Acquire" components of the EBP framework early in this new longitudinal thread on population health and evidence-based practice. .
Description: The Library wanted to develop a highly interactive, hands-on session using novel teaching techniques and tools. Two librarians in consultation with the course instructor redesigned the "introduction to library resources" session to include: flipped classroom, individual and team activities, structured and self-directed learning activities, real-time polling, and assignment feedback. Using all these modalities, the instruction was delivered in two sessions (½ class per session) in a state-of-the-art classroom designed for interactive learning (propeller tables with monitors at each table). Topics covered included: question types (background vs. foreground), PICO, publication types, evidence pyramid, resource overviews (e.g., PubMed, AccessMedicine, DynaMed Plus, UpToDate, advanced Google searching), and health information literacy. Assignments were submitted through the university Qualtrics survey tool. Using Qualtrics offered the ability to deliver a developmentally structured assignment, give feedback in real-time, and easily collect and analyze student assignments.
Conclusion: Formal and informal feedback was overwhelmingly positive for the sessions, with we "would like more time for the individual assignment" as the only suggested improvement. This may be remedied by offering students more context in advance of this component. Students demonstrated competency in understanding the EBP process and developing their own PICO questions. Assignments showed room for improvement in distinguishing publication types and identifying and incorporating synonyms into searches. This session was a useful and engaging starting point to introduce learners to EBP and evidence resources. Opportunities to build on these skills can be reinforced and developed throughout the curriculum.

Designing a Brand New Evidence-Based Research Course to First Semester Physical Therapy Graduate Students

Kyle Downey, Health Sciences Librarian, Seton Hall University, Nutley, New Jersey

Background: In the summer of 2018, Seton Hall's brand-new-interprofessional Health Sciences Library worked in collaboration with a faculty member from the physical therapy program to implement a two-day evidence-based research course for first semester graduate students in the second week of their curriculum. Faculty from the Physical Therapy program have noticed a lack of proper evidence-based research skills in their second- and third-year students. The faculty consulted with the library and concluded that introducing a two-day evidence-based research course early in the curriculum would be beneficial for the students in developing the necessary skills to conduct evidence-based practice research.
Description: The first session focused on major databases including PubMed, CINAHL, PEDro and Cochrane Database of Systematic Reviews. The librarian instructed the students on how to develop a researchable question with the PICO framework, how to search through an academic database, and how to use controlled vocabulary and Boolean operators to conduct advanced searches. During instruction, the students were asked to participate in interactive sessions that focused on basic and advanced searches. A PICO search strategy rubric was developed to help guide and evaluate their skills. The last two-hour session was a review of what was previously discussed as well as one last interactive session in which the students took the remainder of the class time to conduct a literature search. The librarian and faculty member assisted the students with any questions that came up during the session.

Conclusion: Student understanding of conducting evidence-based practice research increased after the two-day session. Anecdotal evidence from the professor reported that the students developed a better understanding of conducting a literature search based on assignments given after the sessions. The librarian also expanded his presence on the new interdisciplinary campus and saw an increase in consultation visits. Future courses have been planned to involve a librarian and for the librarian to assist in developing assignments to assess students understanding of evidence-based practice research.

Learning and Teaching Evidence-Based Chaplaincy

Kerry Dhakal, Assistant Professor/Research and Education Librarian, The Ohio State University, Columbus, Ohio

Background: Our library has been serving chaplains for several years however in 2017, I became a faculty mentor to the new chaplain residents at our hospital to teach evidence-based practice and to introduce clinical research methodology as part of a funded project pilot for the chaplain residency.
Description: Prior to becoming a librarian, I was a social sciences researcher and program evaluator conducting research and evaluating community health programs locally, nationally and internationally. For these reasons, and my role in teaching evidence-based practice in other capacities in the hospital, I was invited to be a faculty mentor for the chaplain residency program.

I developed tailored curriculum in collaboration with the chaplain residency program manager for the 2017-2018 chaplain residents and followed them through their EBP and research activities throughout the year. I also graded their final capstone project, development of annotated bibliographies focused on resident PICO questions and topics of interest in clinical chaplaincy.

I will describe the curriculum that we developed, insights and feedback from the chaplain residents of the implementation of the curriculum and instruction and recommendations for librarians considering offering liaison services to chaplaincy residents.
Conclusion: 1. Post-residency evaluation survey data about the EBP and clinical research curriculum and library instruction.
2. Self-evaluation of librarian knowledge and skills needed to serve chaplain residents.
3. Best practices for serving chaplain residents in a large, hospital setting.

Monday, May 6, 2:00 p.m.–3:25 p.m.
Room: Grand Ballroom B (East Tower, Ballroom/Gold Level)

Global Health & Health Equity Immersion Session 2

Moderator: Aimee Gogan, AHIP

Elevating Health Equity: Wikipedia Edit-a-thon

Elaina Vitale, Academic and Data Services Coordinator, National Network of Libraries of Medicine, Middle Atlantic Region, Pittsburgh, PA, Pennsylvania

Aimee Gogan, AHIP, Technical Information Specialist, National Library of Medicine, Catonsville, Maryland

Alicia Lillich, Kansas Outreach and Technology Coordinator, National Network of Libraries of Medicine, MidContinental Region, Kansas City, Kansas

Elaina Vitale, Academic and Data Services Coordinator, National Network of Libraries of Medicine, Middle Atlantic Region, Pittsburgh, PA, Pennsylvania

Aimee Gogan, AHIP, Technical Information Specialist, National Library of Medicine, Catonsville, MarylandThe average American visits the doctor three times per year, but spends 52 hours searching for health information on the internet. With 7 billion views a year of health topics pages, Wikipedia may be the most popular online health information resource. This session will describe the importance of Wikipedia as a resource for health information and how librarians can utilize their research skills to make Wikipedia a better evidence-based resource. Participants will not only learn to edit Wikipedia articles, but also become part of a community of Wikipedians dedicated to improving health information. Sign up for your Wikipedia account before the session starts so you can start editing right away! Bring a computer or tablet along with you for edits, and use #citeNLM to spread the word about your edits!

Monday, May 6, 2:00 p.m.–3:25 p.m.
Room: Columbus EF (East Tower, Ballroom/Gold Level)

Information Management Session 3

Moderator: Joe Swanson Jr., AHIP

Strengthening Institutional Partnerships to Support Clinical Research: Hosting a Clinical Data Training Series in an Academic Medical Center

Kevin Read, Lead, Data Discovery and Data Services Librarian, NYU Health Sciences Library, New York, New York

Background: An academic health sciences library, with experience providing clinical research data management (CRDM) and REDCap training to faculty, students, research coordinators and other staff, identified that attendees had a lack of knowledge about institutional clinical research support. Evaluation data from these trainings indicated that attendees had a desire to learn about advanced clinical research topics related to compliance, data transfer, statistics, data integrity, and Good Clinical Practice. To fill institutional knowledge gaps and satisfy training needs, the library spearheaded a Clinical Data Series by collaborating with stakeholders from across the institution to establish a holistic clinical research data training program.
Description: To lead this effort, the library partnered with stakeholders from the Office of Science and Research, General Counsel, Information Technology, Population Health, and Research Compliance and invited them to offer training alongside library-taught CRDM and REDCap classes as part of the Clinical Data Series. To improve institutional awareness of clinical research data support, the following classes were developed and taught by these departments in collaboration with the library: Interacting with Statisticians, Data Transfer, Research Compliance, Data Integrity and Preventing Data Loss, and Good Clinical Practice-Related Data Issues. Classes were assessed using an evaluation form administered at the end of each class. The evaluation form asked attendees if they would use what they learned, how they would specifically apply what they learned in their current role, and if they would recommend the class to others.
Conclusion: 190 people attended 9 classes. 181 attendees completed an evaluation form, with 97% stating they would use what they learned, and 91% stating they would recommend the class they took to others. The Clinical Data Series provided an opportunity to establish new partnerships across the institution with the end goal being that our research community is better trained, more compliant, and increasingly aware of established institutional clinical research workflows. With the library serving as the entrypoint for teaching researchers and identifying their data management concerns, it can serve as the institutional referral pipeline for ensuring clinical researchers are trained accordingly.

Building a National Training Program for Research Data Management

Jessi Van Der Volgen, AHIP, Assistant Director, University of Utah / NNLM Training Office, Salt Lake City, Utah

Shirley Zhao, Data Science Librarian, The University of Utah, Salt Lake City, Utah

Background: In August 2017, the NNLM Training Office (NTO) was awarded an administrative supplement to create the first Biomedical & Health Research Data Management (RDM) Training for Librarians program. The specific aim of this project was to improve the competency of information professionals in the area of biomedical and health science research data management through a rigorous educational experience.
Description: A data science librarian curated and organized existing RDM resources into an 8-week online modular course, developed, a capstone project and organized an in-person summit. A pre- and post-test assessed knowledge gained and post-course evaluations measured confidence gained in RDM expertise. A follow-up survey was conducted 4 months after the completion of the course to assess project progress, subsequent actions of students, and impacts to their institutions.
Results: Thirty participants completed the online coursework, a project, and gathered at NIH for the Capstone Summit. On average, students demonstrated improved knowledge of RDM concepts between the pre- and post-tests. Most students also self-reported increased skill and confidence. Hands-on, practical assignments with personalized feedback from experienced data librarians were the most valued aspect of the online course. Time required to complete weekly modules was underestimated and should be revised. Twenty (67%) of participants responded to the follow-up evaluation. Nearly all students agreed that the training increased their confidence in promoting data management services and in several other areas. At least of the respondents have used or plan to use the skills gained at their institution. Students who had completed at least half of their proposed project were more likely to have used or plan to use the skills and knowledge gained in the course.

Data Science Training for Library and Information Science Graduate Students and Practicing Health Sciences Librarians

Diane G. Schwartz, FMLA, Research Associate Professor, University at Buffalo, East Amherst, New York

Ying Sun, Associate professor, University at Buffalo, Buffalo, New York

Rajita Shukla

INTRODUCTION
The Department of Biomedical Informatics, and the Department of Information Science, University at Buffalo (UB), State University of New York, received funding from the National Library of Medicine (NLM) to develop curricula to teach graduate students and health sciences librarians (LIS) the skills needed to work in the data science ecosystem. The goal is to expand the employability of participants by offering them the opportunity to develop new expertise in one of five data science domains: data analytics, data management, data archiving/curation, data visualization, and terminology/ontology, and to receive micro-credentials for completing the training.
METHODS

The initial phase of the project identified the essential didactic areas. These were matched to UB's course catalog, and vetted for suitability and appropriateness. Courses not currently available at UB will be developed. Courses will be taught online. The rational for selecting the courses will be presented in the context of each micro-credential. Because UB views a micro-credential as a "credit-bearing program that may 'stack into' a larger certificate or degree program, it is essential to ensure that the courses meet the highest standards of excellence.
RESULTS
The environmental scan of UB's catalog identified 26 potential courses across all graduate programs. An assessment of each course narrowed down the number of useable courses to 8. UB and two other NLM informatics training programs are developing mini-courses to train health professionals data science skills. We will incorporate these courses into the training program, thereby creating a diverse educational experience to support the micro-credentials.
DISCUSSION
The data science program will offer new professional development and continuing education pathways for LIS students and professionals. Each micro-credential will prepare these individuals to support an institution's clinical and scientific research enterprise by providing new services and partnerships with data science researchers. The program also will retain a valued cadre of health sciences professionals who will contribute to the health of our communities, and preserve the well-respected capabilities for which librarians are known.
CONCLUSION
The curriculum will provide LIS students and professionals with the knowledge, skills, and attributes needed to work as data science librarians. The micro-credentials and digital badges will document each learner's knowledge, skills, and accomplishments.
FUNDING
This work was supported by NIH NLM T15 LM012595, along with T3 supplement, 3T15 LM012495-02S2.

Librarian Co-Teaching a Graduate Medical Education Research Practicum

Fred Willie Zametkin LaPolla, Research and Data Librarian, NYU Langone Health, New York, New York

Colleen Gillespie, Associate Professor, NYU School of Medicine, New York, New York

Background: Residents have limited time for research and working with librarians, and therefore are often not exposed to the full breadth of skills and services available from a library. Our academic medical center offers a dedicated 2-week Research Practicum, an immersive research experience for PGY3s in which they analyze a real dataset and produce an abstract for either a publication or conference presentation. Skills taught in the class are often possessed by librarians: literature review; data management, sharing, visualization and analysis. This paper presents a case study of a librarian's collaboration in teaching a GME Research Practicum for PGY3s.
Description: A librarian was invited by one of the Research Practicum faculty to join as an instructor. The librarian had experience teaching data visualization workshops, as well as working with residents to teach Evidence Based literature searching, and the practicum allowed for an opportunity to deepen engagement with residents and improve skills in data analysis. Two blocks of the Research Practicum were held, each with a group of four residents. The librarian joined brainstorm sessions and offered help finding relevant literature. Additionally, the librarian worked with the main professor to discuss methods

of analysis and visualization and to discuss with the residents ways to move past roadblocks in their research. Finally, towards the end of the Practicum, the faculty provided feedback on presentations and advised on improvements to be made before publication.
Conclusion: Residents who participated in the Research Practicum are currently revising abstracts and/or manuscripts for submission to national journals in their field. By MLA we expect to hear if they have been accepted. In addition to the publications, residents learned about issues relating to data sharing and reuse, data analysis and visualization, and research, and the library gained inroads into teaching and research with this group.

Current Practices in Data Management Education: Surveying Nursing Doctoral Programs Research

Rebecca Raszewski, AHIP, Associate Professor & Assistant Information Services & Liaison Librarian, University of Illinois at Chicago, Chicago, Illinois

Abigail Goben, Associate Professor, University of Illinois-Chicago, Chicago, Illinois

Martha Dewey Bergren, Director Advanced Population Health and Health Systems Leadership & Informatics, University of Illinois Chicago College of Nursing, Chicago, Illinois

Krista Jones, Urbana Campus Director and Clinical Associate Professor, UIC

Catherine Jean Ryan, Clinical Associate Professor, University of Illinois at Chicago, Chicago

Alana Steffen, Research Assistant Professor, Health Systems Science, College of Nursing

Susan Vonderheid, Clinical Assistant Professor, University of Illinois at Chicago, College of Nursing

Objectives: The inclusion of data management (DM) topics within nursing doctoral curricula has not been systematically examined. Collaborations between library and nursing faculty identified a need to understand DM in nursing doctoral programs. The purpose of this study will be to identify existing DM educational practices within nursing curricula.
Methods: This descriptive study will employ a survey of nursing doctoral program directors. A list of 338 doctoral programs was obtained from the American Association of Colleges of Nursing. An interdisciplinary team of library and nursing faculty created a survey based on the stages of the UK Data Archive data life cycle. Questions for the survey include how and by whom DM is being taught within the nursing doctoral curricula, the kinds of data students are using for their DNP projects or dissertations, how students' data are being preserved, and if there any data sharing policies in place once the students' projects are completed. This study was approved by the institution's IRB.
Results: There are a total of 75 responses received from both doctoral program directors. Only 2 indicated that no portions of the data life cycle were being taught. The collecting (n=60) and processing and analyzing (n=54) stages of the data life cycle were the most frequently reported areas of instruction. DM is most frequently taught through single lectures (n=39) or individual mentoring (n=43). Nearly half (n=35) reported a lack of institutional preservation storage solutions for project data. Half of the

responding program directors indicated that there were no requirements for supplemental files such as codes or data dictionaries to be retained.

Conclusions: Although nursing programs are providing instruction on data collection and processing and analysis, there are gaps in long term preservation, reproducibility, and institutional support.

Monday, May 6, 2:00 p.m.–3:25 p.m.
Room: Columbus GH (East Tower, Ballroom/Gold Level)

Information Services Session 3

Moderator: Martha Meacham

Leading Easy Access to Content: ra21's Final Recommendations and Insights from the RA21 Hospital Clinical Access Working Group

Michelle Mary Brewer, Librarian/Manager of Market Intelligence, Wolters Kluwer Health/Learning Research & Practice, Lawrenceville, New Jersey

Catherine Dixon, Product Manager, Wolters Kluwer, Norwood, Massachusetts

Jean P. Shipman, AHIP, FMLA, VP, Global Library Relations, Elsevier, Cottonwood Heights, Utah

Don Hamparian, Sr Product Manager, Platforms, OCLC, Dublin, Ohio

Background: RA21's goal is simple, trusted access—anywhere, anytime on any device. Publishers, libraries, and consumers should understand that authorizing access to content based on IP address no longer works in today's distributed world. The project resolved some fundamental barriers to moving to federated identity in place of IP address authentication. With its multi-stakeholder process, STM and NISO determined best practice for future implementations going forward. RA21's recommendations deliver seamless authentication as users move between online resources, while preserving anonymity. Independent of workflow or location, it enhances security, supports customizations and enables usage analytics. NISO's Recommended Practices document showcases this effort.
Description: Building on CNI's 2016 Report on the Authentication and Authorization Survey, and the 2015 P.D.R. pharma librarian initiative, the STM Association and NISO convened conversations with multi-stakeholders on how to improve the user experience. After 3 years of work and pilots, final reports and recommendations are available. The goal of more seamless access will enter its next phase. In 2019, the STM RA21 efforts will go to NISO for development of recommended practices.
This session will present select recommendations, data and themes from a few final efforts:
• "NISO Recommended Practices (RP) for Improved Access to Institutionally-Provided Information Resources"
• "GÉANT Data Protection Code of Conduct"
• RA21 Hospital Clinical Access Working Group "2019 Survey of Hospital/System Librarians Regarding Hospital/Clinical Access and RA21" conducted Feb.12 to March 22, 2019
Conclusion: RA21 program reports and recommendations from a Corporate Pilot, Academic Pilot and Security Group are included in the current NISO RP. From a thorough evaluation, the technical architecture prototyped was chosen and the security and privacy principles and practice were determined. Best practices for the future include 1) improved user login at publisher sites; 2) granular usage statistics; 3) set up/maintenance of Single Sign On with multiple publishers. The RA21 Hospital

Access Working Group began work in 2018, and is evaluating its survey and focus group data, writing recommendations and a final report. It will include defining the unique use case and challenges for 'single sign-on' SSO and SAML technology within a hospital/healthcare system for accessing licensed resources, and adopting RA21 as an access strategy.

Biomedical Preprints and the Future of Scholarly Communication: The Librarian's Role

John Gallagher, Director, Cushing/Whitney Medical Library, Yale University, New Haven, Connecticut

Lindsay Barnett, Collection Development & Scholarly Communication Librarian, Yale University, Branford, Connecticut

Kate Nyhan, Research and education librarian, Yale University, New Haven, Connecticut

Ginger Gamble, Research Assistant II, Yale School of Medicine

Holly K. Grossetta Nardini, AHIP, Associate Director, Cushing/Whitney Medical Library, Yale University, New Haven, Connecticut

Background: In the physical sciences, preprint servers are well established and are facilitating rapid dissemination of new findings. In the biomedical sciences, preprints have lagged, partly due to concerns about human health. The launch of medRxiv portends growth in the use of preprints in medicine and a new era of openness, discoverability, and impact for preprints. In order to advise and guide our clientele, librarians must be versed in the advantages of biomedical preprints and should understand the role such articles play within the larger scholarly communication ecosystem and our own research.
Description: As the dissemination of medical research through preprints evolves, medical librarians can contextualize preprints in the larger ecosystem of scholarly communication, along with trial registrations, pre-registered protocols, conference papers, open data, and peer-reviewed journal articles. Medical librarians at Yale anticipate the further integration of biomedical preprints in many services we already provide. We recommend and discuss the advantages and disadvantages of preprints with authors. We suggest that authors include preprints in NIH applications and other records of scholarly impact. We advocate searching preprint servers when conducting systematic reviews. We help students and researchers develop information literacy skills to critically appraise preprints. We have started depositing complex search strategies and other interim research projects of our own on Open Science Framework, as part of our commitment to openness and reproducibility.
Conclusion: Biomedical preprints are poised to become common in the health sciences, providing a new mechanism by which researchers share, communicate, and receive feedback about their work. Librarians will need to be familiar with the use of preprints and be able to position them in the general – and quickly evolving – world of scholarly communication. Librarians will also benefit from understanding how preprint servers can help their own work and underscore a commitment to open access.

Transparency in Publishing: How to Best Inform the Journal Selection Process

Taneya Y. Koonce, Associate Director for Research, Center for Knowledge Management, Strategy and Innovation, Vanderbilt University Medical Center, Nashville, Tennessee

Mallory N. Blasingame, Information Scientist, Center for Knowledge Management, Strategy and Innovation, Vanderbilt University Medical Center, Nashville, Tennessee

Annette M. Williams, Senior Information Scientist, Center for Knowledge Management, Vanderbilt University Medical Center, Nashville, Tennessee

Jerry Zhao, Senior Application Developer, Center for Knowledge Management, Vanderbilt University Medical Center, Nashville, Tennessee

Zachary E. Fox, Associate Director for Information Services, Center for Knowledge Management, Vanderbilt University Medical Center, Nashville, Tennessee

Elizabeth T. Frakes, Information Scientist, Center for Knowledge Management, Strategy and Innovation, Vanderbilt University Medical Center, Nashville, Tennessee

Spencer J. DesAutels, Information Scientist, Center for Knowledge Management, Vanderbilt University Medical Center, Nashville, Tennessee

John D. Clark, Sr Application Developer, Center for Knowledge Management, Vanderbilt University Medical Center, Nashville, Tennessee

Dario Giuse, Associate Professor, Vanderbilt University Medical Center, Nashville, Tennessee

Nunzia B. Giuse, FMLA, Vice President for Knowledge Management | Professor Biomedical Informatics, Medicine, Strategy & Innovation, Vanderbilt University Med. Ctr., Nashville, Tennessee

Background: The advent of open access publishing afforded unparalleled opportunities for increasing the accessibility of scientific research for public and academic community benefit. Yet, its exploitation led to alarmingly high rates of publishing behavior that disrupts the integrity of the scientific communication process. In absence of rigorous peer review and transparent publishing/editorial practices, the impact, visibility, and reproducibility of scientific research suffer. Experienced librarian and information science professionals are well-equipped to advise our communities on recognizing and avoiding deceptive techniques using well-established evaluation methodologies.
Description: Given the strategic role of academic publishing in the advancement of science, it is troublesome to see in the publishing industry an ever-increasing number of journals lacking the necessary transparency required of a rigorous and established scholarly communication process. Recognizing the surfacing of this problem, multiple publishing organizations, in the attempt to guarantee the value and trustworthiness of published scientific data, developed resources/checklists to aid authors in evaluating journals' adherence to publication best-practices. Using a semi-automated process, the Vanderbilt University Medical Center (VUMC) Knowledge Management (KM) team is

developing a framework to link existing well-established tools developed by highly reputable organizations (e.g., Directory of Open Access Journals; Think, Check, Submit), to create a support infrastructure for authors seeking transparency to inform their publication decisions. The framework will use well-known tested criteria to provide authors with journal assessments and greatly facilitate the journal selection process.
Conclusion: To best establish the editorial rigor of a journal, the team created a VUMC KM journal record comprised of more than 20 fields, each representing distinct publishing transparency criteria. Using semi-automated approaches, information is compiled from multiple sources, including the National Library of Medicine and publisher websites. To reach critical mass, database fields were prioritized for data population based on information most critical for authors' journal selection decision-making. A detailed review of the first 500 journals selected for analysis, where VUMC authors most frequently published, showed that transparent definitive answers for all targeted criteria were available for approximately 74% (359/500). A majority (357/369; 97%) were indexed in MEDLINE and the most common criteria lacking definitive information was a statement of adherence to the International Committee of Medical Journal Editors' Recommendations for the Conduct, Reporting, Editing and Publication of Scholarly Work in Medical Journals. The project's resultant infrastructure and end-user interface reflect a knowledge management approach that can be leveraged by authors and information professionals for journal identification and evaluation.

From Global to Local, a Case Study in Using 101 Innovations in Scholarly Communication Research

Madeleine Bruwer, Subject Librarian, Monash University, Parkville, Victoria, Australia

Penny Presta

Cassandra Freeman

Objectives: This project set out to develop our research skills, inform our professional practice and strengthen our capabilities to align with the strategic objectives of a research intensive university. We embarked on practitioner based research, utilising a globally conducted survey to determine the tools used in the evolving scholarly communication workflows of our early career researchers in the health sciences.
Methods: Our project team contacted the creators of "101 Innovations in Scholarly Communication" and received permission to utilise their survey. Ethics approval was obtained and the survey re-created using Qualtrics. Permission was sought from the Faculties of Medicine, Nursing & Health Sciences, and Pharmacy & Pharmaceutical Sciences to distribute the survey. Approximately 1140 researchers received an invitation via the assigned faculty mailing lists. Eighty-five researchers initiated the survey and 84 completed it, yielding a participation rate of 7.36%. Survey results were analysed using SPSS software. Of those respondents who completed the survey, 18 indicated that they were willing to be contacted further, and eight semi-structured interviews have been conducted, recorded, and analysed in NVivo.
Results: Results indicated that although early career researchers are overwhelmingly in support of open access and open science, tension exists due to the expectations associated with advancing their careers. These attitudes signal that key developments in scholarly communication in coming years will

foreseeably be related to open access. To remain relevant and prepare ourselves for future roles, librarians need to be cognisant of the big changes in practices ahead.
The project connected theory to practice and highlighted gaps in existing knowledge. By presenting the project to colleagues and sharing newly gained knowledge, the librarians initiated dialogue around new developments such as open peer review, preprints and reproducibility.
Conclusions: Librarians must increasingly adapt and take control of their professional development in order to remain relevant within a changing university environment. In addition to maintaining familiarity with scholarly communication tools and other factors that contribute to the openness of research, the project resulted in increased visibility and standing within the organisation. It enabled librarians to expand their networks and afforded opportunities to collaborate externally. The librarians have been invited to contribute and participate in a number of projects, committees and working parties, and to facilitate training sessions for peers and faculty.

EEK! Is Everything Predatory? Elevating the Conversation about Predatory Publishing and the Ethics of Scholarly Communication

Kathleen Elizabeth Phillips, Nursing & Allied Health Liaison Librarian, Penn State University, University Park, Pennsylvania

Christina L. Wissinger, Health Sciences Librarian, Penn State, University Park, Pennsylvania

Background: The world of poor and predatory scholarly communication now extends from traditional publishing to conferences and phishing emails, and a disturbing trend in academia of knowingly publishing in low quality resources. How do librarians serve as agents of change in protecting our patrons from predatory bodies and scholarly misconduct? We arm them with knowledge.
A lecture and discussion targeting faculty and graduate students was created to teach the methods of evaluating unknown publications or conferences, recognize poor quality and predatory practices, and discuss ethical scholarly communication and researcher reputation issues.
Description: In 2016, the librarian was invited to talk about predatory journals. She developed a 60-minute lecture consisting of an introduction to Beall's List, the Directory of Open Access Journals, and tips for evaluating journal quality. In 2017, after the dissolution of the Beall's List, this lecture was elevated to a 90-minute talk adding active learning and the discussion of ethics in scholarly communication.
Participants receive three articles prior to the seminar. During the session, they learn about the steps to determine a publication, conference, or email invitation's credibility, and judge examples of good quality, poor quality, and predatory materials. Key points of discussion focus on several topics, including hijacked identities (i.e. journal titles), protecting author reputation, and the role scholarly communication plays in open access and predatory publishing. The seminar concludes with the discussion of the ethics of scholarly communication.
Conclusion: Seminar attendees leave with a strong understanding of the differences between good quality and poor quality open access resources, the essential elements of evaluating resource quality and credibility, and avoiding inadvertently publishing in a predatory or poor quality journal. The product of this lecture is a robust discussion about ethical decisions in scholarly communication and the affects publishing in a predatory or poor journal can have on their reputation as researchers. They leave with answers to their questions, and a realization of the vital role they play in the future of scholarly communication.

Monday, May 6, 2:00 p.m.–3:25 p.m.
Room: Grand Ballroom A (East Tower, Ballroom/Gold Level)

Innovation & Research Practice Immersion Session 3

Moderator: Jolene M. Miller, AHIP

Closing the Circle with Reflection: Elevating Professional Practice

Jolene M. Miller, AHIP, Director, University of Toledo, Mulford Health Science Library, Toledo, Ohio

Stephanie Friree Ford, Manager, Library Resources, McLean Hospital, Belmont, Massachusetts

Anna Yang, AHIP, Health Sciences Librarian, California Health Sciences University, Clovis, California

Elena Azadbakht, Health Sciences Librarian, University of Nevada, Reno, Reno, Nevada

Lin Wu, AHIP, Assistant Director for Research & Learning Services, University of Tennessee Health Science Center, Memphis, Tennessee

Jolene M. Miller, AHIP, Director, University of Toledo, Mulford Health Science Library, Toledo, Ohio

Jolene M. Miller, AHIP, University of Toledo, Toledo, Ohio
A recent survey of health science librarians found that some of the common barriers to reflective practice were knowledge and skills (Miller, manuscript in development). The goal of this experiential immersion session is to give participants the knowledge, skills, and confidence to engage in reflective practice in their work. Participants will learn about and practice reflective writing and conversation and hear from a panel of librarians who engage in reflective practice. At the end of the session, participants will be able to (1) identify key components of reflective practice from published models; (2) apply the SEA-change model with reflective writing; (3) have a reflective conversation with a colleague; and (4) identify potential barriers and ways to overcome these barriers to using reflective practice. The session is relevant for any conference attendee, regardless of work environment, length of time in the field, or position. All of us can use reflective practice as a tool to improve our professional knowledge and skills. No prior knowledge or skills are required.
Keywords: reflection, reflective practice, reflective writing, peer conversation, professional development

Monday, May 6, 2:00 p.m.–3:25 p.m.
Room: Columbus KL (East Tower, Ballroom/Gold Level)

Lightning Talks 3

Moderator: Amanda Adams

Librarian Partnerships in a Program-Wide Systematic Review Capstone Project: Elevating Team Work

Leila Ledbetter, AHIP, Research and Education Librarian, Duke University Medical Center Library & Archives, Durham, North Carolina

Sarah Cantrell, Associate Director for Research & Education, Duke University, Durham, North Carolina

Brandi Tuttle, AHIP, Research & Education Librarian, Duke University, Durham, North Carolina

Amanda Woodward, Research & Education Librarian, Liaison to the School of Nursing, Duke University Medical Center, Durham, North Carolina

Megan G. Van Noord, Health Sciences Librarian, University of California, Davis, Davis, California

Emily S. Mazure, AHIP, Clinical & Research Librarian, Mountain Area Health Education Center Library, North Carolina

Background: Building upon existing relationships with the faculty, the liaison librarian to the Doctor of Physical Therapy (DPT) program set out to streamline the required systematic review project in order to help the large number of student groups (15 in 2018) simultaneously meet assignment benchmarks on a four to six week timeline. Due to the time-intensive nature of systematic reviews and the time-sensitive nature of the course, it became clear that the liaison librarian would need to involve all Research & Education Librarians. This talk describes how a librarian team approach allowed for successful completion in the demanding timeframe.
Description: The liaison librarian is embedded in the DPT Evidence Based Practice course and teaches sessions on developing clinical and research questions using PICO, advanced literature searching strategies, and systematic review methodology.
Faculty mentors require students to meet with an assigned librarian. The DPT liaison librarian matched each group to a librarian, provided an overview session to participating librarians, and developed guidelines and expectations for working with the student teams. On average, librarians worked with three student groups. Librarians provided in-depth consultations with their teams in order to further explain the systematic review process which included reviewing the PICO-formatted questions, discussing protocol registration in PROSPERO, and teaching the software for title/abstract screening, as well as citation management. Librarians conducted exploratory and final searches, drafted search

appendices, and managed citations in both the title/abstract screening software and the citation management tool.
Conclusion: Success can be attributed to building strong interprofessional relationships with the faculty and students that allowed librarians to set expectations and provide clear guidance to help students complete advanced preparation for each of the steps in the projects. It was essential to involve all liaison librarians at the library in order to balance the workload of simultaneous in-depth projects.

Promoting Evidence-Based Literature through Journal Clubs in Residency Programs

Kristin M. Chapman, Clinical Librarian, Howard University, WASHINGTON, District of Columbia

Background: To keep up with the ever-expanding body of medical knowledge for the better care of patients, physicians need to be lifelong learners. Journal clubs are the most effective technique in which to teach evidence-based medicine during residency, and their origins in the late 1870s emphasize the sources of information in which we teach critical appraisal skills.
Description: Most residencies and GME committees require a standard in which scholarship needs to take place, and librarians participating in journal club provide the foundations to offer strategies on how to retrieve reliable information to address patient problems. Librarians will promote their services to support Chief Residents in teaching a skillset of evaluating scientific papers prior to formalizing a journal club. After relationships are created to encourage responses, a Hartzell survey will be sent to define characteristics of practice and general study opinions. The librarian will then sit in to provide supervision or instruction, offer help with retrieving articles that were chosen, and guide journal club members to high quality research. After 6 months of the commencement of librarian-assisted journal clubs, a repeat survey will be sent to participants about opinions the changes in journal club and compare the results.
Conclusions: We are hoping to reinforce that journal clubs are an integral part of residency education but that there is component missing in non-librarian assisted journal clubs. Librarians can manage the overabundance of information, overcome barriers of find and disseminate articles, and be an overall invaluable educational supporter to create the next generation of physicians. This study aims to show that under the guidance and supervision of a trained librarian, it is feasible to produce a successful journal club.

Informational Needs of Individuals Who Experienced a Terminal Cancer Diagnosis of a Loved One: An Exploratory Research Study

Megan Coder, Associate Librarian, State University of New York at New Paltz, New Paltz, New York

Objectives: This exploratory research investigated the information needs of family of terminal cancer patients through quantitative methods. The definition of "terminal cancer" for this study was a prognosis that included a predicted lifespan (e.g. 5 months, 2 years, etc.).
Methods: This study was approved by the Human Research Ethics Board at the State University of New York at New Paltz. An online survey was designed using Qualtrics that consisted of 40 questions with a combination of Likert-scale and open-ended questions. It was administered to those who dealt with a terminal cancer patient. The survey explored the types of information resources that were used,

anticipated information uses, obstacles in obtaining information, and fluctuation of information needs. The survey was distributed to a university listserv, library organization listservs and message boards, and local medical facilities with an average of 63 responses per question. Participation was anonymous and no identifying information was collected. Survey responses were collected between August 1, 2018 – November 30, 2018.
Results: Most respondents were parents (31%) and children (24%), with 45% representing other family members like spouses/partners, siblings, grandparents or grandchildren. The data revealed that respondents always used doctors (41%), family (36%), and nurses (32%) as their main information resources followed by often used resources like medical websites (45%), cancer organization websites (35%), and Google (33%). The least used resources were phonebooks (90%), Twitter (89%), Snapchat (89%), and Instagram (88%). The data suggested that respondents used the information in order to better understand the disease (32%), cope with illness (26%), communicate with physicians (23%), and to make medical decisions (17%).
Conclusions: Results suggest that family members of terminal cancer patients have vast and diverse information needs that may vary along the cancer journey. The resources frequently used among this population are similar to those used by cancer patients, such as physicians, other health-care professionals, and internet resources. Family members have information needs that pertain to themselves and also the cancer patient. As the results indicate, they use the information for their own understanding about the disease and treatments and to seek clarification from the physicians. Family members also use the information on behalf of the cancer patient since they are often their advocates and responsible for many of the medical decisions.

Using a Poster and Survey Model to Reach New Heights at Library Orientation

Gary S. Atwood, Health Sciences Education Librarian, University of Vermont, Burlington, Vermont

Nancy A. Bianchi, Research & Education Librarian, University of Vermont Dana Medical Library, South Burlington, Vermont

Background: Although active learning techniques have the potential to enhance the learner's experience, it can be difficult to efficiently and effectively incorporate them into unstructured events outside of the classroom such as an orientation fair. This presentation will show how we took a successful poster and survey activity used by academic medical librarians at a community career fair and then adapted it for graduate medical education orientation fairs. We designed a simple poster along with a short survey to help us actively connect with small groups of new medical residents while introducing them to library resources and services.
Description: Since 2011, librarians have participated at an annual community career fair. Despite being creative with themes at the fair, our exhibit was largely overlooked by disinterested students due to its lecture-based format until its redesign as an active learning poster and a short survey activity. The poster's information and graphics did all the "talking" while students were asked to "help" the librarians by reading and critiquing the poster information using a short survey. The success of the poster and survey in the community encouraged us to try the same model with new medical residents at their orientation fair. The redesign prompted more questions and interesting conversations among residents and librarians than in the past. Preliminary review of three years of survey data has revealed an 80% survey completion rate with 100% satisfaction with the poster as an active learning tool.

Conclusion: Survey results suggest that the poster and survey model resulted in students retaining more information about the library resources and services while expressing greater satisfaction with this teaching format. Dana Medical librarians continue to use this active learning activity, to study its results, and to build on its success with other library presentations.

Boosting the Work of Librarians in Community Health Information

Margot G. Malachowski, AHIP, Education & Outreach Coordinator, National Network of Libraries of Medicine, New England Region, Worcester, Massachusetts

Background: The National Network of Libraries of Medicine, New England Region (NNLM NER) provides training, funding and support for health information outreach. To raise awareness of these opportunities, NNLM NER conducts annual Focused Outreach (FO) initiatives. FO targets two geographic areas each year, one rural and one urban. NNLM NER contacts librarians, educators, health care providers, public health workers and/or community leaders to identify key informants. NNLM NER conducts eight-twelve interviews before scanning available data to identify health priorities. FO includes synthesizing community observations of health information needs; identifying target populations and health information foci; and designing health information outreach strategies. Evaluation is based upon process assessment, noting changes in participation rates in NNLM NER activities.
Description: For Y3, NNLM NER conducted FO in Bangor, ME with a spotlight on the work of librarians. NNLM NER interviewed nine librarians at various locations, including two hospital libraries, two urban libraries, two rural libraries, a community college library and a state library. NNLM NER looked at data from the U.S. Census, Annie E. Casey Foundation, Robert Woods Johnson Foundation, local newspapers and local community health needs assessments. Librarians identified these health priorities: mental health; disability; substance use; sexual abuse; violence; trauma; poverty and suicide. Data supported these reports, and revealed concerns about child welfare, obesity, alcohol-impaired driving and sexually transmitted infections. Upon recommendation of library personnel at Maine State Library, Bangor Public Library and Eastern Maine Community College, NNLM NER identified four strategies: database training for academic and hospital libraries; training on compassion fatigue for public library staff; technology funds for community college library; and professional development reimbursement for library staff.
Conclusion: NNLM NER provided tailored PubMed webinar training for twenty librarians. NNLM NER funded Bangor Public Library staff training on compassion fatigue to provide information on how mental health, trauma, homelessness and substance use disorder impacts the community. The training, conducted by local social service agencies, raised awareness of self-care methods that enable good customer service. Library staff at Eastern Maine Community College acquired tablets to be used in the promotion of health resources. One Eastern Maine Community College librarian received funding to attend a national conference. Three Bangor-area librarians registered to attend the NNLM NER funding meeting for Y4, in contrast to zero participants in Y3 and five participants in Y2. FO increased participation in training but not requests for funding in Y4.

Supporting Data Harmonization and Discovery in the BRAIN Initiative

Alisa Surkis, Assistant Director, Research Data and Metrics/Vice Chair for Research, NYU Health Sciences Library, New York, New York

Kevin Read, Lead, Data Discovery and Data Services Librarian, NYU Health Sciences Library, New York, New York

Background: The NIH BRAIN Initiative is a program to fund the development and application of innovative technologies to aid in understanding the human brain. In 2017, it began funding streams to support: 1) the development of data archives, standards, and tools; 2) research teams studying brain circuit functions underlying behavior. Data science cores were required as part of the circuit function grants, and were tasked with ensuring that the FAIR Principles were considered with respect to the data collected. An NIH-supported data science consortium included the directors of the data science cores to promote collaboration and sharing of tools and resources.
Description: A librarian in an academic health sciences library serves as the data science core director for one of the circuit function grants funded in 2018, with a second librarian serving as key personnel on the core. Work within our project team includes generalizing a data model developed in one lab to capture metadata of all participating labs, and assessing data and metadata practices across these labs to improve metadata collection and harmonization and reduce the impact on existing research workflows. Across the data science consortium, we have brought a discovery-oriented perspective; there is is only one librarian among the data science core directors; the others represent the following disciplines: neuroscience (4), computer science/statistics/engineering (6), and physics (2). This has resulted in an exploration of the use of a data catalog to improve the discovery of BRAIN Initiative data.
Conclusion: The FAIR Guiding Principles address data findability, accessibility, interoperability, and reusability. While the BRAIN Initiative is funding the development of repositories, standards, and tools, which address the latter three principles, librarians bring an otherwise missing focus on discovery, and were able to propose a tool to improve findability of BRAIN Initiative datasets. Within their project team, the librarians partnered with a domain expert who had developed metadata that served the needs of a single lab, in order to generalize the metadata schema to effectively capture the metadata from all project labs.

On the Same Page: Aligning Librarians' and Requestors' Relevance Perceptions to Improve the Search-and-Weed Process

Jane Morgan-Daniel, AHIP, Community Engagement and Health Literacy Liaison Librarian, University of Florida, Gainesville, Florida

Nancy Schaefer, AHIP, University Librarian, University of Florida, Gainesville, Florida

Linda Struckmeyer, Clinical Assistant Professor, University of Florida, Gainesville, Florida

Luther King

Christine T Myers

Shabnam Medhizadah

Mary Jeghers

Jason Beneciuk, Research Assistant Professor, Department of Physical Therapy, University of Florida, Gainesville, Florida

Objectives: Lack of a comprehensive database on allied health educational topics challenges those seeking such research. Searching multiple discipline-specific resources complicates database selection and increases duplicate and irrelevant search results. To identify effective techniques for reference-interviewing, selecting databases, and weeding results, this study assesses 1) alignment between librarians' and requestors' relevance judgments of search results and 2) factors influencing relevance perceptions.
Methods: Occupational Therapy, Physical Therapy, and Rehabilitation Science faculty/practitioners contributed six research questions on instructional methods and materials for patients (n=2 questions), caregivers (n=2), and allied health professions students (n=2). Two librarians searched five databases using available controlled vocabulary and search functions. Requestors evaluated their own search results for relevance using a rubric based on PICO that included check-boxes and open-response boxes for comments. Both librarians individually evaluated all results using the same rubric. Comparison of the requestor and averaged librarian relevance scores and thematic analysis of comments prompted revision of the rubric to account for factors that unexpectedly influenced relevance perceptions. The revised relevance rubric was tested against the results of searches in six new databases on the same six research questions. Finally, the eleven databases were ranked by percentage of unique relevant search results.
Results: Librarians tended to judge more references irrelevant than requestors. For three questions, librarians marked nearly 6% more references irrelevant, although for one caregiver education question librarians marked 12% more references relevant. Thematic mapping identified three factors influencing differing relevance perceptions: varying terminology definitions, unexpressed information needs, and unanticipated intended information use. Revision of the rubric enabled the librarians to more closely align their relevance judgments with the requestors during the second searching/assessment round. Overall, CINAHL and PubMed appear the most useful resources for patient and student education questions, and ERIC for caregiver education questions.
Conclusions: Optimal database selection and results weeding technique vary by requestors' relevance criteria and the group targeted for educational intervention (patients, caregivers, students). Reference interviews should determine intended information use, ideal specificity of results, and terminology definitions (the latter still evolving in newer fields like rehabilitation). Librarians should clarify the discipline of allied health practitioners providing educational interventions, the time-frame of interventions, and the education-level of students receiving interventions. In weeding, librarians should err towards inclusivity when assessing results relevance. This study indicates that time spent discussing relevance criteria can result in closer alignment of requestors' expectations to librarians' search-and-weed efforts.

Role of an Academic Medical Library in Research Impact and Evaluation

Arian Abdulla, Research Librarian, University of Virginia Health Sciences Library, Charlottesville, Virginia

Andrea H. Denton, Research & Data Services Manager, University of Virginia Health System, Charlottesville, Virginia

Background: Publications and citation data have increasingly been applied as performance indicators in the context of research evaluation. Bibliometric services create an opportunity for librarians to expand their offerings to their constituents and thus raise the importance of the libraries within the institution. An academic medical library developed resources and services that support research impact and evaluation work for researchers and health care professionals at the university.
Description: The library has developed a portfolio of bibliometric services designed to aid researchers, clinicians, program administrators, and university leadership understand the applications and limitations of bibliometric data, as they seek to highlight the impact of their research. The library's current service includes a customized Citation Analysis Report that includes author, article, and journal-level metrics such as citing articles, citing patents, funding sources, inter-institutional collaborations, Relative Citation Ratios, and Impact Factors. Data are retrieved from Web of Science, iCite, and lens.org, among other sources. Reports and consultation services for bibliometric and research impact have been provided to university faculty, departments and groups.
Conclusion: This talk will describe the report types requested, workflows related to preparation, and an overview of typical report content, particularly focusing on analyses based on Web of Science data.

Confronted by Copyright: Creating Social Media Policies in the Face of Litigation

Margaret Emily Ansell, AHIP, Nursing & Consumer Health Liaison Librarian, Health Science Center Libraries, University of Florida, Gainesville, Florida

Melissa L. Rethlefsen, AHIP, Associate Dean and Fackler Director, University of Florida, Gainesville, Florida

Background: In 2019, the president of a large public university was approached by the copyright enforcement company Pixsy seeking compensation for the use of a stock photo by the health science library's marketing team. This request was passed through the university's legal department to the health science library, which had to quickly assess the nature and validity of the claim. The health science library director and the head of marketing collaborated with the university libraries' social media manager and administration to determine a course of action, both in handling the specific case and in setting policies to prevent future infringement claims.
Description: Despite being well-versed in copyright issues, and having trained library staff and faculty in the appropriate use of images for social media purposes, the health science librarians faced several challenges in resolving the infringement claim. These issues included 1) uncertainty about the sourcing of images used in social media messaging, 2) a university legal department that was unwilling to debate claim's validity or provide clarity for future policy making, and 3) the larger implications of utilizing aggregators of public domain images and GIFs, such as Pexels, Pixabay, and GIPHY. This incident also raised the issue of the application of fair use doctrines in the realm of social media, which is simultaneously permanent and ephemeral. Ultimately, this incident sparked a series of conversations across the libraries to clarify the use of images in social media and other marketing efforts.
Conclusion: To educate university libraries' staff involved in social media efforts about this issue, the new scholarly communications librarian collaborated with the libraries' social media manager to create and host a workshop on copyright and social media. Larger conversations with the university's social media coordinators and legal department are also planned.

Library Support for Faculty Recruitment Efforts

Andrea H. Denton, Research & Data Services Manager, University of Virginia Health System, Charlottesville, Virginia

Arian Abdulla, Research Librarian, University of Virginia Health Sciences Library, Charlottesville, Virginia

David Martin, Clinical Data Research Specialist, University of Virginia Health System, Charlottesville, Virginia

Jay Scott, Associate Partner, Opus Partners, Charlottesville, Virginia

Background: Recruiting a qualified and diverse workforce is a goal of any organization. An academic medical center administrator became aware of the NIH Scientific Workforce Diversity Toolkit as a method to expand faculty diversity. The Toolkit protocol is a multi-step process that includes identifying potential candidates in a systematic and unbiased way through a literature search, so the administrator contacted the library for support for faculty hiring efforts. While librarians did not contribute to diversity-related aspects of the search, they assisted by generating names of potential candidates by performing literature searches, filtering results, and refining deliverables for use by search committees.
Description: Librarians worked with two search committees seeking new clinical faculty. Librarians adapted the Toolkit protocol, which included guidance for conducting a bibliometric, person-blind search for most qualified authors in a field. Using Web of Science and PubMed, librarians worked with search committee leaders to create a search strategy to identify candidates based on keywords related to the position. Challenges faced included the broad topical nature of the searches which retrieved large sets of results. Librarians employed criteria such as number of publications, number of citations, funding amounts, and others to ultimately produce a workable list of potential candidates. Library data specialists devised scripts to categorize results for usability by the search committee. However, not all search requirements (e.g. candidates with a minimum rank, experience, and/or degree), can be easily described through search strategies.
Conclusion: Librarians can help with the Toolkit goal to assemble a comprehensive pool of potential recruitment candidates in a systematic and unbiased way. In one case, search committee leaders felt search results could be useful in generating a list of potential candidates, identifying potential nominators (of qualified candidates), and prioritizing candidates the committee has identified through other means. Assisting with recruitment efforts may be a novel way librarians can involve themselves in strategic efforts at their institutions.

#metoo and medlibs: How to Start Conversations about Sexual Harassment in Your Library

Jill Barr-Walker, Clinical Librarian, ZSFG Library, University of California, San Francisco, San Francisco, California

Background: The #metoo movement has facilitated national conversations about sexual violence, including workplace sexual harassment. Although anecdotal evidence suggests that many library workers experience sexual harassment, no comprehensive studies exist and no professional guidelines or recommendations are available for those interested in creating change in their libraries. How do we begin these conversations in our profession and our libraries?
Description: This talk will describe several recommendations for starting the process of addressing sexual harassment in your library. These recommendations are based on recent work done by the Sexual Harassment Task Force at [Institution] Library, including a library-wide survey to explore staff experiences of sexual harassment and educational efforts based on these survey results.
Conclusion: The purpose of this talk is to empower library workers to start conversations about sexual harassment in their libraries.

Build the Library Brand through the Power of Storytelling

Janice Marie Young, Director, Veterans Affairs Central Office Library, Arlington, Virginia

Background: This talk will help participants make the connection between the art of storytelling and brand development. Just as for millennia families have used storytelling as a teaching tool to pass on information and knowledge to the next generation. This talk will illustrate that storytelling is a powerful strategy in brand development for libraries to make the user experience significant and unforgettable.
Description: To introduce a framework to improve communication, increase the visibility of the library, and writing an elevator speech that is an authentic reflection of the library.
Conclusion: At the conclusion of the lighting talk, participants will discovery that storytelling is a powerful strategy in brand development to show the relevance and value that libraries and librarians deliver to their communities.

Coping Strategies for Impostor Phenomenon among Health Sciences Librarians

Debra Werner, Director of Library Research in Medical Education, John Crerar Library, Chicago, Illinois

Michelle B. Bass, AHIP, Manager, Research and Instruction, Harvard Medical School, Countway Library of Medicine, Boston, Massachusetts

Liz Kellermeyer, Biomedical Research Librarian, National Jewish Health, Denver, Colorado

Jill Barr-Walker, Clinical Librarian, ZSFG Library, University of California, San Francisco, San Francisco, California

Objectives: Impostor phenomenon is the inability to internalize accomplishments while experiencing the fear of being exposed as a fraud. Impostor phenomenon can have serious adverse effects including: anxiety, depression, lack of confidence, decreased job satisfaction and performance, and burnout. Our research objective is to discover effective coping strategies to help health sciences librarians address feelings of impostor phenomenon.

Methods: In our study, the first to measure impostor phenomenon in health science librarians, we distributed an anonymous online survey via REDCap to measure impostor phenomenon among US health sciences librarians and learn about strategies to cope with impostor phenomenon. We used the Harvey Impostor Phenomenon scale (higher scores are associated with feelings of fraudulence) and asked two open-ended questions about coping with feelings of inadequacy at work. The first question asked what strategies respondents used to address these feelings, and the second asked how effective the respondents believed the strategies were. We performed a thematic analysis of the responses to the open-ended questions.
Results: Our study found that one in seven health sciences librarians experienced impostor phenomenon and, importantly, younger and newer librarians had higher impostor scores. 703 participants completed the survey, of those, 459 answered the open-ended questions. Participants reported many strategies to cope with feelings of inadequacy; 75% reported their strategies were effective. We categorized strategies as either “external” (seeking help outside oneself) or “internal” (coping alone). External strategies include: educational opportunities, mentorship, and talking to others. Internal strategies include: avoidance, “fake it til you make it”, or mindfulness. We found that external strategies were associated with lower impostor scores.
Conclusions: Feelings of impostor phenomenon can be harmful to those who experience them. Individual librarians, managers, and professional organizations all have a role in addressing impostor phenomenon in the profession. Recognizing the signs of impostor phenomenon and understanding the most effective strategies to cope with it, such as mentoring, colleague support, and educational efforts, are beneficial to health sciences librarians, particularly to younger or newer librarians who experience more feelings of impostorism.

Monday, May 6, 2:00 p.m.–3:25 p.m.
Room: Columbus IJ (East Tower, Ballroom/Gold Level)

Professionalism & Leadership Session 5

Moderator: Jeffrey G. Coghill, AHIP

A Year of Creative Assessment: Incorporating Micro-Assessments into the Library

Shalu Gillum, AHIP, Head of Public Services, University of Central Florida College of Medicine, Orlando, Florida

Natasha Williams, AHIP, User Services Librarian, University of Central Florida College of Medicine, Orlando, Florida

Terri Gotschall, AHIP, Scholarly Communications Librarian, University of Central Florida College of Medicine, Orlando, Florida

Background: The library team endeavored to find creative ways to better assess library users' needs and to get real-time responses in order to shorten the interval between deploying surveys, gathering responses and implementation of solutions. In the past the library employed email surveys to assess user needs but the process was inefficient and ineffective due to low response rates and time spent creating online surveys.
Description: Over the course of one year, the library team used the following simple micro-assessments: Free response using sticky notes, short paper surveys, LibGuide polls, and a HappyOrNot terminal. For the free response assessment, boards were set up outside the library with different questions (e.g., "How can we improve your library experience?"). Sticky notes and pens were left for users to provide answers. Short paper surveys of no more than five questions, were deployed throughout the year to students and faculty. Completed surveys were collected and results tabulated the same day. A one-question poll was added to the end of LibGuide pages to assess whether the information provided on the guide was helpful. Finally, the HappyOrNot terminal was used regularly to gauge users' opinions on a variety of topics, for example "Should food be allowed in the library quiet room?"
Conclusion: These creative solutions to gather library user feedback have informed policy decision-making, and created opportunities for new services and programming. The free response survey revealed new user needs, for example access to monitors to use with laptops. Results of the paper surveys and HappyOrNot helped librarians understand students' use of library-issued iPads, informed policy decisions in regards to allowing food in the library quiet room, and led to the development of short presentations on scholarly communication topics and EndNote training sessions. Overall, using these micro-assessments provided a vehicle for librarians to listen to users' needs and develop solutions more quickly.

Faculty Status: Academic Elevation, Job Satisfaction, and Performance of Health Sciences Librarians Research

Danielle Aloia, Collection Management Librarian, New York Medical College, Valhalla, New York

Marie T. Ascher, Lillian Hetrick Huber Endowed Director, New York Medical College, Valhalla, New York

Deborah A. Crooke, Associate Director, New York Medical College, Health Sciences Library, Valhalla, New York

Objectives: This research explores the impact of faculty status in health science libraries by looking at factors that contribute to individual and institutional outcomes. It is hypothesized that librarians with faculty status are more likely to score higher in terms of esteem and motivation, satisfaction in their work, and as a result, high performance and job effectiveness which benefits the institution.
Methods: An online survey will be distributed via both the Association of Academic Health Sciences Libraries listserv, to which most academic health science library directors subscribe, with instructions to forward to their staff and to the Medical Library Association's Medlib-L listserv. The survey will collect demographics data, information regarding faculty status, and score respondents in the domains of esteem, satisfaction, and performance.
Results: 313 valid responses were received from librarians at 131 discrete institutions. 67% of respondents reported having some form of faculty status or faculty like appointment (FS) versus 39% with no faculty status (NFS). Preliminary results indicate that there is little difference between FS and NFS in terms of job satisfaction, esteem and self-reported performance level scores. 42% of FS and 36% NFS reported overall extreme satisfaction with their jobs. FS are more likely to participate in continuing education, research, publishing, and teaching activities, whereas the impact of faculty status is negligible on university committees, engagement with students, and grant-writing and fundraising activities. The majority of FS have never received a promotion in faculty rank at their current institution and only 11.7% have tenure.
Conclusions: There was a good response to this survey indicating a lot of interest in the topic and produced a lot of data to be mined. The analysis will be useful for institutions seeking to offer faculty status to librarians or to change their faculty evaluation criteria.

Burnout among Health Information Professionals: Elevating the Issue to Inspire Change Research

Angela Spencer, AHIP, Manager, Medical Library, St. Lukes Hospital, Chesterfield, Missouri

Elizabeth Laera, AHIP, Medical Librarian, Brookwood Baptist Health, Birmingham, Alabama

To obtain data on how many health information professionals meet the Copenhagen Burnout Inventory criteria and how many feel they are burned out or close to burning out despite survey results. This study also seeks to determine which category of health information professionals are facing the highest risk of burnout and what is being done to combat or prevent burnout.

METHODS: Potential study participants were contacted via various medical and special library listservs. Participation was limited to health science information professionals, including paraprofessionals. Using SurveyMonkey, study participants first completed a modified version of the Copenhagen Burnout Inventory (CBI) and then were asked about their general experiences with burnout, what, if anything, they are doing to relieve stress or prevent burnout, and demographic questions to quantify their results with others in similar settings, age ranges, and experience levels.
RESULTS: 497 responses were recorded. Of these, 41 respondents did not complete enough of the survey to be counted, bringing the total number of usable surveys to 456. 179 participants reported an overall level of burnout. Using average CBI scores, we determined that 117 of those reporting burnout had scores suggested at least a moderate level of burnout. 41.88% of these scored at the significant level of burnout or higher. 5 participants or 1.14% reported not feeling burned out scored at the moderate or significant level in the CBI. Of those scoring at least moderate in the CBI (n=143), 52.45% were from an academic environment and 62.22% had 15 or less years of experience. The participants with the highest CBI averages (n=26) had very little in common among their reasons for burnout or demographics.
CONCLUSIONS: Are health information professionals burned out? Research shows that burnout has been a theme in librarianship literature since the early 1980s. Public, academic, school, and special library journals have published articles on recognizing signs of burnout, preventing burnout in the workplace, and ways to combat burnout in librarians and information professionals. However, there is limited research to determine the number of librarians and information professionals experiencing burnout. This study helps to determine the level of burnout health information professionals are experiencing, and in the future, may help librarians and other information professionals find ways to prevent burnout.

The Evolving Role of Clinical Librarianship in a Value-Based Health Care Environment: A Qualitative Assessment Research

Elaine Attridge, Quality & Performance Improvement Librarian, The Claude Moore Health Sciences Library, Charlottesville, Virginia

Bart Ragon, Associate Director, Univ. of Virginia, Charlottesville, Virginia

Andrea H. Denton, Research & Data Services Manager, University of Virginia Health System, Charlottesville, Virginia

Objectives: Changes in healthcare reimbursement have created monetary incentives, enacted through the Affordable Care Act and CMS to encourage institutions to improve quality and possibly reduce costs. We investigated health care professionals' quality improvement activities, and perceptions of their information needs to meet institutional initiatives with the aim of developing a new library service model.
Methods: A qualitative study using semi-structured interviews was conducted on a voluntary sample of seven health professionals who were knowledgeable of library services and the health system environment as a whole. Open-ended interview questions in regard to information needs were used to explore ideas fully. The interviews were recorded and transcribed. De-identified transcripts were analyzed using the thematic analysis method. Three investigators read each transcript separately to

preliminarily code and identify possible codes. Codes were reviewed and consolidated if overlap existed. Coding differences were discussed until consensus was reached, and a standardized code list was developed. The researcher grouped the codes into themes and sub-themes to produce a qualitative report.
Results: All respondents were involved with quality and safety initiatives at varying levels within the University of Virginia Health System. Librarians can help meet the demand for quality-related information by searching the published and grey literature, by making connections to institutional resources and services, by providing data support and professional development learning opportunities, and finally, by sharing knowledge of new technologies.
Conclusions: Changes in the healthcare environment have greatly increased the demand for all institutions to engage in quality improvement efforts. The health sciences librarian can play a valuable role in meeting the unique information needs created by these initiatives within their organization.

Investigating Emerging Roles for Medical Librarians at College and University Libraries Research

Alexander J. Carroll, AHIP, Lead Librarian for Research Engagement, NC State University, Raleigh, North Carolina

Jason B. Reed, Health Sciences Information Specialist, Purdue University, West Lafayette, Indiana

Objectives: This study seeks to identify emerging roles for health sciences librarians by examining position descriptions at U.S. college and university libraries. We hypothesize that because of increasing interdisciplinary research within colleges and universities, there may be emerging roles for medical librarians at academic institutions without dedicated academic health sciences libraries as signified by AAHSL membership.
Methods: To gather these position descriptions, we are using two methods. We are collecting position advertisements posted to several job boards and health sciences librarian mailing lists between September 1 2018 and March 1 2019. Positions that include at least one of the following term in the position title, qualifications, or responsibilities are flagged for review: medic*, health, life, nursing, veterinary, bio*, pharma*. We are also advertising this study on several health sciences librarian mailing lists, soliciting individuals who meet these criteria to deposit their position descriptions into a repository via a Qualtrics survey. We are using grounded theory (an inductive, qualitative research methodology) to identify themes within the collected position descriptions that meet the inclusion criteria.
Results: We found a total of 104 job postings that met our inclusion criteria. Of those 104, 60 were advertised at AAHSL member institutions, and 44 from non-AAHSL institutions. 81 of these advertisements were for front-line librarian positions, with the remaining 23 for management level positions. Job postings were listed in 32 distinct states, with at least one posting in all 8 National Network of Libraries of Medicine (NNLM) Regions. We found the highest number of postings in Region 2 (Southeastern / Atlantic Region), and the fewest in Region 6 (Pacific Northwest Region). Qualitative and additional quantitative analyses of the content of the collected position descriptions are ongoing at this time.
Conclusions: Our preliminary quantitative data suggest the existence of a relatively high number of health sciences librarian job postings at institutions outside of AAHSL. Librarians entering into these advertised positions, specifically into front-line positions, will likely seek out professional development opportunities that will align with the MLA Competencies for Lifelong Learning and Professional Success.

This suggests an opportunity for MLA to expand its membership by designing continuing education opportunities for health sciences librarians employed at academic institutions without dedicated academic health sciences libraries.

Monday, May 6, 4:30 p.m.–5:55 p.m.
Room: Grand Ballroom A (East Tower, Ballroom/Gold Level)

Clinical Support Immersion Session 3

Moderator: Terri Ottosen, AHIP

Elevating by Evaluating: Recognizing and Avoiding Implicit Bias in Consumer Health Information

Terri Ottosen, AHIP, Community Engagement and Health Literacy Librarian, University of North Carolina – Chapel Hill, Chapel Hill, North Carolina

Karen Stanley Grigg, Health Sciences Librarian for Collections and Instruction, UNC Chapel Hill Health Sciences Library, Chapel Hill, North Carolina

Terri Ottosen, AHIP, Community Engagement and Health Literacy Librarian, University of North Carolina – Chapel Hill, Chapel Hill, North CarolinaIn fall of 2018, a flagship state university found itself in the midst of a controversy when a textbook for a required fitness class was criticized for calling cancer a "disease of choice", describing a theory that many Holocaust victims "failed to tap into their inner strength", and appeared to blame people for their poor health. Sadly, this textbook is not an isolated example of potentially offensive and biased consumer health information. There are many textbooks, patient handouts, and other publications on health sciences topics that use language that may be offensive or even discriminatory in terms of race, religion, sexual preference, gender identity, ability, weight, or socioeconomic status. This session will explore the issue through lecture, a fishbowl discussion, and a panel of Librarians discussing their perspectives. We will also provide a Consumer Health Bias Rubric we developed to assist in the evaluation of materials, which will be used to engage participants with a hands-on activity.

Monday, May 6, 4:30 p.m.–5:55 p.m.
Room: Columbus AB (East Tower, Ballroom/Gold Level)

Education Session 4

Moderator: Jennifer E. Dinalo

Reinventing the College of Veterinary Medicine Curriculum

Andrea C. Kepsel, AHIP, Health Sciences Educational Technology Librarian, Michigan State University, East Lansing, Michigan

Background: In 2016 the Michigan State University College of Veterinary Medicine was charged with reimagining their Doctor of Veterinary Medicine (DVM) curriculum and turning it into a cost-effective, learner-centered, integrated curriculum that produces high-value, career-ready graduates. The veterinary medicine librarian has been involved since the beginning of the process, with increasing responsibilities that ultimately led to being embedded in the instruction team when the new curriculum launched in the fall of 2018. This session will describe the process of developing a new curriculum and how the librarian leveraged their expertise to help shape the education of future veterinarians.
Description: After being identified as a College of Veterinary Medicine stakeholder the veterinary medicine librarian was invited to participate in a day-long workshop to identify key competencies for the new DVM curriculum. As the reimagining process progressed the librarian reached out to the college working groups and instructional design teams to suggest ways to integrate library resources and services into the new curriculum. This led to the librarian receiving an invitation to join the instruction team for the first course in the new curriculum, helping to create the course using backward design principles and developing course objectives and instructional materials on the topics of evidence-based veterinary medicine and information literacy.
Conclusion: Increasing involvement in the redesign process allowed the veterinary medicine librarian to develop a deep working knowledge of the DVM curriculum and identify key points where library services and librarian expertise is necessary. As part of the instruction team the librarian established close working relationships with members of the faculty and a well-known presence among the students. Evidence-based medicine and information literacy are integral competencies introduced early in the students' education and provide a foundation for the librarian to advocate for their inclusion throughout the four years of the curriculum.

Improvements to Assessment of Library-Led Doctoral Class Lead to Insights into Library Programming

Fred Willie Zametkin LaPolla, Research and Data Librarian, NYU Langone Health, New York, New York

Kevin Read, Lead, Data Discovery and Data Services Librarian, NYU Health Sciences Library, New York, New York

Nicole Contaxis, Data Catalog Coordinator, NYU Health Sciences Library, New York, New York

Alisa Surkis, Assistant Director, Research Data and Metrics/Vice Chair for Research, NYU Health Sciences Library, New York, New York

Background: Our health sciences library offers a required one-credit, research fundamentals class for first year PhD students. The class covers literature search, citation, data management, publication metrics, visualization, team science and data sharing. Because we are in the unique position of having a captive audience, we are able to conduct assessment deeper than typically possible in library workshops, presenting an opportunity to improve our educational offerings. This paper provides a case study exploring the history and evolution of assessment of a librarian-taught graduate level class and how we have come to improve over time to include both baseline and summative assessments.
Description: During the first two years of our class, we offered a final summative assessment that asked questions addressing topics covered in each of the 8 sessions of our class, as well as a questionnaire to understand attitudes about the class. This year in attempting to get a stronger sense of what was known by students coming into the workshop, we implemented a baseline questionnaire that offers multiple-choice and free text questions to assess what students come in knowing about course topics. The assessment was created in REDCap and administered on the first day of class after reading the syllabus. The experience of seeing what students know has led to improvements in our teaching to address these gaps within the class, as well as adjustments to general library workshops on similar topics.
Conclusion: The new baseline assessment already highlighted gaps in student knowledge entering the class, particularly in literature databases, data sharing, team science, and publication metrics, and these have informed how we have tailored the sessions. Moreover, data gleaned from formative and summative assessments highlight gaps in our teaching, allowing for improvements both in the context of the PhD class and general library workshops for the broader academic community. We expect that when the class is complete, analyzing improvements will highlight areas for improvement in our instruction as well as underscore difficult concepts for students.

Elevating Nursing Librarianship: Assessing Trends in Distance Librarian Services for Advanced Practice Nursing Programs Research

Gregg A. Stevens, AHIP, Health Sciences Librarian, Stony Brook University, Stony Brook, New York

Elizabeth G. Hinton, AHIP, Instruction and Research Librarian, University of Mississippi Medical Center, Jackson, Mississippi

Roy Eugene Brown, AHIP, Research and Education Librarian, Virginia Commonwealth University, North Chesterfield, Virginia

Objectives: With the increasing popularity of distance education among universities and busy students, many Advanced Practice Nursing (APN) programs have shifted to become either online or hybrid programs. To meet the research and instruction needs of these students, some nursing librarians are using technology for virtual research and instruction.
Methods: This study was designed to assess the extent to which nursing librarians in North America are providing virtual research and instruction services for APN students. An IRB-approved survey with ten

objective questions was developed to determine how librarians are providing services for APN students at their universities. It was announced in October 2017 through several health sciences librarian listservs. The survey ran for four weeks. Data were analyzed using Qualtrics and Excel.
Results: Eighty complete responses were received. The majority of respondents (66%) indicated that their universities' APN programs were conducted in a hybrid format and the same percentage also indicated that they generally provide library instruction in person. Most librarians indicated that they have provided research assistance through some virtual method (phone or email) and some have also used online chat (11%) and video chat (9%). A strong majority of librarians (95%) indicated that they feel comfortable using technology to provide research assistance and instruction.
Conclusions: Many opportunities exist for nursing librarians to use technology to provide virtual research assistance and library instruction. Greater promotion of these alternate methods can supplement traditional in-person services, providing greater flexibility for the busy schedules of graduate nursing students. Some outreach may be necessary to highlight the advantages of virtual services.

Embedded Librarianship in a First-Year Experience Program for Future Health Professionals

Brandon Patterson, Technology Engagement Librarian, Eccles Health Sciences Library, Salt Lake City, Utah

Tisha Mentnech, Research Librarian for Life Sciences & Research Metrics, North Carolina State University Libraries, Salt Lake City, Utah

Nena Schvaneveldt, AHIP, Education Librarian, University of Utah, Salt Lake City, Utah

Donna Baluchi, Library Supervisor, University of Utah, Salt Lake City, Utah

Background: Teaching librarians often refer to embedded librarianship as the confluence where inspiration, collaboration, and adventure happen. Embedded librarianship is rewarding and a pinnacle of library teaching and learning (Kemp, 2006). It is also complex, challenging, and requires continuous assessment. This panel presentation will highlight the experiences of four teaching librarians, with expertise in a range of topics, embedded in a state flagship institution's first year experience program for future health professionals.
Kemp, J. (2006). Isn't being a librarian enough? Librarians as classroom teachers. College & Undergraduate Libraries, 13 (3), 3-23.
Description: Four librarians will present perspectives on the challenges and successes of embedded library teaching in undergraduate first-year health professional courses. We'll present individualized ways librarians work with faculty partners to align library information literacy learning outcomes to the program's course outcomes and the ACRL framework. Throughout the discussion, we'll share a set of guidelines, library syllabi, and a checklist we used for designing, building, teaching, and assessing library instruction in collaboration with faculty partners. The program is comprised of two-semester cohort learning community courses grounded in health sciences. The primary partnership in each learning community are a lead instructor, a fully collaborative librarian partner, and a peer mentor selected from the top students of the previous academic year.

Conclusion: One highlight of the discussion will be results from a recently administered survey for all students in the program to assess their skills in information literacy. During the 2017-2018 school year, funding was secured for a program-wide assessment using Standardized Assessment of Information Literacy Skills (SAILS) test. The SAILS Test confirms the benefits of having an embedded librarian and compares students in the program with other institutional and national information literacy initiatives. Librarians will speak to the role assessment has played in validating the librarian role and how we've refined how information literacy content is delivered and infiltrated throughout coursework.

Building Library Capacity with a Newly Accredited Graduate Medical Education Program

Janet Lindsay Hobbs, AHIP, Manager, Library Services, Ventura Community Memorial Health System, Ventura, California

Background: Building library capacity at newly accredited libraries can be challenging as there are numerous physical, financial and cultural challenges. Establishing budgets, and academic priorities are critical components of enhancing a medical and research library. Designing library services to support newly accredited programs are daunting yet achievable. This abstract focuses on preliminary steps required to quickly organize and deliver effective library collections and services at a recently ACGME accredited teaching hospital located in Southern California.
Description: The process of developing library services to graduate medical education faculty, staff and students starts with the constuctivist adult learning theory. This theory relies on the assumption that adults are motivated to learn based on personal intrinsic factors. Each learner is seeking to apply learned knowledge to experiences. Adult learners enrolled in medical training programs are seeking evidence based resources that affirm their knowledge and experiences as Medical students.
Medical Libraries in academic medical centers play an important role with epistemology which involves the nature of learning. This abstract will focus on how to transition a Medical Library in a Community Hospital Library to an active Research Library that supports the academic needs of over 80 medical residents.
The constructivist learning theory provides the framework from which to base effective library services.
Conclusion: At the end of the presentation, attendees will:
be able to identify elements of the Constructivist Adult Learning Theory
be able to apply Constructivist Adult Learning theory to academic medical libraries
be able to prioritize core elements that enable effective library support to newly established programs

Monday, May 6, 4:30 p.m.–5:55 p.m.
Room: Columbus CD (East Tower, Ballroom/Gold Level)

Global Health & Health Equity Session 3

Moderator: Jessica Kilham, AHIP

It Takes All of Us: Engaging the Community in Biomedical Research and Precision Medicine

Peter C. Shipman, Dental Medicine and Cancer Librarian, Augusta University, Augusta, Georgia

Gail Kouame, Assistant Director for Research & Education Services, Augusta University, Augusta, Georgia

Background: Through a successfully funded outreach award, a health sciences library expanded upon traditional consumer health information outreach to include the basics of biomedical research and precision medicine. The goal of the project was to engage the public with increased knowledge of: quality health information resources, the research process and the benefits of participation in biomedical research. In addition the project focused on the concept of precision medicine, using the All of Us Research Program as an example of a precision medicine research study.
Description: Public libraries hosted consumer health information presentations by medical librarians. The presentations had three themes: becoming well-informed about conditions and medications using MedlinePlus; understanding the process and benefits of biomedical research studies; and recognizing the All of Us Research Program from NIH as an example of a precision medicine initiative that recruits partners (not subjects) from populations that do not traditionally participate in research. The importance of personal data and informed consent were secondary themes. The All of Us program aims to recruit a highly diverse population of one million or more partners. Presentation participants filled out a brief questionnaire following the event. Hosting organizations received iPads to facilitate ongoing access to health information for their audiences.
Conclusion: This project is still in process.

Elevating Public Librarians to Empower Patients

Elizabeth Irish, AHIP, Assistant Professor, Albany Medical College, Albany, New York

Kara Burke, Director for Community Engagement, Albany Medical College, Albany, New York

Ingrid Allard, Associate Dean for Community Outreach and Medical Education, Albany Medical College, Albany, New York

Enid Geyer, AHIP, Associate Dean for Information Resources and Technology, Schaffer Library of Health Sciences, Albany, New York

Background: After spending a year delivering community health information workshops at local sites, including public libraries, the project team identified a need for public librarian professional development. The team designed a curriculum with the goal of improving librarians' consumer health resources knowledge base and training them to assist patrons in utilizing effective communication methods with their healthcare providers. The curriculum was also developed with the objective of increasing the number of area librarians with MLA's Consumer Health Information Specialization (CHIS) designation.
Description: The curriculum incorporated three MLA 4-credit CE courses approved for CHIS. Two existing courses were selected, but one course was developed by the project team. This new CE course incorporates health literacy concepts, patient-physician interaction strategies, health information resources, and how they can be combined to improve healthcare communication and empower patients. Funding was received to support the purchase of twenty-five iPads for the public libraries to deliver health information in a confidential setting, MLA CE fees, and CHIS designation for participating librarians.
Twenty-three librarians from six public libraries, representing urban, suburban, and rural communities, completed the curriculum and twenty of these achieved CHIS designations. Evaluation consisted of the required MLA CE evaluations, a pre- and post- questionnaire for the newly developed course, and a three month follow up questionnaire sent to all participants. A one-year follow up is planned.
Conclusion: The evaluations indicated a change in behavior and knowledge. Librarians reported increased competence in subject content, comfort in their ability to help patrons prepare for their doctor's appointments, and knowledge of health information resources. For example, 32% were confident or very confident prior to the workshop that they could help patrons find quality health information. That number rose to 95% in the post-test. The three-month follow-up survey indicated that these results were sustained. All responding librarians indicated that the curriculum changed where they go for health information. The one-year follow-up will measure the same metrics as the three-month survey.

Motivated to Engage in Creating Healthier Communities: Evaluation of a Workshop for Librarians on Implicit Bias, Health Disparities, and Health Literacy

Michele Spatz, NNLM All of Us Community Engagement Coordinator, NNLM Pacific Northwest Region, Seattle, Washington

Catherine M. Burroughs, Associate Director, National Network of Libraries of Medicine, Pacific Northwest Region, Seattle, Washington

Background: NNLM PNR partnered with ALA ODLOS and PLA to present the workshop, "Implicit Bias, Health Disparities and Health Literacy: Intersections in Health Equity" for public librarians. We aimed to increase understanding of the role implicit bias plays in health disparities and health equity; help public librarians envision how health literacy can be utilized as a tool to improve health equity within their community and increase awareness of NNLM's support to improve health literacy through the All of Us

Community Engagement Network (CEN). The goal of the workshop was to inspire public librarians to engage in community health outreach.
Description: What is implicit bias? How is it connected to health disparities? And what's health literacy got to do with health equity? With the ALA Office for Diversity, Literacy and Outreach Services (ODLOS), the National Network of Libraries of Medicine – Pacific Northwest Region (NNLM-PNR planned a highly informative and interactive workshop that explored these timely themes through insightful presentations, self-reflection and group discussions. Since every library serves vulnerable citizens, participants learned how libraries can deepen their work in health literacy to ensure a lasting impact for improving the health of their community. An immediate post-conference evaluation was conducted to assess knowledge gained along with a two month post-conference follow-up survey to assess how participants applied new skills and resources acquired at the workshop to impact the health information needs of vulnerable populations within their community.
Conclusion: We will share evaluation and outcomes data that will show an increase in health literacy outreach, programs or services by our workshop participants.

Creating HOPE: A Directory of Local Health Resources for Community Engagement

Lorraine Sheldon, Community Engagement Librarian, Gibson D. Lewis Health Science Library, UNTHSC, Fort Worth, Texas

Brandy Klug, Web Services Librarian, UNTHSC, Fort Worth, Texas

Tom Lyons, Technical Services/Metadata Librarian, UNTHSC, Fort Worth, Texas

Background: The purpose of this paper is to describe the process of development and impact of a database designed to support the library's community engagement work. Overall, this project provides an example of how medical libraries and librarians can exercise their role as information experts to impact access to quality health information.
Description: The Health Organization's Program and Event (HOPE) Directory project is funded by the National Network of Libraries of Medicine (NNLM) to help centralize access to reliable and authenticated local information about programs and events related to health. The purpose of creating this tool is to democratize access and improve public health. As a resource library for the NNLM, the Gibson D. Lewis Health Science Library is better able to fulfill its role to serve the population of Tarrant County and in later phases greater geographic areas. The process of development includes the generation of a custom metadata scheme, website development, focus group evaluation, collaboration with community partners to collect data, and marketing.
Conclusion: The HOPE Directory is on-going and complete results will be gathered at the projects end date in May 2019. The success of the project will be measured by the launch of the website and mobile app, increasing the number of resources added, and the number of visits to the site.

Monday, May 6, 4:30 p.m.–5:55 p.m.
Room: Columbus EF (East Tower, Ballroom/Gold Level)

Information Services Session 4

Moderator: Amanda J. Wilson

Situating Systematic Reviews and Librarians at the Nexus of Teaching and Research

David A. Nolfi, AHIP, Head, Research Engagement, Health Sciences/STEM Initiatives & Assessment, Duquesne University, Gumberg Library, Pittsburgh, Pennsylvania

Maureen D. Sasso, Director, Information Services, Duquesne University, Pittsburgh

Background: Collaborating on systematic reviews, as well as other types of advanced reviews, situates librarians at the nexus of teaching and research. Mastering the steps in advanced reviews, including formulating clinical questions, developing comprehensive search strategies, evaluating study quality, and synthesizing results, offers rich opportunities for students to learn while participating in impactful research. This paper will present the case of a librarian-led collaboration to introduce advanced reviews campus-wide, not only to support faculty and clinician research but also to support student learning in varied formats including graduate seminars, dissertation preparation, and coursework in pharmacy, nursing, and other health professions.
Description: A librarian and nursing school administrator co-authored a successful grant to teach faculty to conduct systematic reviews. One goal was to increase use of systematic reviews in teaching graduate students. As a result, librarians partnered with faculty in health sciences and other programs to teach students how to conduct systematic and other advanced reviews. Librarians currently work with pharmacy graduate student seminars producing grant-funded systematic reviews, nursing PhD students using integrative reviews as part of a “manuscript alternative” to dissertations, educational psychology students conducting systematic review proposals for early dissertation research, and athletic training undergraduates producing and presenting critically appraised topics. The library is working with faculty to assess needs, and develop new workshops and tutorials to help students work through these processes. A roundtable for faculty using advanced reviews as a teaching method is planned to share best practices.
Conclusion: This program is ongoing. We plan to collect ideas from the faculty roundtable to develop programming to further library support for using advanced reviews in teaching. Recent library efforts have enabled faculty to make curricular changes to help students become better researchers earlier in their studies. Since these efforts began in late 2015, the university’s researchers published 39 systematic, integrative, or scoping reviews compared to eight in the previous 20 years. Graduate student researchers have had 19 manuscripts accepted or published by peer reviewed journals, another 12 manuscripts have been submitted to journals, and eleven reviews are currently in progress.

Search Strategy Protocol Compliance and Reproducibility in Nursing Systematic Reviews Research

Kerry Dhakal, Assistant Professor/Research and Education Librarian, The Ohio State University, Columbus, Ohio

Objectives: To find, review and analyze systematic reviews published in nursing journals via an in-depth literature review.
To determine if any of these reviews identified in the literature review follow protocol guidelines for reporting search strategies.
To rerun search strategies reported in nursing systematic reviews found in this study to ascertain if they are reproducible strategies.
Methods: Primary Research Question: Are nurse authors who are writing systematic reviews reporting search strategies as recommended by the PRISMA protocol or other systematic review guidelines? Methods: I am conducting an in-depth literature review of systematic reviews published in nursing journals, in four databases in the health sciences, including PubMed, CINAHL, Scopus and Embase.
Secondary Research Question: Are search strategies that have been documented reproducible? Methods: Post-literature review, I will analyze each of the systematic reviews found to determine if any of them include search strategy documentation in their methods sections, appendices or supplemental information. If I find that search strategy documentation has been reported, I will establish if the authors have reported their searches as suggested by the protocol guidelines they are following and if the search strategy is reproducible.
Results: I am currently conducting this study. My results will be available prior to the MLA 2019.
Conclusions: I am currently conducting this study. My conclusion will be available prior to the MLA 2019.

A Mixed-Methods Analysis of Authorship Requirements among Systematic Review Services Research

Shenita Peterson, Public Health Informationist, Woodruff Health Sciences Center Library, Decatur, Georgia

Hannah Rogers, Head of Information Services, Emory University, Atlanta, Georgia

Objectives: This study identifies the ways by which authorship is broached by librarians who participate in systematic reviews (SRs). Through close examination of the websites of the member institutions of the Association of Academic Health Sciences Libraries (AAHSL), libraries with a publicized systematic review service were identified. The institutions that describe authorship or acknowledgement were further examined and categorized.
Methods: The AAHSL member list includes 165 unique Health Sciences institutions, both foreign and domestic. Our research team thoroughly examined each member's websites for any mention of a systematic review service (SRS). Among those libraries that did mention a SRS, websites and accompanying linked forms were re-examined for any mention of their criteria for authorship and/or acknowledgement. These "mentions" were qualitatively coded using MAXQDA to draw out themes and tiers of service. Descriptive statistics were calculated with SPSS.

Results: One hundred sixty institutions were included in this analysis. While many libraries mentioned systematic reviews as a topic, 79 (49.4%) of the AAHSL libraries mentioned a SRS. Forty-eight (61.0%) of those libraries mentioned authorship. Co-authorship was merely suggested by 7 libraries (8.8%), while 26 libraries (33.0%) mentioned co-authorship directly. Placing a librarian on the author team was mentioned by 17 institutions (21.5%) and nine SRSs (11.4 %) required co-authorship for service. Additional themes that emerged were justifications for co-authorship and the monetary costs associated with librarian participation in SRs.
Conclusions: Although the International Committee of Medical Journal Editors (ICJME) sets criteria that define the role of authors or non-author contributors, librarians are frequently overlooked for authorship despite making significant contributions to systematic review projects. Libraries that are establishing or revising their systematic review services can use the information from this study to identify ways to advocate authorship for their SR team.

#BLESS: Starting a Systematic Review Service from the Ground Up

Nisha Mody, Health & Life Sciences Librarian, UCLA, Los Angeles, California

Bethany Myers, AHIP, Research Informationist, UCLA, Los Angeles, California

Antonia Osuna-Garcia, Health and Life Sciences Librarian, UCLA Biomedical Library, Los Angeles, California

Background: Librarians at a large research university receive requests for systematic reviews on an individual basis through informal means such as personal connections, e-mail, or referrals. A reference interview is conducted to obtain the type of review after which, librarians follow the appropriate guidelines for developing and documenting search strategies. In order to provide a streamlined service model and establish librarians as partners in the research process, we decided to create a formal submission process for literature searches.
Description: Librarians conducted an environmental scan of systematic review services in health sciences academic libraries. Requirements for our local service model were determined and methods of assessment were discussed. Based upon this information, we designed an intake form and service model workflow. Jira Service Desk (JSD) was chosen as the best project tracking tool to implement this service. The team collaborated with the IT department to implement and refine the intake form and request workflow within JSD. The service was named BLESS (Biomedical Library Expert Search Services). We QA tested the BLESS service desk site from the patron and librarian roles and reported issues back to the IT department. We plan to evaluate this service model using internal tracking and reporting features within JSD.
Conclusion: BLESS has not launched at the time of this submission, however, we plan to launch and market this service by the end of 2018. The follow outcomes will be measured in the future: number of requests received, user department, search type, number of completed searches, number of completed publications, and librarian work hours. The service model will be continually assessed and refined for any improvements.

The Impact of Covidence on Systematic Review Projects: Enabling Peak Performance for Researchers and Students Research

Jamie L. Conklin, Health Sciences Librarian / Liaison to the School of Nursing, University of North Carolina at Chapel Hill, Chapel Hill, North Carolina

Sarah Towner Wright, Clinical Librarian and Graduate Medical Education Specialist, UNC-Chapel Hill, Chapel Hill, North Carolina

Rebecca C. McCall, AHIP, Clinical Librarian, University of North Carolina-Chapel Hill, Chapel Hill, North Carolina

Nandita S. Mani, AHIP, Associate University Librarian & Director, Health Sciences Library, University of North Carolina, Chapel Hill, Chapel Hill, North Carolina

Elizabeth Moreton, Clinical Librarian, UNC Chapel Hill, Chapel Hill, North Carolina

Jennifer S. Walker, Cancer Information Librarian / Liaison to the School of Dentistry, University of North Carolina at Chapel Hill, Chapel Hill, North Carolina

Mary White, Global Public Health Librarian, UNC Chapel Hill, Chapel Hill, North Carolina

Objectives: Our library has licensed an institutional subscription to Covidence, a systematic review screening tool, to help researchers and students streamline their review processes. This study 1) gauged the impact of this tool on researchers' efficiency and collaborative efforts 2) determined whether the tool aligned with students' needs and 3) identified the most frequent methods for learning how to use Covidence.

Methods: Setting: An academic health sciences library meeting the systematic review needs of researchers and students within six health sciences schools, a healthcare system, several institutes, and other external partners.

Population: 1,116 faculty, staff, and student researchers who registered for a Covidence account from August 2017 through August 2018.

Study Design: Cross-sectional survey design with 28 close-ended questions focused on characteristics of participants' Covidence projects and review teams, perceptions of the tool in streamlining review processes, and training experiences, as well as one general open-ended question that allowed for additional comments.

Data Analysis: Researchers used SPSS to analyze data by groups—those who are researchers working toward publication and those who are students working on course assignments.

Research Ethics: This study was approved as exempt by the university's Institutional Review Board.

Results: A total of 184 researchers and students completed the survey, with an overall response rate of 16.5%. Researchers primarily used Covidence for systematic reviews and found the tool helped them in their individual work a great deal (67%) or a moderate amount (24%), as well as in their teams' ability to track progress, reduce email and meetings, and resolve conflicts (67% a great deal and 23% a moderate amount). Students used the tool most frequently for the following tasks: title/abstract screening (88%), full text screening (74%), adding/removing reviewers (70%), and importing citations (70%).

Conclusions: Overall, we found Covidence useful for researchers and students alike. Librarians can enhance the product's usefulness by informing researchers of its capabilities within a team environment and informing students of the steps entailed in creating reviews, making the most of the settings options, and screening at the title/abstract and full text stages. Librarians considering a systematic review tool should test the tool with their user groups' needs in mind and then evaluate the tool to ensure those needs are met.

Monday, May 6, 4:30 p.m.–5:55 p.m.
Room: Plaza Ballroom (East Tower, Lobby/Green Level)

Innovation & Research Practice Immersion Session 4

Moderator: Michelle Kraft, AHIP

Gerald (Jerry) Perry, AHIP, FMLA

Disorienting Dilemmas: Transforming the Librarian's Understanding of How Today's Health Professionals Discover and Use Information Resources Outside the Library Setting

Martin Wood, AHIP, Director, Florida State University, Tallahassee, Florida

Jeff D. Williams, AHIP, Chair, Medical Library and Director, Health Sciences Library, NYU Langone Medical Center, New York, New York

Jeff D. Williams, AHIP, Chair, Medical Library and Director, Health Sciences Library, NYU Langone Medical Center, New York, New York

Mary M. Langman, Medical Library Association, Chicago, Illinois

Michelle Kraft, AHIP, Director Medical Library, Cleveland Clinic Foundation, Cleveland, Ohio

Gerald (Jerry) Perry, AHIP, FMLA, Associate Dean, and Director, University of Arizona Health Sciences Library, University of Arizona Libraries, Tucson, Arizona

Librarians will engage in discussion
● about how library users find the information they need
● how this is similar or different from their existing perceptions
● what insight can be gained in challenging perceptions
● how can transformed perceptions result in librarians meeting their users needs more effectively

Innovation & Research Practice Session 5

Moderator: Nita K. Mailander, AHIP

Text Mining for Clinical Support

Jonathan Hartmann, Director of Clinical Integration Services & Data Discovery, Georgetown University Medical Center, Washington, District of Columbia

Linda Van Keuren, AHIP, Assistant Dean for Resources and Access Management, Georgetown University Medical Center, Dahlgren Memorial Library, Washington, District of Columbia

Background: Text mining is an artificial intelligence (AI) technology that enables fast retrieval of key content from text. Text mining identifies facts, relationships and assertions that would otherwise remain buried in large masses of text. In 2013 Dahlgren Memorial Library (DML) at the Georgetown University Medical Center began using text mining software in order to enable its clinical informationists to quickly retrieve specific, relevant information from MEDLINE abstracts while on patient rounds. In 2015 DML also began licensing large numbers of full text journal articles from selected publishers so that they could be downloaded and text mined as well.
Description: In 2013 DML licensed the use of a text mining program via the cloud, and the clinical informationist began using it to text mine MEDLINE abstracts on patient rounds. In 2015 DML moved to a local instance of the text mining software and negotiated with several publishers the right to download and text mine the full text of high impact journals in support of clinical decision support. DML also worked with its institution's information services department to utilize publisher APIs to facilitate article downloads onto the local server. A second informationist was also trained to use the software.
Results: The informationists found they were able to quickly find useful information not retrievable by traditional methods and clinicians reported the information was valuable.
Conclusion: The ability to text mine MEDLINE abstracts and selected journal articles on patient rounds has allowed DML's clinical informationists to quickly search large amounts of medical literature which can be used to answer physicians' clinical questions. DML plans to acquire additional journal articles from selected publishers in the future which should increase the usefulness of the project.

Quantifying Biomedical Data Reuse: Do Citations Tell the Whole Story? Research

Lisa Federer, AHIP, Data Science and Open Science Librarian, National Library of Medicine, North Bethesda, Maryland

Objectives: Many funders and journals now require researchers to share their final research data. Understanding how these data are reused could strengthen sharing policies, inform decision-making about curation, and facilitate development of metrics to reward sharing. However, tracking reuse remains challenging. This study explores the extent to which article citations to datasets accurately reflect their reuse.
Methods: This study measures the correlation between data reuse and citation, as well as characterizing types of reuse underlying data citations, by analyzing use requests for and citations to datasets from three biomedical repositories; two collect clinical data and one collects genomic data. Comparing use requests, which serve as a proxy for reuse, to citations, provides insight into how accurately data citations reflect reuse. Citing articles were analyzed to understand how datasets are reused, such as for original studies, meta-analyses, or methods validation, as well as how authors cited the dataset they had reused. Finally, semantic similarity was used to compare MeSH terms for the articles to the terms assigned to their corresponding datasets. This analysis provided a quantitative measure of whether data were being reused in similar contexts for which they had been collected or in novel topics.
Results: While use requests and citations for datasets in this study are correlated, the average dataset had only one citation for about every 8 requests. Articles citing data represented many types of reuse, with different patterns of reuse for clinical versus genomic data. While most articles reused datasets in a context similar to that for which the dataset was collected, 10% of the article/dataset pairs had a semantic similarity score of 0, meaning they were reused in a very different context. Citations themselves lacked consistency, with authors indicating they had reused datasets in a range of locations within the article.
Conclusions: The large disparity between citations and use requests suggests that citations do not adequately capture the extent or characteristics of data reuse. These results have implications for how data reuse is measured and evaluated, and therefore, how impact of datasets can be assessed to reward researchers who share their data. These findings could provide guidance to journals, funders, repositories, and researchers who share data about how to increase the visibility of reuse of datasets.

Building an Interprofessional Literature Review Service Using a Service Framework and Design Thinking

Nicole Capdarest-Arest, AHIP, Head, Blaisdell Medical Library, University of California, Davis, Sacramento, California

Megan G. Van Noord, Health Sciences Librarian, University of California, Davis, Davis, California

Erik Davis Fausak, Health Sciences Librarian, Carlson Health Sciences Library - UC Davis Library, Davis, California

Amy Christine Studer, AHIP, Health & Life Sciences Librarian, University of California, Davis, Sacramento, CA, California

Bruce T. Abbott, Librarian, University of California, Davis, Sacramento, California

Background: The library at University of California, Davis includes two health sciences libraries (the HSLs) – one serving graduate programs in human health sciences, and the other serving undergraduate

and graduate programs in veterinary medicine, basic biomedical sciences, and public health. Though separated by 20 miles, the HSLs wanted to leverage librarian expertise across the HSLs to develop a unified interprofessional literature review and bibliometric analysis service. This paper describes the process and framework for creation of this unified service.
Description: A project outline was developed for the planning of the service. Project phases included: project planning kick-off, user needs assessment, data gathering and analysis, building a service framework, collaborative design meetings, implementation, testing, and continual process improvement. Regular meetings and task assignments were set with defined goals for each project development phase. Given the wide array of expertise of our librarians and the interprofessional nature of our communities, special consideration was given to creating a service that would meet the various user needs (e.g., human- and animal-related topics, literature searching as well as bibliometric analysis service components). In order to facilitate continuous quality improvement of the service, user feedback/assessment was built in as a key part of the service model.
Conclusion: This service building project is still underway. We expect to discuss outcomes related to whether using a service framework and design thinking process is a useful model for creating services in an academic health sciences library setting, and preliminary feedback on this new service.

Biomedical Bhutan through Bibliometrics Research

Kate Nyhan, Research and education librarian, Yale University, New Haven, Connecticut

Objectives: Bhutanese researchers and international partners are publishing research about clinical medicine and health services in Bhutan, as a result of English-language medical education in Bhutan and abroad. Who is writing, funding, publishing, and citing this work? What domains are currently being addressed, and are there gaps in published research?
Methods: Through bibliometric analysis of published research (in the databases PubMed, Scopus, Web of Science, Global Health, Embase, and IMSEAR, and in the Bhutan Health Journal), a corpus of published biomedical literature about Bhutan will be created. Key stakeholders -- authors, institutions, funders, and journals -- will be identified, and co-authorship networks will be investigated. Subject coverage will be analyzed based on journal subjects and subject indexing of individual papers.
Results: I hypothesize that we will find an increasing volume of published papers about Bhutan, of published papers by Bhutanese authors, and of citations to papers in the Bhutan Health Journal. I hypothesize that open access papers are more likely to accrue citations by Bhutanese authors than paywalled papers are. I hypothesize that Drungtshos (doctors of traditional medicine) will be underrepresented as authors of English-language papers, compared with their presence in the Bhutanese medical education and health care delivery system. Finally, I hypothesize that emergency medicine and travel medicine will be among the subject areas well-represented in English-language Bhutanese medical literature.
Conclusions: The findings of this research project will be valuable to researchers, medical librarians, and policymakers at educational institutions, government agencies, and third sector agencies active in Bhutan. The findings will also assist researchers and medical librarians planning systematic reviews or other evidence syntheses about Bhutan, South Asia, or Tibetan traditional medicine.

Impact of Retracted Publications in Evidence-Based Dentistry
Research

Nicole Theis-Mahon, AHIP, Liaison to the School of Dentistry & Collections Coordinator, University of Minnesota, Minneapolis, Minnesota

Caitlin Bakker, AHIP, Research Services Liaison Librarian, University of Minnesota, Minneapolis, Minnesota

Objectives: Publications are retracted for many reasons, but the continued reading and citation of retracted publications may impact patient care and future research. This project analyzes retracted publications in the dentistry, including reason for retraction, citation patterns, and nature of citing items. The objective is to investigate which factors contribute to the continued influence of a retracted publication in evidence-based dentistry.
Methods: Retracted publications in the dental literature were identified through the RetractionWatch Databases's Dentistry category. Additional information including the study design, the reason for retraction, PMID number, and DOI were added to the dataset. Known item searching was conducted in Scopus and Web of Science to identify articles that have cited these retracted publications. The full-text of citing items were consulted and those published or submitted for publication prior to the publication of the retraction notice were removed, as were the retraction notices. Theoretical frameworks developed by Bar-Ilan and Halevi were applied to categorize the type of retraction and the negative, neutral, and positive nature of the subsequent citations of retracted publications. Statistical analysis was performed to determine which characteristics were significant predictors of continued citation following retraction.
Results: 136 retracted publications were identified, of which 108 had been cited by over 1,400 items. The majority of the 136 papers were retracted due to ethical misconduct (80/136, 59%), followed by scientific distortion (37/136, 27%). Excluding items that were published before retraction, over 700 citing items remained, which cited 85 retracted items. The majority of the cited retractions were either positive or neutral in the cited papers with fewer examples of the negative categorization. Study design of both the original article and citing items were also considered.
Conclusions: Retracted publications continue to have an impact in the dental literature post retraction. Advances need to be made to identify retracted publications throughout the submission, review, and revision of manuscripts to ensure that retracted publications do not have a positive impact in future dental literature.

Monday, May 6, 4:30 p.m.–5:55 p.m.
Room: Columbus KL (East Tower, Ballroom/Gold Level)

Lightning Talks 4

Moderator: Natasha Williams, AHIP

How Medical Students Discover Information Tools

Margaret A. Hoogland, AHIP, Clinical Medical Librarian, Mulford Health Sciences Library - The University of Toledo, Temperance, Michigan

Objective
Many studies discuss the use of medical information tools by clinical medical students (i.e. third-and-fourth year students). Few studies examine how preclinical students (i.e. first-and-second year students) discover and use medical information tools. By understanding the needs of preclinical medical students, medical librarians can adjust the content and delivery of information in their training sessions. The purpose of this study is to better understand the medical information needs of preclinical students.
Methods
Medical students received and email containing the study description, link to an online survey, and an opportunity to answer additional questions about medical information tools. Discussion participants got an Amazon or Starbucks gift card. Survey participants received no compensation.
Results
Of the 525 students who received an invitation to participate, 122 completed the survey and 18 participated in a discussion. Preclinical students primarily use UptoDate and Epocrates. By contrast, Clinical students use Google, Google Scholar, and UptoDate. During the discussion sessions, most students mention UptoDate first but mention using other medical information tools. Out of 18 students, two students or 11% consulted UptoDate exclusively in a clinical setting.
Conclusion
Students primarily discover medical information tools through conversations, during classes, or one-on-one sessions with faculty and health science librarians. Study results show even a short session with a librarian improves preclinical students' knowledge of available tools and services. Librarians, who adapt sessions and conversations with preclinical students, can impact how students use medical information tools for the remainder of medical school.
Keywords: Medical Information Tools, Medical Students, Information Discovery, and Information Use

Improving Core Entrustable Professional Activities (EPA) 7 Compliance through Librarian Feedback

Saori Wendy Herman, AHIP, Head of Education and Access Services, Donald and Barbara Zucker School of Medicine at Hofstra/Northwell, Hempstead, New York

Background: Fourth year students at the Zucker School of Medicine at Hofstra/Northwell rotate through three acting internship (AI) blocks. At the end of each AI, the students are required to present an evidence-based oral presentation based on a clinical encounter from their rotation. Along with the oral presentation, students are expected to create a PICO formatted clinical question, perform a literature search, and select a relevant citation(s). Starting in 2017, a librarian was integrated to provide formative feedback to students about their submissions. The purpose of the librarian's feedback was to improve the students' question formation and searching skills for fulfillment of Core Entrustable Professional Activities (EPA) 7.
Description: Previously, the students' PICO question, search, and citations were not submitted or assessed separately. Following the introduction of the librarian, students are now responsible for emailing their PICO formatted clinical question, PubMed search strategy, PubMed Search Details box information, a brief description of their search strategy creation process, and a relevant citation/abstract to the librarian. In collaboration with the assistant dean for clinical preparation for residency, the assessment analyst, and the librarian, an assessment form was created and uploaded onto one45, a curriculum management software. The assessment form contains 5 questions and a free text feedback box. The librarian reviews each submission and provides feedback using the form within 2 weeks. A complete list of assessment form responses can be exported from one45.
Conclusion: The integration of the librarian has provided a unique opportunity to provide feedback to fourth year students. Through the assessments, we were able to capture search habits and common mistakes as well as getting a sense of what information literacy topics need to be covered during the first two didactic years. The quality of submissions from the first to last rotations have remained fairly constant. This is partially due to the students' question formation and searching skills falling in the middle of the novice-to-expert continuum. Further research measuring students' confidence levels is currently underway.

The Force of Magnetism: Nursing-Library Collaborations to Support Magnet

Barbara (Basia) Delawska-Elliott, AHIP, Medical Librarian, Providence St. Joseph Health, Portland, Oregon

Helene Anderson, Director or Nursing and Patient Care Services, Providence St Vincent Medical Center, Portland, Oregon

Marla London, Manager, Clinical Performance Support, Providence St. Vincent Medical Center, Portland, Oregon

Background: The ANCC Magnet model is built on 5 Model Components and 14 Forces of Magnetism. The Components and Forces, including Transformational Leadership, Structural Empowerment, Exemplary Professional Practice, New Knowledge, Innovation & Improvement, and Empirical Quality Results (Models), and Professional Development, Quality Improvement, Nurses as Teachers, and Quality of Care (Forces) encourage and recognize professional growth, care improvement, and knowledge dissemination. Familiarity with searching professional literature and information literacy are key skills needed to grow Magnetism. This presentation will describe a collaboration between Magnet leadership and the library to build programs designed to support Magnet values and outcomes.

Description: Magnet leadership at a four-time Magnet-designated hospital approached the library with a proposal for an evidence-based program pilot. The librarian's role in the pilot was co-developing the instructional module introducing EBP, searching the literature and evidence appraisal, and hands-on small group search training with a PhD-prepared nurse. The program was launched in 2015 and post-program analysis showed improved understanding of the EBP process and higher information literacy confidence. Building on the success, the following year the interdisciplinary team consisting of hospital magnet leadership, nursing research leadership, and the librarian added a nursing research program. The ongoing educational programs were named best practice by ANCC Magnet appraisers and the program projects were cited in the Magnet documentation. Following appraiser evaluation, to address the need to disseminate knowledge externally, this year the hospital is launching a program helping nurses publish.
Conclusion: The program started as an instructional venture introducing the concepts of evidence-based practice and nursing research to hospital staff nurses. Since its inception, 6 projects have led to practice change, 3 have been presented at conferences, one is being written up for publication. Some projects evolved from evidence-based to research questions and from smaller to larger studies in different hospital settings. Many original program participants have continued with the program or have developed independent research studies. The success of this collaborative program lies not only in renewed Magnet designation, but also by nurse engagement in practice change and nursing research.
Keywords: ANCC Magnet, education, evidence-based practice, nursing research, nursing publications, nurse engagement, nursing care, interprofessional collaboration, nursing leadership, hospital libraries

Virtual Reality: Elevating Teaching and Learning

Kellie N. Kaneshiro, AHIP, Assistant Director for Library Technology, Indiana University, Indianapolis, Indiana

Jason A. Lilly, Library Systems Manager, Indiana University School of Medicine Ruth Lilly Medical Library, Indianapolis, Indiana

Chelsea Misquith, Emerging Technologies Librarian, Indiana University, Indianapolis, Indiana

Brandon Dennett, Manager of Medical Library Technology, Indiana University School of Medicine, Indianapolis, Indiana

Background: Virtual reality is already changing health care for patients and providers alike, whether it is viewing a surgical operation in VR (Medical Realities http://www.medicalrealities.com/), distracting patients from pain, helping future physicians gain empathy by stepping into the shoes of an elderly patient or a patient with Alzheimer's (https://embodiedlabs.com/) , or using a virtual anatomy atlas. Starting in 2017, the Medical Library has been offering VR experiences to faculty, staff, and students at the School of Medicine.
Description: Since 2017, a portable VR set-up has been purchased, enabling us to "take the show on the road" to regional campuses. Another potential use is to bring VR into a classroom setting. In the fall of 2018, four virtual reality stations were installed in the Library's collaborative learning space. The stations were subsidized by the library and in part by the University's Advanced Visualization Lab. The idea behind the stations is to lower the entry barrier to VR by making it available with a menu of pre-loaded applications. Students, staff and faculty can login using their University computing credentials. Those

more comfortable with the technology have instructions and list of applications to choose from, those wishing for a more guided experience may make an appointment. The Medical School's Simulation Center has purchased VR equipment and there are opportunities for collaboration.
Conclusion: We are collecting data on the number of unique users across all four VR stations, and evaluating application usage. We are also working on gaining feedback from current and potential users of the service, and other ways to lower barriers to access and develop collaborative partnerships.

Elevating the Understanding of Library Resources: Librarians Working with Clinical Departments' Administrative Assistants

Alexandria Leigh Brackett, AHIP, Clinical Librarian, Cushing/Whitney Medical Library, Yale University, New Haven, Connecticut

Caitlin Meyer, Research & Education Librarian, Yale University

Background: Two librarians at an academic medical library created a workshop series for administrative assistants in the cancer center to build capacity in different library-provided resources and tools to make aspects of their jobs more efficient and manageable. This project enabled the administrative assistants to expedite tedious processes and taught the librarians about a new patron population they could work with.
Description: In the fall of 2017, the library was approached by an administrative services supervisor in the cancer center to assist his new team's work responsibilities. Each team member served as an administrative assistant to at least one principal investigator (PI).
The librarians designed four workshops that would help team members with the research and professionalization needs of the PIs. Classes included: PubMed and the library catalog, Introduction to EndNote, Research Impact and Scopus, and Qualtrics. These classes taught the participants how to find articles by their PI efficiently, export formatted citations from EndNote for CVs and bibliographies, find and understand research impact information, and create surveys and forms.
Conclusion: The first run of the program was so successful that another group approached the librarians to teach the series again. The administrative assistants learned how the library can support their work and the librarians were able to work with and learn more about a new patron population. Since the workshops, the two librarians have worked with other administrative assistants throughout the academic medical center as individuals and as a group to show how to use library resources to help their PIs, including a class on PubMed and SciENcv.

Establishing the Open Access, Student-Faculty-Run Cooper Rowan Medical Journal: Lessons Learned and Next Steps

Amanda Adams, Reference & Instruction Librarian, Cooper Medical School of Rowan University, Camden, New Jersey

Benjamin Saracco, Reference and Research Librarian, Cooper Medical School of Rowan University, Camden, New Jersey

Background: In 2018 the Cooper Medical School of Rowan University (CMSRU) library was approached by a clinical faculty member and two medical students who expressed interest creating a legitimate, peer-reviewed journal to publish student research from around the world. The library took on the challenge of facilitating this project by creating all of the infrastructure necessary for students and faculty to then make the journal their own. The newly created Cooper Rowan Medical Journal (CRMJ) now has students embedded into the scholarly publishing and peer review process. The lightning talk will provide advice on starting peer-reviewed journals that are partially student-operated.
Description: The journal fills the niche of providing early-career scholars a home to highlight their scholarship, while learning the best practices of scholarly open access publishing. Technical details to cover are branding, website design, policy creation, training, and marketing. Students and faculty directed the journal's scope. The publishing platform uses the university's institutional repository (IR) BePress software. A key step, along with many others, was to provide instruction for students on how to conduct peer review. The librarians' roles as Managing Editors will be described, along with highlighting the current status of the journal project and next steps. There are many tips, best practices, as well as mistakes and warnings that may help health sciences librarians interested in carrying out similar publishing projects. CRMJ is now actively accepting manuscript submissions and students are working with faculty in the peer review process.
Conclusion: This project revealed unique challenges of working with multiple stakeholders and demonstrated that librarians can be excellent partners starting publishing projects. They ensure projects follow best practices such as: informing partners about Open Access issues, copyright, issuing of ISSNs/DOIs, and ethical publishing guidelines to increase the chances of the journal eventually being included in indices. Utilizing an IR can be a powerful educational tool and provide a great foundation to build on for future careers as scholars. Further, library professionals can take on the role of publisher using an IR's platform. Students will continue to gain valuable scholarly publishing experience.

Library Support of the Research Lifecycle

Bart Ragon, Associate Director, Univ. of Virginia, Charlottesville, Virginia

Objectives: In what ways do health sciences libraries support the research lifecycle?
Methods: Qualitative data was collected from biomedical researchers using a semistructured interview instrument. The instrument asked researchers to describe their workflow from idea inception to the dissemination of findings. A document analysis of research lifecycle models assisted in the creation of the interview instrument. Analysis of the interview data utilized open coding techniques, which allowed for the data to be explored and conceptual categories to emerge. Data was collected until saturation of codes occurred and then reviewed for consistency to increase reliability of code application throughout data analysis. In many cases, participants described aspects of their research that co-occurred with additional activities of the research process. Network analysis software was used to visualize the relationship between codes and illustrate the connection between library research support services and the practices of biomedical researchers.
Results: The network analysis visualization tool demonstrates that libraries were frequently mentioned as a resource supporting the research lifecycle. However, the visualization also illustrates that library support resides on the fringe of research activity. In addition, the clustering algorithm does not associate library support with the traditional library strengths of literature searching, systematic reviews, and citation management. Results from the analysis suggest that library resources and services are important to research, but not integrated within other research practices.

Conclusions: This study sought to uncover how health sciences libraries support the research lifecycle. Qualitative interviews with researchers provided rich information on the practices of biomedical workflows. Analysis of the co-occurrence of codes from interview data analysis illustrate how library resources and services connect with other aspects of the research lifecycle. Findings from this study suggest that many traditional library services are not integrated with other workflows in biomedical research. Additional research on the needs of research workflows might increase libraries' understanding of their role in biomedical research.

When the National Institutes of Health (NIH) Warn Researchers about Spies: Implications for Open Science

Melissa L. Rethlefsen, AHIP, Associate Dean and Fackler Director, University of Florida, Gainesville, Florida

Tisha Mentnech, Research Librarian for Life Sciences & Research Metrics, North Carolina State University Libraries, Salt Lake City, Utah

Shirley Zhao, Data Science Librarian, The University of Utah, Salt Lake City, Utah

Background: On August 20, 2018, NIH Director Francis Collins distributed a letter to grant recipient institutions describing newfound discoveries about foreign entities' "systematic programs to influence NIH researchers and peer reviewers." The letter goes on to describe concerns about diversion of intellectual property, sharing of confidential grant information, and failure of some researchers to disclose funding and resources from foreign governments. The NIH Working Group convened to examine these concerns made numerous recommendations for the NIH to consider to mitigate these issues, while preserving relationships with foreign nationals.
Description: As the NIH works to resolve these issues, it must balance its policies favoring openness and sharing with research integrity. Librarians need to be aware of these issues to best help our colleagues share their data appropriately. We will briefly review the NIH's recent concerns, export control laws, and data sharing policies in context of librarians' roles in data management, data sharing, and open science. We will discuss implications of NIH warnings for librarians working with data management, data sharing, and open science.
Conclusion: Combined with existing export control laws, concerns about foreign influences on research may cause researchers to think twice about openly sharing data. Librarians working with data must be familiar with both data sharing and data restrictions to most effectively work with researchers. Librarians can take a proactive stance working with their institutions to both raise awareness of the concerns raised by the NIH as well as help our fellow researchers navigate what data can and should be openly shared.

The Data CAVE: Developing a Multifunctional Space for Research Data Services and Instruction

Peace Ossom Williamson, AHIP, Director of Research Data Services, The University of Texas at Arlington, Arlington, Texas

Background: In two years, an academic library worked to develop research data services focused on the provision of expertise around data analysis and visualization as well as data management support. Infrastructure that arose around these services includes a popular workshop series offered each semester, an institutional data repository, data management guidelines, data portal with information on all available resources and services created by a team of staff from across the larger institution. To expand these services, an effort began toward the creation of a physical space that had multiple functions – instruction, consultations, and open lab for software and hardware access.
Description: This lightning talk will give a quick look into the implementation and use of the Center for Data Creation, Analysis, Visualization, and Exploration – or the Data CAVE. The Data CAVE provides multi-use furniture and hardware, with rolling tables, chairs, and screens allowing. It is also outfitted with computers with a special image with software for data exploration and reporting as well as varying processing power, all of which are higher than those offered for general use on campus. The Data CAVE has hours in which staff provide support for users learning new techniques or new programs. The presenter will give information about what is present in the space, tips about how to plan a space for libraries of varying sizes and budgets, as well as successes and lessons learned.
Conclusion: At the time the abstract was written, the Data CAVE had recently opened, and we planned data science workshops, hands-on sessions around tools (programming languages and ArcGIS), and a hackathon for Open Data Day to test the functionality of the space, including the physical arrangement and available hardware and software. The presenter will provide the outcomes of these events and how well they worked in the space as well as barriers that were faced, obstacles that were overcome, and conclusions that come from these events.

"Sieving" Your Search: Semi-Automated Citation Screening for Search Strategy Refinement

Edwin Vincent Sperr Jr., AHIP, Clincial Information Librarian, AU/UGA Medical Partnership, Athens, Georgia

Background: Those who develop search hedges and systematic review strategies often use a process where they measure how effective a given search is at retrieving items from a set of "gold standard" articles. While this is a robust method, it is also resource-intensive. There are many occasions when a searcher needs to refine a search strategy in an iterative way, but she does not have time to manually assemble a set of exemplar citations. This describes an application that would allow a searcher to test different iterations of a search strategy against a set of "known-good" citations in a streamlined way.
Description: This application, currently under development, will allow a user to test and refine searches against PubMed, which is both eminently suited to biomedical searchers and possessed of a robust API for developers. The task flow will begin with a user's initial search, after which she will be presented with a limited, randomly chosen set of search results. She will then examine the citation (and abstract, if available) of each result and determine whether it is relevant to her search task or not.
Citations marked as relevant will be stored as "known-good" (or at least "likely-good") items for that search, while those that are obviously irrelevant can be deemed "known-bad". From there, successive search strategies can be automatically tested in the application as to how well they retrieve the desired citations while also avoiding the bad ones.
Conclusion: By having a way to easily quantify the performance of different strategies, it is possible that this application could improve the rigor of "everyday" searches for complex topics. User testing will be necessary to refine this concept. In particular, the author would wish to determine the minimum

number of items needed for examination in the initial stage in order to provide reliable information as to how well successive searches perform.

Elevating Access: Transitioning from OCLC Discovery to EDS Health

Tiffney A. Gipson-Goodwin, Head of Collections & Development, University of Louisville, Louisville, Kentucky

Lauren E. Robinson, Medicine Liaison Librarian, University of Kentucky, Lexington, Kentucky

Background: Discovery services are increasingly popular within the library community because of the users' ability to conduct simply searches and receive unified results. Five years ago, the University of Louisville Libraries converted from Voyager to OCLC Discovery. In that timeframe, Kornhauser Health Sciences Library witnessed a rise in user dissatisfaction, in addition to an increase in time spent by librarians solving access issues.
Description: Since the transition from Voyager to OCLC Discovery, Kornhauser Library recognized that the OCLC product did not meet the needs of health sciences users. The lack of medical terminology and taxonomies provided a unique challenge for both users and librarians. As a result of the challenges presented by OCLC Discovery, the Head of Collections and the Emerging Technologies Librarian researched two health specific Discovery Services: OVID's Discovery and EBSCO Discovery Service (EDS) Health. The two products were presented to library faculty, and the decision was made to move forward with implementing a trial of EDS Health. In this presentation, the challenges of attracting buy-in, funding, and implementation will be discussed.
Conclusion: In order to evaluate EDS Health, the Head of Collections and Emerging Technologies Librarian formed a team consisting of representatives from all departments within Kornhauser Library. This team will evaluate searching efficiency, report any access issues, and trial the product with test users. The ultimate goal is to have this preliminary testing completed by the end of Spring 2019 and a full launch by orientation season.

Managing Expectations: Lessons Learned from My First Scoping Review

Kelly Johnson, Veterinary Outreach and Information Resources Librarian, Cornell University Flower-Sprecher Veterinary Library, Freeville, New York

Background: Scoping reviews, like systematic reviews, are increasingly popular across multiple disciplines and research fields. A librarian's unique skill set positions them to add significant value to a research team, but many librarians lack the experience to fully manage researcher expectations. Recognizing and planning for potential pain points along the evidence synthesis timeline allows a librarian to protect the bounds of their participation while maintaining positive team dynamics. This presentation aims to introduce librarians to some of the smaller unanticipated issues associated with working on a SR team, and ways to avoid them.
Description: As a new systematic review service team member, I joined a research group in conceptualizing and executing my first scoping review. Good training prepared me to navigate the

project successfully, but inexperience precluded anticipation of some smaller complications that slowed progress and negatively affected team dynamics. Here I present some of the less obvious frustrations a librarian may experience as they first participate in systematic or scoping reviews, and suggest ways to avoid them.
Conclusion: Along with advice from others, lessons learned from participating on my first scoping review have prepared me to better communicate the role (and the limits of that role) I expect to play on a research team. For example, I now explicitly address expectations related to team meetings, full-text retrieval, manuscript preparation, and author lines at the beginning of the review.

An Exercise in Diversity: Providing Resources to Disabled Faculty and Staff at a Large University

Anna Ercoli Schnitzer, Informationist, Taubman Health Sciences Library, ANN ARBOR, Michigan

Background: Although our large academic institution has been well equipped with resources and services for its students with disabilities through its Office of Student Services, there has never been a centralized process to assist faculty and staff members with disabilities who need to know about available disability and accessibility resources on campus and where to find special services. This information gap adversely impacts both recruiting and maintaining employment of faculty and staff with disabilities. A cross-campus committee, the Council for Disability Concerns, chaired by a librarian and including a number of librarians, among other colleagues, decided to address this problem.
Description: The Council for Disability Concerns applied for and received a grant of $7,000 from a campus initiative called Diversity, Equity and Inclusion (DEI). These funds were allocated for constructing a website portal to which group members would contribute a wide variety of disability resources and services. The initiative “Success at Work” was created as a PowerPoint slide to outline the initial stage of the project. Such topics as accessibility aids, transportation assistance, and disability affinity groups will be included. The disability resources will be evaluated and updated on a continuous basis. The website, when completed, will be part of the Office of Institutional Equity, under the supervision of the campus director of the Americans with Disability Act. The second phase of the project, provided it can be adequately funded, will include a physical space for disabled individuals to meet.
Conclusion: Since the program "Success at Work" is still a work in progress in its early stages, our plans are to continue to populate the online portal. When a critical mass of relevant resources has been compiled, we expect to evaluate the website resources by means of a survey to our mailing list of over 330 Council members. Subsequently, we will consult with another university unit specifically charged with recruiting diverse faculty members, to measure the outcomes of their recruitment of individuals with disabilities and discover whether the "Success at Work" resources portal has been an effective tool for this purpose.

Monday, May 6, 4:30 p.m.–5:55 p.m.
Room: Grand Ballroom B (East Tower, Ballroom/Gold Level)

Professionalism & Leadership Immersion Session 6

Moderator: Ariel Deardorff

Find the Right Planning Framework for Your Project

Ariel Deardorff, Data Services Librarian, UCSF Library, San Francisco, California

Keith W. Cogdill, AHIP, Director, NIH Library, Bethesda, Maryland

Mary Beth McAteer, Librarian, Virginia Mason Medical Center, Seattle, Washington

Ariel Deardorff, Data Services Librarian, UCSF Library, San Francisco, California

Ariel Deardorff, Data Services Librarian, UCSF Library, San Francisco, California

Planning frameworks can be a useful tool for libraries struggling to managing competing priorities and projects, but some models may be better suited to specific environments than others. During this session participants will learn about three planning frameworks (Objectives and Key Results, Lean, and Balanced Scorecard) and hear from 3 speakers who will share their experiences implementing planning frameworks for routine goal setting as well as strategic planning.

Tuesday, May 7, 2:00 p.m.–3:25 p.m.
Room: Columbus GH (East Tower, Ballroom/Gold Level)

Education Session 5

Moderator: Timothy Roberts

Creating and Implementing an Integrated Information Literacy Roadmap for a Nurse Practitioner Program

Rachel Charlotte Lerner, AHIP, Public Services Librarian, Quinnipiac University, Hamden, Connecticut

Laima Karosas, Chair, Graduate Nursing Programs, Quinnipiac University, Hamden, Connecticut

Susan D'Agostino

Background: In 2010, the Institute of Medicine (IOM) published the "Future of Nursing Report." The IOM posed that nurses need to view themselves as change-agents and better represent themselves as leaders. One pathway to the boardroom is becoming skilled and responsible discoverers, consumers, creators, and disseminators of information. A partnership between a School of Nursing (SON) and a nursing librarian led to the creation and implementation of a stepwise information literacy (IL) curriculum integrated throughout the Doctor of Nursing Practice (DNP) courses and coursework, delivered to all DNP students, both online and on-ground.
Description: Three theoretical frameworks (including the ACRL IL Framework) guide the interactions; each is centered on at least one threshold concept. Ten major topics were identified and condensed into content for nine separate courses with multiple sections. Both formative and summative assessment is built into many of the interactions, and is subject to change yearly. Content is optimized for on-ground or distance learning. Content is written into official course objectives, ensuring its presence regardless of staffing changes. There is complete buy-in and understanding from faculty and course directors, who have been involved in the creation and implementation of the scaffolded curriculum. IL topics are matched to course projects in order to facilitate learning. In some cases, the librarian assists in creating major projects for the course that revolve around IL. The program has been well received by both students and faculty.
Conclusion: IL integration is possible in a DNP curriculum and necessary for students to develop their skills. Ongoing review of library use as well as assessments will continue to direct and improve the program. Preliminary pre/post tests show an improvement of basic IL knowledge, but full reporting cannot take place until a cohort has completed the full 3-year cycle. Faculty and librarians report seeing a marked improvement in searching, critical appraisal, citation management, and overall knowledge of IL. This can serve as a model not only for DNP curricula, but also for other academic programs.

Raising Clinician Genetics Information Literacy

Joe Wu, Bioinformatics Support Specialist, University of Florida Health Science Center Libraries, Gainesville, Florida

Margaret Emily Ansell, AHIP, Nursing & Consumer Health Liaison Librarian, Health Science Center Libraries, University of Florida, Gainesville, Florida

Ariel Pomputius, Assistant University Librarian, University of Florida Health Science Center Libraries, GAINESVILLE, Florida

Michele R. Tennant, AHIP, Head, ARCS, Health Science Center Libraries, Gainesville, Florida

Background: Precision Medicine endorses the use of genetic blueprint to custom-cater patient treatment rather than relying on a one-size-fits-all approach. To be effective in implementing precision approaches to patient care, clinicians need to be well-trained in genetics. Here, a health science library has conducted an outreach project aimed to raise the genetics information literacy of clinicians by teaching various clinicians on campus to navigate online genetics information resources that are pertinent to their medical practice.
Description: In this project, several clinical units on campus agreed that further training on the use of online genetics information resources would enhance their practice. We developed in-person training to demonstrate the use of genetics databases that house information pertaining to
1) Genetic influences of disease and drug response (GeneReviews, OMIM, and Pharmacogenomics Knowledgebase).
2) Appropriate tests to detect disease causing gene mutations and where testing can be done (Genetic Testing Registry).
Databases housing information regarding genetics and health that a clinician could provide as patient education were also introduced. Instructional videos that document the use of these resources were also created and made available online for clinicians to view during their spare time. A four-question survey was sent to gauge effectiveness of in-person training. Success of the videos will be measured by the number of views received a year post-production.
Conclusion: As the hub for information resources and education, this outreach project allowed the project team to enhance the genetics information literacy of clinicians at a university. This opportunity was innovative because the databases presented are authoritative, well organized, and well curated; thus, preventing the need for lengthy literature searches in a busy and fast-paced clinical environment.

Raising Frames of Mind: Elevating Learners Using the ACRL Framework and Active-Learning Strategies Research

Robyn Rosasco, AHIP, Public Services Librarian, College of Medicine, Florida State University, Tallahassee, Florida

Erica L. Heasley, AHIP, Information Services and Outreach Librarian, Florida state University, Tallahassee, Florida

Susan A. Epstein, AHIP, Systems Librarian, Florida State University, Tallahassee, Florida

Objectives: An active-learning approach to information literacy instruction can promote student engagement and higher-order thinking, which complement current instructional standards and conceptual frameworks in higher and medical education. This paper demonstrates the value of active-learning strategies mapped to the Association for College & Research Libraries' (ACRL) Framework guidelines by assessing health sciences students' perceived self-efficacy of their information literacy skills after participating in varied instructional sessions.
Methods: Medical librarian instructors integrated active-learning strategies into, on average, 10 one-shot information literacy lessons per semester for health sciences students at the Florida State University College of Medicine (FSU COM) during spring 2018, fall 2018, and spring 2019. Strategies included guided, abbreviated research simulations; interactive modules for small-group learning; and flipped classroom techniques that required students to submit pre- and post-class assignments. Instructors mapped active- and passive-learning methods to learning concepts identified in appropriate ACRL frames. A cross-sectional, online survey with a 11-point Likert scale that measured perceived self-efficacy for initiating and conducting clinical and scholarly research was distributed to graduate, undergraduate and physician assistant students following information literacy instruction that used active-learning and student-engagement concepts recommended in the ACRL Framework. Quantitative analysis was performed on self-efficacy scores submitted in fall 2018 and spring 2019.
Results: Library instruction sessions and data collection are ongoing. Preliminary results collected from students after library instruction sessions suggest moderate-to-high confidence ratings for information literacy competencies. Based on observation, an unanticipated outcome has been an increase in undergraduate senior capstone students seeking one-on-one research consultations with medical librarians when compared to previous semesters.
Conclusions: We hope active-learning methods connected to conceptual frameworks and educational standards will support formalized integration of information literacy competencies across the FSU COM curricula. Further conclusions will be described at the time of the presentation.
Keywords: ACRL Framework, Active Learning, Information Literacy, Instruction

Understanding Nursing Faculty's Perceptions of the Role of the ACRL Information Literacy Framework in Nursing Instruction Research

Bethany S. McGowan, Assistant Professor of Library Science and Health Sciences Information Specialist, Purdue University Library and School of Information Studies, West Lafayette, Indiana

Laureen Patricia Cantwell, Reference & Distance Services Librarian, Colorado Mesa University, Grand Junction, Colorado

Julie Planchon Wolf, Research & Instruction/Nursing & Health Studies Librarian, UW Bothell & Cascadia College, Bothell, Washington

Alexandra Williams, Instructional Designer, Frontier Nursing University, Oakwood, Ohio

Maribeth Slebodnik, Research & Learning Librarian, University of Arizona, Tucson, Arizona

Rebecca Raszewski, AHIP, Associate Professor & Assistant Information Services & Liaison Librarian, University of Illinois at Chicago, Chicago, Illinois

Jamie L. Conklin, Health Sciences Librarian / Liaison to the School of Nursing, University of North Carolina at Chapel Hill, Chapel Hill, North Carolina

Objectives: A task force is updating the 2013 Information Literacy Competency Standards for Nursing standards to match the recently revised ACRL Framework for Information Literacy for Higher Education. This research seeks to understand how nursing faculty utilize information literacy (IL) concepts in coursework to inform the development of an updated set of nursing IL standards.
Methods: Setting: Nine academic institutions with nursing programs
Population: Nursing faculty who teach any level of nursing students and/or nurses—from associate to doctoral level nursing candidates and practicing nurses.
Study Design: Cross-sectional survey design with 14 close-ended questions related to respondents' IL teaching experience, familiarity with and use of IL standards, and perceived relevance of IL principles to nursing education.
Data Analysis: Researchers will manage and analyze data using Qualtrics.
Research Ethics: The primary institution's IRB has approved the study, and the researchers will gain approval from the remaining institutions.
Results: Eighty-seven nursing faculty completed the survey. Results indicate 79% of respondents are unaware of the ACRL Framework. Thirty-nine percent of respondents said their nursing program had a goal or outcome related to IL, while 27% were unsure. When provided a description of IL, 79% of respondents said they teach these concepts in their courses, through learning objectives, course readings, research sessions from librarians, and modeling, among others. Reasons for IL instruction included student need, instructor interest, institutional student learning outcomes, accreditation, use of Problem-Based Learning, and because keeping current with Evidence-Based Practice requires IL.
Conclusions: Overall, nursing faculty deem IL skills relevant to nursing curriculum and practice yet are largely unaware of ALA guidelines. Some faculty do not teach IL competencies because they either expect students to receive IL instruction elsewhere, leave it to librarians, find it irrelevant, have never considered it, or think it is outside their area of expertise. There is an opportunity for librarians to promote the ACRL Framework and IL concepts and to collaborate with nursing faculty in incorporating IL competencies into their curricula.

Elevating Information Literacy Research in Health Sciences Libraries: A Bibliometric Study Research

Alexandria Quesenberry, Assistant Professor/Research & Learning Services Librarian, University of Tennessee Health Science Center, Memphis, Tennessee

Lin Wu, AHIP, Assistant Director for Research & Learning Services, University of Tennessee Health Science Center, Memphis, Tennessee

Objectives: Information literacy (IL) opens opportunities for health sciences librarians to design research related to library instruction sessions and collaboration with faculty to enhance student learning. There is limited published literature to assess health sciences libraries and librarians' productivity on IL research. This study aimed to examine the status and characteristics of IL research in health sciences libraries and performed by health sciences librarians over the period of 2008 to 2018.

Methods: Bibliometrics, the statistical analysis of publications, was used to assess the indicators of productivity, collaboration, and impact or visibility on IL research by health sciences librarians. Data was collected from Scopus and Web of Science, two citation databases with multidisciplinary and international scopes. EndNote software was used to remove duplicates and Rayyan was used to systematically screen search results for inclusion. Quantitative analysis was performed, including citation analysis, number of publications per year and per country, the most prolific authors and their affiliations, most prolific journals, number of articles published in library science-related and non-library science-related journals, the origin of the first author's country, etc.
Results: Of the 3,387 search results, 479 (14.8%) unique articles met the inclusion criteria of being authored by health sciences librarians. Study results showed that the number of included publications on IL increased 13% each year on average with the peak in 2016 (n=62). Over half (54%, n=258) of included articles were published by authors affiliated within the United States. Health Information and Libraries Journal was the most prolific journal that published IL studies in the domain of health sciences librarianship. However, the most highly cited article was published in the Journal of the Medical Library Association. In terms of visibility, 373 (78%) articles have been cited at least once, and the average citation number is 7.8. Authors were highly collaborative with a collaboration index of 2.8.
Conclusions: Over the ten-year period, the volume of IL literature authored by health sciences librarians has increased. Health sciences librarian researchers tend to publish in health sciences library/information journals. This study provides insight for health sciences librarians on IL research and publication practices. Further research might be needed to examine differences in IL publication characteristics after the 2016 implementation of the Association of College and Research Libraries (ACRL) Framework for Information Literacy.

Tuesday, May 7, 2:00 p.m.–3:25 p.m.
Room: Grand Ballroom A (East Tower, Ballroom/Gold Level)

Global Health & Health Equity Immersion Session 4

Moderator: Brenda M. Linares, AHIP

Microaggressions and More: Continuing the Conversation on Implicit Bias

Andy Hickner, Jersey City, Connecticut

Diana Almader-Douglas, AHIP, Librarian, Mayo Clinic, Phoenix, Arizona

James Eddy Anderson, Physician Assistant, Evergreen Treatment Services, Seattle, Washington

Shannon D. Jones, AHIP, Director of Libraries, Medical Univ. of South Carolina, Charleston, South Carolina

Brenda M. Linares, AHIP, Health Sciences Librarian, University of Kansas Medical Center, Olathe, North Carolina

Hannah Rutledge, AHIP, Head of Clinical Informationist Services, Emory University, Atlanta, Georgia

Megan Threats, Doctoral Candidate, University of North Carolina at Chapel Hill, School of Information and Library Science, Durham, North Carolina

Tara Douglas-Williams, Library Manager, Information Services, Morehouse School of Medicine, Atlanta, Georgia

Brenda M. Linares, AHIP, Health Sciences Librarian, University of Kansas Medical Center, Olathe, North Carolina

This session is part of an ongoing series of MLA programs exploring implicit bias. Participants will learn about and discuss key themes on implicit bias. We will focus in particular on the concept of microaggressions, including defining the term, providing concrete examples of microaggressions, and sharing techniques for responding to them. An expert on these topics will share evidence on how microaggressions and implicit bias impact patients with marginalized identities, after which a panel of MLA members will share their experiences with implicit bias and microaggressions. There will be a variety of opportunities for audience participation in this highly interactive session, including Q&A for the panelists and the opportunity for audience members to share their own perspectives.

Tuesday, May 7, 2:00 p.m.–3:25 p.m.
Room: Columbus EF (East Tower, Ballroom/Gold Level)

Information Management Immersion Session 4

Moderator: Michelle B. Bass, AHIP

“Information Management for a Thousand, Alex”: Play Jeopardy! to Develop Your Understanding of the MLA Information Management Competency

Michelle B. Bass, AHIP, Manager, Research and Instruction, Harvard Medical School, Countway Library of Medicine, Boston, Massachusetts

Sharon R. Willis, Senior Cataloging Specialist, NLM Cataloging and Metadata Management Section (CAMMS), Bethesda, Maryland

Sharon R. Willis, Senior Cataloging Specialist, NLM Cataloging and Metadata Management Section (CAMMS), Bethesda, Maryland

Michelle B. Bass, AHIP, Manager, Research and Instruction, Harvard Medical School, Countway Library of Medicine, Boston, Massachusetts
Medical librarians should learn more about the revised MLA competencies. This session will be a fun way to become more familiar with Competency 2 (Information Management) based on content developed by experts in the field, the Jeopardy! game played by members of the audience, and our own MLA version of Alex Trebek. The format for information gathering will be Jeopardy but each audience member will have their own Jeopardy board for point gathering to self-grade their basic to expert competencies on the seven performance indicators. Sponsored by the Technical Services Section and Research Section.

Tuesday, May 7, 2:00 p.m.–3:25 p.m.
Room: Columbus IJ (East Tower, Ballroom/Gold Level)

Information Services Immersion Session 6

Moderator: Emily Patridge, AHIP

Establishing a Successful Data Visualization Service: Lessons from the Field

Emily Patridge, AHIP, Assistant Director of Clinical Research and Data Services, University of Washington, Seattle, Washington

Vedana Vaidhyanathan, Health and Life Sciences Librarian, Baylor University, Waco, Texas

Benjamin Hoover, Library Associate Director Coordinator for Innovative Technology Services, The Pennsylvania State University, Hershey , Pennsylvania

Terri Gotschall, AHIP, Scholarly Communications Librarian, University of Central Florida College of Medicine, Orlando, Florida

Lynly Beard, Research Impact and Social Work Librarian, Health Sciences Library, Seattle, Washington

Emily Patridge, AHIP, Assistant Director of Clinical Research and Data Services, University of Washington, Seattle, Washington

Tania P. Bardyn, AHIP, Associate Dean & Director, University of Washington, Seattle, Washington

Emily Patridge, AHIP, Assistant Director of Clinical Research and Data Services, University of Washington, Seattle, Washington

The librarians presenting this panel will discuss the challenges and solutions in setting up a variety of data visualization services for academic libraries. They will share tips and lessons learned from the field covering data mining, creating data visuals workshops, utilizing different visualization programs and enhancing their work with faculty with data visualizations. The panelist will provide a description of data visualization services for audience members to take with them.

Tuesday, May 7, 2:00 p.m.–3:25 p.m.
Room: Columbus AB (East Tower, Ballroom/Gold Level)

Information Services Session 5

Moderator: Elizabeth Dyer, AHIP

Evaluation of Literature Searching and Article Selection Skills of an Evidence Synthesis Program Research

Emily P. Jones, AHIP, Research and Education Informationist, Medical University of South Carolina, Charleston, South Carolina

Emily Brennan, Research and Education Informationist, Medical University of South Carolina (MUSC), Charleston, South Carolina

Amanda Davis, Sr. Evidence-Based Practice Analyst, Medical University of South Carolina, Charleston, South Carolina

Objectives: A quality improvement project was initiated to evaluate literature search and article selection skills of a new librarian (less than 2 years' experience), a mid-career librarian (more than 10 years' experience), and a critical appraisal expert. This team works together to create Evidence Briefs by request for clinicians at an academic medical center.
Methods: Evidence Briefs include an evidence table with a summary of best evidence for a single clinical question and are used to update hospital policies and protocols. For this project, each librarian and the critical appraisal expert developed individual search strategies for 10 Evidence Brief requests. They then selected what they considered to be the most relevant articles to answer the clinical question. These articles were combined into one RefWorks folder and de-duplicated. The critical appraisal expert reviewed the RefWorks folder and determined, based on study design, directness and actionability, which articles to include in the Evidence Brief. Each individual's search results and most relevant articles were assessed to determine the proportion included in the Evidence Brief. These proportions were ranked for each of the 10 Evidence Briefs to determine searching and selection capability for each individual and overall.
Results: The mid-career librarian's search strategy captured the highest number of articles included in 6 of 10 Evidence Briefs, followed by the critical appraisal expert (3 of 10) and the new librarian (1 of 10). The search where the new librarian, also the pharmacy liaison, was most adept was a medication question. The critical appraisal expert identified the highest number of relevant articles included in 7 of 10 Evidence Briefs, followed by the mid-career librarian (4 of 10) and the new librarian (2 of 10). There were some Evidence Briefs where multiple individuals were equally ranked based on search strategy or article selection.
Conclusions: The mid-career librarian and critical appraisal expert identified the most citations selected for inclusion in Evidence Briefs overall. However, the critical appraisal expert was more accurate at selecting articles for inclusion in Evidence Briefs. This project may lead to changes in workflow, and

offers opportunities to improve the literature searching skills of the new librarian and article selection skills of the experienced librarian.

Applying Text Mining Analytics to Virtual Reference Services: A Case Study on the Email Question & Answer (Q&A) Service at an Academic Health Sciences Library

Fei Yu, Health Informatics Librarian, Health Sciences Library at the University of North Carolina at Chapel Hill, Cary, North Carolina

Nandita S. Mani, AHIP, Associate University Librarian & Director, Health Sciences Library, University of North Carolina, Chapel Hill, Chapel Hill, North Carolina

Jennifer S. Walker, Cancer Information Librarian / Liaison to the School of Dentistry, University of North Carolina at Chapel Hill, Chapel Hill, North Carolina

Background: Virtual reference services have been widely adopted by academic libraries for almost two decades. Patrons can ask librarians for help either through live chat or via a web email form. The purpose of this study is to investigate how the web email service has been used at an academic health sciences library by applying text mining analytics to email inquiries.
Description: The Health Sciences Library (HSL) at a large research university in the Southeastern U.S. was the study site. One-year, de-identified email records (January-December 2016) collected through the "Ask Us" Web form at the HSL website were used as the data sample.
Python scripts were developed to clean data, remove stop words, build datasets, and perform basic term-frequency counting. In addition, LightSide and VOSviewer were adopted as text-mining tools. The former was used to investigate the correlation between the top extracted terms from the datasets and patrons' Ask-for-meeting requests; the latter was applied to extract and visualize the key topics in patron questions.
Conclusion: Patrons from the University's School of Public Health used the Email Q & A service the most frequently. Particularly, students submitted more inquiries than faculty or staff. In 2016, the heaviest usage of this service occurred in September; Monday through Wednesday, and the beginning and ending of a month were often the peak time. There is some positive correlation between certain terms in patron inquiries (e.g., "literature review," "request consultation") and the Ask-for-a-meeting request. The key topics of patron questions include "systematic literature review," "endnote," "search," "access," and "submission." This study helps HSL better allocate time, personnel needed to provide support, and resources.

Anatomy of a Clinical Research Paper: IMRAD and CONSORT

Terry Kit Selfe, AHIP, University of Florida, Gainesville, Florida

Sarah Meyer, Assistant University Librarian, University of Florida, Gainesville, Florida

Background: Knowing how medical journal articles are organized and where specific content is likely to be found, improves students' ability to read articles critically, efficiently, and confidently. Our objective is to demonstrate how a clinical research article is ideally organized according to the International Committee of Medical Journal Editors (ICMJE) recommendations and the CONsolidated Standards of Reporting Trials (CONSORT) guidelines for randomized controlled trials. By the end of the presentation, attendees should be able to identify where specific content should be found in a well-written article, and apply this knowledge to instruct students to efficiently read and write clinical research articles
Description: ICMJE has updated widely adopted recommendations specifying what should be reported in a scholarly article published in a medical journal. While much of what is covered by ICMJE is standard across article types, there are some differences based on study design (e.g., for randomized trials ICMJE recommends following CONsolidated Standards of Reporting Trials (CONSORT) guidelines). These standards can be successfully used as a reference to become familiar with the organizational structure of medical journal articles. We demonstrate the Introduction, Methods, Results, and Discussion (IMRAD) structure prescribed by ICMJE, as well as the content to be contained in each section per the CONSORT guidelines. This content has been presented to different audiences in various venues, including a library stand-alone class on critical appraisal; guest lectures to MD/PhD students, medical residents, and fellows; and new researchers writing their first journal article.
Conclusion: Becoming familiar with the ICMJE and CONSORT recommendations can aid students, researchers, and librarians in understanding how medical journal articles are organized and where specific content is likely to be found; thus enhancing their ability to efficiently read and critically appraise clinical research articles with confidence. In addition, the guidelines are a useful resource to present to researchers authoring a research article for the first time; as they provide direction to what can be a daunting task, and if followed, they will likely elevate the quality of the resulting journal article.

Measuring the Attitudes of Academic Health Sciences Libraries Library Directors toward Discovery Tools Research

Harold S. Bright, IV, AHIP, Arizona Campus Branch Manager / Electronic Resources Librarian, A.T. Still Memorial Library, Scottsdale, Arizona

Kirsty Gaither

Objectives: To identify if academic health sciences library (AHSL) Library Director attitudes and perceptions towards Discovery Tools are affecting the lower (but growing) adoption rate of Discovery Tools as compared to general academic libraries.
Methods: We identified 158 AHSL (Academic Health Sciences Libraries) institutions from the annual AAHSL (Association of Academic Health Sciences Libraries) survey and the AAHSL member web site. Several data points were collected in 2013 and updated in 2018 from each AHSL site, the use/nonuse of a discovery tool being one. Survey questions were developed after consulting the literature and in conjunction with several library colleagues. The survey instrument was built in Qualtrics and will be distributed to AHSL library directors in late 2018 via individual links through email messages sent in Qualtrics. Separate tracks with similar questions will be used for institutions that have and do not have discovery tools. We will identify themes and attitudes of these directors towards discovery tool use in academic medical libraries.
Results: AHSL directors who have implemented discovery tools generally feel the implementation is successful. Students and faculty both use them and a majority of directors felt their users would be

dissatisfied if the tools were discontinued. Contrasted with this, AHSL directors without discovery tools felt they would not be useful nor would their user groups care if the tool were deployed by their library. Cost and functionality were the main reasons for deployment and non-deployment.
Conclusions: Upon conducting our research we have found that external factors such as budget/cost play a significant role in whether or not a library adopts a discovery tool, functionality plays a minor well as well. When it works well, a discovery tool simplifies and makes resource discovery easier and more central, but they are perceived as a generalist or undergraduate tool, not a specialty tool. Discovery tools are friendlier for the growing generations of technological natives and their desire and expectation of a one-stop-shop for information. The future of discovery tools within AHSL may hinge on two factors: either a lowered cost to justify their purchase or improved function whereby directors' attitudes toward these tools would be favorable enough to merit the additional expenditure. Given the widespread adoption by general academic libraries, the needs of this specialized community may need to catch up as we come to the generation of students who have never been taught to search outside of a generalized tool.

Variation and Outliers in Search Results among MEDLINE-Based Platforms: A Longitudinal Study Research

Tyler Nix, Informationist, University of Michigan, Ann Arbor, Michigan

Robert M. Shapiro II, Visiting Assistant Professor, University of Kentucky, Lexington, Kentucky

C. Sean Burns, Assistant Professor, School of Information Science, Lexington, Kentucky

Jeffrey T. Huber, Director and Professor, University of Kentucky, Lexington, Kentucky

Objectives: This study is part of a larger project to understand the search result variations across MEDLINE-based platforms. With this current research, we seek to examine longitudinal changes in how five MEDLINE-based platforms respond to basic searches.
Methods: We examined five MEDLINE-based platforms by creating sets of queries for each platform and comparing search count results. The five platforms include: EBSCOhost/MEDLINE, Ovid/MEDLINE, ProQuest/MEDLINE, PubMed/MEDLINE, and Web of Science/MEDLINE. Our queries were organized into 29 sets of five queries each (one query per platform). The five queries in each set were designed to be equivalent and were modified only to match the syntax required by the each platform. Searches are being run monthly for 8 months. To answer our research question, we used a repeated measures approach based on the median absolute deviations (MAD) and the modified z-scores (m_i) to examine the search result counts in each query set. We centered our scores on the PubMed count in each set of queries in order to compare how result counts deviate from PubMed/MEDLINE.
Results: Initial results from one month of search results indicate substantial variance across platforms. Web of Science/MEDLINE results were most likely to deviate from PubMed/MEDLINE followed by ProQuest/MEDLINE. Both EBSCO/MEDLINE and Ovid/MEDLINE exhibited deviations, though Ovid/MEDLINE returned results most consistent with PubMed/MEDLINE. Preliminary longitudinal results seem to reflect initial findings, with continued and substantial variances across platforms.
Conclusions: It appears proprietary, platorm-specific interventions (possibly including data ingest workflows, term indexing and retrieval algorithms, and interface features) are affecting the retrieval of

structurally similar searches across MEDLINE-based platforms. These variances have the potential to impact clinical decision-making, reproducibility studies, systematic reviews, or any research which depends on the consistent and predictable performance of searches.

Tuesday, May 7, 2:00 p.m.–3:25 p.m.
Room: Columbus CD (East Tower, Ballroom/Gold Level)

Innovation & Research Practice Immersion Session 6

Beyond the Data Management Plan: Expanding Roles for Librarians in Data Science and Open Science

Lisa Federer, AHIP, Data Science and Open Science Librarian, National Library of Medicine, North Bethesda, Maryland

Lisa Federer, AHIP, Data Science and Open Science Librarian, National Library of Medicine, North Bethesda, Maryland

Lisa Federer, AHIP, Data Science and Open Science Librarian, National Library of Medicine, North Bethesda, Maryland

Ariel Deardorff, Data Services Librarian, UCSF Library, San Francisco, California

Margaret Henderson, AHIP, Health Sciences Librarian, San Diego State University Library, Ramona, California

Elaine Russo Martin, Director and Chief Administrative Officer, Francis A. Countway Medical Library, Shreswsbury, Massachusetts

Jian Qin

Kevin Read

John Borghi, Data Services Librarian, Lane Medical Library / Stanford University, Stanford, California

Support for research data management (RDM) has become a popular focus for library services, and many courses exist to prepare librarians to take on new roles in RDM. However, fewer opportunities exist for librarians to develop the more advanced skills and expertise necessary to support data science and open science. Some libraries have been successful in developing such services, thanks to motivated information professionals with specialized expertise, but this type of knowledge is not currently widespread in the field, nor in iSchool curricula. This session will present the findings of a workshop that convened 15 librarians and information professionals with a range of expertise in data and open science at the National Library of Medicine, with the purpose of gaining insight into how to develop a library workforce that is prepared to move RDM and engage with issues and challenges in the emerging areas

of data science and open science. The audience will not only hear about the findings of this session, but also have the opportunity to engage with several of the participants in a panel discussion.

Tuesday, May 7, 2:00 p.m.–3:25 p.m.
Room: Grand Ballroom B (East Tower, Ballroom/Gold Level)

Professionalism & Leadership Immersion Session 7

Moderator: Edwin Vincent Sperr Jr., AHIP

Using Improvisation to Enhance Communication Skills for Librarians

Amy Baldwin, Associate Professor of Microbiology, AU/UGA Medical Partnership, Athens, Georgia

Edwin Vincent Sperr Jr., AHIP, Clincial Information Librarian, AU/UGA Medical Partnership, Athens, Georgia

Edwin Vincent Sperr Jr., AHIP, Clincial Information Librarian, AU/UGA Medical Partnership, Athens, Georgia

Edwin Vincent Sperr Jr., AHIP, Clincial Information Librarian, AU/UGA Medical Partnership, Athens, Georgia

Librarians, particularly those who engage in instruction, need to communicate effectively to do their jobs well. Improvisational theatre is a discipline that has been used in many settings, including health care education, to promote and improve robust communication skills. In this workshop, we will introduce some basic concepts of improvisational practice and discuss how they are related to mindfulness and more generalized communication skills. More importantly, participants will gain first-hand experience with several improv structures in a supportive, collegial environment.

Tuesday, May 7, 4:30 p.m.–5:55 p.m.
Room: Columbus AB (East Tower, Ballroom/Gold Level)

Clinical Support Immersion Session 4

Moderator: Angela Spencer, AHIP

Strategies for Library Mergers and Centralizing Library Services

Janet Lindsay Hobbs, AHIP, Manager, Library Services, Ventura Community Memorial Health System, Ventura, California

Linda Schwartz, AHIP, Director, Library Services and Patient Education, Lehigh Valley Hospital, Allentown, Pennsylvania

Patricia Ulmer, AHIP, Director, Geisinger Health Sciences Libraries, Danville, Pennsylvania

Heather J. Martin, AHIP, Director, System Library Services, Providence St. Joseph Health, Portland, Oregon

Michelle Kraft, AHIP, Director Medical Library, Cleveland Clinic Foundation, Cleveland, Ohio

Michele Matucheski, AHIP, Clinical Librarian - Library Services & Research Consultant, Ascension Wisconsin Library Services / Clinical Professional Development, Oshkosh, Wisconsin

Angela Faye Tucker, AHIP, Medical Librarian, Memorial Health System, Marietta, Ohio

Angela Spencer, AHIP, Manager, Medical Library, St. Lukes Hospital, Chesterfield, Missouri

Angela Spencer, AHIP, Manager, Medical Library, St. Lukes Hospital, Chesterfield, Missouri
This session will provide success stories and lessons learned from librarians who have gone through library centralization or library integration following hospital mergers. Presentations will cover needs assessment/SWOT analysis, licensing, budgeting, technical challenges, staff, solo perspective, and other considerations.
1) Discuss the benefits of a needs assessment/SWOT analysis when merging
2) List some of the considerations involved in unifying services
3) Study the licensing and budgeting issues faced by merging
4) Recognize technical issues involved in a merger
5) Describe staffing challenges
6) Discuss challenges faced by solos

Tuesday, May 7, 4:30 p.m.–5:55 p.m.
Room: Columbus IJ (East Tower, Ballroom/Gold Level)

Education Immersion Session 6

Moderator: Bradley A. Long, AHIP

Active Learning and Librarians: An Evolving and Impactful Partnership

Bradley A. Long, AHIP, Embedded Health Sciences Librarian, Penn State College of Medicine - University Park Regional Campus, State College, Pennsylvania

Annie Cloud Nickum, AHIP, Research and Education Librarian, University of North Dakota, Grand Forks, North Dakota

Stephanie J. Schulte, Associate Professor/Head, Research & Education Services, Ohio State University Health Sciences Library, Columbus, Ohio

Lori B. Snyder, Collection Development and Digital Resource Management Librarian, Harrell Health Sciences Library: Research and Learning Commons, Hershey, Pennsylvania

Bradley A. Long, AHIP, Embedded Health Sciences Librarian, Penn State College of Medicine - University Park Regional Campus, State College, Pennsylvania

Bradley A. Long, AHIP, Penn State University, State College, Pennsylvania
Many health profession education programs across the country have been undergoing changes to provide rich learning environments to foster student active, self-directed learning to better prepare students as lifelong learners and future health care providers in the rapidly changing health care system. This session will provide attendees with an overview of the librarian's role within an active learning environment in health profession education programs. Specific areas that will be covered include the role of the librarian within active learning sessions; collection development in support of active learning; and the role of librarians serving on curriculum committees, in order to better integrate and embed librarians into active learning. Examples from several institutions will be provided to showcase how librarians' role and contributions to the educational programs help increase the librarian's visibility and elevate the value of the library in its home institution. The audience will also be engaged through the use of a polling mobile app.

Tuesday, May 7, 4:30 p.m.–5:55 p.m.
Room: Grand Ballroom A (East Tower, Ballroom/Gold Level)

Information Management Immersion Session 5

Moderator: Nina Exner

Reach out to Elevate Your Research Data Management Services

Nina Exner, Research Data Librarian, Virginia Commonwealth University, Richmond, Virginia

Nina Exner, Research Data Librarian, Virginia Commonwealth University, Richmond, Virginia

Jessica Koos, AHIP, Health Sciences Librarian, Stony Brook University, Stony Brook, New York

Shannon Sheridan, AHIP, National Library of Medicine Associate Fellow, Drexel Universities, Danville, Pennsylvania

Nicole Contaxis, Data Catalog Coordinator, NYU Health Sciences Library, New York, New York

Tisha Mentnech, Research Librarian for Life Sciences & Research Metrics, North Carolina State University Libraries, Salt Lake City, Utah

Melissa Ratajeski, AHIP, Coordinator of Data Management Services, University of Pittsburgh, Pittsburgh, Pennsylvania

Nina Exner, Research Data Librarian, Virginia Commonwealth University, Richmond, Virginia

Supporting faculty and other stakeholders through research data management (RDM) services relies on building relationships. These relationships depend on a solid understanding of stakeholders' needs, clear communication about library services, and effective library strategies that support outreach. This session will share insights on building relationships around RDM and how to plan and market RDM services. Participants will concentrate on drafting their own outreach plans and role-playing strategies for effective communication.

Tuesday, May 7, 4:30 p.m.–5:55 p.m.
Room: Regency Ballroom D (West Tower, Ballroom/Gold Level)

Information Services Immersion Session 7

Moderator: Michelle Cawley

Harness the Power of Machine Learning and Artificial Intelligence (AI) to Change the Way You Approach Large, Complex Research Questions

Michelle Cawley, Head of Clinical, Academic, and Research Engagement, UNC Chapel Hill Health Sciences Library, Chapel Hill, North Carolina

Nandita S. Mani, AHIP, Associate University Librarian & Director, Health Sciences Library, University of North Carolina, Chapel Hill, Chapel Hill, North Carolina

Arun Varghese, Technical Director, ICF, Durham, North Carolina

Jamie L. Conklin, Health Sciences Librarian / Liaison to the School of Nursing, University of North Carolina at Chapel Hill, Chapel Hill, North Carolina

Rebecca C. McCall, AHIP, Clinical Librarian, University of North Carolina-Chapel Hill, Chapel Hill, North Carolina

Jennifer S. Walker, Cancer Information Librarian / Liaison to the School of Dentistry, University of North Carolina at Chapel Hill, Chapel Hill, North Carolina

Elizabeth Moreton, Clinical Librarian, UNC Chapel Hill, Chapel Hill, North Carolina

Fei Yu, Health Informatics Librarian, Health Sciences Library at the University of North Carolina at Chapel Hill, Cary, North Carolina

Nandita S. Mani, AHIP, Associate University Librarian & Director, Health Sciences Library, University of North Carolina, Chapel Hill, Chapel Hill, North Carolina

Michelle Cawley, Head of Clinical, Academic, and Research Engagement, UNC Chapel Hill Health Sciences Library, Chapel Hill, North Carolina

Adam Dodd, Data Analyst, Health Technology and Informatics, University of North Carolina, Chapel Hill, Durham, North Carolina

Michelle Cawley, Head of Clinical, Academic, and Research Engagement, UNC Chapel Hill Health Sciences Library, Chapel Hill, North Carolina

In this session, we will uncover how machine learning algorithms can be used to transform the way information professionals approach large, complex searches. When faced with a research question that requires a comprehensive search likely to generate thousands or tens of thousands of results, machine learning can be used as a tool in place of limiters that are often artificially imposed simply to limit results (e.g., date limits, additional keyword sets). After providing an overview of basic concepts, we will demonstrate how we have used machine learning to refine search strategies, deduplicate search results, and reduce the volume of search results that must be screened manually. We will also facilitate small-group discussions of barriers to implementing AI-enabled searches at participants' institutions and provide validation data that demonstrates the efficacy and efficiency of these approaches

Tuesday, May 7, 4:30 p.m.–5:55 p.m.
Room: Columbus EF (East Tower, Ballroom/Gold Level)

Innovation & Research Practice Session 7

Moderator: Margaret A. Hoogland, AHIP

Evolving Curricular Assistance Using Custom Question Banks Research

Skye Bickett, AHIP, Assistant Director of Education and Engagement, PCOM Georgia, Suwanee, Georgia

Meghan Di Rito, Education and Outreach Librarian, PCOM Georgia, Suwanee, Georgia

Jiehyun Lee, Assistant Professor, Philadelphia College of Osteopathic Medicine - Georgia Campus, Suwanee, Georgia

Dennis Peffley

Objectives: Students often utilize practice questions to improve understanding and retention of session content and to prepare for exams. However, a major challenge is aligning questions with session learning objectives. The goal of this study is to change student behavior to use questions as a learning tool, create intuitive question searching at the correct level, and align commercial and professor-written question banks.
Methods: A developmental model was created combining faculty-generated questions with commercial question bank questions. Three surveys were sent out to assess perceptions of question bank utilization. The first two surveys were sent out simultaneously, one to faculty and one to students, to assess how practice questions were used for study purposes. Following the developmental model, a pilot study was created using two practice quizzes with faculty-generated practice questions for a pharmacy anatomy course. After the completion of the pharmacy anatomy course, the third survey was sent out to pharmacy students to assess their perception of the utility of practice quizzes and to assess whether student utilization of quizzes had changed.
Results: Twenty-three percent of faculty answered the survey, responding that they create their own questions, but do not direct students to use commercial question banks. Seventy-two students (12.04% response rate) responded to the first survey, indicating that 53% of students use faculty provided questions, 40% use commercial question banks, and 34% use both. The student surveys indicate that while initially most students used question banks to study immediately before a test, after the pilot study, they began using quiz questions earlier. Additionally, the number of students who always incorporate quiz questions into their studying jumped from 37.5% to 50%.
Conclusions: The initial survey showed students inhibit their study strategies by not quizzing themselves early enough. However, the curated set of progressive questions helped students incorporate question banks into their studying. Additionally, they used the question banks to start studying more effectively by quizzing themselves earlier. One lesson we learned is that it is necessary to give ourselves more lead time in between receiving questions from faculty and assigning quizzes to students. Giving ourselves

more lead time gave us time to better organize the quizzes and enhance individual questions by adding question difficulty level and explanations for answers.

Excel-evate: Developing a Data Visualization Workshop in Excel

Fred Willie Zametkin LaPolla, Research and Data Librarian, NYU Langone Health, New York, New York

Background: Our academic medical center library began offering data visualization assuming the majority of interest would be in advanced, technology centered workshops like R coding and specialized tools, but a gap remained in teaching “lower-tech” options. In order to provide programming that is meaningful and useful, we surveyed our community and found that a clear majority of participants indicated interest in Excel visualization. This paper explores how we developed expertise in Excel visualization and began teaching this topic. We find Excel visualization relevant to researchers given the ubiquity of Microsoft Office products and the perceived lower barriers to entry.
Description: To develop the course, we began using online-resources, particularly blogs, and e-books already owned by our university’s library to take an intensive dive in using Excel for data visualization. We demo-ed the class in a small group setting for medical students, and made corrections to content. The class is formatted to be mostly hands-on. The workshop begins with relatively simple visualization tasks and moves on to more advanced features, culminating with "publication-ready" annotated figures that combine chart types. The Excel data visualization workshop that has been hosted four times. Teaching the class has led to iterative updates in content as well as assessment of learning.

Conclusion: To date, over 40 people have taken the workshop. Over half of these have been project coordinators or data analysts. Postdocs and interns collectively represented roughly 25% of attendees. 100% of attendees indicated they definitely or probably will use what they learned. Finally 96% said the material level was just right, and 100% would either highly recommend or recommend the workshop. That said a major challenge in the workshop is uneven levels of computer literacy, as well as difficulty using Excel for advanced visualizations. Based on our experiences we also hope to explore offering workshops in PowerPoint for infographic design.

Designing and Teaching a Hands-on Reproducibility Workshop in REDCap and R

Alisa Surkis, Assistant Director, Research Data and Metrics/Vice Chair for Research, NYU Health Sciences Library, New York, New York

Fred LaPolla, Research and Data Librarian, NYU Health Sciences Library, New York, New York

Kevin Read, Lead, Data Discovery and Data Services Librarian, NYU Health Sciences Library, New York, New York

Mark Butler

Nicole Contaxis, Data Catalog Coordinator, NYU Health Sciences Library, New York, New York

Background: In recent years, there has been increased scrutiny on issues of scientific reproducibility. In 2016, NIH began requiring that grant applications address rigor and reproducibility. Issues of reproducibility arise across the data lifecycle, from collecting to processing to analyzing data. Our library provides lectures on rigor and reproducibility to graduate students, and hands-on training for faculty, students, and research staff in REDCap and R. We saw an opportunity to integrate the hands-on tools-based training into a framework that emphasized issues of reproducibility and highlighted how to use those tools to maximize reproducibility throughout data collection, processing, and analysis.
Description: We developed a full-day reproducibility workshop. Lectures covered clinical research data management taught by a librarian and an introduction to recommended practices for reproducibility taught by a senior data analyst from the Department of Population Health. Hands-on training, taught by librarians, covered REDCap and R. We created two datasets to use for hands-on exercises: clinical study data in REDCap, and EHR data that included data from the REDCap study “participants”. The class covered: 1) best practices in collecting, processing, and analyzing data including best practices in scientific computing, 2) using REDCap’s online designer, shared libraries, and data dictionary feature to create data collection instruments that facilitate reproducibility, and 3) basics of R including the tidyverse packages and R Markdown. The class concluded with a case study using R Markdown to reproducibly process and analyze the simulated REDCap and EHR data.
Conclusion: Eighteen people attended the workshop (55 registrations including waitlist), including faculty, students, and staff. Workshop evaluations were positive (94% would recommend workshop, 94% would use what they learned). However, five students indicated the R instruction was too advanced, and others suggested decoupling the topics or allowing more time for the material. Library instructors concluded that modifications were warranted before offering the workshop again. Issues to consider ranged from the technical -- finding ways to streamline the somewhat time-consuming and error-prone set-up process -- to broader questions such as whether there was value in linking REDCap and R in this way.

Reshaping Evidence-Based Practice with Real-World Evidence: Opportunities and Challenges for Librarians/Information Specialists

Research

Janice Y. Kung, Public Services Librarian, John W. Scott Health Sciences Library, University of Alberta, Edmonton, Alberta, Canada

Dagmara Chojecki, Information Specialist/Librarian, University of Alberta/Institute of Health Economics, Edmonton, Alberta, Canada

Lisa Tjosvold, IHE Research Librarian, Institute of Health Economics, Edmonton, Alberta, Canada

Objectives: The use of real world evidence (RWE) to inform evidence-based practice and decision making is playing an increasingly important role in evidence syntheses. Since librarians are at the front lines of retrieving evidence, this scoping review will evaluate search methodologies used in order to locate RWE studies in systematic reviews and the level of involvement from librarians.
Methods: The research team incorporated the PRISMA Extension for Scoping Reviews (PRISMA-ScR) checklist (1) to increase the transparency of the reporting method for this scoping review. Searches were conducted in Ovid MEDLINE, Embase, ProQuest Dissertations & Theses Global, and Web of Science

for systematic reviews using real world studies. Non-English language publications were excluded. Two independent reviewers screened search results and abstracted the relevant data. Information about the sources searched, search terms and strategies used, and librarian involvement were all examined and narratively synthesised. A total of 361 articles were retrieved and 46 articles met the inclusion criteria for data extraction. One librarian extracted the data while a second librarian verified the work. Conflicts were resolved until consensus was reached.
1 Tricco AC, Lillie E, Zarin W, et al. PRISMA Extension for Scoping Reviews (PRISMA-ScR): Checklist and Explanation. Annals of Internal Medicine. September 2018. doi:10.7326/M18-0850.
Results: Of the 46 included studies, no consistent definition of RWE was used. 59% of studies were funded by industries and drug companies. The majority of reviews searched Embase, Cochrane, Pubmed, and MEDLINE for RWE. Only 46% of studies used keywords related to RWE and 24% of studies used subject headings to retrieve RWE. Librarian involvement in these reviews was minimal.
Conclusions: Overall, the searches were not well reported. Based on the studies reviewed, developing a search filter to identify RWE would be difficult and complex due to the inconsistency of how RWE is defined and the breadth of RWD. Since few librarians were involved in the systematic reviews, future RWE studies may benefit from having expert searchers on the team.

Trends in Librarian Evidence-Based Practice Instruction Research

Stephanie Tomlinson, Research and Education Librarian, Shimberg Health Sciences Library/ University of South Florida, Tampa, Florida

Ardis Hanson, Assistant Director, Research and Education, Shimberg Health Sciences Library / University of South Florida, Tampa, Florida

William D. Kearns, Associate Professor (Meritorious, Retired), Child and Family Studies, University of South Florida, Tampa, Florida

John Orriola, Asst. Librarian, University of South Florida, Tampa

Randall L. Polo, Research & Education Librarian, Shimberg Health Sciences Library / University of South Florida, Tampa, Florida

Allison M. Howard, AHIP, Research and Education Librarian, Shimberg Health Sciences Library / University of South Florida, Tampa, Florida

Krystal Bullers, AHIP, Emerging Technologies Librarian, Liaison to the College of Pharmacy / Assistant Librarian, Shimberg Health Sciences Library / University of South Florida, Tampa, Florida

Using survey research, the authors hope to build an understanding of standard practices for embedded instruction in evidence-based medicine courses.
Objectives: The primary objective if this research study is to understand the level of librarian involvement in teaching Evidence-Based Practice (EBP). The secondary objective is to identify prevailing andragogical practices.

Method: The IRB-approved mixed-methods Qualtrics® survey was sent to health sciences librarians. It included questions on demographics, levels of librarian involvement, types of instruction, discipline-specific faculty collaboration, and curriculum design. We examined the survey data for descriptive measures.

Competencies met:

C1) Information Services - What services are provided to health science school students by embedded librarians and what services could be provided?

C5) How is EBM taught by librarians in embedded health science programs?

Results: The survey was sent to MLA MEDLIB-L twice. This data was analyzed using the Qualtrics® proprietary analysis resources. Of the 107 completed surveys 82 respondents self-reported being involved in EBP curriculum.

Conclusions:

We examined the data for major trends, and discussed implications for future research. Selected trends we identified were lack of engagement with journal clubs, lack of involvement in critical analysis, and institutional barriers to librarian engagement in EBP curriculum. These factors will be examined more closely as we build our professional development goals addressing evidence based practice.

Tuesday, May 7, 4:30 p.m.–5:55 p.m.
Room: Grand Ballroom B (East Tower, Ballroom/Gold Level)

Innovation & Research Practice Session 8

Moderator: Holly S. Kimborowicz, AHIP

Collaborating with Clinical Faculty to Create a Library-Based Virtual Reality Studio

Michael Moore, Grants & Special Projects Librarian, University of Washington Health Sciences Library, Seattle, Washington

Tania P. Bardyn, AHIP, Associate Dean & Director, University of Washington, Seattle, Washington

Adam Garrett, Assistant Director, Systems, University of Washington Health Sciences Library, Seattle, Washington

Deric Ruhl, Linux System Administrator, University of Washington, Seattle, Washington

Gili Meerovitch, Principal, Pfeiffer Partners Architects, None

Background: The University of Washington (UW) Health Sciences Library (HSL) partnered with surgeons, nurses, radiologists, research scientists, and programmers to establish a library-based virtual reality (VR) studio to support pre-surgical consultations for cardiothoracic surgery. The project was designed to allow the UW cardiothoracic surgery team to use static, two-dimensional MRI and CT images to create patient-specific and fully interactive three-dimensional models that could be viewed using virtual reality hardware and software. The results of the project were disseminated to other health sciences libraries through a how-to primer. This project was funded by an Institute of Museum and Library Services Leadership Grant.
Description: HSL converted its existing Translational Research and Information Lab (TRAIL) to support VR as an additional service layer, purchasing a VR-capable laptop and Oculus Rift and HTC Vive VR headset packages. The project team conducted full- and half-day retreats to test and demonstrate software, identify core requirements for implementation into the clinical workflow and pre-surgical consultations, and map future clinical and educational uses for VR. Software include Pyrus Medical's Bosc, designed at UW, and other commercially-available products. A design architect was hired to provide feedback and best practices for selecting and renovating a room to host VR, with topics including space considerations, natural and artificial light, privacy, and seating. To disseminate the findings, HSL staff designed and wrote a 44-page how-to primer for other health sciences libraries interested in introducing VR.
Conclusion: Enthusiasm for VR and potential implementation into clinical workflows and curricula was high among all participants, across all experience levels. Though VR has advanced significantly over the last decade, this project discovered that current software isn't sufficiently refined to be implemented

into the clinical practice for real surgical procedures. Surgical teams are too busy to learn new applications still in the development stage. HSL will continue to offer as a service for non-clinical applications. Printed copies of the primer were mailed to AAHSL directors and other interested parties, and a PDF placed on the Health Sciences Library website (https://hsl.uw.edu/vr-studio/).

Developing a Suite of BD2K-Funded Learning Modules Related to Next-Generation Sequencing

Rob Wright, Basic Science Informationist, Welch Medical Library, Baltimore, Maryland

Background: The creation, sharing, reanalysis, and recombination of biomedical big data, including next-generation sequencing (NGS) data, offer unprecedented opportunities for new discoveries in biology and human health. This project fulfills the requirements of an R25 grant from the NIH Big Data to Knowledge (BD2K) Initiative for developing open educational resources to facilitate the sharing of biomedical big data. It focuses on NGS data through five open online learning modules that address: (1) the NGS research life cycle, (2) ethics, (3) data sharing, (4) experimental design, and (5) data storage. The modules are targeted to researchers, clinicians, and the informationists/librarians who support them.
Description: Each module was developed by an instructional coordinator working with subject matter experts. The instructional coordinators included a data librarian, a basic science informationist, and a data management consultant. The primary subject matter expert was a research scientist and an authority on bioinformatics and the application of NGS. Module content was organized into topic-specific short lessons, which include background information, scenarios, important considerations, and resource lists. A separate instructional design team loaded this content into an e-learning platform along with graphics and expert-narrated videos. A designated project manager monitored progress toward goals, and experts on an advisory board and an evaluation panel offered key feedback. The modules are hosted locally and linked from the BD2K Training Coordinating Center website. The project evaluation plan includes monitoring details about how the modules are being used and administering user surveys.
Conclusion: This project was designed to fill a gap in online educational materials about NGS. It offers both essential information about specific aspects of NGS projects and a bird's-eye view showing how these aspects are related and must be planned for. User engagement will help gauge whether the project modules successfully fill this gap. Usage data will be tracked, including the number and location of users and the modules used. User surveys will help measure to what extent the modules support the work of their intended audiences, including the informationists/librarians supporting researchers and clinicians.

Elevating Engagement with Health Sciences Faculty: The Implementation of Research Sprints at the University of Minnesota

Shanda Hunt, Public Health Librarian & Data Curation Specialist, University of Minnesota, Minneapolis, Minnesota

Sarah Jane Brown, Medical School Liaison, University of Minnesota, Minneapolis, Minnesota

Jenny McBurney, Research Services Coordinator for Social Sciences and Professional Programs, University of Minnesota Libraries, Minneapolis, Minnesota

Background: The University of Minnesota Libraries adopted Research Sprints as an innovative faculty engagement approach. The primary goal was to offer faculty the opportunity to partner with a team of expert librarians on a specific research or pedagogical project during an intensive 4-day period, and differed from traditional consultations in timing and depth of interaction. A secondary goal was to solidify the status of librarians as valuable collaborators. The Libraries hosted two iterations of Research Sprints in May of 2017 and 2018 and evaluated both using surveys and reflection.
Description: A call for Sprint proposals was announced to faculty in January. A core planning committee utilized a rubric to assess proposals for feasibility, impact, and fit with the Libraries' goals. Seven proposals were selected in 2017 and six in 2018. The planning committee assembled teams of library staff possessing skills and knowledge aligned with each proposal. Of the 30 applications received, five were from the health sciences, one of which was selected for participation in the Sprints. This project led to the creation of a website [publichealth.dash.umn.edu] that houses freely-available resources for eight public health topics and numerous professional development tutorials. Faculty not selected were contacted for collaboration on their proposal outside of the Sprint setting. These proposals (Public Health, Medicine, and Nursing) led to face-to-face consults and comprehensive literature reviews, and continue to be ongoing collaborations.
Conclusion: Assessment of the 2017 Sprints led to changes for 2018: a formalized charter, streamlined rubric and templates, an orientation session for library staff, and additional pre-Sprint meetings. Library (92%) and faculty (94%) participants reported that the Sprints met or exceeded expectations. The potential impact of Sprints is exemplified in the curricular integration of the public health website at Hanoi Medical University, but even proposals not selected benefited from submitting an application. Research Sprints proved to be an effective engagement tool with faculty from any discipline, but perhaps offer health sciences faculty the upper hand they need in their demanding fields.

Using Suma to Assess Space and Resource Use of an Academic Veterinary Medical Library

Erin RB Eldermire, Head, Flower-Sprecher Veterinary Library, Cornell University Library, Ithaca, New York

Background: A small academic veterinary library with a limited budget and staff wished to improve our spaces and resources. We used Suma to collect information on how our users are utilizing our furniture, resources, and spaces. Suma is an open-source, tablet-based software for collecting and analyzing observational data to help understand space and resource use. In the fall of 2018, we collected data to capture use patterns across the semester. Suma was easy to use, and trends quickly emerged and helped us to make informed decisions, to streamline our efforts and resources, and to uncover future assessment needs.
Description: At our small academic veterinary library, users frequently make individual requests to staff on how they would like us to improve the services and resources. Although it is helpful to receive such requests, it can be difficult to prioritize which to fulfill and which to defer, particularly in light of the limited budget and staff resources that we have. To strategically inform how we can improve our space, we undertook a comprehensive assessment of our spaces and resources to best address user needs and behaviors. We used Suma to collect observational data over nine weeks that were targeted to capture

the variety of library use over a semester. During collection periods, data were collected five times per weekdays and two times per weekends. Data collection took 5-15 minutes per collection session and required the participation of five individuals.
Conclusion: This assessment tool was easy to use, but its data interpretation was complicated. Nevertheless, results helped us to understand practical issues such as how to shift our computer fleet, and which pieces of furniture are used heavily versus those that are rarely touched. It helped us to understand how library patrons use our space, including a typical group size, and the activities that they undertake while in the library. Finally, this project helped us to develop valuable relationships with library patrons who were excited to see that we are paying attention to how they use the spaces that we provide, and impromptu conversations with them led to excellent feedback on how to improve our library.

Tuesday, May 7, 4:30 p.m.–5:55 p.m.
Room: Columbus GH (East Tower, Ballroom/Gold Level)

Professionalism & Leadership Session 8

Moderator: Bette Bissonnette

Librarians and ECHO: Forging High-Impact Partnerships to Improve LGBTQ+ Health Care Outcomes

Laura Menard, Assistant Director for Medical Education and Access Services, Indiana University, Indianapolis, Indiana

Background: Project ECHO (Extension for Community Healthcare Options) leverages technology to reduce healthcare knowledge disparities. This program uses case-based and didactic learning to provide virtual training on LGBTQ+ healthcare to clinicians all over the US. Our ECHO "hub" is based out of a large university system and consists of nine expert clinicians and healthcare providers willing to train others who are interested in improving their knowledge and skillsets relevant to LGBTQ+ patients. In addition to our expert clinical hub team, a medical librarian is embedded in each session to answer clinical questions in real time using library resources.
Description: The LGBTQ+ ECHO hub team reached out to the librarian in August of 2018 based on her presence on the library's LGBTQ Healthcare libguide. The librarian met with the team and agreed to a bi-weekly commitment of two hours in order to participate live in each ECHO session. The focus of these sessions is a case presentation and discussion among participants, and the hub team felt that it would be helpful to have live information and resource support. Based on the one case study available on an embedded librarian in an ECHO program, library administration agreed that this would be a productive use of time. At time of submission, the librarian has participated in 5 sessions and contributed an average of 10 resources per session, with an additional 12 sessions to be completed prior to the Annual Meeting in May.
Conclusion: This program is tentatively slated to run for a year, with continuation possible based on participant survey results. Outcomes that the librarian expects to measure to show impact include number of questions answered per session, number of resources provided per session, and overall participant satisfaction and self-reported learning based on the ECHO participant survey completed at the end of each session. The librarian also intends to survey the hub team to gauge their perceptions of the value of this partnership at the six month mark in March of 2019.

Raising Awareness of Diversity: One Chapter’s Experience

Brenda M. Linares, AHIP, Health Sciences Librarian, University of Kansas Medical Center, Olathe, North Carolina

Beverly Murphy, AHIP, FMLA, Asstistant Director, Communications and Web Content, Hospital Nursing Liaison, Duke University Medical Center LIbrary & Archives, Durham, North Carolina

Tony Nguyen, AHIP, Executive Director, National Network of Libraries of Medicine, SE/A Region, Baltimore, Maryland

Ene O. Belleh, AHIP, Clinical Information Librarian, Penn Medicine, Philadelphia, Pennsylvania

Carenado Davis

Background: In 2015, the Diversity Task Group was appointed to investigate ways the Chapter could encourage, develop and promote diversity within Chapter Membership as well as promote diversity within the profession of medical librarianship. The group developed a survey for its members to get feedback on diversity and important issues the group could address. This presentation will highlight lessons learned about creating a diversity committee/task force, results from the membership survey about diversity topics, and the plan of action to address the issues of diversity in response to the survey results.
Description: To create an action plan to promote diversity within the Chapter membership, the committee developed an in-depth survey of 27 questions approved by IRB. The survey was sent via email to the Chapter membership with a corresponding 39% respondent rate. The data gathered from this survey will be analyzed and evaluated to create an action plan with recommendations for the Chapter to start implementing in the organization. Potential solutions include conference programming and continuing education opportunities.
Conclusion: Analysis is underway. Based on survey results, an initial committee recommendation has been to invite a speaker on implicit bias to address attendees at the Annual Conference.

Distracted in the Library: Supporting Staff and Patrons with ADHD

Kate Flewelling, Executive Director, National Network of Libraries of Medicine, Middle Atlantic Region, University of Pittsburgh, Pittsburgh, Pennsylvania

Background: Millions of adults in the United States have Attention-Deficit/Hyperactivity Disorder (ADHD). Many others may exhibit symptoms without an official diagnosis. Library staff and patrons may be hesitant to disclose that they have the condition, due to stigma and other factors. After a short case study of its author, recently diagnosed with ADHD as an adult and a leader in her library and the profession, the presentation will provide resources for finding information about the disorder and common sense ways that libraries, instructors and managers can support people with ADHD.
Description: After being diagnosed with ADHD as an adult, the author has researched, tried and implemented numerous tools to improve her executive functioning, productivity and skills as a manager and as a current graduate student. The presentation will also describe available research into how library instruction, space and services can be designed to support patrons with ADHD. Resources for supporting staff with ADHD, such as relevant university departments, potential accommodations, and ways to fight stigma and shame, will also be discussed.
Conclusion: Adults with ADHD can be assets to academic libraries and health professions. Many adults with ADHD are creative thinkers and innovators. With little to no cost, libraries can become better workplaces and learning environments to people with ADHD and neurotypical people alike. As trusted

sources of information and training, libraries can be powerful tools to fight misconceptions and misinformation about ADHD and people with the disorder.

Diversity in Degrees in Libraries

Tara Douglas-Williams, Library Manager, Information Services, Morehouse School of Medicine, Atlanta, Georgia

Brenda M. Linares, AHIP, Health Sciences Librarian, University of Kansas Medical Center, Olathe, North Carolina

J. Michael Lindsay, AHIP, Head of Collections and Access Services, Preston Medical Library, University of Tennessee Graduate School of Medicine, Knoxville, Tennessee

Background: Libraries have taken advantage of the opportunity to include non-MLS members on their health information team to better serve their clients. With the addition of PhD's, bioinformatics specialists and others, libraries are adding expertise in data management and big data that improves our value to those we serve. As MLA strives to engage non MLIS team members in professional activities; this discussion will provide an opportunity for this group of members to share their experiences and how the libraries, as well as, they have learned and benefitted from being a member of health information teams.
Description: The goal of this panel discussion will be to engage attendees in understanding the roles and opportunities for non MLS colleagues and how these professionals can improve the ability of libraries to serve patrons by helping to build interdisciplinary teams. Participants will also gain perspective on how working with colleagues from different disciplines can help librarians understand the needs of patrons more effectively. This session will also include a panel discussion and non - MLIS team members will share their experiences in the health information profession and the benefits of inclusion on our library teams.
Sponsors: AAMLA, LMS and MLES
Panelists:
Kristi L. Holmes, PhD, Northwestern University, Galter Health Sciences Library
James "Jim" Anderson, PA-C, MPAS - Evergreen Treatment Services
Shenita Peterson, MPH - Emory University, Woodruff Health Sciences Library
Jackie Wirz, PhD - Ohio State University(video)
Conclusion: In conclusion, as a profession it is important that we continue to embrace the concept of diversity in degrees and seek individuals who will enhance our instruction and learning.
The growth in importance of the research enterprise in medical education has revealed the need for library expertise in bioinformatics, big data, biostatistics, and research data management. As a profession it is important that librarians embrace the concept of diversity in degrees and seek individuals who will enhance our instruction and learning.

Words Matter: Interpretations and Implications of "Para" in Paraprofessional and Paralibrarian Research

Hannah Schilperoort, Information Services Librarian, University of Southern California, Los Angeles, California

Frances R. Lezcano, Access Services Manager, University of Southern California, Los Angeles, California

Alvaro Quezada, Access Supervisor, University of Southern California, Los Angeles, California

Objectives: 1. To determine the identification term or terms (e.g. paraprofessional or library staff) preferred by non-librarian library staff.
2. To determine how library staff interpret terms, such as paraprofessional or library staff.
Methods: We conducted a literature review to determine terms used to refer to non-librarian library staff as a collective group. We identified terms such as library staff, library support staff, paraprofessional, paralibrarian, and nonprofessional. There are no recent studies attempting to identify the terms preferred by library staff. One 1991 ALA study indicated that library staff interpret "nonprofessional" as demeaning, and are divided on "paraprofessional," with some participants interpreting the latter as demeaning as well.
We created an online survey in Qualtrics with the purpose of identifying current terms preferred by library staff. We distributed via an anonymous link to medical and health sciences library listservs and social media outlets. The survey was approved by IRB review.
Survey participants include full- and part-time medical and health sciences library staff in any non-librarian position. We did not accept responses from librarians.
Results: Two hundred and twenty-two (222) people responded to the survey. Results indicate that the number one choice of participants is library staff, preferred over paraprofessional, paralibrarian, and library support staff. Thirty-three (33) of the 105 participants who completed the comments section specifically indicated that "para" in paraprofessional and paralibrarian is either demeaning, degrading, insulting, strange, or confusing, or had never heard of the terms before.
Results will be presented at the conference based on SPSS and Qualtrics analytical tools and reports.
Conclusions: We conclude that terms like "paraprofessional" should no longer be used to refer to non-librarian library staff in literature or by medical and health sciences library organizations, associations, and institutions.
Terms like "paraprofessional" and "paralibrarian" may have once made sense due to past library organizational structures, but these terms are now outdated. Library staff is more inclusive and less divisive, encompassing the wide range of non-librarian and librarian positions.

Tuesday, May 7, 4:30 p.m.–5:55 p.m.
Room: Columbus CD (East Tower, Ballroom/Gold Level)

Professionalism & Leadership Session 9

Moderator: Katie A. Prentice, AHIP

Using a Reflexive Process to Investigate Organizational Change: The Use of the Research Spider Matrix

Ardis Hanson, Assistant Director, Research and Education, Shimberg Health Sciences Library / University of South Florida, Tampa, Florida

Allison M. Howard, AHIP, Research and Education Librarian, Shimberg Health Sciences Library / University of South Florida, Tampa, Florida

Randall L. Polo, Research & Education Librarian, Shimberg Health Sciences Library / University of South Florida, Tampa, Florida

Krystal Bullers, AHIP, Emerging Technologies Librarian, Liaison to the College of Pharmacy / Assistant Librarian, Shimberg Health Sciences Library / University of South Florida, Tampa, Florida

Stephanie Tomlinson, Research and Education Librarian, Shimberg Health Sciences Library/ University of South Florida, Tampa, Florida

John Orriola, Asst. Librarian, University of South Florida, Tampa

Background: Health sciences librarians are being asked to provide more research services for faculty, including grant writing, project development, and systematic reviews. Being ready to engage in the research process may require significant changes to existing organizational cultures and work practices. These changes also require librarians to assess their readiness to provide research services. We describe a self-evaluation pilot study by U. S. academic health librarians (n=7) to assess their skills in preparation for the development of these services.
Description: With the advent of a new Assistant Director brought in to develop research services, the 'Research Spider' matrix (Smith et al., 2001) was used to start discussions on how librarians addressed their professional knowledge within the framework of research expertise. Librarians read the Smith article and independently evaluated themselves in each area (scale of 1 to 5; novice to expert). After meeting several times to discuss the definition of terms, objective scale criteria, and levels of competencies, librarians reassessed themselves using newly-revised benchmarks. The instrument showed face and construct validity, with concurrent validity confirmed by performance evaluations. Participation in this self-assessment exercise enhanced trust relationships among existing team members, helped integrate new faculty, determined necessary competencies and skills, and collectively defined minimal competencies for each of the Spider's legs.

Conclusion: The potential for change often is found in the internal dynamics of a group and in the contextual frames constructed by individuals. The Research Spider is a useful framework for understanding discrete characteristics of the research process. From an organizational perspective, the process provided pertinent data for individual and unit professional development goals, increased interpersonal and professional trust among the participants, and provided an innovative way to address new organizational challenges. Although self-assessment can be intimidating, the Research Spider broke the research process into manageable components, providing the librarians a way to identify competency levels, and inform future professional development.

Collaborative Marketing in a Health Sciences Library: A First-Year Assessment

Margaret Emily Ansell, AHIP, Nursing & Consumer Health Liaison Librarian, Health Science Center Libraries, University of Florida, Gainesville, Florida

Bonnie Sue Green, OPS Clerical Staff - Assoc. Dean/ Fackler Director's Office, Smather's Libraries in HSCL, University of Florida, Smather's Libraries, Gainesville, Florida

Joe Wu, Bioinformatics Support Specialist, University of Florida Health Science Center Libraries, Gainesville, Florida

Chloe Hough, Library Associate 1/ Evening Circulation Supervisor, University of Florida, Health Science Center Library, Gainesville, Florida

Background: In August 2017, an interdepartmental marketing team at an academic health science library instituted a new method of managing marketing through a marketing calendar system. At their quarterly meeting, team members developed a three-month schedule of marketing messages to be distributed via the library’s print and digital dissemination mechanisms, including signage, email, and social media. Messages were related to timely topics (beginning of the school year, finals, or holidays/health commemorations), as well as library-related events or services (instructional workshops, circulated items, or study spaces). After one year of conducting marketing efforts using this system, the team is assessing their efforts.
Description: The assessment includes both qualitative and quantitative data, and seeks to answer two core questions: 1) Is this method of marketing library services and resources effective in consistently engaging with potential/current library users, both to share about health information resources and services, and to gain feedback about those services? 2) Is this method of marketing library services efficient in creating a feasible and sustainable workflow for library staff and faculty? Data sources include social media statistics from Facebook and Twitter, attendance at library-sponsored events, survey data on library users’ experiences with/opinions of social media, focus group data from current and former marketing team members on the marketing calendar system, and survey data from library staff and faculty on the marketing calendar system.
Conclusion: The new marketing system increased user engagement with library social media accounts significantly, in part due to the hire of a social media coordinator for the university libraries, creating opportunities for cross-library promotion and engagement with university-level social media. The content of marketing messages has broadened to include services previously under-promoted, thanks to

circulation staff input, leading to the creation of new library signage. There were some challenges in recruiting, retaining, and training marketing team members; the team may need to formalize an orientation process for new members to learn the project management and design tools used by the team.

Beyond the Elevator: Improving Library Staff Communication Everywhere

Emily J. Glenn, Interim Associate Director, Education & Research Services, McGoogan Library of Medicine, Omaha, Nebraska

Heather L. Brown, Associate Director, Collection Services, University of Nebraska Medical Center, Omaha, Nebraska

Mary E. Helms, AHIP, Head, Strategic Initiatives, McGoogan Library, University of Nebraska Medical Center, Omaha, Nebraska

Emily McElroy, Director & Assistant Vice Chancellor for Academic Affairs, McGoogan Library of Medicine/ University of Nebraska Medical Center, Omaha, Nebraska

Background: Over the past four years, one academic health sciences library delivered a series of three staff development events aimed to facilitate better internal and external communication, partly in preparation for a major renovation. Idea generation, empowerment for advocacy, and audience awareness were key focus areas for development. We wanted to use semi-structured learning settings and facilitated exercises to provide common reference points for all staff. We aimed to build skills and increase staff confidence while honoring different learning and communication styles. Presenters will describe the three staff development events and demonstrate one key takeaway from each. We will share key outputs and impact and offer insight into the development of a library staff communication training plan aligned with long-term staff development and library objectives.
Description: The library's management team discussed strategies to promote improved communication among all staff. Previous training in change agent tools was useful for managers in meeting settings. However, we recognized a need to develop other skills as we entered a significant period of planning and disruption due to a library renovation. We identified gaps: all staff needed to shift toward "big picture" and advocacy-related thinking to address communication and group participation gaps. The first training event, a design thinking workshop, provided an environment to practice iterating on ideas using props and structured scenarios. Elevator speech creation and practice, the second event, helped staff fit content to short speeches that could communicate big picture ideas in 30 seconds or less. In the third training, a series of three facilitated sessions directed by a physician with a background in improvisational theater ("improv"), improv-inspired techniques were introduced to hone listening and general communication skills. Design thinking training supported idea generation and later strategic planning efforts. The elevator speech and improv training underscored the importance of audience awareness, listening, and advocacy. A staff survey and anecdotal observations from library leaders illuminate the impact of the training.
Conclusion: Modalities of the training worked well for some staff, but not others, which we attributed to differences in learning style, personality, and motivation. Not all staff participated in all training. Staff

turnover affected overall communication and participation. Library staff knowledge, skills, and confidence did improve in specific areas. One benefit from the trainings was the shared experience and related common language gained from participation. Reflections on these trainings will inform future communication skills exercises.

Informing Users and Ourselves: A Systematic and Holistic Approach to Communicating Resources and Services

Ricardo Andrade Jr., Assistant Director for Information Resources, Columbia University Medical Center Health Sciences Library, New York, New York

Eric Dillalogue, Access Services Manager, Health Sciences Library at Columbia University Irving Medical Center, New York, New York

Background: The program objective is to describe the creation and implementation of a communication working group that was charged with creating a campus-specific, sustainable, and systematic two-way communication process for library resources and services. The purpose of the program is to provide details on developing a communication plan, approaches in communicating to campus stakeholders, examples of messaging, and methods for receiving feedback. Attendees of the program will leave with information on how to create and implement a project plan, the findings of the working group, and details of the final communication plan that was developed.
Description: The Health Sciences Library lacked a process for effectively communicating to and getting feedback from the campus community. The communication working group, consisting of five library staff, was charged with developing a process that incorporated different approaches and methods for two-way communication about library resources and services. To develop a comprehensive project plan in a systematic way, we used a project management approach. This approach provided structure for our pilot projects, including those to: reach out to specific user populations, attend faculty meetings, utilize social media, and post messages on the library website and digital screens. Usage of resources, feedback from users, social media metrics, and questions received were used to evaluate the communication pilot projects. The final result was a plan that will be used for future communication efforts, including targeted and larger communications to the community.
Conclusion: Conclusions will be described at the time of presentation. The outcomes we expect to measure are:
1. Overall success metrics for the final communication plan
2. Effectiveness of specific communication approaches and methods
3. Usage statistics for selected resources and services
4. Social media engagement
5. Feedback from users
