# Supplementary material for: 119th Annual Meeting Medical Library Association, Inc. Chicago, IL May 3–8, 2019
Source: J Med Libr Assoc. 2020 Jan 1;108(1):E1–E17. doi: 10.5195/jmla.2020.897 (PMC6920001; doi:10.5195/jmla.2020.897)
Supplement: Appendix [file jmla-108-E1-s002.pdf]

Medical Library Association
MLA '19 Poster Abstracts

Abstracts for the poster sessions are reviewed by members of the Medical Library Association National Program Committee (NPC), and designated NPC members make the final selection of posters to be presented at the annual meeting.

Poster Number: 1

Time: Sunday, May 5, 3:30 p.m.–4:25 p.m.

Resuscitation, STAT! Evaluating and Updating an E-Book Collection

John Siegel, AHIP, Coordinator of Information Literacy; **Camille McCutcheon**, Coordinator of Collection Management and Administrative Services; University of South Carolina Upstate, Spartanburg, SC

Background: The hiring of a new nursing librarian and an impending integrated library system (ILS) migration provided an opportunity to examine the electronic book (e-book) collection at a metropolitan, regional comprehensive university with a bachelor of nursing (BSN) and master of nursing (MSN) degree program in clinical nurse leadership. The purpose of this project was to streamline e-book access and ensure the most current and relevant materials are available to nursing students and faculty. Objectives included determining review criteria, creating a procedure for suppressing titles, and improving collection development.

Description: The nursing librarian and Coordinator of Collection Management reviewed titles purchased through OVID and Rittenhouse (R2). Review criteria included age, number of uses, reviews (including Doody's Core Titles® and Choice Reviews), and scope based on course offerings. The Coordinator of Collection Management worked with the library's technical services team and vendors to establish a process for suppressing identified titles from the online catalog and platforms. Evaluation was based on informal feedback from users and reference librarians that assist patrons. As an added measure, e-book use statistics were examined.

Conclusion: Older editions of the same text were often available and patrons were using these instead of the most current version. Older editions were suppressed, therefore reducing confusion for users and increasing access to timely information. Reference librarians are better able to respond to inquiries and direct patrons to e-book titles. Popular titles, such as nursing care plans and drug guides, as well as specialized titles that were out-of-scope and had limited uses were identified. This will enable better informed purchasing decisions moving forward. This effort also resulted in a process that will be used with the library's other e-book vendors.

Poster Number: 2

Time: Sunday, May 5, 3:30 p.m.–4:25 p.m.

Operation Rescue: Resuscitating an Academic Print Nursing Collection

Camille McCutcheon, Coordinator of Collection Management and Administrative Services; **John Siegel, AHIP**, Coordinator of Information Literacy; University of South Carolina Upstate, Spartanburg, SC

Background: The hiring of a new nursing librarian and library renovations provided an opportunity to assess the print reference and circulating collections at a metropolitan, regional comprehensive university with a bachelor of nursing (BSN) and master of nursing (MSN) degree program in clinical nurse leadership. The collections had not been evaluated in 20 years. The purpose of this project was to review existing holdings and ensure that the most current and relevant materials are available to nursing students and faculty. Objectives included determining review criteria, identifying gaps in the collection, and improving collection management.

Description: The Coordinator of Collection Management and Administrative Services and the nursing librarian reviewed reference and circulating titles. Review criteria included age, condition, number of uses, reviews (including Doody's Core Titles® and Choice Reviews), and scope based on course offerings. Nursing faculty input was also obtained. Selected titles were withdrawn, and current editions of essential and other relevant titles were identified and purchased. Questionable as well as out-of-scope and highly specialized titles based on the curriculum were also identified. Evaluation was based on informal feedback from students and faculty as well as reference librarians who assist patrons. In addition, the currency and the relevance of the collection in relation to the curriculum – as well as space saved – were also factors.

Conclusion: In many cases, older editions of the same text were available, and these were often used or checked-out more frequently – which meant undiscerning students were often referring to outdated materials to potentially complete assignments and provide patient care during clinicals. Withdrawing these titles reduced confusion for students. Also, the Coordinator of Collection Management and Administrative Services and the nursing librarian were able to reduce the size of the collection – saving space and reducing the amount of shifting from one floor to another during renovations. This collection analysis will also inform future purchasing decisions which are critical in a time of limited budgets.

Poster Number: 3

Time: Monday, May 6, 3:30 p.m.–4:25 p.m.

#citeNLM2018: Partnering Together for Women's Health

Nisha Mody, Health & Life Sciences Librarian, UCLA, Los Angeles, CA; **Nora Franco**, Consumer Health Librarian, National Network of Libraries of Medicine Pacific Southwest Region, Los Angeles, CA

Background: In November 2018, each NNLM region hosted #citeNLM2018, focused on improving Wikipedia entries about women's health issues using NLM resources. Seizing the opportunity to promote collaboration and women's health, the NNLM PSR Consumer Health Librarian, who resides at UCLA, and a UCLA Health & Life Sciences Librarian co-organized an inaugural Wikipedia Edit-a-thon. They joined forces and extended the invite to all library staff, health sciences students, and gender studies students in order to produce an interprofessional, crowd sourced event. The librarians piloted this program design with the intention of inviting the broader UCLA community in the future.

Description: The librarians launched the project by creating a collaborative planning webpage which included project outcomes, information from the #citeNLM2018 Wikipedia page, sources for women's health, logistical information, and checklist items. The librarians proposed the concept of opening the event to the liaison librarians for the Schools of Medicine, Nursing, and Gender Studies. The liaisons were receptive to the idea and reached out to their connections, emailing invitations to the library staff, health sciences and gender studies departments. The librarians decided to pilot the event with only these four groups and use this experience as a guide for expansion in the future. They sent regular emails inviting all library staff to attend three NNLM-hosted webinars centered around introducing the project, how to navigate Wikipedia editing, and detailed descriptions of content within women's health resources.

Conclusion: The event encountered some setbacks including technical issues with the WebEx platform and low to moderate attendance (11 people). The attendees did find the event beneficial, and several commented they would like to see similar events in the future. This was the first Wikipedia Edit-a-thon for the majority of them, and they reported gaining new insight into the Wikipedia editing process and using it to improve women's health. By expanding outreach events outside of their typical partners and audiences, medical librarians promoted shared knowledge and an interdisciplinary approach, resulting in improved women's health information online using NLM resources.

Poster Number: 4

Time: Tuesday, May 7, 3:30 p.m.–4:25 p.m.

19th Century African American Female Physicians

Erica R. Powell, Librarian Assistant Professor, Metadata and Special Collections, Miami, FL

Objectives: To identify Black female physicians in the 1800s. What their struggles were and when and where they attended medical schools and set up their practices.

Methods: Retrieved the names of several female physicians from African American Studies Center, Black Studies Center, PubMed, Schomburg Center, databases of archival material from several states. I also searched women in science and medicine books including some specific about Blacks in medicine and science such as Contemporary Black Biography, NLM's Changing the Face of Medicine, Facts on File Encyclopedia Black Women in America Science, Health and Medicine, and biographies.

Results: Many resources had to be consulted, no one resource noted all the women. Women physicians regardless of race, need to noted in medical history. Obstacles seen then are still present today.

Conclusions: In highlighting a total of 19 women they are being shown as the first in their states awarded medical licenses or the first to be accepted in predominately Caucasian medical schools. These women need to be recognized and honored for their pioneering contributions to medicine. Women physician contributions were rarely noted and the information on minority women in medicine is even more scarce.

Poster Number: 5

Time: Sunday, May 5, 3:30 p.m.–4:25 p.m.

A #randomcoffee Controlled Trial: How Coffee Breaks Can Strengthen the Library Community

Kelly Johnson, Veterinary Outreach and Information Resources Librarian, Cornell University Flower-Sprecher Veterinary Library, Freeville, NY; **Katerina L. Stanton**, Access Services Supervisor, Cornell University, Ithaca, NY

Background: #randomcoffee, a program designed and implemented by Hootsuite, is meant to build relationships across an organization by randomly pairing colleagues for coffee dates. At large organizations, library staff often feel disconnected from their colleagues in other departments or units. Recognizing that a strong community supports a more satisfying and productive workplace, our institution initiated a #randomcoffee program in April 2018. Since implementation, we have witnessed growing popularity and wildly positive feedback. Here we outline our process, share success stories, and consider future improvements in order for others to envision a similar program at their own institutions.

Description: Each month, #randomcoffee participants' names are entered into an Excel spreadsheet for randomization. Pairs are introduced via email and encouraged to avoid drop-off by contacting their match immediately. Information is included to help participants prepare for and participate in the meet-up, including conversation starters and recommended time-allotments. A shareable sign-up link is included to prompt current participants to recruit co-workers. Within-unit and repeated pairings are manually corrected after consulting a spreadsheet of unit-affiliations and previous matches.

Conclusion: Program success is evaluated using a two-question Qualtrics survey that asks participants to comment on how or if #randomcoffee has affected their work, and to provide general feedback. The program has received 100% positive feedback from those participants who responded to the voluntary survey. Many respondents report the value of familiarizing themselves with other library units and extending their professional networks, and remark on the high benefit-to-effort design of the program. The most common comment/suggestion has been to continue #randomcoffee.

Poster Number: 6

Time: Monday, May 6, 3:30 p.m.–4:25 p.m.

A Bibliometric Analysis of Systematic Review Automation Tools

Elizabeth Moreton, Clinical Librarian, UNC Chapel Hill, Chapel Hill, NC; **Jennifer S. Walker**, Cancer Information Librarian / Liaison to the School of Dentistry, University of North Carolina at Chapel Hill, Chapel Hill, NC; **Fei Yu**, Health Informatics Librarian, University of North Carolina at Chapel Hill; **Michelle Cawley**, Head of Clinical, Academic, and Research Engagement, UNC Chapel Hill Health Sciences Library, Chapel Hill, NC; **Jesse Akman**; **Shannon Delaney**, Research Assistant, University of North Carolina at Chapel Hill, Chapel Hill, NC; **Adam Dodd**, Data Analyst, Health Technology and Informatics, University of North Carolina, Chapel Hill, Durham, NC

Objectives: To perform a bibliometric analysis on the body of literature discussing the use of automation tools to facilitate systematic reviews.

Methods: A comprehensive search was performed to identify articles describing the automation of systematic reviews using artificial intelligence (e.g. machine learning, text mining, algorithms, etc.). Searches were conducted in biomedical, library & information science, and engineering databases. Results were aggregated and de-duplicated. The number of results indicated that machine learning was an appropriate technique for reviewing such a large body of literature. A random sample of records was screened to identify relevant papers and create a training data set. After the machine learning process was employed to complete screening on the remaining records, final results were uploaded into a visualization & network mapping tool.

Results: Our bibliometric analysis found considerable collaboration around a core of prolific authors based in countries where evidence-based medicine has been widely adopted. Over time, representation in publications has moved from computer science journals and conference proceedings to domain-specific application with many newer enhancements focused on health sciences. Additionally, publications have moved from theory to problem-solving in specific parts of the systematic review process. Results will be delivered as a poster at the MLA 2019 Annual Meeting with visualizations including: size and scope of literature, key journals and conferences, collaboration networks, and author demographics including affiliation and role.

Conclusions: The field of systematic review automation has grown substantially in recent years, both in the addition of new authors and the expansion of collaboration networks. While adoption has increased in systematic review-focused publications and groups, more work must be done to gain acceptance in biomedical publications, including validations of algorithms and tools, guidelines for use in systematic reviews, and education of researchers. As key partners in systematic review performance and educators in systematic review methods, librarians are poised to play an important role in the adoption and acceptance of this technology.

Poster Number: 7

Time: Tuesday, May 7, 3:30 p.m.–4:25 p.m.

A Campus-Wide Collaboration: Digital Discovery Database

Patricia F. Anderson, Emerging Technologies Informationist, University of Michigan-Ann Arbor, Ann Arbor, MI; **Chase Masters**, Enabling Technologies Informationist, University of Michigan–Ann Arbor

Background: Over past decades, many groups have attempted to collect and share useful online tools both for their own re-discovery in the future as well as to create awareness for others on campus. The tools collected included technology and security resources, productivity tools, teaching and creativity resources, emerging social media platforms, online and mobile apps, and more. To the best of our knowledge, these efforts to collect and share resources have failed due to time investment required and issues of sustainability. Other challenges with these resources were duplication of effort across communities, duplication of content, and challenges of discovery across campus.

Description: We propose developing a collaborative crowd-sourced database with a simplified and streamlined entry form to allow individuals to contribute and share discovered content to facilitate open discovery both for the University of Michigan community as well as beyond.

The most unique and desirable features of the database will be an interface to facilitate easy, sustainable content-sharing and content-discovery by self-defined University of Michigan technology experts (campus geeks), utilizing best practices for crowdsourcing content.

Additional critical features to consider include: design for sustainability; community management, including oversight and responsibility; long term support; and a long term governance strategy. We attempt to include these considerations in the proposed design described.

Conclusion: We believe this resource would provide high value for the campus community, and would position the University of Michigan as an influential leader in the area of emerging technologies and collaboration tools. There is a need for this type of database at the university and the goal of this project is to cover all colleges, disciplines, units, and needs. There may be relevant issues or information needs which we have overlooked in this document, and we are open and eager to further conversations and collaborations to enrich and expand upon this framework.

Poster Number: 8

Time: Sunday, May 5, 3:30 p.m.–4:25 p.m.

A Feather in Our Research Cap: Implementing REDCap in the Library

Jackie K. Werner, Scholarly Communications and Research Librarian, Philadelphia College of Osteopathic Medicine, Philadelphia, PA; **P J Grier**, Associate Director, Philadelphia College of Osteopathic Medicine, Philadelphia, PA; **Meghan Di Rito**, Education and Outreach Librarian, PCOM Georgia, Suwanee, GA; **Skye Bickett, AHIP**, Assistant Director of Education and Engagement, PCOM Georgia, Suwanee, GA

Background: This poster presents the Library's experiences with all steps of planning and implementing REDCap. The Library works to facilitate and support research across all disciplines. One of the methods the Library does this is by implementing, promoting, and teaching REDCap: a tool for building and managing online surveys and databases.

Description: After several requests from researchers, the Library adopted REDCap in August 2017. Over the next thirteen months, librarians adapted and updated videos and tutorials from the University of Colorado Denver to fit the needs of our institution. Library staff created a REDCap LibGuide with best practices and FAQ's that also hosts the tutorials and videos. We did a soft launch for faculty who had previously used REDCap in December 2017 and who were willing to work as the Library's beta testers. REDCap officially launched at the institution in September 2018. Library staff continue to increase awareness and use of REDCap by offering support and assistance to REDCap users, as well as teach standalone and curriculum-based REDCap sessions.

Conclusion: Outcomes measured will include number of REDCap users at the institution, instruction session attendance, number of reference questions and consultations, and ongoing research using REDCap.

Poster Number: 9

Time: Monday, May 6, 3:30 p.m.–4:25 p.m.

A Health Sciences Library Empowering Community Health

Tiffany Grant, Assistant Director for Research and Informatics; **Don P. Jason, III**, Health Informationist; **Sharon Ann Purtee**, Cataloger; University of Cincinnati, Cincinnati, OH

Background: Despite improvements in medical care and efforts to make healthcare more affordable for all Americans, many still experience significant health disparities that result in poor health outcomes. Racial and ethnic minorities, those in rural and/or urban areas, and those living in medically underserved areas have a high risk of experiencing health-related disparities. We sought to increase health literacy among those most affected by health disparities by providing detailed, user-friendly health information, blood pressure monitoring, and instruction to assist families in learning more about nutrition in order to make healthier food choices.

Description: High blood pressure (HBP) is a leading cause of heart disease, which is a leading cause of death in our area. Providing accessible and easy-to-use health-related information is one way to help solve health disparities. With this in mind, we placed a blood pressure kiosk and a health information kiosk in physical locations that are trafficked by individuals from health disparity populations. We also provided a series of cooking demonstrations as a means to improve health outcomes through better nutrition. We collaborated extensively with our community partners to determine the most relevant health content and to assess ease of use of the kiosks. Use of the health information kiosk was limited initially due to a perceived technology barrier, however, a brief demonstration solved this issue. The blood pressure kiosk has had considerable use to date.

Conclusion: We are in the process of collecting kiosk data, and the cooking demonstrations are to be held at a later date. The information collected from the kiosks will yield great insight into the information seeking behavior of the targeted population. The results will be reported regularly to the community advisory board to assist them in devising a sustainable plan for community enrichment and in determining further health education programs that will be most impactful to the community. Anecdotal data received from our community partners has been overwhelmingly positive, suggesting that this program has been well received and utilized.

Poster Number: 10

Time: Tuesday, May 7, 3:30 p.m.–4:25 p.m.

A Hospital Library and an Ambulatory Care Pediatrics Department Collaborate to Elevate Patient Waiting Time to Patient Learning Time

Deborah Bonelli, Library Director, Medical Library, Bronx, NY; **Erenis Hidalgo**; **Samantha Moldonado**; **Amanda Nickles**; **Isabella H. Shattenkirk**, Project Assistant/Intern, Saint Barnabas Hospital Medical Library, Bronx, NY; **Ciara Walshe**; **Lauren Aronin**; **Anita Clarke**; **Paulo Pina**

Background: Generally, patient education in physician and clinic waiting areas is delivered via printed materials or videos. This poster describes a new educational approach: live presentations in the waiting room of a busy urban pediatric clinic. A pilot education program on medicine safety, completed in July 2017, inspired us to apply for an NNLM grant to fund this project. With NNLM grant funding, the pilot program PowerPoint presentation was expanded and revised to reflect information needs of low literacy, multicultural audiences. Pre-and post-presentation questionnaires given to parents/caregivers showed a marked increase in knowledge.

Description: College students enrolled in health education, pre-med, and pharmacy programs were recruited to revise the pilot presentation; create take-home cards and pre-post questionnaires; and to deliver the presentations to parents/caregivers in the clinic waiting room. The librarian recruited the students; introduced them to MedlinePlus; and prepared a syllabus on health literacy, plain English, and medicine safety. Supervised by the librarian and pediatric ambulatory care director, the student team created two medicine safety PowerPoint presentations: 1. Over-the-counter medications 2. Prescription drugs. The presentations covered how to read labels; proper dosing, administration and storage; emergencies; and MedlinePlus resources. The presentations, take-home cards, and pre-post questionnaires were reviewed for plain English. All materials were translated into Spanish.

Using the library's portable wireless Aquos Board, teams of two students gave the presentations in the clinic waiting room, which included hands-on demonstrations and audience participation.

Conclusion: Comparison of pre-post questionnaires showed a marked increase in knowledge. Parents/caregivers were appreciative of the presentations and asked for presentations on other topics. Several clinic physicians stated the presentations made them aware of the importance of using plain English when discussing prescriptions with parents/caregivers. Since this project, a team of medical students created a presentation on decreasing sugar intake. Focused live presentations on relevant topics, delivered by non-healthcare staff, can elevate waiting time to learning time; and the librarian can play a central role in achieving this.

Poster Number: 11

Time: Sunday, May 5, 3:30 p.m.–4:25 p.m.

A Multisite Collaboration to Improve Data Curation and Discovery in Academic Health Sciences Centers: The Data Catalog Collaboration Project

The Data Catalog Collaboration Project, Consortia, Data Catalog Collaboration Project, New York, NY

Background: Since late 2017, the Data Catalog Collaboration Project (DCCP) has grown to include eight institutions: NYU Langone Health, Duke University, University of Maryland Baltimore, University of North Carolina at Chapel Hill, University of Pittsburgh, University of Virginia, Wayne State University, and Zucker School of Medicine at Hofstra/Northwell. Each institution has installed or is exploring an open source data catalog to improve the discovery and reuse of (primarily) local datasets. The DCCP consists of librarians and developers who work collaboratively to address challenges related to improving metadata, curation, user functionality, and librarian-researcher engagement to address institutional data sharing needs.

Description: In 2018, DCCP institutions made inroads to establish workflows to facilitate indexing from a wide variety of research data sharing modes. Due to the diversity of each DCCP’s institutional environment, we established best practices for identifying and incorporating the breadth of data sharing use cases. Data sharing use cases include indexing supplementary data files in open access journals, queries made to institutional electronic health records for research, datasets purchased or licensed by our institutions, open source software generated by researchers, databases and resources created by researchers, and consortium-hosted research data. By maintaining flexible metadata and functionality, DCCP members use their data catalogs as a pathway for discovering institutional research data regardless of where it stored.

Conclusion: To date, more than 400 datasets have been indexed across the DCCP institutions’ data catalogs. These datasets represent the heterogeneous nature of data sharing use cases and the unique environments supported by DCCP institutions. Through the data catalog model and this inter-institutional collaborative effort, the DCCP has developed a standardized metadata schema and a single point of discovery for each institution to support data sharing.

Poster Number: 12

Time: Monday, May 6, 3:30 p.m.–4:25 p.m.

A Natural Fit: Expanding the Librarian's Role in Health Literacy and Patient Education

Melynda Ozan, Consumer Health Librarian, Levine Cancer Institute, Greensboro, NC

Background: [xxx] has a regional network of [xxx] cancer clinics in [xxx] Clinics in this network use their own patient education materials with few shared, system-wide materials. Many of these are written at an 11th-12th grade reading level and fail to meet health literacy and plain language standards. The selection of oncology materials from the network's patient education vendor is limited, so clinicians find themselves creating their own materials or pulling them from multiple sources of varying quality and readability. In order to address these issues, a new Consumer Health Librarian position was created.

Description: This poster will describe the duties and responsibilities of this new librarian position, along with an outline of skills from other areas of health sciences and medical librarianship that are transferable to this type of work. It will also discuss the challenges and rewards that come with this role. The goal is to encourage librarians to think outside the box and explore unconventional roles in healthcare.

Conclusion: Librarians have a unique set of skills that can be easily applied to health literacy and patient education programs. These types of initiatives offer librarians the opportunity to step out of traditional library-based roles and bring their expertise to a new forum.

Poster Number: 13

Time: Tuesday, May 7, 3:30 p.m.–4:25 p.m.

A New Way to Look at Old Bones: Launching a Virtual Reality Service at a Health Sciences Library

Tariq Rahaman, Emerging Technologies & Collection Development Librarian, Martin & Gail Press Health Professions Division Library, Fort Lauderdale, FL

Background: This project's primary objective is to establish a sustainable and scalable virtual reality (VR) library service with a focus on virtual anatomy and medical simulation. A secondary objective is to develop inter-professional collaboration within our institution and support VR projects on medical/health sciences education and research initiatives.

Description: This initiative stemmed from an exploration of digital anatomy resources; VR was favored for its three-dimensional, immersive, and gamified approach to educational content. This presentation describes our process of launching and integrating VR as a new resource, including our decisions on hardware and software, staffing/workflow, policy and safety guidelines, outreach and programming, as well as use cases for VR technology in a health sciences library beyond anatomy resources.

Conclusion: The VR Lab has been successful in offering unique, virtual experiences that are academically relevant to our students, faculty and staff. As we continue to evaluate and demo applications, the VR equipment has been utilized for curricular activities in the case of anatomy simulation, and co-curricular activities in the case of self-study. Future projects will include surveys to validate apps, and increased outreach and programming to our students, faculty and staff.

Poster Number: 14

Time: Sunday, May 5, 3:30 p.m.–4:25 p.m.

Ab Errantry: Librarian Roles in Developing a Video Game around Online Safety Skills for At Risk Individuals

Patricia F. Anderson, Emerging Technologies Informationist, University of Michigan-Ann Arbor, Ann Arbor, MI; **Bruce R. Maxim**, Professor CIS, University of Michigan-Dearborn, Dearborn, MI

Objectives: Extending classic roles of librarians in bibliographic instruction and internet safety, this project describes a collaboration between librarians and game developers to create a resource for building online safety skills in at-risk individuals. The initial target audience was teens and young adults with autism. Library roles included searching, community building, theme extraction, project management, hosting play testing, and licensing negotiations.

Methods: The game was developed in Unity3D as a 2D side scroller with a medieval-themed fantasy storyline. Game mechanics were selected to target common behaviors of online sexual predators, including social grooming, inappropriate trust building, and social distancing from previously trusted communities. Dialog scripting was developed, in part, to model language extracted from linguistic patterns of actual predators via text mining, and to further the game storyline. The game concept and design were developed in collaboration with a person with autism. The scripting and game mechanics were developed in conjunction with a consultant in online sexual predators. The game was designed to be enjoyable for a broad audience, not just the original target audience, with the underlying purpose presented as subtext, with the specific lessons to remember brought out in a post-game "debrief" that is made available to the player.

Results: The game was tested with diverse communities representing middle school children, college students, and persons with a wide variety of physical and cognitive disabilities. Middle school testing was performed more in large groups, with quantitative results, while the play-testing for college students and persons with disabilities was done in more of a focus group approach, with qualitative results. Gender balance of testers was close to equal. Game play testing addressed both design and comprehension. A strong majority of the play-testers not only expressed willingness to replay the game, but wanted notification when the game becomes officially available.

Conclusions: Challenges of the project centered around community and access to art assets. It is hoped that open-source licensing will ameliorate the latter for others. All game assets have been made available in GitHub with either open-source or Creative Commons licenses. Playable versions of the game are available free online for multiple platforms in order to facilitate broad access. The library is partnering with rehabilitation and psychosocial professionals at our institution on further testing and development. The project was prominently profiled in campus media and news reports, building reputation and collaboration opportunities.

Poster Number: 15

Time: Monday, May 6, 3:30 p.m.–4:25 p.m.

Accessible Impact: Introducing an Asynchronous Impact Evaluation Mini Workshop

Erin Anthony, Public Health and Research Support Librarian, Brown University, Providence , RI; **Kelsey Sawyer**, Biomedical and Life Sciences Librarian, Brown University, Providence, RI

Background: In the spring of 2018, Brown University's library began to consciously strengthen its involvement in scholarly research impact evaluation. Previously, the library had provided some online materials on impact evaluation, but these were disparate and not widely shared. The increased role of impact metrics in departmental evaluations, award applications, and grant reporting presented a new avenue of service that the library could address. A new/expanded library staff provided an opportunity to expand and showcase the reach of Brown's research community. An online, asynchronous workshop, delivered via email, was created in order to reach a wide audience within the University community.

Description: We developed a week-long series of concrete tasks designed to increase individual scholarly impact as well as understanding of research impact metrics. Participants received a daily email with one or two tasks, such as registering an ORCiD or setting citation alerts in Web of Science.

We also created a complementary LibGuide to centralize information on scholarly impact evaluation and profile management. This included information on common metrics used to evaluate both individual- and journal- level impact, tools for disambiguating identity, and techniques for tending to scholarly profiles.

The "Research Impact Challenge" was piloted internally and released to the wider Brown community in July, 2018. Thirty-three participants registered. In October, the challenge attracted 30 participants. For each challenge we administered a Qualtrics survey to gather feedback. Overwhelmingly, the feedback has been positive and indicates people were previously unaware of the resources.

Conclusion: This project increases awareness and use of impact, evaluation and publishing tools, highlights the library's expertise, and provides a context for future support. We strive to promote open access and institutional publishing practices for greater access of research by the community. The challenge could increase community discussions of research.

Since the introduction of the workshop, we have presented at the Transforming Research conference and as a webinar for ACRL. Many librarians are now hosting the challenge in their own institutions.

The idea was based on an event at Duquesne University and Stacy Konkiel's book The 30-Day Impact Challenge.

Poster Number: 16

Time: Sunday, May 5, 3:30 p.m.–4:25 p.m.

Adulting 101: Elevating the Health Literacy Skills of Undergraduates

Megan N. Fratta, Community Outreach and Global Health Librarian, Health Sciences Library, University of North Carolina at Chapel Hill, Chapel Hill, NC; **Caitlin Kennedy**, Carolina Academic Library Associate, Robert B. House Undergraduate Library, University of North Carolina at Chapel Hill, Carrboro, NC

Background: In summer 2018, librarians at the undergraduate library reached out to the health sciences library with a proposal to develop an Adulting 101 session on health literacy for undergraduate students. In keeping with the university's mission to support the whole student and prepare them for participating in a democratic and global society, the series introduces students to health, financial, and civic literacy concepts and skills, using an active learning and information literacy-based approach. Specific objectives of the health literacy session included the introduction of trustworthy sources of health information and the development of skills to evaluate health information found online.

Description: Marketing for the series drew upon the popularity of the term "adulting" and focused on the opportunity to learn practical skills that will help students succeed in college, career, and life. Topics for the first workshop series, piloted in fall 2018, included evaluating online health information, creating a budget, and researching local political issues and candidates. In collaboration with staff from the undergraduate library, the librarian developed and led the health literacy workshop, "Is There a Doctor in the House? Your Prescription for Finding Quality Health Information." The librarian created a companion LibGuide containing reliable sources of information and in-class activities. Participants were given a standard three-question assessment at the end of each workshop focusing on what they learned and what could be improved.

Conclusion: The fall 2018 health literacy workshop was attended by a mixture of fifteen undergraduate and graduate students, which demonstrates the broad appeal of the Adulting 101 series and health-related content. As a result of positive feedback in post-workshop assessments, the libraries planned a second round of Adulting 101 content for the Spring semester. The librarian planned and led "Self-Care for the Mind: Researching Mental Health" in collaboration with campus partners including counseling services and a student-led mental health advocacy organization. Eighteen students attended the second workshop. Additional health literacy workshop ideas and marketing strategies will be explored for future semesters.

Poster Number: 17

Time: Tuesday, May 7, 3:30 p.m.–4:25 p.m.

Adopting a Rare and Historical Monograph Collection

Brittany L. Haliani, Manager, SAGE Medical Library, Cottage Health, Santa Barbara, CA; **Jessica Reeves**

Background: The SAGE Medical Library archive collection contains 534 titles with the oldest dating back to 1845. The collection was started by a donation from the Santa Barbara County Medical Society Library. The donation was to the former Reeves Medical Library which is now SAGE Medical Library. At the beginning of 2018 the library staff initiated an archival project to catalog the collection as well as, determine the value of the collection. In addition, the collection in now located in climate controlled, closed shelving in a start of the art new library located in the main hospital.

Description: The project was developed by reviewing all historical books. The review was documented in an excel file which was used to catalog the books into our library management system. In addition, the excel file was used to document the value of the books. This was done by using the Website AbeBooks.com. Furthermore, library staff used other historical, medical book collections available online to assist in cataloging and referencing value. If books were deemed to have a large monetary value, they would be placed in a protective box and flagged in our library system.

Conclusion: After the completion of the project our collection was valued at $40,000.

Poster Number: 18

Time: Monday, May 6, 3:30 p.m.–4:25 p.m.

All of Us Research Program and Your Health Educational Events at Sam Houston State University

Lisa Connor, Assistant Professor Research & Instruction Librarian Health Sciences, Sam Houston State University, Huntsville, TX

Background: As a new Research and Instruction Librarian supporting a School of Nursing (SON) I scanned the American Association of Colleges of Nurses (AACN) website to better understand the needs of my nursing faculty. I identified an opportunity to apply for a mini-grant award to facilitate the engagement of the academic nursing community with the All of Us Research Program. Having been aware of this program from prior work and from MLA '18 presentations I forwarded the announcement to the Director of SON. She shared the information with faculty that led to a collaborative effort to win the grant!

Description: The AACN min-grant supported by the NIH clearly specified a need to reach historically underrepresented communities in biomedical research communities. We collaborated and concluded that our own campus was a highly desirable to reach because of our diversity. Faculty from the School of Nursing and I developed and submitted the application in about a week and were chosen one of nine nursing schools awarded! We networked throughout our university system to identify points of contact with student organizations. We identified venues to accommodate three educational events. We hired two nursing students to prepare invitations and to monitor response to attendance at our events. We prepared promotional material and held promotional events on campus to inform about the event. I created a library display about the event and precision medicine. I networked with NNLM to inform Public librarians in surrounding communities.

Conclusion: The program has not concluded. Our educational events will be January 28, and January 29, 2019. I expect to measure the number of participants we were able to draw to attend. I also expect to comment on the diversity of attendees and meeting the mini-grant objectives of reaching historically underrepresented communities in biomedical research. I would also plan to report on lessons learned in engaging within the university environment as well as the surrounding communities in information about the All of Us Research Program.

Poster Number: 19

Time: Tuesday, May 7, 3:30 p.m.–4:25 p.m.

Analyzing Application Data of Rapid Interlibrary Loan Service to Evaluate Journal Collection: A Case Study of the Taipei Medical University Library

Cheng-yi Lin, Librarian, Taipei Medical University Library, Taipei, Taipei, Taiwan (Republic of China); **Tzu-heng Chiu**, University Librarian, Taipei Medical University, Taipei, Taipei, Taiwan (Republic of China)

Objectives: The Taipei Medical University Library has been provided Rapid Interlibrary Loan (Rapid ILL) service since July 2013. This study intends to analyze the application data of the Rapid ILL to explore user ' s demand on academic journals. Furthermore, findings of this study can benefit to evaluate selection rules of recommended journals and adjust collection development policy of the TMUL.

Methods: This study discusses demand and satisfaction of academic journals in TMU in order to improve the selection rules of recommended journals, promote the Interlibrary Loan service and enhance collection development policy. The authors analyze Rapid ILL application records of the TMUL from July 2013 to December 2017 and got a total of 9,881 valid records. In addition, we collected journal recommendation lists of every department of TMU and journal subscription records of the library from 2014 to 2017 to compare with the Rapid ILL application data, including the journal title, ISSN, article publication year, affiliated department of applicant… etc. We believe that the journal titles applied in the Rapid ILL service represent real information needs of our users, it reflects the insufficient of our journal collection. The most requested titles will be provided to the department liaison for reference.

Results:Among the 9,881 requested Rapid ILL records, the top 10 most requested journals are listed in Table 1. 92.86% of the journals were requested less than 10 times. And articles published within past 1-5 years had highest demand. (see Table 2). As to the amount of application (see Chart 1), College of Nursing ranked the first (32%), followed by College of Pharmacy (16%) and College of Oral Medicine (14%). In addition, College of Nursing's journal demand of the Rapid ILL services was much more corresponded with its journal wish lists than other colleges.

Conclusions: Due to the flat budget of the TMUL and the increasing prices of the academic journals, we can only subscribe 61.6% of recommended journal titles in 2017. (However, it was 75.8% in 2014) As a result, the Interlibrary Loan service plays an important role to fulfill the information and literature need of our faculty and students. The TMUL will promote the service to everyone in the campus, especially to colleges which rarely used the Rapid ILL. Moreover, the findings of this case study has been applied to our 2019 periodical subscription.

Poster Number: 20

Time: Sunday, May 5, 3:30 p.m.–4:25 p.m.

Archival Cataloging, Quickly and Efficiently

Catisha Benjamin, Manager of Library Services, MLIS, Children's Hospital Colorado, Aurora, CO; **Marie St. Pierre, AHIP**, Medical Librarian, Childrens Hospital Colorado, Aurora, CO; **Anna Maria Andrews**, Librarian, Volunteer, Children's Hospital Colorado, Aurora, CO

Background: The Clinical and Research Library of Children's Hospital Colorado oversees the care of the hospital's archives. A survey of the contents and a brief shelf list had been created to have an idea of the materials stored there, and now the librarians wanted to create a full catalog. OCLC's World Share, used in the library, has a basic template for books, video, photographic images and similar. Cataloging can be done in a MARC format template or text templates. The text template was chosen as convenient and appropriate to most of the formats of the items in the archive.

Description: The librarians and volunteers chose the books and photographic materials to catalog first, as they had the easiest template and many of the books could simply be copy cataloged. There were a number of written works that needed original records, such as the Hospital's annual reports dating back to the 1900s. The photographs include employee photos as well as promotional photos. These have been completed, and now other items, such as documents, videos or films and ephemera will be next. Choosing standard titles for the hospital as a 'corporate author", and using pre-established subject headings (when available) helped. Keeping to a set script for the acquisition part of the process made it much quicker. Ephemera will be the most difficult as World Share does not include a template for 'objects'.

Conclusion: In a brief time, a good portion of the archival books and photographs have been added to the library's card catalog, and work is continuing on the rest of the collection. This will be helpful in listing all of the items so that they will be searchable and accessible, as appropriate. Additional thought will be given to the utilization of templates, such as for art or visual materials, thus enabling the cataloging of preserved memorabilia.

Poster Number: 21

Time: Monday, May 6, 3:30 p.m.–4:25 p.m.

Are Resource Sharing Statistics a Reliable Metric to Determine the Impact of Journal Cancellation?

Lindsay Barnett, Collection Development & Scholarly Communication Librarian, Yale University, Branford, CT; **Alyssa Grimshaw**, Access Services/Clinical Librarian, Cushing/Whitney Medical Library, West Haven, CT; **Melanie J. Norton**, Head of Access and Delivery Services, Cushing/Whitney Medical Library Yale University, New Haven, CT

Objectives: The researchers hope to determine if interlibrary loan borrowing statistics after the cancellation of a subscribed journal reflected the same level of use compared to when the journal was active in our collection.

Methods: The researchers gathered Interlibrary Loan (ILL) borrowing statistics for a control list of titles cancelled in fiscal year 2017/2018 and analyzed them against COUNTER JR1 statistics for the same period of time to determine if interest in a journal remained consistent post-cancellation.

Results: Preliminary results indicate that ILL borrowing statics are significantly lower than anticipated based on documented usage while the journal was part of the collection. These results indicate that potential users are not taking the additional steps to obtain articles through ILL that the library no longer subscribes to.

Conclusions: Statistical data with no context cannot reliably capture user behavior and librarians should consider the incomplete nature of these metrics when anticipating user need.

Poster Number: 22

Time: Tuesday, May 7, 3:30 p.m.–4:25 p.m.

Assessing Data-Related Practices among Psychology Researchers: Study Design and Preliminary Results

John Alexander Borghi, Data Services Librarian, Stanford Medicine, Stanford, CA; **Ana Van Gulick**, Liaison to Psychology & Brain Sciences, Program Director for Open Science, Carnegie Mellon University, Pittsburgh, PA

Objectives: Though the field of psychology has begun to embrace open science practices, information about how researchers are currently managing and sharing their data remains largely anecdotal. The objective of this study is to survey the practices and perceptions of active psychology researchers in order to inform the development of data-related standards and best practices.

Methods: Building off of our previous work examining data-related practices in neuroimaging research, we have developed an online survey that includes 64 multiple choice questions for psychology researchers about their practices throughout the course of a research project. Topics include the type of data they collect, the software tools they use, how manage their data, factors that limit and motivate their current practices, and their perceived need for additional training.

In order to characterize data-related practices across the various subdomains of psychology (e.g. clinical, biological, and social psychology), we plan to distribute our survey through a variety of means including direct e-mailing authors who have published psychology research articles in the last year. In order to demonstrate best practices related to data management and sharing, both the resulting dataset and the code used for its analysis will be made openly available.

Poster Number: 23

Time: Sunday, May 5, 3:30 p.m.–4:25 p.m.

Assessing the Quality of a Triage Reference Services Model at an Academic Library: The Role of Reference Assistants

Mariana Lapidus, Reference Coordinator, MCPHS University, Boston, MA; **Irena G. Dryankova-Bond**, Library Manager/Associate Prof. of Library and Learning resources, Massachusetts College of Pharmacy &, Worcester, MA; **Samuel B. King**, Manager, Manchester Campus Library, MCPHS University, Manchester, NH; **Erin Wentz**, Electronic Resources Librarian, MCPHS University, Boston, MA; **Susan S. Mahnken**, Interlibrary Loan & copyright Services Librarian, MCPHS University, Boston, MA; **Gregory Anthony Martin**, Administrative Assistant, MCPHS University, Boston, MA

Objectives: To assess the overall effectiveness of the triage reference services model utilized by the three-campus University Libraries based on the questions complexity, answer correctness, and patron interactions.

To measure the performance of academic library paraprofessional assistants, whose primary role at the reference desk is answering simple directional and research questions and referring patrons with complex inquiries to subject liaison librarians.

Methods: Live chat transactions from Fall 2017 and Fall 2018 semesters, tracked and saved on the Springshare LibAnswers Reference Analytics platform, have been selected for quantitative analysis. Chat questions answered by the professional librarians are excluded from the evaluated transcripts, and the ones completed by the reference assistants are transferred to an in-house designed Access database. Every chat transaction from this data set is then rated by a designated group of professional librarians based on:

• The ALA Reference & User Services Association (RUSA) recommended variables used to measure question- answering performance.

• The answer correctness and patron interaction evaluation criteria table, designed by the librarians as the result of an extensive literature search that allowed to identify the most appropriate indicators adopted by other academic institutions.

• The Reference Service Assessment Data (READ) scale utilized to measure the questions complexity.

Results: Based on the two semesters' transcripts evaluation, the overall positive findings were revealed. On the average, the number of questions answered by reference assistants accounts for 70% of all live chat inquiries. Most of the questions answered by paraprofessional staff appeared to be directional inquiries and/or basic research/minimal instruction requests (levels 1-3 according to the READ scale). The average answer correctness tended to be 2 (partially correct) or 3 (correct). Similarly, patron interaction tended to be 2 (good) or 3 (exemplary) rather than just 1 (acceptable). Some gaps in reference assistants' knowledge and topics for their further training were identified.

Conclusions: This study provided essential statistical data in support of the triage reference services model, currently adopted by the University Libraries. The results of live chat transcripts evaluation clearly demonstrate that, with proper training, reference assistants can provide quality reference services for patrons and are an essential part of today's academic libraries' workforce.

Poster Number: 24

Time: Monday, May 6, 3:30 p.m.–4:25 p.m.

Assessment and Comparison of Online Exam Preparation Resources Available at Academic Health Sciences Libraries

Sarah Towner Wright, Clinical Librarian and Graduate Medical Education Specialist, UNC-Chapel Hill, Chapel Hill, NC; **Karen Stanley Grigg**, Health Sciences Librarian for Collections and Instruction, UNC Chapel Hill Health Sciences Library, Chapel Hill, NC

Background: To evaluate and compare the features, performance, ease-of-use, and currency of a variety of online exam preparation resources available for health sciences examinations, and to explore the features unique to each resource.

Description: Providing online access to the numerous examination preparation resources necessary to students and health professionals for school admittance and health profession certification is a complex and time-consuming task for health sciences librarians. Along with keeping track of the broad spectrum of examinations, it is important to stay current with existing and emerging examination preparation products available for institutional subscription. Collection development librarians need to evaluate and compare these resources, and liaisons must be competent in the promotion and training of these tools. To that end, the Collections and Instruction Librarian and the Clinical Librarian at an academic health sciences library collaborated on a project to identify and compare examination preparation products currently available. A comparison chart was created that compiled the following aspects for each resource reviewed: available content, currency of exam question banks, mobile access, ease of navigation, and strengths and weaknesses.

Conclusion: The creation of an exam preparation resource comparison chart, compiled by a collection development and a liaison librarian at an academic health sciences library, serves as a guide that librarians can use to determine the following: online exam preparation resources that currently are available, examination-specific question bank products that are provided, and an assessment of the strengths and weaknesses in comparison to competing products. This guide will help collection and liaison librarians provide access to the exam prep resources that best fit the needs and budget of academic health sciences libraries.

Poster Number: 25

Time: Tuesday, May 7, 3:30 p.m.–4:25 p.m.

"Before We Chat...": Creating an Online Tutorial Reviewing the Components of a Systematic Review

Michelle B. Bass, AHIP, Manager, Research and Instruction, Harvard Medical School, Countway Library of Medicine, Boston, MA; **Katie Ryan Stinson**, Library Specialist, Stanford University, Stanford, CA

Background: Lane librarians' initial one-on-one consultations for those interested in doing a systematic review consisted of outlining the multi-step, team process of a systematic review repetitively. We only work the day shift and our time is valuable, as is that of our researchers. We wanted to use our time more effectively and provide access to information for our users at any time to reference whether or not they were preparing to do a systematic review. The library decided to investigate online platforms for accessing information about the pieces of a systematic review.

Description: We began with a trial of a vendor-created online course which comprehensively covered the multi-step processes of an intervention-based systematic review. Feedback from our community was critical of the amount of time needed to complete all aspects of the online course. Lane librarians were concerned about the lack of discussion regarding the role and time commitment of the librarian involved in a systematic review. We decided to create a brief self-paced online tutorial for our community which highlighted the pieces of a systematic review, the role of librarian, and the time involved to do a systematic review by the definitions and guidelines provided by research networks. We used the work of those before us to scope our content and utilized the OpenEdX platform so that it could be easily accessed by all users of our Stanford Medicine community and beyond.

Conclusion: Between August 27, 2018 and January 23, 2019, we have had 582 participants enrolled in the freely available online course. While only 3% are affiliated with Stanford as determined by their use of a Stanford email account, we have not yet done an official launch of the course but have been sharing its location with our communities in our instruction sessions during the fall quarter. we are very happy to have a self-paced on-demand option for our community members to access.

Poster Number: 26

Time: Sunday, May 5, 3:30 p.m.–4:25 p.m.

Bringing (Short) Literature to the Health Sciences Community: Launch of a Short Story Dispenser in a Health Sciences Library

Matthew Nicholas Noe, Collection Outreach Librarian, Harvard Medical School, Boston, MA; **Len Levin, AHIP**, Associate Director of Research, Instruction and Collection Resources, Harvard Medical School, Francis A. Countway Library of Medicine, Boston, MA

Background: As part of ongoing efforts by the library to support the Medical School Arts and Humanities Initiative, we have begun a partnership with French publishing house, Short Édition, to add a Short Story Dispenser to the library. The dispenser allows us to offer 1, 3, and 5 minute short literature to the community at the push of a button. The short nature of the readings are a perfect fit for our time-crunched patrons.

Description: The development and launch of what we're calling the Stories program is ongoing and consists of different phases. The first phase involved the purchase, delivery, and initial set-up of the Dispenser, including a two-week period of staff testing and minimal community use. This step allowed us to ensure proper configuration of both Dispenser and online dashboard. Phase two was the official launch of the Dispenser, which included regular announcements through social media, email, and word-of-mouth outreach; these are continuing efforts. During this phase, various locations throughout the library's 1st floor were tested to determine the best location for visibility and use. Phase three is currently in progress and involves working with community partners to develop opportunities for the medical school community to create, submit, and share their own short works of literature through the Dispenser.

Conclusion: Evaluation of the Dispenser's has been multifaceted. During the initial launch, a feedback survey was included in the footer of each dispensed story and provided an opportunity for direct community input. Immediate, thorough, and ongoing usage statistics are collected by the Dispenser itself and available for real-time viewing. These statistics informed and continue to inform the location and marketing methods for the Dispenser. As opportunities for community authored stories become a reality, we will evaluate interest, submission rates, and story quality, in addition to keeping record of any stories that Short Édition seeks to license for broader use.

Poster Number: 27

Time: Monday, May 6, 3:30 p.m.–4:25 p.m.

Building a Framework to Elevate Residents' Accreditation Council for Graduate Medical Education (ACGME) Required Scholarly Activities

Lindsay E. Blake, AHIP, Clinical Services Librarian, University of Arkansas for the Medical Sciences, Little Rock, AR; **Jennie Kirby**, Program Manager, College of Medicine Graduate Medical Education; **Jennifer White**, Program Manager, UAMS, Little Rock, AR

Background: The Accreditation Council for Graduate Medical Education (ACGME) scholarly activity requirement is a thorn in the side of every residency program. The aims of the scholarly activity are to introduce residents to the conduct, evaluation and presentation of research. Each residency finds ways to assist their residents with this requirement, but a central database to assist residents university-wide is not available. With the idea of creating one university-wide scholarly activity guide, the Graduate Medical Education (GME) office approached the Clinical Services Librarian. Discussion led to the idea of building an online resource focusing on publication, presentation, and research.

Description: Meetings were held with educational representatives from the largest residencies to discuss how the scholarly activity requirement was currently being met and the needs of each residency. The librarian collected resources from each residency and collated them with her own resources, then built an online guide using the LibGuides software. The Research and Scholarly Activity Guide combines practical step by step advice, tips and tricks, guidelines, and other resources for completing projects. Sections include information on: how to design a poster and where to get it printed, how to write an article abstract, and how to submit a case report to a journal, among others. The Research and Scholarly Activity Guide was completed mid-2018 and was introduced to new interns, residency directors, residency coordinators, and returning residents beginning in July.

Conclusion: The Research and Scholarly Activity Guide brings a number of disparate resources together easing residents' road to completion of their scholarly activity requirement as well as assisting junior faculty with research endeavors. Residency directors, residency coordinators, residents, and even students have all expressed how helpful the guide has been for them. Since its release, the guide has received over 1,700 total views. The majority of these being in case reports, poster presentations, and literature searching.

Poster Number: 28

Time: Tuesday, May 7, 3:30 p.m.–4:25 p.m.

Building Capacity in Nigeria to Promote Implementation Health Sciences: A Workshop Experience

Xan Y. Goodman, AHIP, Health Sciences Librarian, Lied Library, Henderson, NV

Background: This program aimed to build research capacity among Nigeria Implementation Science Alliance (NISA) networked agency health care practitioners to enhance research skill sets. With a chief aim to increase capacity to write systematic reviews and apply for grants to improve health care. Training on HINARI resources sought to equip researchers with skills to better utilize databases and apply evidence-based research to public health and implementation science issues. This program was conducted as part of the 2018 NISA conference program in Enugu and Abuja, Nigeria with two two-day facilitated pre and post-conference workshops.

Description: Two two-day workshops were developed in coordination with master HINARI trainer Lenny Rhine with supplemental content designed by facilitators. The process involved designing learning outcomes and active workshop exercises including formative and summative assessment questions. Program evaluation is ongoing, a pre-knowledge survey was completed, and a post-conference assessment was administered in April 2019. Post survey results immediately following the workshops are positive, “The workshop was very useful in refining my searching skills.” “The workshop was helpful and [I] learnet skills to help me with my personal research among others.” Research groups were formed during the workshop. Both groups have submitted their systematic review protocols to PROSPERO. A summative assessment shows seventy-one percent of participants are working on at least one SR.

Conclusion: Outcomes: number of SRs completed - 0; Grants completed - 0; Increased confidence and ability to use HINARI resources to support systematic reviews or grants-53% have trained others to use HINARI resources; At least two participants are engaged in other research using resources and skills learned during the workshop.

Poster Number: 29

Time: Sunday, May 5, 3:30 p.m.–4:25 p.m.

Burnout among Medical and Health Sciences Information Professionals Supporting Systematic Review Research: An Exploratory Study

Michelle Demetres, Scholarly Communications Librarian, Weill Cornell Medical College, New York, NY; **Drew Wright**, Research Librarian, Weill Cornell Medical Library, Sunnyside, NY; **Antonio P. DeRosa, AHIP**, Oncology Consumer Health Librarian, Weill Cornell Medicine, New York, NY

Objectives: The aim of this exploratory study is to address the gap in the literature related to burnout among information professionals who support systematic review (SR) research.

Methods: The Copenhagen Burnout Inventory (CBI), a validated tool for assessing burnout, was sent to information professionals supporting SR research. A broad range of health sciences/medical librarians and information professionals were targeted via professional listservs and news outlets. Questionnaire responses were captured electronically using Qualtrics Survey Software and quantitative analysis was performed.

Results: Respondents experienced an average personal burnout score of 48.6, work-related score of 46.4, and client-related score of 32.5. Those who reported spending >80% of their job duties on SR work saw significantly lower total average burnout (28.6) than those who devoted less time to SR work (between 42 and 45). They also averaged much lower personal burnout (31.5) and client-related (19.4). For complete data set, please visit: https://ecommons.cornell.edu/handle/1813/60390

Conclusions: The data suggest that with dedicated time solely for SR work, there is less burnout. When job duties are varied outside just SR work, with less time to devote to a heavy burden, there appears to be slightly higher burnout. This study provides groundwork for further investigation with the aim of potentially developing approaches to combat burnout in this professional population.

Poster Number: 30

Time: Monday, May 6, 3:30 p.m.–4:25 p.m.

Cannabis and Essential Oils: Developing Undergraduate Researchers through an Evidence-Based Practice Course

Jessica Sender, AHIP, Liaison to the College of Nursing, Michigan State University, Lansing, MI

Background: Nursing 375: Research and Evidence-Based Practice is an undergraduate research course with a heavy emphasis on the process of defining a research (PICOT) question, developing keywords, creating a search strategy, and evaluating search results. As undergraduates, this process can be overwhelming. This poster outlines the collaboration between library and professor to create a scaffolded instruction program, including the librarian teaching five times over the course of the semester, providing feedback on submitted assignment through the course management system, and evaluating final projects, all in order to develop undergraduate researchers.

Description: Nursing 375: Research and Evidence-Based Practice is a course in the undergraduate nursing curriculum that has undergone several revisions as the College of Nursing has refocused its direction towards more research and enhanced evidenced-based practice education. The semester-long group project requires students to develop a PICOT question, develop keywords, create a search strategy, and evaluated literature results. For the first few semesters I taught this course, I approached it as a one-shot instruction session, but it just was not enough time to effectively teach the skills necessary. In Fall 2018, I collaborated with the teaching faculty and proposed coming in five times over the course of the semester, to cover each step in the research process, as well as helping student refine their PICOT questions and develop their search strategy.

Conclusion: The outcomes of this program are ongoing. Both the professor and myself, in an informal evaluation process, are seeing more well-develop PICOT questions, robust search strategies, a better understanding of keyword development, database selection and use, and students that are more knowledgeable about the nursing research process. The ongoing, long-term outcome we hope to measure is to determine whether students have a better understanding about the research process, and the necessity of strong search strategies and literature evaluation to inform evidence-based practice.

Poster Number: 31

Time: Tuesday, May 7, 3:30 p.m.–4:25 p.m.

Clinical Social Work at a School of Dentistry: A Little-Known Collaboration

Holly Thompson, Information Services Librarian, USC, Los Angeles, CA

Background: Clinical social workers (CSW) are an established professional presence in medical settings, but few academic dental settings report having integrated social work support into their clinical services. At this University, neither the librarians supporting the dental school nor the librarians supporting the school of social work were aware of the collaboration between these two schools. Social work interns provide services that strive to improve patient compliance and assuage fear or anxiety related to complex dental procedures. The dental librarian shadowed social work interns at the dental school to enhance understanding and provide library support to both interns and patients.

Description: Clinical social workers (CSW) serve as a bridge between clinicians and patients to facilitate communication and understanding, and to navigate barriers to care. It has been observed that more complex dental treatments requiring general anesthesia have high rates of no-show patients, especially pediatric patients. The dental librarian shadowed CSW interns at this school to observe how information is communicated to patients and patient families to ease fears and anxieties, encourage follow-up appointment attendance, and remove barriers to treatment such as challenges with transportation. The social work interns provided vital information to patients, especially those whose primary language is not English. Consumer level dental information was needed to explain complex procedures. CSW interns necessarily learned about biomedical and dental concepts in order to provide clear and accurate information, in addition to learning about administrative and ethical procedures in this clinical setting.

Conclusion: The dental librarian observed avenues for providing information support for patient care at this school of dentistry. A more robust partnership between the librarians and preceptors at the schools of social work and dentistry has been established to improve support of the CSW interns in this setting. In addition to supporting consumer dental health information for patients and patient families, the dental library and librarian will support the CSW interns with appropriate resources to help them learn necessary biomedical and dental concepts. Librarians from the schools of dentistry and social work will collaborate to support the clinical social work program.

Poster Number: 32

Time: Sunday, May 5, 3:30 p.m.–4:25 p.m.

Collaboration and Innovation: The National Network of Libraries of Medicine's (NNLM's) Nationwide Online Wikipedia Edit-a-Thons

Aimee R. Gogan, AHIP, Technical Information Specialist, National Library of Medicine, Catonsville, MD; **Elaina Vitale**, Academic and Data Services Coordinator, National Network of Libraries of Medicine, Middle Atlantic Region, Pittsburgh, PA, PA; **Alicia Lillich**, Kansas Outreach and Technology Coordinator, National Network of Libraries of Medicine, MidContinental Region, Kansas City, KS; **Erin D. Latta**, Coordinator, National DOCLINE Coordination Office, National DOCLINE Coordination Office, Baltimore, MD; **Nora Franco**, Consumer Health Librarian, National Network of Libraries of Medicine Pacific Southwest Region / UCLA Biomedical Library, Los Angeles, CA; **Brian Leaf**, Community Engagement Coordinator, National Network of Libraries of Medicine South Central Region, Fort Worth, TX; **Karen L. Coghlan**, Education and Outreach Coordinator, National Network of Libraries of Medicine New England Region, Worcester, MA; **Ann Glusker, AHIP**, Sociology, Demography, & Quantitative Research Librarian, University of California Berkeley, Berkeley, CA

Background/Objective:

There is a growing trend in libraries of engaging their communities with information and open data by holding Wikipedia edit-a-thons. During these events, participants learn about the culture and norms of this widely used online resource through hands-on editing of articles, including improving citations and adding new facts. The purpose of this poster is to report on the National Network of Libraries of Medicine (NNLM) health-focused online edit-a-thons. These events utilized librarians' research skills to make Wikipedia a better and more evidence-based resource by using National Library of Medicine (NLM) products to add content and citations.

Description:

Spring 2018 Edit-a-thon

A cross-regional planning group of NNLM coordinators organized the first edit-a-thon on April 17, 2018, focusing on rare diseases. Prior to the event, Dr. James Heilman of WikiProject Medicine led a customized online training for participants. Participants were also instructed to create Wikipedia accounts and register their usernames on the NNLM dashboard to track the success of the project. The dashboard content included Edit-a-thon statistics, event information, WebEx details and schedule, citation editing basics, links to NLM resources, and a timeline. On the day of the event, participants were invited to join NNLM's WebEx session to receive online support from NNLM staff and engage with other editors. Members of the planning group took turns acting as virtual instructors. A second edit-a-thon focused on Women's Health was held on November 7th, 2018 and followed a similar format.

Results/Conclusions:

During the Spring edit-a-thon, 32 editors edited articles, 7 articles were created, 111 articles were edited, 736 total edits were made, and 43.5K words were added during the Spring 2018 edit-a-thon. Fewer people than expected joined the WebEx sessions and participants were mainly from the library community. The Twitter feed showed some user engagement, but the majority was from NNLM staff.

The second NNLM Wikipedia Edit-a-thon successfully built on the first edit-a-thon in terms of engagement with more editors and edited articles. Enhanced marketing and promotion lead to increased visibility of the project and more participation. On Twitter, there appeared to be more user engagement than the spring session and librarians from across the United States and Canada posted about the event using the hashtag #CiteNLM2018.

Poster Number: 33

Time: Monday, May 6, 3:30 p.m.–4:25 p.m.

Converting a Face-to-Face Evidence-Based Medicine (EBM) Class to Online Modules

Shannon Sheridan, AHIP, National Library of Medicine Associate Fellow, Drexel Universities, Danville, PA; **Abby L. Adamczyk, AHIP**, Liaison Librarian for Medicine, Drexel University, Philadelphia, PA

Background: Health science librarians taught a two-hour, face-to-face evidence-based medicine workshop monthly as part of a larger program run by the university. Workshop registration was also available for any university affiliates who wished to refresh their search skills. The workshop provided an introduction to locating evidence-based material using resources licensed by the library. The decision was made to convert the workshop to online modules. This was due to the small number of students and high frequency of the class. In addition, one target audience, residents, expressed that online, asynchronous learning opportunities would be more effective for them.

Description: The librarian responsible for creating the modules familiarized themselves with the workshop by shadowing a librarian teaching the class, then teaching a session themselves. The librarian then created a structured outline for the module based on the workshop content. At each stage, input was sought from the three other librarians responsible for teaching the class. The modules included a mix of reading and video lectures, with comprehension questions included in some sections. The librarian wrote scripts for all of the video and reading lectures. The videos were created in Camtasia, and the entire program was assembled in a LibWizard tutorial. This platform was chosen to track module completion for those registered in the university program and therefore required to take the course. To circulate the material further, especially for remote patrons, a companion LibGuide with the same content was created.

Conclusion: This project provides an example of how, with some up-front time investment, librarians can increase information dissemination by converting a face-to-face class to an online format. The completed module was sent to the university program coordinator in late December for implementation in February. We expect to use completion of the module as an outcome measurement for the replacement of the face-to-face workshop. In addition, we will examine the usage statistics of the companion LibGuide to measure the impact and spread of the content beyond the scope of the class, compared to the old metric of affiliate registrants for the class.

Poster Number: 34

Time: Tuesday, May 7, 3:30 p.m.–4:25 p.m.

COI, Oh My! The Current Conflict of Interest (COI) Landscape and the Potential Role of Information Professionals in Supporting Research Quality

Donna S. Gibson, Director; **Robin O'Hanlon**, Associate Librarian, User Services; Memorial Sloan Kettering Cancer Center Library, New York, NY

Background: In 2018, author conflict of interest (COI) controversies made the headlines, bringing this topic to the forefront of the scientific community. COI also became a topic of conversation for information professionals. We explored this issue and uncovered the variability in publishers' guidelines and potential challenges authors may face in navigating the COI landscape. By understanding the complexities of COI as an aspect of the scholarly communications process, information professionals can play a key role in supporting research quality and transparency. We also highlight lingering issues related to publisher COI policies that may impact the development of new service initiatives.

Description: Before developing our support service, information was gathered regarding the scholarly communication environment at our Institution; focusing on the journals that our researchers submit their manuscripts to most frequently and how these publishers inform authors about COI requirements. After compiling the COI guidelines for the top 20 journals/publishers, a chart was developed displaying relevant characteristics including: COI guideline visibility (how many clicks to their policy), whether the guidelines were specific to the publisher or at the journal level, ease of submission (was there a separate COI submission form), if they support the International Committee of Medical Journal Editors (ICMJE) recommendations, and where in the manuscript submission process do authors provide COI statements. This information along with other pertinent guidelines, best practices, and resources will be aggregated into a LibGuide.

Conclusion: Prior to the development of the COI LibGuide, our intended audience will be surveyed to evaluate the most critical elements for resource inclusion and the perceived usefulness of the LibGuide. While the impetus of the COI LibGuide was the direct result of a request from our Legal department, the Library believes this resource will enhance COI best practices for all authors at our institution and beyond. We will update the guide to address emerging standards and related guidelines to support compliance and research transparency. It will also serve as a launching pad for opportunities for librarian outreach and education.

Poster Number: 35

Time: Sunday, May 5, 3:30 p.m.–4:25 p.m.

Course-Long Integration of a Medical Librarian in an Undergraduate Nursing Evidence-Based Practice Course: Effects on Student Attitudes, Skill Development, and Retention

Christopher R. Bishop, Medical Librarian, Marian University, Indianapolis, IN; **Diane Friedman**, Nursing instructor, Marian University, indianapolis

Some undergraduate nursing students report feelings of dread, confusion, and skepticism as they begin the writing-intensive nursing research and informatics course, required by the national nursing education accreditation body. Students worry they will fail and be lost as they are expected to investigate and evaluate recent primary medical literature connected with clinical concerns. Research indicates that these worries shape student commitment to achieving the goals of the course.

An enriched library -professor collaboration has been developed over the past four years to enhance student mastery and comfort with scholarly clinical problem investigation. The librarian not only teaches 8 class periods distributed through the entire semester but also has a required meeting with each student for an exploration of the progress of the student project.

A study was conducted to evaluate if this purposeful collaboration would demonstrate changes of student attitudes and development of student skills of clinical investigation and communication. Data were collected at four points during the semester using Likert-style attitude measures and responses to five clinical scenarios that required the application of information skills.

There is evidence of a simultaneous increase in confidence and competence, as well as in problem-solving. Students report receiving congruent and reinforcing messages of positive attitude, commitment, satisfaction and skills of evidence-seeking from both instructors as a positive, rich experience that will shape their expectations for future experiences of inquiry in their professional careers.

Poster Number: 36

Time: Monday, May 6, 3:30 p.m.–4:25 p.m.

Coverage Analysis of OLDMEDLINE and Embase Classic

Bethany Myers, AHIP, Research Informationist, UCLA, Los Angeles, CA

Objectives: OLDMEDLINE is a database of 2,010,888 journal article citations from from 1946 through 1965. Like MEDLINE, OLDMEDLINE is searchable through PubMed. Embase Classic contains 1,820,335 records, the majority of which are from 1947-1973 (although there are 10,000+ records from 1946). This analysis sought to determine the extent of each database's unique coverage and their overlap over time.

Methods: OLDMEDLINE records were downloaded from PubMed. Since OLDMEDLINE's coverage ends in 1965 but Embase Classic extends through 1973, all PubMed records 1966-1973 were also downloaded. Embase Classic records were downloaded through the Embase platform. Files were then cleaned in OpenRefine. There were several challenges when comparing so many records from two different sources, particularly from historical and international journals. Character encoding proved to be a common problem for author names. Other challenges included the use of hyphens, Roman or Arabic numerals for serial numbering, and spelling differences and/or mistakes. These differences necessitated a matching algorithm that accounted for not only exact matches, but very close matches. Database records were compared with each other using a function from the FuzzyWuzzy Python library. Records that were not matched during this process were considered as unique to their respective databases.

Results: 788,333 records were found as matches between the databases. OLDMEDLINE/PubMed 1966-1973 had 2,240,133 total unique records. Embase Classic had 765,567 total unique records. OLDMEDLINE/PubMed had more unique papers than Embase Classic every year, with the exception of 1965. However, Embase Classic's data quality was very poor compared to OLDMEDLINE, with character issues in database records that were too severe to be corrected during the data cleaning. The data quality prevents an accurate comparison of the two databases by fuzzy matching.

Conclusions: This analysis found that OLDMEDLINE/PubMed through 1973 contained many more unique records than Embase Classic, with the exception of 1965 (this anomaly was likely due to the suboptimal data quality in Embase Classic). However, Embase Classic does contain thousands of unique results that may make it worthwhile for libraries who serve patrons interested in historical medical literature. A future content analysis would be useful to clarify the common and unique research areas represented by Embase Classic and OLDMEDLINE.

Poster Number: 37

Time: Tuesday, May 7, 3:30 p.m.–4:25 p.m.

Creating an Online Institutional Research Record Using Archive Space

Lydia A. Howes, Research and Education Librarian, Southern Illinois University School of Medicine, Springfield, IL; **Geoff Pettys, AHIP**, Head, Reference & Education Services, Southern Illinois University School of Medicine, Springfield, IL; **Michael Moseley**; **Adam John Roloff**, Reference and Education Services Librarian, SIU School of Medicine, Springfield, IL

Background: The School of Medicine was a pioneer in using problem-based learning and simulated patients in medical education. In January 2018 the Library was asked whether the School had a bibliography of the School's contributions to these topics. We did not, but agreed it would be a useful and interesting resource. During this time the Library was also planning to implement an online catalog and finding aid for our archives using the open source platform ArchivesSpace. The death of a distinguished and emeritus professor in June 2018 fast-tracked these projects.

Description: As a starting point for an institutional bibliography the Library first focused on four of the School's earliest and most distinguished researchers. For each, librarians author-searched relevant databases while the archive specialist went through the Medical Library archives. After the bibliographies were "complete," the Library procured a physical copy of each work.

ArchivesSpace, which had been green-lit for installation by the School, was the most logical home for the digital manifestation of the bibliographies. Frustratingly, implementation stalled for months. With the support of the School's Associate Dean of Education & Curriculum, who hoped to unveil the "Founding Figures" Collection of Medical Education at a memorial honoring a recently passed researcher and professor, the Library was able to install ArchivesSpace and begin data entry in November 2018.

Conclusion: These two projects were not initially connected, but by associating our stalled project (ArchivesSpace) with a pressing matter (the memorial), we were able to revive it.

The memorial was held in January 2019 with great attendance, and the Founding Figures Collection was well received. We continue to build an institutional bibliography bit by bit and have identified several researchers that build upon the collection.

Now that ArchivesSpace is up and running, our archive specialist is working on inputting all archival collections and materials. We hope to digitize and, whenever legally possible, make our archive materials available online.

Poster Number: 38

Time: Monday, May 6, 3:30 p.m.–4:25 p.m.

Creating a For-Credit Systematic Review Course for Graduate Students in the Health Sciences

Jason B. Reed, Assistant Professor/Health Sciences Information Specialist, Purdue University, West Lafayette, IN; **Bethany S. McGowan**, Assistant Professor of Library Science and Health Sciences Information Specialist, Library of Engineering and Science, Purdue University, West Lafayette, IN; **Jane Kinkus Yatcilla**, Associate Professor of Library Science, Purdue University Libraries, West Lafayette, IN

Background: We created a for-credit course for graduate students in the health sciences was created alongside a launch of a formal systematic review service, both of which were a response to increasing demand for systematic review support. The learning outcomes are, demonstrated ability to describe the steps in the systematic review process, understand the importance of a reproducible and systematic search strategy, identify bias in health sciences literature, and implement data management strategies. Additionally, it is expected that students will leave with a better understanding of different review types and be able to select the appropriate review for their research project.

Description: The authors worked with the Libraries' curriculum committee to develop a course syllabus and learning outcomes, and to decide on the overall course format. In creating the syllabus, the authors reviewed the literature, drew on their experience offering workshops on systematic reviews and attending systematic review training, and conducted a needs assessment by talking with health sciences faculty members with experience working on systematic reviews with graduate students. The biggest challenge was recruiting students to register for a course listed under the Libraries designation. The authors visited the labs and/or departments of faculty and students they had previously worked with, worked with the Libraries' marketing team to design marketing materials, and utilized various campus listserves to market the course. The first offering, a 1-credit 8-week course, launched in Spring 2019.

Conclusion: The course was met with interest and 10 students enrolled, the maximum course capacity. To assess the course, the authors will collect a post-course assessment that measures students' perceived ability to meet course objectives and outcomes, along with a review of the graded course assignments. The authors will also host a debrief session with the students to assess changes in course motivation and to solicit input in improvements to the structure and content of the course. We plan to offer the course again in Fall 2019, with an added optional hands on lab component to allow for deeper discussion.

Poster Number: 39

Time: Monday, May 6, 3:30 p.m.–4:25 p.m.

Creating an Online Checklist to Evaluate Accessibility of Educational Materials

Charlotte Beyer, AHIP, Instruction and Reference Librarian, Rosalind Franklin University of Medicine and Science, North Chicago, IL

Background: The purpose of this poster is to describe the process for designing an online checklist to review on-demand educational materials such as handouts and videos to ensure they are accessible for users with varying abilities. The checkpoints for this checklist originated from various sources such as Universal Design for Learning (UDL) Guidelines 2.0, W3C, accessibility checkers, and more. This poster outlines the type of checkpoints selected for the checklists as well as how and why the print checklists were converted into an online format.

Description: In 2017, the library sought to make educational materials accessible for users with a wide range of abilities. One of the challenges often faced when improving accessibility is the overwhelming nature of ensuring users with varying levels of ability can access and utilize information presented. Print checklists were made for videos, handouts, and online guides and applied to the library's on-demand educational materials. Some of the challenges with using a print checklist include tracking the usage of the evaluation forms, and being able to easily edit the checklist when needed. In 2018, to make a more efficient evaluation process and find solutions to these challenges, it was decided to transfer the three print checklists into one checklist using Google Forms. In Fall 2018, the majority of the on-demand instructional materials were reviewed using this new more efficient evaluation tool.

Conclusion: Using online form software, the librarian was able to not only save time in terms of data entry, but also easily review results of the evaluated forms in an excel sheet. As the form was used more regularly, best practices of designing accessible materials were reinforced resulting in a smoother design process. This was a win-win as this made the process of designing materials with accessibility in mind less daunting and easier to do for the librarian while improving accessibility for users with a wide range of abilities. Students commented materials were easier to understand after this process was applied.

Poster Number: 40

Time: Tuesday, May 7, 3:30 p.m.–4:25 p.m.

Which Research Data Management Course Is Right for Me? A Flow Chart to Pair Librarians with Training to Meet Professional Goals

Sarah C. Clarke, AHIP, Associate Fellow, National Library of Medicine, Bethesda, MD; **Candace Norton**, Bibliometrics and Outreach Librarian, Zimmerman Associates, Inc., National Institutes of Health Library, Silver Spring, MD

Objectives: Librarians are increasingly bringing Research Data Management (RDM) services to their institutions. Exploring available RDM training can be tedious and time-consuming. The purpose of this project is to produce an easy to navigate decision tree to assist librarians in selecting an RDM course that will best suit their professional development requirements and the data needs of their institution.

Methods: The authors performed a literature search of PubMed, Web of Science, and Scopus to identify online professional development training opportunities in RDM. Search strategies are available on request. In addition, the authors consulted two books on data management for librarians, and performed a web search in Google, supplemented by hand searching select online resources: National Network of Libraries of Medicine RDM resources, Medical Library Association Education Clearinghouse, Coursera, edX, and FutureLearn. Twenty-two courses were identified.

A data capture system was developed in Excel to collect data points on each course for analysis and inclusion in the decision tree: course title, sponsoring organization, time commitment, timeframe, continuing education credit or other completion certificate, cost, and the course activities. Electronic correspondence was utilized when information was not readily available on the training website. A set of general questions regarding unique features of a training course was compiled for use as choices in the tree structure.

Courses were excluded from the decision tree if they did not provide a general overview of RDM, if they did not target information professionals, and if their content was not updated within the last four years.

Results: Seven RDM courses were determined in scope for inclusion into the decision tree. Librarians are directed to follow a series of questions discussing whether certifications, continuing education units or Academy of Health Information Professionals (AHIP) points are desired, whether they favor working at their own pace, if they prefer starting training immediately, and whether they have funding available for their training. As the librarian flows down through these decisions, they will be paired with a course which matches their needs at the bottom. Additional information is included with the course name to include: cost, time commitment, sponsorship, and learning activities.

Conclusions: Researching RDM training courses for librarians is time-consuming. This project produced an easy to navigate, printable decision tree that pairs librarians with an RDM course that best suits their professional development requirements and the data needs of their institution.

Poster Number: 41

Time: Sunday, May 5, 3:30 p.m.–4:25 p.m.

Data Scholar Program: Elevating Our Students through a Cross-Disciplined Program

Vedana Vaidhyanathan, Health and Life Sciences Librarian, Baylor University, Waco, TX; **Joshua Been**; **Christina Chan-Park**, Science Librarian, Baylor University, Waco, TX; **Sinai Wood**; **Eileen M. Bentsen**, Humanities Librarian, Baylor University, Waco, TX; **Carol Schuetz**

Background: Students and researchers in health-related fields increasingly need to find, analyze, and manage data; but they often lack the skills to do so properly. The library has created a library-driven data certificate program in data research to meet this need.

Description: The digital scholarship librarian along with the data management librarian partnered with librarians who work with the health sciences, social sciences, medical humanities and business administration programs to pilot this certificate beginning Fall 2018. This team of librarians created a series of workshops covering analytical tools (Applied Data Research), finding discipline-specific data (Data Content), and Research Data Management. Two tracks with slightly different requirements were outlined: one for undergraduates and one for graduate students and faculty. To allow for greater flexibility, the certificate is designed to be completed between one and four semesters, and participants can pick the most relevant workshops for their research.

Conclusion: We expect to measure an increase in our workshop participation and our individual liaison appointments.

Poster Number: 42

Time: Monday, May 6, 3:30 p.m.–4:25 p.m.

Data to Inform Precision Dentistry

Sarah Meyer, Assistant University Librarian, Univeristy of Florida, Gainesville, FL; **Chris Childs**, Clinical Education Librarian, University of Iowa–Iowa City

Background: The movement toward more precision in healthcare began with precision medicine. The concepts and benefits of the approach are now being adopted into other aspects of healthcare such as dentistry. In dentistry the precision approach utilizes multi-source big and small data coupled with analytical tools to uncover unprecedented insights and broaden the understanding of the connection between oral health and overall health. This type of data-driven research is quickly becoming an essential tool for providers. With more data sources available everyday it is vital for librarians to be cognizant and able to make recommendations on selection of datasets for research

Description: One of the challenges of data-driven research is data that meets the FAIR principles (Findable, Accessible, Interoperable, and Reusable). A team from the dental section of the Medical Library Association aims to create a publicly-available web-based set of tools to facilitate finding and access to relevant data and training for precision dentistry. Its immediate focus is data sources from local, state and national health data. Next project stages will add data including geographic, demographic data, environmental, economic, health services and clinical data on local, state and national levels. As researchers work with the datasets recommended by librarians this will facilitate a better understanding on the methodologies and usefulness of the data to address oral health.

Conclusion: A Library Guide, http://guides.uflib.ufl.edu/dentaldata, links to datasets, data repositories and data providers for potential researchers. Additions will include links to training videos, and literature to inform precision dentistry researchers. As researchers provide insights on the relevance of datasets for precision dental research adjustments will be made to the portal to reflect this feedback. As an emerging field, much of the promise of precision methods, in terms of uncovering insights with data-driven research relies on ensuring the FAIR data principles. Finding and highlighting relevant datasets with a publicly-available web portal will facilitate access, and should advance the research capacities of researchers.

Poster Number: 43

Time: Monday, May 6, 3:30 p.m.–4:25 p.m.

Data Visualization as a Point-of-Need Service: Working with Faculty to Showcase Their Professional Connections

Alexandria Leigh Brackett, AHIP, Clinical Librarian, Cushing/Whitney Medical Library, Yale University, New Haven, CT; **Sawyer Newman**, Data Librarian for the Health Sciences, Yale University, New Haven, CT

Background: This poster demonstrates a service model that blends clinical departmental support, with data services, while also instilling good data management practices. It does this by describing how a Health Sciences library responded to the data needs of the Academic Medical Center's community to be able to communicate the extent of collaboration prevalent in their departmental research efforts. In the service model demonstrated, a Data Librarian and a Clinical Librarian worked with Emergency Medicine (EM) faculty to create data visualizations that show trends in partnerships and mentorships for faculty members within the department and national colleagues.

Description: This service started fall 2018 when the EM department approached the library with a need to develop visualizations of professional connections for two fellows of the medical school's Center for Implementation Science (housed in the EM department), and later to create a visualization of the program's collaborative publishing efforts within the program.

The clinical librarian, as department liaison for EM, was able to use her experience with the department to assist with the citation needs of the project. The data librarian was able to demonstrate network modeling in the open source tool Gephi, and discussed other software options for data visualization. These sessions were also used to discuss principles in data processing and data visualization, as data was transformed from CSV or Scopus (Elsevier) to curated network diagrams.

Conclusion: This experience allowed the librarians to discover and meet new information needs for a clinical department as well as showcase existing data tools and services. On the users' side, the diagrams enable the EM department to demonstrate the value of collaboration, which will be used for program advancement and as supplemental material within an application for funding. This service will be evaluated by noting the increase in number of people involved from Emergency Medicine in these projects, growth in interest by Emergency Medicine departmental affiliates in Research Data Management instruction through the library, and the sustained use of these diagrams.

Poster Number: 44

Time: Sunday, May 5, 3:30 p.m.–4:25 p.m.

Destination Librarian: Helping Others on the Road to Librarianship

Barbara Rochen Renner, Library Services Evaluation Specialist/Allied Health Sciences Liaison and Adjunct Professor, Allied Health Sciences, University of North Carolina at Chapel Hill Health Sciences Library and School of Medicine, Department of Allied Health Sciences, Chapel Hill, NC; **Lee Richardson**, Information Discovery and Metadata Librarian, University of North Carolina at Chapel Hill Health Sciences Library, Chapel Hill, NC; **Jennifer S. Walker**, Cancer Information Librarian / Liaison to the School of Dentistry, University of North Carolina at Chapel Hill, Chapel Hill, NC; **Michele L. Clark**, Business Systems Analyst, University of North Carolina, Chapel Hill, Chapel Hill, NC

Background: The library and information science profession does a good job educating, training, and mentoring students and new professionals. Much of this work is done through formal programs such as internships and networking through professional associations. It can be difficult to provide this support when a person is not eligible or able to participate in typical formal opportunities. It can also be difficult to promote the profession to teenagers and young people when resources about the many different types of positions in libraries and information centers are diffuse and not created with this population in mind.

Description: A health sciences librarian newly assigned to coordinate requests for non-paid experiential opportunities was faced with requests from a broad range of prospective library professionals. The librarian worked with other librarians to design creative approaches that went beyond typical formal programs such as internships and networking through professional associations. These included online outreach to teens interested in the profession, learning experiences for a high school student wanting to become better informed about the profession in order to make good decisions about education and job training, experiences for students with disabilities, and projects for recent library school graduates that provided more practical experience and references to use in their job searches.

Conclusion: This poster shares experiences thinking outside the box to elevate the profession and help students and others pursue a career in libraries. It includes brief lessons learned from coordinating a non-paid experiential program at an academic institution. When students cannot participate in typical formal programs, they may be able to gain knowledge or experience in other ways. Each type of prospective information or library science professional has its own set of rules and challenges that can be met in ways that are rewarding to all involved.

Poster Number: 46

Time: Tuesday, May 7, 3:30 p.m.–4:25 p.m.

Developing a 3D Printing Service: Breaking New Ground

Karli E. White, Technology Enhancement Coordinator / Instructor, Medical Library, Springfield, IL

Background: Using a borrowed 3D printer, staff at the Medical Library printed several models for clinician pre-surgery education and research projects. Based on the positive responses we received, we applied for and received a grant from the NLM GMR (award 16151) to buy our own printer and introduced the Library's 3D printing program to faculty and students in May 2018. Since then we have met with clinical departments to offer 3D printing services, printed models used in medical student and patient education, physician reference and added 3D printing to our community outreach programs.

Description: To raise awareness of our Library 3D printing program, we met with faculty to describe the new service and provided information on creating and acquiring digital models. We also took this opportunity to discuss Library resources and inquired if there were other resources or services the library could offer. We learned that there was high interest in using 3D printing hindered by a lack of models or knowledge of how models could be created and used. With free software, we created printable models from CT scans for physicians to use for patient education and procedure preparation. News of the Library's 3D printing program spread via "word of mouth" and additional members of our medical community visited us and requested information, help with creating models, and printing services.

Conclusion: We continue working with the Surgical Skills Lab to produce models for education, and collaborate with faculty who are actively seeking to bring FDA approved 3D printing to our partner hospital and adding 3D design and printing skills to our curriculum. We have presented at community outreach events for High School students interested in Health Sciences and have been visited by staff from a partner hospital interested in starting a similar 3D printing program. In addition to enabling us to introduce a new and exciting resource, the 3D printing program is helping us build new relationships with our medical community.

Poster Number: 47

Time: Sunday, May 5, 3:30 p.m.–4:25 p.m.

Developing a Controlled Vocabulary for Curriculum Mapping

Annie Cloud Nickum, AHIP, Research and Education Librarian, University of North Dakota–Grand Forks; **Marcia Francis, AHIP**, Southwest Clinical Campus Librarian, University of North Dakota–Bismarck; **Kelly Thormodson**, Director, University of North Dakota–Grand Forks

Background: Librarians at a small medical school joined a new Curriculum Evaluation and Management Subcommittee tasked with revising policies that guide writing learning objectives and mapping those objectives within the curriculum. Other members included instructional designers, academic faculty, course directors, and the medical curriculum coordinator. While these policies have been revised before, this marks the first time librarians have been invited to join teaching faculty in this work. Curriculum mapping is an essential activity in complying with accreditation standards, and librarians can play a vital role in creating policies related to it.

Description: Committee work commenced with a retreat that included presentations highlighting successes and challenges of curriculum mapping at another medical school. A timeline then was established with goals relating to assigning terminology to describe content within the curriculum mapping software, identification of specific content within the curriculum, gap and redundancy analysis, and updating of course objectives. In order to effectively locate content within the curriculum and map objectives appropriately, librarians advocated for creation of a controlled vocabulary as the software did not include this. The subcommittee tasked librarians with creating a controlled vocabulary based upon the USMLE® Content Outline and a list of "hot topics" terms generated by faculty. Librarians compiled a controlled vocabulary list that was adopted for use and also provided recommendations regarding a policy for ongoing maintenance of the list.

Conclusion: While serving on a subcommittee comprised of professionals with various expertise, librarians were provided an opportunity to educate colleagues about their professional roles and unique expertise, especially expertise in the effective organization of information to optimize searching. Librarians developed a controlled vocabulary list to be used when mapping curriculum objectives for efficient searching and analysis of curricular content that has been well-received. Librarians also expect to be involved in the ongoing maintenance of the list to ensure its continued functionality and to adapt it as needed to accommodate new curricular content.

Poster Number: 48

Time: Monday, May 6, 3:30 p.m.–4:25 p.m.

Developing a Data Services Program at Stanford Medicine: Taking a Data-Driven Approach

John Alexander Borghi, Data Services Librarian, Stanford Medicine, Stanford, CA; **Colleen Cuddy**, Director, Research and Academic Collaboration, Stanford University, Lane Library, Stanford, CA

Background: In line with its stated mission to enable biomedical discovery by connecting people with knowledge, the Lane Medical Library has begun to develop a data services program. The goal of this program is to work with data stakeholders throughout Stanford to foster best practices as Stanford Medicine affiliates collect, manage, and share research data. To meet this goal, we are currently engaging in an environmental scan to identify the availability of data-related resources and quantify outstanding needs.

Description: Throughout Stanford Medicine, a variety of programs offer services related to data access, management, and analysis. Stanford Medicine affiliates also have access to programs outside the School of Medicine, such as by the Stanford University Libraries. As part of developing its own data services program, the Lane Medical Library has embarked in an environmental scan to identify key stakeholders and outstanding needs. As part of this scan, we are conducting a short survey of Stanford Medicine affiliates to identify the characteristics of their research data, what software tools they use, and their degree of expertise and desire for training across a number of data-related activities. The results of this survey - which we hope to repeat at regular intervals in the future - will inform the development of workshops and outreach programs throughout the year.

Conclusion: Our poster will detail the results of our survey of data-related practices at Stanford Medicine and how we intend on using those results to inform the development of Lane Medical Library's new Data Services program. These results will provide us with insight about how Stanford Medicine affiliates are presently working with data and will allow us to make data-driven decisions about what software tools, workshops, and other initiatives we should support moving forward. Our poster will also detail how we intend on using our survey results to frame Lane Library's role within broader efforts to coordinate data-related activities at Stanford.

Poster Number: 49

Time: Sunday, May 5, 3:30 p.m.–4:25 p.m.

Developing Online Library Staff Training Using Adult Learning Principles

Anna Ferri, Research and Learning Librarian, Roseman University of Health Sciences, Henderson, NV; **Nena Schvaneveldt, AHIP**, Education Librarian, University of Utah–Salt Lake City

Background: A training gap was identified among non-MLS holding staff triaging health sciences reference requests at the library service desks across two campuses. To close this gap, an asynchronous, online training program was planned, developed, and implemented. The development of training content was guided by the principles of adult learning, and e-learning technology was selected that could support and align with these principles. This included identifying features in Canvas, our Learning Management System where the training was hosted, that enhanced instruction techniques aligned with adult learning principles such as discussions, problem-based activities, and feedback rich assessment.

Description: Before content development began we completed three things: core competency statements in reference skills for staff; needs assessment for selection of an e-learning platform; and an instructional design plan. During the instructional design planning stage, adult learning principles were identified as being critical to instructional design and content development. These included, engagement with and respect for the learner's existing knowledge and skills; problem or task focused content and activities; learner self-direction and control over the training; explanations for why each training module is relevant to the learner; high levels of collaboration, sharing, and engagement with instructors and other learners; and extensive and multi-directional feedback on practice quizzes, activities, and discussions. An iterative content development process ensured that learning materials were reflective of adult learning principles and that e-learning technologies were used to support and enhance learning under these principles.

Conclusion: Challenges to aligning instructional design and content development with adult learning principles included: time, labor, and the skills of instructional staff; variance in existing knowledge and skills in reference services by non-MLIS staff; selection of appropriate e-learning technology; and allowing for learner self-direction and control over the training. Alignment of resulting training modules with adult learning principles and the amount of labor involved in maintaining the active training program will be provided following completion of the first several months of the program.

Poster Number: 50

Time: Sunday, May 5, 3:30 p.m.–4:25 p.m.

Development and Validation of a Search Filter in PubMed MEDLINE for Immigrant Health Disparities

Corinne H. Miller, Clinical Informationist, Galter Health Sciences Library & Learning Center, Northwestern University, Chicago, IL; **Annie Wescott**, Research Librarian, Galter Health Sciences Library & Learning Center, Northwestern University, Chicago, IL; **Ramune K. Kubilius, AHIP**, Col Dev/Spec Proj Librarian, Galter Health Sciences Library & Learning Center, Northwestern University, Evanston, IL; **Q Eileen Wafford**, Research Librarian, Galter Health Sciences Library and Learning Center, Northwestern University, Chicago, IL

Objectives: This project is part of a larger series in which we aim to assess or create filters for health disparities. Our objective is to design and validate a search filter for PubMed MEDLINE to retrieve literature pertinent to immigrant health disparities.

Methods: We solicited expert opinion to address ambiguities in existing definitions of health disparities and immigrant status. Expert feedback guided the creation of inclusion and exclusion criteria. We performed a generic topic search in PubMed and screened the results using the inclusion/exclusion criteria to identify records for the development set. Frequency analysis and hand-searching of the development set were used to generate terms for an immigrant health disparities filter. We hand searched the Journal of Immigrant and Minority Health indexed in PubMed to establish the validation set of articles (the gold standard). This validation set was used to test the sensitivity, specificity, precision, and accuracy of the immigrant health disparities filter.

Results: Through text mining and hand searching of the 86 records in our development set, we identified terminology for the filter. The resulting search strategy retrieved 80 of 86 records. The six missed records from the development set were deemed as "acceptable losses," due to a lack of appropriate title and abstract terminology, resulting in an accuracy of 93%. When tested against the validation set, the filter correctly identified 1028 citations and missed 28 relevant citations. The search strategy correctly excluded 424 citations that were irrelevant and incorrectly included 391 irrelevant citations. Overall, the search strategy relative to the validation set resulted in 97% sensitivity, 52% specificity, 78% accuracy, and 72% precision. The NNR was 1.38.

Conclusions: In phase one of development for the immigrant health disparities filter, we focused on objectively deriving filter terminology from our development set. Filter aspects warranting further consideration include: its focus on U.S. populations, missing relevant terminology (e.g., "displaced persons") not captured by our development set, and geographic and language-specific qualifiers applied to broad keywords. We were unable to incorporate pertinent long phrases, such as "time living in the United States," which have countless iterations, due to Pubmed's phrase index and lack of proximity searching.

Plans for further development include the addition of terms based on expert opinion and topic research, exploration of geographic and language qualifiers, and improvement of filter specificity. Though a work-in-progress, this preliminary filter serves as a foundation on which searchers can build.

Poster Number: 51

Time: Monday, May 6, 3:30 p.m.–4:25 p.m.

Disability and Accessibility: Training Needs of Librarians

JJ Pionke, Applied Health Sciences Librarian, University of Illinois at Urbana-Champaign, Champaign, IL

Objectives: The main objective of this research was to determine the ability level of librarians in regards to accessibility and disability as well as the librarians' perceived needs in regards to assisting patrons with disabilities and making libraries more accessible. To gather a richer contextualization of the problem, a quantitative and qualitative approach was taken in the administered survey by using a combination of multiple choice and open ended questions.

Methods: A national survey was administered in the fall, 2018 via email and social media invitation. Questions were both quantitative and qualitative in nature. Convenience sampling was used. The survey was open for two weeks with reminders sent out every few days. A professional statistician performed descriptive statistics on the results and was also consulted in the creation of the survey.

Results: The survey indicated that respondents feel that accessibility and disability are currently relevant and will be even more relevant in the next five years. The survey also indicated that respondents have varying levels of comfort with assisting patrons with accessibility needs including no comfort with troubleshooting/using assistive technologies. Of the qualitative portions of the survey, respondents had many concerns including: all aspects of accessibility, training around accessibility and disability, poor support from library management, empathy training, and being inclusive of library employees with disabilities.

Conclusions: This survey shows that there are real training deficits around assisting patrons with disabilities and that library employees are concerned about providing excellent services to people with disabilities, both patrons and fellow employees. There is also a strong desire in respondents to have the training necessary to assist patrons with disabilities. Ultimately, the survey indicates that there are training deficits and there is also an awareness that those deficits exist. There is also frustration around the lack of emphasis and resources being dedicated to correcting the deficits. The fact that there is also a desire to correct the training deficits indicates that library employees want to do better and are dedicated to doing so.

Poster Number: 52

Time: Sunday, May 5, 3:30 p.m.–4:25 p.m.

Diversity and Inclusion Initiatives: Countway Library and Harvard Medical School Curriculum

Meredith I. Solomon, AHIP, Outreach Officer, Harvard Medical School, Boston, MA

Background: Countway Library has introduced a new approach with its outreach efforts by taking a more pro-active and integrated role to enrich the student experience. The objective of this program was to incorporate Diversity & Inclusion initiatives directly into the medical school curriculum and to show how the library can be an embedded collaborator. The goal is to supplement academic life by offering the library as a mental/physical/spiritual home for its students, staff and faculty.

Description: Countway Library in collaboration with Harvard Medical School's Program in Medical Education embedded a Diversity & Inclusion event into the medical school curriculum for first year medical students. Each student was given a copy of Black Man in a White with the opportunity to submit questions for the author ahead of time. Author, Damon Tweedy spoke to students about his book, had a Q&A session, signed his books and taught two consecutive writing workshops with a Harvard Medical School faculty member. In addition, the medical school's Office of Diversity Inclusion and Community Partnerships sponsored a Black Med in Medicine dinner where black mend from first year medical students to attending physicians discussed the racial inequalities they have faced as well as topic of mentorship, imposter syndrome and more.

Conclusion: Some of the outcomes we hope students have from this experience is a better understanding of unconscious bias towards their patients, their families and caregivers; to understand the importance of having racially charge conversations to improve the practice of medicine for all.

Poster Number: 53

Time: Tuesday, May 7, 3:30 p.m.–4:25 p.m.

Disability and Accessibility: Understanding the Education Needs of Library Graduate Students

JJ Pionke, Applied Health Sciences Librarian, University of Illinois at Urbana-Champaign, Champaign, IL

Objectives: The main objective of this research was to determine the education that LIS graduate students were receiving in regards to accessibility and disability as well as what students think they need to know. To gather a richer contextualization of the problem, a quantitative and qualitative approach was taken in the administered survey.

Methods: A national survey was administered in the fall, 2018 via email and social media invitation. Questions were both quantitative and qualitative in nature. Convenience sampling was used. The survey was open for two weeks with reminders sent out every few days. A professional statistician performed descriptive statistics on the results and was also consulted in the creation of the survey.

Results: The survey indicated that respondents felt that they were generally not well prepared by their library graduate school educations to assist people with disabilities or to address accessibility issues. The also indicated that they had varying levels of comfort with activities related to disability with the greatest discomfort coming from troubleshooting assistive technologies. Of the qualitative section, respondents indicated that they wanted to assist people with disabilities and also be inclusive and supportive of their fellow library employees who had disabilities. They also indicated that they felt underprepared by their library graduate school educations to adequately assist people with disabilities or utilize assistive technologies. Respondents were particularly concerned about the lack of inclusion in the profession in regards to patrons with disabilities as well as the lack of their voices in the policies that libraries make.

Conclusions: While library graduate programs are strong in teaching library graduate students theories of librarianship, there is a severe deficit in teaching practical skills in regards to how to assist patrons with disabilities and how to troubleshoot assistive technologies. Training for both library graduate students and for current library employees about how to work with both patrons and library employees with disabilities is needed. The current lack of training, both in graduate programs and in the profession, represents a significant oversight by both and is a detriment to providing excellent service to people with disabilities.

Poster Number: 54

Time: Monday, May 6, 3:30 p.m.–4:25 p.m.

E-Books and Print Books: What Do Medical Students and Residents Prefer and Why

Erin Watson, AHIP, Health Sciences Librarian, University of Saskatchewan, Saskatoon, SK, Canada

Objectives: The objective of this research is to determine in which format medical students and residents prefer to use monographs to support their learning and clinical work. Informal feedback from library users at the researcher's institution, and results reported in the literature, have both varied, making acquisition decisions difficult.

Methods: The researcher surveyed medical students and residents at her institution to determine their preferred reading format, and the reasons and circumstances surrounding it. The survey also asked what respondents do if their preferred format is not available. Medical education is delivered through a distributed program at the researcher's institution, therefore, respondents were asked to indicate where they are located; this will help determine if students studying through the distributed program prefer to use electronic materials, or whether they borrow/acquire print materials.

Results: Of 844 possible respondents, 232 answered the survey, a response rate of 27.5%. Not all respondents answered all questions. When reading only a few pages, the vast majority (72%, n=166) preferred to use ebooks. When reading a chapter of 10 pages, the largest number (48%, n=105) preferred ebooks. When reading an entire book, the majority (72%, n=166) preferred print books. Distributed students and residents were not statistically more likely to prefer ebooks to print books. The top reasons for choosing to use ebooks were immediate access to content (n=205; 89%), searchability (n=175; 76%), and accessiblity on the go (n=166; 70%). Top reasons for choosing print books were reduced strain on the eyes (n=177; 77%), easier absorption of information (n=128; 56%), and being able to hold the book in one's hands (n=128; 56%). When respondents preferred to use ebooks, but only the print format was available from the library, a majority (n=140; 61%) would use the print book, though a sizeable number (n=109; 47%) felt they would get the ebook from elsewhere. When print was preferred but only ebooks were available from the library, the majority (n=173, 76%) would use the ebook, though 34% (n=78) might buy their own copy of the print.

Conclusions: While medical students and residents enjoy using ebooks for their convenience and accessibility, they prefer print books for sustained reading. Libraries should continue to make print books available to their patrons, even to those in distributed locations.

Poster Number: 55

Time: Tuesday, May 7, 3:30 p.m.–4:25 p.m.

Easy Steps to Building a Systematic Review Service: A Course for All Health Sciences Librarians

Stephanie C. Roth, AHIP, Biomedical & Research Services Librarian, Temple University Health Sciences Library, Philadelphia, PA; **Natalie Tagge**, Head, Podiatry Library, Temple University Health Sciences Libraries, Philadelphia, PA; **Jenny Pierce**, Head of Research, Education and Outreach, Temple University, Philadelphia, PA

Background: A course, Easy Steps to Building a Systematic Review (SR) Service, was developed for librarians. It's often a belief that one has to obtain expertise before delivering systematic review services. While true for those providing the search, there are several areas that can leverage the expertise of non-experts, especially when providing education. The course was designed for medical librarians with varying degrees of expertise in systematic reviews and from institutions both large and small. Providing systematic review education to librarians was not the goal of this course, instead the course was solely about implementing or developing a systematic review service with a team of experts and non-experts at all levels of educational service as is specified in the coordinating SR service model (1).

Description: This MLA 2 CE course was sponsored by the MLA Philadelphia Regional Chapter and a registration cap of 25 attendees was met. It took place on November 9, 2018 at the Temple University Ginsburg Health Sciences Library. The two hour course consisted of an informal meet and greet, an overview of the systematic review model, an overview of the learning outcomes for researchers and ways to utilize liaison or reference librarian expertise to provide different levels of educational systematic review services. At the end of the course there was a roundtable lunch discussion and attendees could answer and discuss thought provoking questions with table moderators at each table to help guide the discussion. The course instructors included a place for attendees to write in questions to be answered privately by email.

This course is currently available in the MLA course catalog and is approved for 2-4 CE credits (2).

Conclusion: A unique aspect of this course was that all course materials were placed in the Open Science Framework (OSF) and were immediately made available after the course for attendees to reuse or remix the content useful to their needs or their library. At the end of the course, participants left feeling capable of implementing or developing a systematic review service because they had the training and the core materials necessary to be successful. Participants were told that they do not have to ask permission to reuse or adapt course materials as long as attribution is given using a Creative Commons license (CC-BY-NC-SA).

Course Materials:

https://osf.io/u9327/

Poster Number: 56

Time: Sunday, May 5, 3:30 p.m.–4:25 p.m.

E-Books in an Academic Medical Library: Is Our E-Book Collection Aligned with the Medical Programs?

Ella Hu, Biomedical Sciences Reference & Research Informationist, Shiffman Medical Library, Wayne State University, Troy, MI

Objectives: Due to the increasing demand of access to online content, the Shiffman Medical Library has expanded its ebook collection in the past years. The purpose of this study is to explore the correlation of the usage of current ebook collection and the academic programs in the School of Medicine to better inform future collection development decisions.

Methods: This study will analyze the usage data of current ebook collection in the past three years. All raw data will be analyzed by using Microsoft Excel. Usage data will be analyzed by specialties and then mapped to the academic programs in the medical school.

Poster Number: 57

Time: Monday, May 6, 3:30 p.m.–4:25 p.m.

Elevating Allied Health Learners' Knowledge of Health Humanities

Lisa A. Marks, AHIP, Director of Libraries, Mayo Clinic Libraries, Arizona, Scottsdale, AZ; **Bella Panchmatia**; **Katherine (Kit) Kough**, Program Director, Mayo Clinic Center for Humanities in Medicine, Scottsdale, AZ

Background: Health Humanities is becoming more integrated into medical/allied health education. Focusing on allied health learners and their awareness of health humanities, this project included the following programs: NP/PA fellowships, echocardiography, radiography programs, medical physics, physical therapy and pharmacy residencies, dietetic and audiology internships.

• Aim #1: Improved insight into the human condition by providing tools for better, more positive patient encounters, along with increased compassion and empathy for patients and their families.

• Aim #2: Development of a Health Humanities curriculum to support students in connecting the art and science of health care, and applying this to their daily practice.

Description: Students were exposed to a variety of themes to assist in their understanding, awareness and application of health humanities. Students met as a group 4 times over a 6 month period of time. Each meeting had a different theme including an introduction to health humanities, visual art, theater and literature. A pre and post evaluation was completed to compare pre and post knowledge of health humanities. Overall, learners took away a positive experience from the sessions attended based on post evaluation results and comments. This poster will show the work done during sessions as well as the outcomes of the pre and post evaluations.

Conclusion: This poster will show the work done (photos) during sessions as well as the outcomes of the pre and post evaluations on learners knowledge and awareness of the health humanities.

Poster Number: 58

Time: Tuesday, May 7, 3:30 p.m.–4:25 p.m.

Elevating and Disseminating Patient Education Instruction: A Videoconference Class Pilot

Sydni L. Abrahamsen, AHIP, Librarian, Mayo Clinic, Scottsdale, AZ; **Jill Walters**, Patient Education Specialist, Mayo Clinic, Rochester; **Julie Hathaway**, Research Program Coordinator/Patient Education Specialist, Mayo Clinic, Rochester, MN; **Rebecca A. Smith**, Director, Office of Patient Education, Rochester, MN

Background: Instructors at the Patient Education Resource Center and Library of a large academic medical center in the Midwest teach patient education classes to over 11,000 patients yearly. This pilot project tested the feasibility of delivering these classes via videoconference technology to patients at the Southwest site of the same medical center. Instructors at the Midwest site taught classes to patients at the Southwest site. The goals were to:

#1: Test the viability of regularly hosting remote video classes for patients at other locations

#2: Assess patient and staff satisfaction with the videoconference class process and format

Description: Patient education and library staff from the two sites worked collaboratively to develop the process to deliver the education content. The free 60-minute class, Healthy Sleep, was taught over a period of 16 weeks. There were 21 participants, made up of patients and guests, ages 42-85. The class was taught by educators in the Midwest; the patient librarian at the Southwest site facilitated scheduling, welcome, a 'warm hand off' to the instructor, trouble shooting technology and collected patient evaluation surveys post class. Though there were technology and scheduling issues, participants were very satisfied with the education, instructor, librarian interactions, and videoconferencing delivery method. Most would recommend no changes in the class or the format.

Conclusion: This poster will discuss the process of developing this pilot service, course format, lessons learned, feedback from learners and staff, and next steps.

Poster Number: 59

Time: Sunday, May 5, 3:30 p.m.–4:25 p.m.

Elevating Health Literacy: Researching the Landscape

Judy Carol Stribling, AHIP, Assistant Director, Clinical Services, Weill Cornell Medical College, New York, NY; **Antonio P. DeRosa, AHIP**, Oncology Consumer Health Librarian, Weill Cornell Medicine, New York, NY

Objectives: The authors explored viewer satisfaction level and use of interactive tools contained within a four-panel traveling exhibit and Subject Guide designed to raise awareness of and address the extreme problems low health literacy creates in the United States.

Methods: Viewers of the Exhibit offer feedback through surveys in paper or electronic format. Health literacy awareness and Exhibit satisfaction are recorded using ‘yes’ or ‘no’ questions and Likert scale statements. All survey results are recorded in a HIPAA compliant REDCap database. The Exhibit premiered at a health fair held in Manhattan, New York in September 2018.

Results: Preliminary data from 129 surveys (n = 129) provide insight into opinions about health literacy. Respondents reported comfort levels with health information in three domains: importance, ability to assess and understand, and usage in life. Respondents ‘strongly agree’ (n = 100) it is important to be informed about health issues. Respondents ‘disagree’ (n = 50) and ‘strongly disagree’ (n = 17) that it is easy to assess the reliability of information. A majority of respondents reported they use information in their life or for people close to them, ‘strongly agree’ (n = 49) and ‘agree’ (n = 65).

Conclusions: The data requirements for this research protocol are not yet complete. Data will be statistically analyzed upon completion. The Exhibit will travel to other medical libraries and medical school campuses in different areas of the country. The authors believe input from future Exhibit viewers at other locations will reveal important additional insights.

Poster Number: 60

Time: Monday, May 6, 3:30 p.m.–4:25 p.m.

Elevating Instructional Media: Mission, Goals, and Outcomes of a Health Sciences Library's Digital Instructional Media Working Group

Angela Barr, AHIP, Reference and Digital Information Services Librarian, Georgetown University, Washington, DC; **Charles Scott Dorris, AHIP**, Associate Director of Research Services, Georgetown Univ. Med. Cntr., Washington, DC

Background: Health science libraries produce and/or publish an array of digital instructional media, created by a variety of library staff. Questions inevitably arise about how to best manage a growing collection of digital content that may include documents, tutorials, webinars, and more. In order to tackle those questions, Dahlgren Memorial Library's (DML) staff formed a Digital Instructional Media Working Group (DIMWG), tasked with developing best practices for producing, storing, and sharing digital instructional media. Discover our process, observe our progress, and take what you've learned from our experience to put into practice in your own library!

Description: Prior to DIMWG's formation, DML's instructional content lacked design uniformity. Files were named, formatted, and saved inconsistently. Links to content were not standardized. Staff implementation of accessibility features, like captioning, was limited. DML sought balance between sharing instructional materials and encouraging physical attendance at workshops. No tools were being utilized to collect viewing metrics.

Collaboratively, DIMWG identified categories of curation, accessibility, marketing, and archiving which would benefit from standardization. The team developed consistent branding via unified title slide and button designs. We agreed upon naming conventions, file formats, and storage locations. Instructions, stored in a shared cloud drive, were crafted for:

- Creating and processing videos
- Captioning
- Posting content to LibGuides and CMS

Creation of workflows and policies that address integrating accessibility features, controlling access to content, and collecting metrics are ongoing.

Conclusion: Outcomes to measure

- % of DML's videos/workshops that include consistent branding
- % of LibGuide links or embedded videos that have been converted to buttons in standardized sizes
- % of content with agreed upon naming convention
- % of files stored in preferred drive location
- % of videos with captioning; tables of contents
- % of pdfs that have been remediated for accessibility
- Number of guiding policies/documents created re: content production and processing
- Decision on a platform on which to store/share our videos and workshops
- Preliminary data on usage metrics

Poster Number: 61

Time: Tuesday, May 7, 3:30 p.m.–4:25 p.m.

Elevating Library Outreach: Hosting Author Readings

Mary E. Helms, AHIP, Head, Strategic Initiatives, McGoogan Library, University of Nebraska Medical Center, Omaha, NE; **Stuart Dayton**

Background: You may think that someone who works at an academic medical center would concentrate their writing and publishing efforts toward the scientific literature. This is not always true for staff and faculty at the University of Nebraska Medical Center. After participating in the Seven Doctors Project (a multi-week writing course) several years ago, we learned about the many talented and creative people working at UNMC.

To show case these writers and others from the university system, the McGoogan Library decided to hold "author readings" to provide an opportunity for others to learn about the creativity in the UNMC community.

Description: Since fall of 2015, there have been eight author events in the Library, bringing novelists, poets, memoirists, and biographers to read from their books, with two more planned for the spring 2019 semester.

We have had from 20 to 55 attendees at these events and started live streaming over the library's Face Book page in fall of 2017. Our first stream had 77 hits within a week. We use our website, email blasts, digital signs around campus, and traditional snail-mail postcard announcements (to our off campus library supporters). The campus daily online newsletter features a story about the author a week before the reading. At some events, we have worked with the campus bookstore or the author to allow for book purchases and an autograph session at the end of the reading and Q & A with the author.

Conclusion: With the current interest in medical humanities, the author readings are an excellent outreach program for the library to engage and expose the humanist side of our campus community. The McGoogan Library plans on continuing this outreach program in the coming years.

Poster Number: 62

Time: Tuesday, May 7, 3:30 p.m.–4:25 p.m.

Elevating Library Practice through Process Improvement Teams: The Electronic Resources Task Force

J. Michael Lindsay, AHIP, Head of Collections and Access Services, Preston Medical Library, University of Tennessee Graduate School of Medicine, Knoxville, TN; **Rebecca Harrington, AHIP**, Assistant Professor, Research Services Librarian, University of Tennessee Graduate School of Medicine, Knoxville, TN; **Martha Earl, AHIP**, Director/Associate Professor, University of Tennessee–Knoxville; **David W. Petersen, AHIP**, Assistant Professor, Research & Learning Services Librarian, Preston Medical Library, University of Tennessee Graduate School of Medicine, Knoxville, TN; **Cameron Watson**, Library Associate III, Preston Medical Library, Knoxville, TN; **Erin D. Nunley**, Graduate Research Assistant, Preston Medical Library, Murfreesboro, TN

Background: Organizations in many fields, from manufacturing to health care, use process improvement teams, an important aspect of LEAN practice, to improve how they do their work. The library faced a number of challenges with interfaces for electronic resources, including a dated electronic journals interface, out-of-control LibGuides, and lack of control over our web presence. The library formed the Electronic Resources Task Force to foster a collaborative approach to solving these problems. This poster will show what we accomplished and the techniques we used.

Description: The library experienced many changes in the last year including changes in leadership and staff. With our new director's emphasis on improving electronic access and the addition of new team members, this was a great opportunity to use new techniques and develop a team with the goal of providing access to evidence-based, high-quality information resources in a manner that was attractive, modern and seamless as possible. The team meets regularly and has developed a basic flowchart to track ongoing projects. Each meeting provides members with an opportunity to discuss outstanding issues and the status of ongoing projects including the transition to a new electronic journal management tool. The team also reviews metrics, including PubMed Linker stats, full-text downloads, and LibGuide use.

Conclusion: To date, the Electronic Resources Task Force has reduced the number of active LibGuides to 33, and updated these guides. The transition to the new electronic journal management system is nearly complete, and the team has prepared a wish list of improvements for the future.

Poster Number: 63

Time: Monday, May 6, 3:30 p.m.–4:25 p.m.

Elevating Medical Students through Media Education

Alexandra Gomes, AHIP, Associate Director, Himmelfarb Health Sciences Library, Vienna, VA; **Anne M. Linton, AHIP**, Director, The George Washington University, Washington, DC

Background: Building on an existing social media class, librarians sought to teach medical students how to leverage media for their professional benefit. Together with a medical school dean, two librarians wrote and presented a proposal for a "Using Media to Share Health Information" three-credit elective course to the Curriculum Committees, developed the syllabus and grading rubric, wrote lessons plans and assignments, and taught the class in Spring 2018. The class is scheduled to be taught again in Spring 2019.

Description: The media course was well received by the enrolled students. Students developed knowledge and skills in writing for media, shooting and editing digital video, media training, research, advocacy, social media, and health literacy, culminating in the creation of individual public service announcement videos. Many students indicated they plan to continue using their new skills to create additional PSA videos, share health information on social media, and use their media training to deliver polished remarks in public forums.

The experience of developing and teaching the course also expanded the librarians' knowledge and skills in curricular planning and administration as well as in the specific subject content of the course.

Conclusion: It is important for students to be able to utilize media proactively to share health information. Acquiring experience with interviewing and media creation allows students to leverage these popular platforms and reach a broader audience.

In addition, librarians are well-suited to create and deliver this type of course. Librarians are organized and creative, can tap a network of faculty relationships for guest lectures, and can easily handle the associated administrative and teaching responsibilities. Taking on the role of curriculum development strengthens ties with the School of Medicine's faculty and administration, and solidifies the library's role in the formal curriculum.

Poster Number: 64

Time: Sunday, May 5, 3:30 p.m.–4:25 p.m.

Elevating Social Justice through Community Reading and Discussions

Lynly Beard, Research Impact and Social Work Librarian, Health Sciences Library, Seattle, WA

Background: Our university's six health sciences schools have offered a Common Book program for the last eight years. The purpose of this poster is to describe the successes and challenges of library collaboration on Common Book programs.

The book choices are made by a committee with representatives from all the schools. The choice has to appeal to a wide audience. We have covered a variety of topics: most recently we chose books relating to homelessness, mental health and climate change. The events surrounding two of the books were tremendously successful, while the events for the third book were not.

Description: A health sciences librarian participated in the Common Book program and related activities for the last three years, and has learned what makes a successful program for a voluntary activity among busy professionals and professionals-in-training. The committee considers programmatic questions such as whether or not to require students to purchase the Common Book, how to work with faculty to incorporate the Common Book themes into courses, and methods to engage the health sciences community.

Choosing the right book is key to the success of this program. We look at ratings, page length, timeliness of the topic, and if there is a strong faculty sponsor. We look for a topic that crosses many health disciplines.

Conclusion: Assessing the program's success includes evaluating attendance at events and participation over time, participant feedback, and the number of courses that incorporated Common Book themes.

Poster Number: 65

Time: Sunday, May 5, 3:30 p.m.–4:25 p.m.

Elevating Library Services via Data Science Workshops and Consultations

Andrea H. Denton, Research & Data Services Manager, University of Virginia Health System, Charlottesville, VA; **Bart Ragon**, Associate Director, Univ. of Virginia, Charlottesville, VA; **David Martin**, Clinical Data Research Specialist, University of Virginia Health System, Charlottesville, VA; **Marieke K. Jones**, Research Data Specialist, University of Virginia–Charlottesville; **Abbey Elizabeth Heflin**, Analytics and Discovery Services Manager, University of Virginia Health Sciences Library, Charlottesville, VA

Background: The provision of free, open enrollment workshops and one-on-one consultations are long-standing services of an academic medical library . These educational opportunities have traditionally focused on training around information resources such as literature searching and reference management. In 2015, our library added workshops on data analytics and statistics to its roster by contracting with a campus bioinformatics specialist. These initial workshops were well attended and reviewed, so the library made provisions to continue to offer training and also add data analysis consultations by converting first a staff position, and then a librarian position, to data specialist roles.

Description: Library data specialists with expertise in biostatistics and computer science now provide consultations, teach open enrollment workshops, lead courses in degree-granting programs, and participate in campus outreach. Training focuses on applied analysis and statistics topics such as R, SPSS, Stata, and SAS, with the R workshops having the highest attendance. Invited instructors from other campus units provide training in other topics such as GIS and qualitative research tools. A typical semester features over 30 workshops, from beginning to advanced topics, with more than 500 attendees representing varied disciplines and experience levels. Our individual consultations are equally popular, covering topics ranging from how to clean data to prepare for analysis to statistical model diagnostics. The number of requests for statistical support was unexpected, but fortunately both data specialists brought statistical expertise to their roles.

Conclusion: The library’s provision of data analysis services has proven to be a highly impactful way to support researchers at all levels, and further the library’s participation in the research lifecycle. This poster will describe the data specialists’ roles through example services, and illustrate their impact to date through visualizations of topics and attendee metrics for both workshops and consultations.

Poster Number: 66

Time: Monday, May 6, 3:30 p.m.–4:25 p.m.

Elevating Professional Development: Designing an In-House Professional Development Series

Jessica Kilham, AHIP, Manager, Library Education and Clinical Services, University of Massachusetts Medical School, Worcester, MA; **Regina Fisher Raboin**, Associate Director, Lamar Soutter Library, University of Massachusetts Medical School, Worcester, MA; **Mary Piorun, AHIP**, Director, UMass Medical School, Worcester, MA

Background: As part of the library's revised strategic plan, key professional development areas were identified as high priority skills that all staff should receive training in order to collectively acquire a baseline understanding in these areas. These key areas included: Content Strategy (focused on LibGuides and design practices), Assessment, Marketing, Customer Service, and Copyright. The training sessions were designed to improve upon existing skills and develop new skills for all staff, and that aligned with the library's strategic plan.

Description: The series identified core professional development areas based on the strategic plan. Experts on various topics were identified regardless of location and invited to lead a workshop or series of workshops. Most workshops included pre-work to allow for a hit-the-ground running experience. Library management provided protected time for staff to participate in learning sessions, and all staff were encouraged to attend professional development activities. Follow-up task forces were formed to apply the content and to work through action items in the strategic plan. Feedback about the programming was collected from the staff.

Challenges of the program included gaining initial buy-in from the staff to attend workshops outside the scope of their job, identifying topic experts, and balancing the costs of the series.

Conclusion: This program allowed us to align skills of all staff members with the strategic plan by providing core professional development opportunities. It has contributed to the unified effort to develop the skills of all staff by removing the artificial barrier between professional and non-professional staff as well as increase and enhance collaboration on strategic priorities. Based on the success of this pilot program, additional professional development programming including topics such as Diversity and Inclusion, will be offered in the future.

Poster Number: 67

Time: Tuesday, May 7, 3:30 p.m.–4:25 p.m.

Embedded as Adjunct Faculty: Librarians Teach Information Resources for Health Information Management Program

Prasanna Vaduvathiriyan, AHIP, Health Sciences Librarian, Research & Learning, University of Kansas Medical Center, Kansas City, KS; **Sonny Painter**, Educational Technologist, University of Kansas Medical Center, Kansas City, KS; **Rachel Vukas**, Asst. Dir. of Research and Learning, Dykes Library, University of Kansas Medical Center, Kansas City, KS

Background: Librarians identified the information needs of the first-year health information management program students and initiated discussion with the faculty. Discussions were focused on how faculty-librarian collaboration can successfully implement information resources curriculum in the program. The goal of this project was to improve the information literacy skills including critical thinking, literature searching, and writing research papers.

Description: Learning modules such as online searching, evaluating the information, avoiding plagiarism, and using bibliographic management software were created to integrate in Blackboard. Additionally, learning objects created by a consortial curricular project has been included to promote information literacy foundation. A list of topics relevant to health professionals were provided to the students; they selected a topic and wrote a research paper using the skills they learned through the modules.

In the final module, “research in practice”, students conducted literature searches in library databases for writing the research paper. One-on-one consultation with the librarian guided each student through the process. Students were assigned to complete a research map with the research question, search histories, and the selected citations of high-quality peer reviewed articles from the search results. The final research paper was formatted in APA style using the bibliographic management software.

Conclusion: Student learning outcome were assessed based on the summative assignments of each module, and the quality of the final review paper. Students were successful in conducting literature searches, using the bibliographic management program, avoiding plagiarism, and writing a research paper. Students showed interest and enthusiasm to learn and use the research skills they learned to accomplish their academic professional goals.

Poster Number: 68

Time: Sunday, May 5, 3:30 p.m.–4:25 p.m.

Elevating Literary Wellness: A Hospital Library Adds a Leisure Collection and Measures the Impact on Use of the Health Information Center's Resources

Kelsey Grabeel, AHIP, Assistant Director of the Health Information Center, University of Tennessee Graduate School of Medicine / University of Tennessee Medical Center, Knoxville, TN; **Jennifer Luhrs**, Library Supervisor, UT Graduate School of Medicine, Knoxville, TN; **Martha Earl, AHIP**, Director/Associate Professor, University of Tennessee–Knoxville

Objectives: To discover whether adding a policy driven, carefully developed leisure book collection to a consumer health collection (CHC) would lead to an increase in the number of Health Information Center (HIC) clientele and a change in the use of resources.

Methods: The collection development policy, specifically for the Leisure Reading Collection (LRC), included bestselling titles within 3 years, award winning fiction, and classics. Librarians also set criteria with clear parameters related to gifts, donations, and collection maintenance. Twenty-four books were selected for the leisure collection in July 2018. After the addition of the LRC, the new circulation numbers were compared to previous data. Staff also measured the number of new applications for monthly membership in the HIC by consumers. The subject content of materials most used prior to the addition of the LRC were compared to the most popular titles post LRC.

Results: There has been a 41% increase in books checked out from 2017 to 2018. Monthly memberships have increased, averaging 18 new members per month compared to 15. The CHC titles most frequently checked out before the initiation of the LRC were related to diet and nutrition. After, the most popular CHC titles were related to nutrition and stress/anxiety. Librarians informally observed the increased use of the LRC by hospital staff. Staff visited the HIC to check out LRC books for their patients. Attempts to quantify the number of staff using the materials through circulation statistics failed due to system limitations.

Conclusions: With the addition of leisure reading books, the library provided a more valuable and comprehensive collection to its patron base. Changes in the subject material of circulated items, as a result of the LRC were small but worthy of consideration. Further research is planned to measure staff participation. This elevation of the HIC collection has led to a rise in new members, an increase in circulation statistics, and has expanded staff interest in the HIC.

Poster Number: 69

Time: Monday, May 6, 3:30 p.m.–4:25 p.m.

Elevating Scholars' Impact and Influence

Lynly Beard, Research Impact and Social Work Librarian, Health Sciences Library, Seattle, WA

Background: Research Impact is important in so many ways: it measures one's influence outside of the immediate university, and can affect citation counts, research funding possibilities, and promotion and tenure.

This poster will describe how a new Research Impact and Social Work Librarian started to assist Social Work faculty and grad students in managing and expanding their Research Impact. This position provides general library support to the School of Social Work while also implementing and then expanding a Research Impact directive for all of the Health Sciences schools.

Description: The Research Impact Librarian is in the process of developing a program and a toolkit guide that other Health Sciences Librarians can use to assist with Research Impact. Initial activities include identification of easily-implementable pieces and an environmental scan of where faculty are in the publishing process.

Subsequently, specific outreach steps were taken: introduction of ORCID to Social Work faculty, and review of the journals faculty have published in, looking at fit, impact and quality, as well as green self-archiving policies. Additionally, an analysis of citation data in different databases resulted in a report proposing a policy change for the school regarding the use of citation data in promotion analysis.

The Research Impact Librarian prepared a 5 day Research Impact Challenge, based on Brown University's program, which will be tested on Social Work PhD students in January 2019.

Conclusion: Outcomes to be measured include:

1. How willing these students were to engage in a 5 day challenge, 15 minutes a day.
2. If the activities chosen meet their needs.
3. How easy is it to start an Impact Story profile or enhance a Google Scholar profile in a short amount of time.
4. What are the best resources for explaining research to a larger non-research community.

Poster Number: 70

Time: Sunday, May 5, 3:30 p.m.–4:25 p.m.

Evaluation Strategies for Library Services

Alexa Mayo, AHIP, Assoc. Director for Services, Univ. of Maryland, Baltimore, Baltimore, MD; **Katherine Downton, AHIP**, Head of Research, Education and Outreach Services, University of Maryland, Baltimore, Baltimore, MD; **Everly Brown**, Head of Information Services, University of Maryland, Baltimore, Baltimore, MD

Background: This academic health sciences library, serving schools of Dentistry, Nursing, Medicine, Pharmacy, and Social Work, has integrated assessment and evaluation into a range of library services and programs. The objective of this comprehensive evaluation strategy is to understand more fully the preferences, satisfaction, and needs of users to inform service improvement and development. This poster focuses on the comprehensive evaluation of services in 2018-2019: the long-standing library Liaison Program, a newly implemented Bioinformation Program, the single service point Information Service Desk, and an innovative poster printing service.

Description: The library integrates multiple methods in its evaluation strategy. These methods include designing both short, point-of-use opportunities for feedback, and longer surveys and interviews. The Information Services Desk was evaluated using a point-of-use four question survey with emojis representing satisfaction and a field for comments. The Library Liaison Program evaluation included a targeted email to each school at the university with a link to a detailed, 12 question survey. Respondents were invited to provide contact information to participate in a follow-up focus group style discussion. A satisfaction survey was also sent to selected faculty, staff, and students who participated in research consultations. The Bioinformation Program was evaluated using a targeted survey sent to users of the service. The poster printing service online management system automatically generates an online survey each time a poster is printed.

Conclusion: These strategies have enabled the library to improve services and respond to user needs. This poster reports on methods used, results of each of the program and service evaluations, and changes made to the program/service as a result of the evaluation. The successes and challenges in applying the evaluation strategies are outlined. Plans for assessments using new methodologies are also described.

Poster Number: 71

Time: Monday, May 6, 3:30 p.m.–4:25 p.m.

Evidence-Based Practice: Enhancing Teaching Methods with Time-Saving Tools

Sarah Meyer, Assistant University Librarian, Univeristy of Florida, Gainesville, FL; **Terry Kit Selfe, AHIP**, University of Florida–Gainesville

Background: Utilization of Evidence-Based Practice (EBP) as a lifelong tool in clinical practice, has the ability to reduce an estimated time lag of seventeen years, to translate research into practice. Therefore, it is vital to develop methods that are cognizant of time constraints which limit a practicing clinician's ability to implement new evidence into practice. Finding evidence is a critical step of EBP. In the Evidence-Based Dental Practice course this step is taught by librarians. In 2018 librarians refined this section of the course with an objective of instilling a time-saving method students could easily adapt for daily clinical practice

Description: Previously, this section of the EBP course was limited to instruction on selecting and searching relevant biomedical literature databases to identify potentially relevant evidence for common evidence-based clinical scenarios. This information was not used again until a subsequent lecture on critical appraisal weeks later. Librarians refined the course section by including simultaneous instruction on citation management software to document and organize identified literature. Students were instructed on a quick method to collect, share and organize identified evidence for the next stage of EBP, critical analysis.

Conclusion: Finding and organizing evidence are critical steps in the EBP process. To ensure long-term impact of the EBP approach, it is critical to refine teaching methods to consider time constraints that will intensify once students become practicing clinicians. Effective teaching of EBP which includes relevant tools can help meet this objective. Class attendees stated that inclusion of citation software helped to instill a methodology for EBP, improving their ability to organize evidence and prepare for the next stage, critical appraisal. Other teaching faculty, stated that students had a more in-depth understanding about the importance of organizing and documenting evidence

Poster Number: 72

Time: Tuesday, May 7, 3:30 p.m.–4:25 p.m.

Executing a University-Wide Research Dissemination Blitz

Emily J. Glenn, Interim Associate Director, Education & Research Services, McGoogan Library of Medicine, Omaha, NE; **Heather L. Brown**, Associate Director, Collection Services, University of Nebraska Medical Center, Omaha, NE; **Christian Minter**, Community Engagement and Health Literacy Librarian, University of Nebraska Medical Center, Omaha, NE

Background: In preparation for the university's high-profile Research Month held each October, and in light of Open Access Week, also in October, librarians at one academic medical center developed plans to spur engagement in research support offerings by the library. Taking advantage of two major events, we devised a multi-faceted promotion approach that involved university-wide messaging, librarian liaison direct communications with faculty, staff, and students, and research-related event hosting. This poster describes several tangible, low-to-moderate effort ways for an academic medical center library to engage with their communities about support for researchers throughout the research lifecycle.

Description: The university's annual monthlong celebration of research-related education, training, conferences, and events provided the perfect backdrop for the library to demonstrate support for researchers throughout the research lifecycle. Librarians proposed several ideas to the university's research month planning team. The library would lead or facilitate sessions on data visualization, authorship, web presence, systematic reviews, open access, research dissemination, and author rights. We would also hold an author reading by a university researcher and host an invention competition. Lastly, we proposed the promotion of student research and open access publishing via one-time funding of article processing charges (APC) for a student-authored scholarly research article. Several venues would be used for the promotion of events and library resources. The primary means of evaluation would be attendance and engagement at events and follow-up contact with the library.

Conclusion: Outcomes focused on engagement: number of impressions on due to promotion, number of attendees, and the number of submissions for the student funding opportunity. The most successful event was the Invent-a-thon, which brought together about 70 high school students. Late-breaking training on our institutional research portal was popular with administrative staff. While only a handful of people attended other in-person sessions, many participated in the livestreaming. Skills-related outcomes were not immediately measurable in participants, but awareness of library support for research was increased with the participant contact in each program. Outcomes from these events will future Research Month programming.

Poster Number: 73

Time: Tuesday, May 7, 3:30 p.m.–4:25 p.m.

Experiences of Animal Researchers: Their Required Literature Search for Alternatives and Their Use of Reporting Guidelines

Melissa Funaro, Clinical Librarian, Cushing/Whitney Medical Library, Yale University, New Haven, CT;
Kate Nyhan, Research and education librarian, Yale University, New Haven, CT

Objectives: Researchers who work with USDA covered species are required to do a literature search for alternatives to painful procedures. The purpose of the study is to determine how these researchers view their literature search. In addition, the survey asks participants who have published their research on animals if they've used any reporting guidelines such as PREPARE or ARRIVE.

Methods: A Qualtrics survey was developed and edited to ensure quality and clarity. The protocol was submitted to the Institutional Review Board (IRB) and found to be exempt. A survey was distributed on March 14, 2019, via the university message system to 2,850 faculty and laboratory staff that use USDA covered species in research. A reminder email was sent to the same listserv on April 1, 2019.

Results: Of the 24 respondents required to do a search for alternatives, 9 of them received help from the library either through consultation or the librarian did the search for them. Of the 13 respondents who didn't receive help from the library, 4 stated they didn't need help and 6 people didn't know the library could help them.

Researchers who responded to our survey expressed respect for and gratitude towards librarians who designed and ran mediated IACUC literature searches, but they did not consider the IACUC literature search requirement to be useful in finding alternatives to painful or distressful procedures.

Respondents who have not yet published reported that they will use reporting guidelines, including Gold Standard Publication Checklist and ARRIVE. However, few respondents who have published already reported that they used reporting guidelines.

Many respondents did not know what reporting guidelines are.

Conclusions: The findings of this study have informed the continuing development of the IACUC literature search program at our institution. We are happy to hear respondents are comfortable doing the searches on their own but we will continue to provide outreach to make sure researchers are aware of our services. We continue to conduct mediated searches to help researchers comply with IACUC requirements. We will expand our educational offerings around reporting guidelines.

Poster Number: 74

Time: Monday, May 6, 3:30 p.m.–4:25 p.m.

Exploring the Reuse of Open Clinical Trial Data through Citation Analysis

Sawyer Newman, Data Librarian for the Health Sciences, Yale University, New Haven, CT; **Holly K. Grossetta Nardini, AHIP**, Associate Director, Cushing/Whitney Medical Library, Yale University, New Haven, CT; **Ginger Gamble**, Research Assistant II, Yale School of Medicine; **Jessica D. Ritchie**, Project Manager, Yale University, New Haven, CT; **Joseph S. Ross**

Background: The purpose of this poster is to examine the reuse of clinical trials research data through a campus-based open data access project in order to understand data reuse in health science research, and to demonstrate how the library can build ties with other data and information centers within the University system through the use of library tools and services.

Description: The library and the selected open data access project have been exploring how best to collaborate. The groups both work as information/data providers at a University and have decided to work together to promote use of the open data access project.

In this work, the library mapped clinical trial data to approved research proposals requesting data from the open data access project. Based on this initial mapping, other data and citations of published works resulting from the original request for data were mapped. The resulting data visualizations illustrate reused research data as a part of the citation network of health science publications.

This research will be evaluated by the thoroughness of the data collection measures, and by its applicability and adaptability to other data-centric citation analysis research.

Conclusion: The expected measurable outcomes of this effort will be the insight about reuse of data gained from the completed analysis. We will also continue to evaluate our collaborations to meet the needs of researchers seeking data for reuse. Our expectation is that our joint investment in this project will elevate the awareness of data services.

Poster Number: 75

Time: Tuesday, May 7, 3:30 p.m.–4:25 p.m.

Exploring Use of Alternative Web Collecting Tools to Supplement Current Archive-It Web Collecting

Gabrielle Barr, Research Fellow, Northwestern Galter Health Sciences Library & Learning Center, Chicago, IL

Background: Prior to 2018, the web-archiving team solely relied on the Internet Archive's Archive-It web harvesting service to collect information about health and disease outbreaks. Archive-it has many attributes, from ease of use to having responsive support, but archiving social media has been a challenge. Given the fact that social media has become such a dominant form of communication in the 21st century, the library embarked on a project to explore some of the new tools designed for capturing dynamic content as well as review the feasibility of implementing one or more of these tools if the trials were successful.

Description: After conducting an environmental scan of web-archiving tools, Webrecorder, Brozzler, and Social Feed Manager were selected for further exploration. Installing the software was the second step. The third part of the project was testing and analyzing crawls. With Webrecorder, I crawled sites using Internet Explorer, Chrome, and Firefox. Some of the sites were transferred to the Webrecorder Player while others remained on online storage provided by the program. To observe the effectiveness of Brozzler, I tested social media sites using Archive-It and the Brozzler technologies. The crawl instructions for capture were made to be identical to get a more valid comparison. Quality assurance work was performed, and crawl parameters were adjusted accordingly. Because Social Feed Manager could not be installed during the project, arrangements were made to view a demonstration of it at a local university.

Conclusion: Outcomes were not definitive. Social Feed Manager was unable to be installed for technical and ethical reasons. Webrecorder generated quality, consistent results but was time-consuming to use and presented curation challenges due to the subjectivity inherent with the technology. While crawls were easy to conduct and access with Brozzler, in our own tests, the crawler did not perform better than the standard Archive-It crawler. Since the conclusion of the project, the library continues to experiment with Webrecorder, making use of a new Archive-It feature that allows for the uploading of WARCs from external captures, and has had success with Brozzler.

Poster Number: 76

Time: Sunday, May 5, 3:30 p.m.–4:25 p.m.

Extending Our Reach: Integrating Librarians and Library Resources into Canvas

Beth Auten, AHIP, Health & Human Services Librarian; **Catherine Tingelstad**, Instruction & Curriculum Engagement Coordinator; UNC Charlotte, Charlotte, NC

Background: In the fall of 2017, our university completely transitioned from Moodle to Canvas for all courses. Several librarians developed and implemented a plan to create coordinated library access for all students through the Canvas learning management system. The goals for developing a library presence in Canvas included reaching students at the point of need, creating awareness of library services and resources, defining a librarian role in courses, and better serving online students.

Description: Partnering with campus information technology services, librarians developed a specialized role in Canvas allowing instructors to embed them in courses. The role was tested before being implemented during the spring semester in 2018. Librarians also used the LibApps LTI (learning tools interoperability) to integrate research guides into Canvas, using course metadata to map guides to the appropriate subject, course, or course section. The librarians leading the project also trained their colleagues to enter appropriate metadata linking guides to courses during a summer editing event. The LTI was implemented and guides were mapped to Canvas courses by the fall semester of 2018.

Conclusion: Evaluation of the impact of adding a librarian role and mapping research guides to the Canvas LMS is ongoing. As of March 19, 2019, course instructors used the librarian role to incorporate a librarian into 124 courses. Usage statistics available from LibGuides show that research guides received 8,417 more views in the fall of 2018 than in the fall of 2017. The team is looking at the changes in access at the individual guide level, since neither Google Analytics or the data available from Springshare accurately represent the changes in guide usage that have taken place.

Poster Number: 77

Time: Tuesday, May 7, 3:30 p.m.–4:25 p.m.

Faculty Knowledge and Attitudes Regarding Predatory Journals: A Needs Assessment Survey

Stephanie M. Swanberg, AHIP, Associate Professor, Information Literacy & eLearning Librarian, Oakland University William Beaumont School of Medicine, Rochester, MI; **Joanna Thielen**, Research Data and Science Librarian, Oakland University Libraries, Rochester, MI; **Nancy Anderson**, Director, Medical Library, Oakland University William Beaumont School of Medicine, Rochester, MI

Objectives: An unfortunate consequence of the Open Access movement is predatory journals whose sole purpose is to make a profit, not disseminate quality, peer-reviewed research. Publishing in such journals can negatively impact faculty reputations and promotion/tenure. Yet many publish in these journals, either knowingly or unknowingly, including at our institution. This project investigated faculty knowledge and attitudes regarding predatory journals.

Methods: A medical library at a Midwestern university has offered faculty education sessions on predatory journals since 2016 and has tracked several faculty publishing in questionable journals in our institutional scholarly publication tracking process. This study served as an educational needs assessment of university and medical school faculty knowledge and attitudes about predatory journals. A 20-item Qualtrics questionnaire containing both quantitative and qualitative questions was developed and piloted. All university and medical school faculty were invited to participate via university email listservs in February 2019. The survey remained open through the end of April 2019. The survey included knowledge and attitudinal questions aimed at assessing participants' ability and confidence in identifying characteristics of predatory journals and their opinion on the importance of discussing such journals at the institutional and professional levels. Demographic items collected included rank, field, and total career publications. Data will be statistically analyzed using chi-square testing.

Results: A total of 186 faculty have completed the survey to date including 38% medical school and 62% university faculty. Initial analysis reveals that 27% (n=50) of respondents had not previously heard of the term "predatory journal" and when asked to review and identify the legitimacy of a predatory journal in their field, 15% incorrectly determined it was legitimate (n=25) while 60% determined it was predatory (n=100) and 25% unsure (n=41). Yet, 83% (n=135) reported feeling very confident or confident in their ability to assess journal quality. These initial results suggest gaps in faculty knowledge including correctly identifying indicators of predatory journals. With only 14% (n=22) reporting previous training on predatory journals, results will be used to develop targeted workshops for faculty.

Conclusions: Most literature on predatory journals has been opinion-based aimed at increasing awareness. By measuring faculty knowledge of such journals and understanding if they perceive them as a threat, libraries can better promote and tailor education sessions to meet their needs. The results could also be applied at other institutions in understanding faculty knowledge and attitudes about predatory journals more broadly.

Poster Number: 78

Time: Tuesday, May 7, 3:30 p.m.–4:25 p.m.

Fast Track to Consensus with SR Express: Elevating Systematic Review Partnerships

Rie Goto, Medical Librarian, The Hospital for Special Surgery, New York, NY; **Bridget Jivanelli**, Medical Librarian, Hospital for Special Surgery, New York, NY

Background: The purpose of this presentation is to illustrate how a hospital library with 2 full time librarians successfully implemented a new service. SR Express expands our services beyond SR consultations by creating a packaged service including research consultation, instruction session, search strategy creation, citation management, and full text retrieval and upload, and elevated ourselves to be recognized as a partner in research.

Description: SR Express service started in 2018. The volume grew and it almost doubled from 19 SRs in 2017 to 30 SRs in 2018, a 58% increase between two full time librarians. In March 2018, the Anesthesiology and Pain Management Department approached the library with a SR search strategy request to answer 2 questions for their consensus meeting. The librarians played a big role as members of the core research team, provided assistance beyond our regular SR Express service by assisting the project manager, troubleshooting technical difficulties with Covidence, manipulating folders of citations by combining related questions into one, dividing up to into sub-groups, and tagging. We participated in the consensus meeting by reporting search strategy, PRISMA flow chart, and final citation numbers as a member of the International Consensus on Anesthesia Related Outcomes after Surgery (ICAROS) group.

Conclusion: The librarians wrote the method section and provided PRISMA flow chart, and contributed in manuscript reviews and revisions. The librarians are listed as authors in the manuscript and it was submitted to BMJ for publication. The success of SR Express has led to other department approaching us for assistance for their consensus meeting, as well as increased requests for SR participation. The library will offer consensus project management service and include feedback survey in the SE Express service in the future.

Poster Number: 79

Time: Sunday, May 5, 3:30 p.m.–4:25 p.m.

From Nursing Student to Practicing Nurse: One Institution's Pipeline from School of Nursing to Health Care System and Its Impact on Library Instruction and Nursing Research Publications

Sharon Leslie, AHIP, Nursing Informationist, Emory University, Atlanta, GA; **John K. Nemeth**, Clinical Informationist, Emory University. Woodruff Health Sciences Center Library, Atlanta, GA; **Hannah Rutledge, AHIP**, Head of Clinical Informationist Services, Emory University, Atlanta, GA

Background: Historically, our academic health sciences library has served two nursing populations: The School of Nursing within the private university, and an expanding healthcare system associated with the university. These two organizations are joining together to alleviate the healthcare system's nursing shortage by creating a student-to-employee pipeline program. Following graduation and successful licensure, the nurses will be hired by the healthcare system with a two-year commitment. One library serving two different yet related populations can be challenging, but the pipeline program gives the library an opportunity to align information literacy training provided during nursing education and nursing practice.

Description: This poster describes a new nursing program at Emory University. The information is based on preliminary interviews with program administrators and early (reactive) efforts of the library. While there are commonalities between student nurses and practicing nurses, distinct issues determine the library instruction offered, including access to electronic resources and computer workstations. Literature suggests that clinical nurses may have lower information literacy skills, preferring basic internet searches while nursing students are taught to rely on subscription-based information products. We will interview key players of these distinct user groups to determine their priorities and use this information as a catalyst to strategically rethink and implement revised training at each institution.

Conclusion: As the program evolves, we are excited to gain a better understanding of the journey from student to practitioner and to see the immediate translation of learning into clinical practice. We plan to share our ongoing efforts and challenges at future MLA meetings.

Poster Number: 80

Time: Monday, May 6, 3:30 p.m.–4:25 p.m.

Faster Together: Facilitating the Recruitment of Minorities in Clinical Trials through Modular, Evidence-Based Training Using Coursera

Elizabeth T. Frakes, Information Scientist, Center for Knowledge Management, Strategy and Innovation, Vanderbilt University Medical Center, Nashville, TN; **Sheila V. Kusnoor**, Senior Research Information Scientist, Center for Knowledge Management, Strategy and Innovation, Vanderbilt University Medical Center; **Taneya Y. Koonce**, Associate Director for Research, Center for Knowledge Management, Strategy and Innovation, Vanderbilt University Medical Center, Nashville, TN; **Mallory N. Blasingame**, Information Scientist, Center for Knowledge Management, Strategy and Innovation, Vanderbilt University Medical Center, Nashville, TN; **Victoria Villalta-Gil**, Research Scientist Senior, Meharry Vanderbilt Alliance, Vanderbilt University Medical Center; **Consuelo Hopkins Wilkins**, , None; **Nunzia B. Giuse, FMLA**, Vice President for Knowledge Management | Professor Biomedical Informatics, Medicine, Strategy & Innovation, Vanderbilt University Med. Ctr., Nashville, TN

Background: Faster Together is a federally-funded research initiative aimed at enhancing the recruitment of minorities in clinical trials. One of the key components of the project is to develop a training course for clinical trial recruiters stressing the importance of diversity in clinical trials and strategies to increase minority recruitment. Here we discuss the role of our team of knowledge management information scientists in developing the course. Our main contributions were to: 1) evaluate online learning platforms; 2) ensure course materials were evidence-based and adhered to clear communication best practices; 3) implement the content into the chosen technological courseware platform (Coursera).

Description: The project team consisted of content experts, videographers, and our team of information scientists. After developing and applying a systematic and rigorous evaluation process to categorize and differentiate learning platforms, Coursera was chosen. Coursera has many attributes in its favor including an online open modular learning format, name recognition, auto-graded assignments, community discussion forums, technical support, and mobile learning. We reviewed educational material developed by the content experts to ensure it was supported by evidence, adhered to clear communication best practices, and met Coursera's pedagogical and technological requirements. Throughout the course of this collaboration, we became experts on the abilities, limitations, and restrictions of Coursera while also using our in-house expertise to deliver additional skills and knowledge (e.g., exploring parameters for integration with third-party survey software). We also developed custom local programming (e.g., matching exercises, generation of course completion certificate).

Conclusion: The course launched on April 1st, 2019. Outcomes that will be assessed include the number of individuals who enroll and ultimately complete the course, as well as performance on learning assessments. Our team's experience may be helpful for other librarians and information professionals interested in developing their own online learning courses or assisting others in creating them. Coursera is a viable choice for knowledge professionals to create an educational experience in an existing framework.

Poster Number: 81

Time: Tuesday, May 7, 3:30 p.m.–4:25 p.m.

From Obsolescence to Ingenuity: Selecting and Implementing a Replacement for Defunct Instructional Technology

Christina Heinrich, Research & Instruction Librarian; **Berika Williams**, Emerging Technologies and Web Librarian; Hirsh Health Sciences Library, Boston, MA

Background: Technology obsolescence often necessitates unforeseen disruption in library instruction that can have consequences for students, administrators, faculty, and library staff. The research and instruction department was tasked with finding a new instruction tool to house two self-paced, interactive modules that are a compulsory part of medical students' Problem-Based Learning coursework. This change was prompted by the discontinuation of Microsoft's Office Mix. Through a process of identifying needs and resources, we leveraged the unplanned change to create an improved version of the modules in Canvas.

Description: First, we assessed our needs and developed a list of requirements. Based on these needs, we identified possible software solutions. In addition, we partnered with educational technology services to explore additional software alternatives available through the university. After evaluating our options, we selected the Canvas Learning Management System to create the interactive modules. We then transferred the old content to Canvas, taking the opportunity to refurbish the curriculum with new learning objectives, videos, quizzes, and slides. At the conclusion of the modules, students were asked to provide feedback on the material. They were also required to complete a group assignment on evidence-based medicine, applying the concepts and search techniques from the modules. Throughout the semester, students completed individual assignments where they researched a PICO question and applied EBM principles. We used these assignments along with student feedback to measure engagement with the new tool.

Conclusion: The library received positive feedback from medical school faculty. Several students provided positive feedback about Canvas and the presentation of the material where they were solicited for feedback at the end of the modules. Moving forward, student comments also raised important issues about ongoing access to the modules and improvements for the next cohort.

Poster Number: 82

Time: Sunday, May 5, 3:30 p.m.–4:25 p.m.

Going It Alone: Systematic Reviews without Assistance from a Librarian

Kate Nyhan, Research and education librarian, Yale University, New Haven, CT; **Alyssa Grimshaw**, Access Services/Clinical Librarian, Cushing/Whitney Medical Library, West Haven, CT

Objectives: Despite the availability of an innovative and free systematic review service, some researchers at our institution publish systematic reviews without the assistance of a librarian. To better understand researcher behavior and the quality of research resulting from these projects, we investigated systematic reviews that have been produced at our institution without librarian involvement, as other librarians have at their institution.

Background: At Cushing/Whitney Medical Library, Jan Glover started supporting systematic reviews in 2005. Since then, twelve trained librarians have contributed as co-authors to twenty-five published systematic reviews and other evidence synthesis publications. In addition, the library supports evidence synthesis with a seven-member Cross-Department Team that supports citation management and screening, a site license of Covidence, and tools like the Yale MeSH Analyzer. Despite this institutional and individual investment in high-quality evidence synthesis, we are aware of many systematic reviews being conducted by Yale authors who have not used our services.

Methods: We divided recently published systematic reviews with a Yale first author into three sets: reviews with a librarian co-author, reviews created with some assistance from a librarian (i.e. consultation), and reviews created without any known assistance from a librarian. We investigated these sets in terms of research impact and analyzed the departmental affiliations of first authors who created reviews without librarian assistance. We assessed the quality of the 23 reviews published in 2018 without librarian assistance, in terms of search quality (PRESS), review quality (AMSTAR 2), and compliance with reporting standards (PRISMA).

Results: At our institution, authors publish systematic reviews without librarian involvement more often than they publish reviews with librarians as co-authors or consultants. Reviews without librarian involvement are low in quality. Reporting was incomplete, especially PRISMA items for the methods and results sections. Searches were poorly documented and, based on what documentation was provided, not comprehensive; PRESS revisions were suggested or required in every case we evaluated. AMSTAR 2 appraisals were low or critically low in most cases we evaluated.

Conclusions: The findings of this study could lead to redesigning our systematic review program or improving our current outreach strategy. The findings may also facilitate future research, perhaps with surveys, interviews, or focus groups, into the motives and preferences of researchers who produce systematic reviews without librarian involvement.

Supporting materials: https://osf.io/xjr6t/

Poster Number: 83

Time: Monday, May 6, 3:30 p.m.–4:25 p.m.

Hands-on Practices toward Customized Medical Materials and Patient Education Materials with a Digitized Database

Hui-Chin Chang, Director / lecturer, Library, Chung Shan Medical University Hospital / School of Medicine, Chung Shan Medical University, Taichung, Taiwan (Republic of China); **Kuo-Shu Huang**, Associate Professor, Chung Shan Medical University / Institute of Medicine, Taichung, Taiwan (Republic of China); **Hui-Ying Low**, Department of Obstetrics and gynecology, New Far Eastern OB/GYN Hospital, Taichung, Taiwan (Republic of China); **Tzu-heng Chiu**, University Librarian, Taipei Medical University, Taipei, Taipei, Taiwan (Republic of China); **Kevin Ma**, Researcher, University of Pennsylvania

Objectives: An on-site anatomy education workshop with hands-on session for faculty members working in medical centers was offered, so as to improve the awareness of adopting digital anatomical databases while designing anatomy-related teaching materials for students or patients. The utility of a library-launched CME workshop for introducing 3D anatomy database for educational purpose was evaluated.

Methods: Lectures were delivered to participants with one personal computer per individual and projectors in computer classrooms with screen-sharing and remote-access. Three-dimensional (3D) anatomical visualization systems and medical imaging software for photo editing were offered, along with questionnaires issued before and after the workshop. Faculty members were divided into groups by affiliated department for designing teaching materials in clinically based contexts, and instructors were assigned to each group. The objectives were to assess participant performance outcome, capability of creating customized materials from relevant content of the 3D anatomy database, perception of cooperating and supporting team members to complete the assignment in the hands-on session. Analysis of pre-/ post-test was assessed by two-sample t-test for paired data, and the effect of demographic information on the helpfulness of the workshop was evaluated by two sample t-test or ANOVA.

Results: A total of 56 people attended the course, 48 questionnaires were collected, and 34 questionnaires were valid. The average teaching years of participants is 9.55±6.96 years.

The satisfaction of this course is very high; in the future, more advanced educational training content can be designed for teachers who need to use the database to create teaching material. This fact emphasizes the role of library as an effective continuing medical education (CME) provider. The results suggest that once platforms for resource utilization are established in libraries, faculty members have higher perception, adaptation and acceptance of novel educational technologies ($P < 0.05$).

Conclusions: Such large-scale hands-on workshop is the first one in central Taiwan, and effectively improved the awareness and knowledge toward applying digital anatomic databases for educational purposes. Previously, we launched a workshop only with lectures, and did not observe significance improvement in participant performance. Hence, such hands-on workshop would be an innovative form of library CME, providing an informative platform for libraries to assist both faculties from basic and clinical science to design teaching materials. In addition to sharing education experiences, individuals are also allowed to familiarize database operation and preparing novel teaching materials through practical implementation.

Poster Number: 84

Time: Tuesday, May 7, 3:30 p.m.–4:25 p.m.

Harnessing the Power of ORCID on Campus: ORCID Integration Lessons Learned

Jane Scott, Manager, Digital Services & Technology Planning; **Jon Crossno, AHIP**, Cataloging & Metadata Librarian; UT Southwestern Medical Center, Dallas, TX

Background: The university embarked on a campus-wide ORCID integration process in July 2018 with the Library's Digital Services and Technology Planning unit leading the charge. The purpose of this project was to enroll the campus community in ORCID and to capture their ORCID iDs for future uses in our Information Resources Enterprise Data Warehouse to assist with various campus reporting needs that are currently time-consuming tasks.

Description: The Library embarked on a multi-stakeholder approach of identifying and including appropriate campus departments and divisions. Coordination with Information Resources and the Library's Web Applications Developer determined the optimum infrastructure where ORCID iDs would be permanently captured from various options. Working with the administrative end users of the data helped determine reasonable output expectations and opportunities. A SharePoint workspace was created to regularly communicate with the 80+ campus members vested in this project implementation and scheduled launch.

Conclusion: Collaboration and open communication about the project proved to be valuable in progressing the project along and holding people accountable to various tasks needed to make this process successful. We are exploring various promotional strategies and determining long range onboard training requirements and campus requirements for ORCID iD inclusion in campus scholarly activities like poster sessions. We are also monitoring and reporting enrollment trends.

Poster Number: 85

Time: Sunday, May 5, 3:30 p.m.–4:25 p.m.

Health Literacy Challenges and Opportunities: Bringing Children's Mercy Hospital and Kansas City Together

Jennifer A. Lyon, AHIP, Librarian, Children's Mercy Kansas City, Kansas City, MO; **Angela Knackstedt**, Health Literacy & Bioethics Clinical Coordinator, Children's Mercy Kansas City, Kansas City; **Mamta Reddy**, Medical Director, Quality & Performance Improvement, Children's Mercy Hospital, Kansas City, MO; **Barbra Rudder**, Child Life Manager, Children's Mercy Kansas City, Kansas City, MO; **Courtney R. Butler**, Data Curator, Federal Reserve Bank of Kansas City, Kansas City, MO

Background: A multidisciplinary group at Children's Mercy Hospital applied for and received the NNLM MCR's Immersive Workshop Grant in December, 2017, and used the funding to organize and host a two-day immersive, interprofessional workshop in April, 2018, that 1) brought together health literacy-invested groups and individuals within the Kansas City community to learn about health literacy including cultural, language, numeracy and digital inclusion factors; and 2) provided specialized training to targeted Chjildren's Mercy participants to improve the provision of bedside health information to patients and caregivers. Objectives included: increasing community-wide collaboration, sharing resources, encouraging participants to become change agents, and strategizing organizational initiatives.

Description: The project team consisted of 2 librarians, a practicing physician, a child life specialist, and a nurse educator specializing in equity and diversity; all were past or present members of the institution's Health Literacy Committee. Day One, at the Public Library, was open to the community. Over 25 organizations involved with health literacy and health equity were invited to contribute speakers and participants. Dr. Ruth Parker of Emory University delivered two 'keynote' lectures. Other speakers represented Literacy KC, the Public Library, and an immigration support organization. Day Two was held internally, focusing on Children's Mercy employees professionally invested in promoting health literacy practices in patient care. Additional training on institution-specific resources was supplemented by interactive, hands-on practice sessions and brainstorming exercises intended to develop internal health literacy pilot projects, with encouragement to reconnect with the community organizations.

Conclusion: Day One had 73 attendees (28% from the community). Day Two had 60 attendees. All participants were fully engaged throughout, including interactive brainstorming activities. All informal comments to organizers were positive and the planning team has received requests to repeat the workshop in the future. A survey was sent to all attendees (67-69% response rates). On a Likert scale from 1-5, the means on questions regarding health literacy awareness, motivation to take action, and increased knowledge of resources and collaboration opportunities ranged from 4.88-4.95 for both days. Internal projects are being developed and communication with external groups has steadily increased.

Poster Number: 86

Time: Monday, May 6, 3:30 p.m.–4:25 p.m.

Hinari Workshops in Rwanda: A Training Partnership

Emily J. Glenn, Interim Associate Director, Education & Research Services, McGoogan Library of Medicine, Omaha, NE; **James Edielu Oluka**, Chief Librarian, University of Gitwe, Ruhango District, Rwanda

Background: One librarian from a public academic medical center in the US and one librarian from a private academic medical center in rural Rwanda will lead in-person workshops for several groups of students, researchers, clinicians, and librarians on Hinari Access to Research in Health program and Research4Life resources. The goal of the workshops is to increase access to scientific and health-related digital information in developing countries in part through a "train the trainer" model of instruction. This poster describes the project proposal, project coordination, and the delivery and assessment of workshops held in Rwanda during February 2019.

Description: Capacity building and outreach are critical components of the Hinari Access to Research for Health Program program, which ensures that individuals in organizations with access to Hinari resources can access and use Hinari effectively. Through a grant program, librarians are supported in facilitating Hinari training with partners in developing countries. A project was proposed to provide training in Rwanda for students, faculty, and staff at a university and regional hospitals as well as academic librarians over a period of two weeks. Training materials include modules on information retrieval, information literacy, evidence-based medicine, authorship, and resource advocacy. The impact of training will be assessed via pre- and post-engagement surveys. Anecdotal information will be gathered from participants and the training support team. Library representatives will be contacted after the workshops to ascertain their institution's readiness to deliver training or expand resources.

Conclusion: An assessment of the February 2019 workshops will be derived from the results of pre-and post-instruction assessments of participants, self-assessment of instructors, and anecdotal information. The outcomes we expect to measure across all sessions are increased internet searching skills, increased ability to access different types of Hinari content, increased ability to use the Summon interface of PubMed, and increased awareness of writing and authorship resources. For the "train the trainer" session, we will measure knowledge, engagement strategies, and use of advocacy and marketing strategies for the breadth of items covered in training. New institutional Hinari accounts will also be counted.

Poster Number: 87

Time: Tuesday, May 7, 3:30 p.m.–4:25 p.m.

Health Literacy of Refugee Populations

Margaret Zimmerman, Assistant Professor, School of Library and Information Science, The University of Iowa, Iowa City, IA

Background: Refugees are at a heightened risk for a number of negative health outcomes including diabetes, obesity, and chronic disease. Due to displacement and acculturation stress, refugees are unlikely to be able to seek necessary information and care requisite to good health. The purpose of this project is to pilot a health literacy training for refugee women that will be delivered by library science students. The goal is twofold: to create a replicable curriculum designed to improve health literacy and outcomes of refugee women and their families, and to promote health librarianship and working with disadvantaged populations to library students.

Description: The program was developed by examining scholarly literature, collaborating with experts, and using resources from other programs. Materials were available in English, Spanish, and Swahili. Local refugee resource organizations were contacted to find ten women that were willing to participate. The students were recruited by the project leader from the graduate school where she is faculty. The program will take place during four nights in February. Participants will be pre- and post-tested immediately before and after to demonstrate learning during the project period. They will also be tested six weeks after the program concludes. The participants in the program will be interviewed after the last class and asked for comprehensive feedback on the relevance and helpfulness of the program to their lives. Their feedback will be incorporated heavily into the next iteration of this project, planned for this coming summer.

Conclusion: Participants will be asked to take health literacy evaluations before the course, immediately after, and six weeks later. The goal of the program is for an overall increase in scores of 35 percent at the end of the course and 25 percent to demonstrate retention six weeks later. In addition, participants will be asked which of their health information needs have been met and which have not, which will be a valuable assessment of the efficacy of the program.

Poster Number: 88

Time: Sunday, May 5, 3:30 p.m.–4:25 p.m.

Health Seminars: Supporting Patient Education, Engagement, and Improving Health Literacy

Antonio P. DeRosa, AHIP, Oncology Consumer Health Librarian, Weill Cornell Medicine, New York, NY; **Judy Carol Stribling, AHIP**, Assistant Director, Clinical Services, Weill Cornell Medical College, New York, NY

Background: Some studies suggest a substantial number of patients fail to receive adequate preventive and chronic disease as well as acute care services due to lack of physician time during office visits. Low health literacy remains a problem in the United States yet systematic reviews of health literacy research suggest health literacy interventions are associated with improvements in clinical outcomes. Partnerships between physicians, consumer health librarians (CHL), and other hospital administrators to offer educational seminars on health and wellness topics increases physician-patient face time and helps to address health literacy issues of the patient population at a large academic medical center.

Description: The XXXX Patient Resource Center (PRC) has sponsored a series of public physician-led health seminars featuring topics including state-of-the-art treatment for chronic diseases, preventive measures to delay onset of diseases, general wellness and nutritional guidance, and pain management since its inception in 2007. Marketed to self-selected mailing lists and through flyers distributed around the college and hospital, the seminars attract large audiences. Over three thousand individuals attended 83 health seminars at the PRC since 2014. Realizing the possibility of reaching a wider audience, the managing CHL created a YouTube channel and began posting recorded versions of select seminars in April 2014. The YouTube seminars have global reach and experience a high number of views. As of March 2018, twenty-five videos received 62, 294 views in over one-hundred-eighty-five countries.

Conclusion: The health information seminars in the PRC are a successful service to the medical center community and provide benefits to physicians, CHLs, and patients. The benefits range from improved patient-provider communication and engagement to increased patient knowledge of care plans and informed decision-making. CHLs enjoy partnerships with physicians and gain a better understanding of the patient and community populations served by the PRC. Attendance at the seminars continues to rise, physicians continue to volunteer time and effort to produce quality topics of discussion, and the CHLs remain dedicated to advocating for empowered patients and fighting for health literacy for all.

Poster Number: 89

Time: Monday, May 6, 3:30 p.m.–4:25 p.m.

How Lit Is It? An Analysis of Research Approaches in Health Sciences Libraries

Robin O'Hanlon, Associate Librarian, User Services, Memorial Sloan Kettering Cancer Center Library, New York, NY; **Rachel Pinotti, AHIP**, Associate Library Director, Education and Research Services, Icahn School of Medicine at Mount Sinai, New York, NY; **Barnaby Nicolas, AHIP**, Director, NYU Winthrop Hospital, Mineola, NY; **Judy Carol Stribling, AHIP**, Assistant Director, Clinical Services, Weill Cornell Medical College, New York, NY

Objectives: The nature of library science research has long been a source of interest and debate among information professionals, including variation in health sciences library research approaches. The aim of this study was to classify and categorize health sciences library research by research approach (i.e., qualitative, quantitative, or mixed methods) according to a pre-determined definition over a ten year period.

Methods: A comprehensive search was employed in order to identify all potential research articles published in the health sciences library literature between January 1, 2006 - December 31, 2016 in ten selected journal titles. Journal titles were selected based on relevance within the field, impact factor and searchability via various databases. The search strategy was designed following the Health and Medicine Division of the National Academies of Science, Engineering, and Medicine (formerly known as the Institute of Medicine) Standards for Systematic Reviews. Though not a systematic review, systematic review search practices were employed to ensure a comprehensive search for potentially eligible articles. Studies were then categorized according to research approach or were excluded based upon a list eligibility criteria and definitions of research approaches (i.e., qualitative, quantitative, and mixed methods research approaches) set forth by research scientist John W. Creswell.

Results: After deduplication, a total of 1205 unique records were retrieved. In phase one of the screening (title/abstract), results were positively identified as meeting inclusion criteria, not meeting inclusion criteria, or were referred for phase two full text screening (n=765). Results were only positively identified if all three screeners agreed on categorization and these studies were not subject to further screening. The remaining records were advanced to phase two full text review, conducted by two screeners. The total number of studies from both rounds (n=601) were 446 (74.2%) qualitative, 106 quantitative (17.6%), 49 mixed methods (8.1%).

Conclusions: The high level of qualitative publications in this study deviates from previous findings and may be attributable to an abundance of qualitative case studies, well as large number of studies that employed surveys as research methods which then employed qualitative analysis (i.e., thematic coding). Confusion surrounding publication types (e.g. case studies vs. case reports), combined with the high amount of non-research publications within the search results suggests health sciences information professionals may benefit from increased opportunities for research education and training, specifically in regards to research approaches, research design (i.e., descriptive vs. experimental) and epistemological perspectives.

Poster Number: 90

Time: Tuesday, May 7, 3:30 p.m.–4:25 p.m.

Historical Digital Archives at Saint Joseph Hospital

Karen K. Wells, System Director, SCL Health Library System, Sisters of Charity of Leavenworth Health Care System, Denver, CO; **Dirk Bos**; **Margaret Moylan Bandy, AHIP, FMLA**, Archives Volunteer, Saint Joseph Hospital, Denver, CO; **Ellen Graves**; **Lorene Roth**; **Debra Taylor**

Background: Saint Joseph Hospital in Denver, Colorado was established in 1873 by the Sisters of Charity of Leavenworth, Kansas (SCL). The historical archives was organized in 1978 and is a division within the SCL Healthcare System Library Services. We describe how we are digitizing our collection to preserve and communicate the heritage of our hospital in Denver. The collection contains print and non-print materials, including the 1873 patient record book, photographs, architectural drawings, nursing school uniforms, videos and other items.

Description: The original collection was cataloged on index cards, using vocabulary developed by the Sisters, to locate items that were stored on shelves or in boxes. We now use LC and NLM vocabulary to create records on CONTENTdm software. We do both in-house and outsourced scanning. Our program is under the guidance of an experienced MLS Archivist and we have several former librarians and educator volunteers to assist us. Future projects include the investigation of other software & technologies: ArchivesSpace, Islandora, and hospital data hosting. Our newly funded Architectural Project will allow us to share the records of our various hospital buildings throughout the ages. Records of architects, engineers, contractors, landscape architects, and interior designers are in this compilation. See our evolving digital collection at http://sjharchives.sclhealth.org.

Conclusion: We represent one of the few hospitals across the United States that have an archives. We have received very positive feedback from our hospital leadership, staff, and associates in portraying the perseverance and fortitude of our founders, the Sisters of Charity of Leavenworth, that helped our hospital go from a small cottage to an 8 hospital care site system. We believe that providing this awareness of historicity engenders a sense of understanding between what our associates do now and the implications for our future.

Poster Number: 91

Time: Sunday, May 5, 3:30 p.m.–4:25 p.m.

Impact of the National Institutes of Health's (NIH's) 1993 Enactment of the Public Health Service Act on Demographics Reported in Clinical Trials

Tara Brigham, Assistant Professor of Medical Education & Medical Librarian, Mayo Clinic, Jacksonville, FL; **Hannah Gicalone**, Student, Hematology Oncology; **Taimur Sher**, Associate Professor of Medicine; Physician Consultant in Hematology/Oncology, Mayo Clinic, Jacksonville, FL

Objectives: The aim of the study was to review the prevalence of disparities in the reporting of patients with lymphoid malignancies in phase III clinical trials preceding and following the NIH's 1993 enactment of the Public Health Service Act. This mandate was enacted due to a lack of reporting and/or unsatisfactory inclusion of women and minority demographics in clinical trials.

Methods: A search on the terms Hodgkin Disease, non-Hodgkin, Waldenstrom Macroglobulinemia, and Lymphoma was conducted in Ovid MEDLINE, using both MeSH headings and keywords. The search was limited to articles that either mentioned the words "Clinical Trial, Phase III" in the title or abstract, or were found using the "Clinic Trials, Phase III" publication/ article type filter. Results from this search (314 citations) were imported into a PubMed collection for review.

Implementing the use of Google Sheets, various demographic, geographic, and patient outcome data was collected. Specifically, data on each grouping of patients (Screened Patients, Randomized Patients, Patients receiving the Intervention) was further scrutinized and recorded based on the percentage of Caucasians, African Americans, Hispanics, Asians, other races, Caucasian Females, African Americans Females, Hispanics Females, Asians Females, and other races Females.

Results: Of the 314 citations, 126 clinical trials met study criteria and were reviewed. 114 Non-Hodgkin's Lymphoma (NHL) and 12 Hodgkin's Lymphoma (HL) clinical trials were recorded. The average percentage of women subjects in domestic clinical trials increased from 39% to 41% following the 1993 mandate. As a comparison, women in international trials maintained an average study population of 44%. Trials conducted before the 1993 mandate did not include racial demographic data in the randomized patient population. Following the mandate, 47% of domestic trials and 14% of international trials included racial demographic data for the randomized patient population.

Conclusions: A review of studies focusing on Hodgkin's and Non-Hodgkin's Lymphomas shows a significant lack of demographic reporting. There has been an overall increase in the number of phase III clinical trials published for lymphoid malignancies since the NIH's 1993 mandate. However, that increase does not reflect an increase in gender and race reporting. Journals should mandate the reporting of women and minorities in clinical trials; furthermore, for these differences to be scientifically meaningful, subgroups for race should also be included in reporting.

Poster Number: 92

Time: Sunday, May 5, 3:30 p.m.–4:25 p.m.

Implementing a Digital Experience for the Physical Exhibit: Dental Instruments: Past and Present

Jamie Saragossi, Head of the Health Sciences Library, Stony Brook University, Stony Brook, NY; **Dana Haugh**, Web Services Librarian, Yale University, Cushing/Whitney Medical Library, New Haven, CT; **Jamie Saragossi**, Head of the Health Sciences Library, Stony Brook University, Stony Brook, NY

Background: In 2017, Stony Brook University Libraries secured a NNLM grant in order to digitize and make available a unique collection of dental instruments and associated ephemera from the 18th and 19th centuries. The digital collection was published online in 2018 and features high resolution images, descriptive data, and educational materials designed to enable researchers to study this unique collection from anywhere in the world. The digital collection represents items from numerous personal collections and is meant to preserve this special compilation in perpetuity, as the items will be returned to their respective owners at the conclusion of the physical exhibit.

Description: The grant funds were intended to help us achieve several goals in relation to the collection. The first being continuity, as the collection is comprised of items from multiple donors. Once it is physically dismantled it will now live on in its digital form. The second goal is preservation, as these materials are fragile and many will continue to deteriorate over time regardless of a controlled environment. Third, we wanted to represent dental medicine in the landscape of historical medical archives and digital collections. A large team worked on photographing and scanning each of the items, creating original cataloging and metadata, development of the Omeka platform, programming, and standardization of files.

Conclusion: We continue to promote the use of our digital collection and hope to integrate this with other history of medicine and history of dental medicine collections. The launch of this collection, as well as its promotion and digital exhibit itself, raised the profile of the collection to interested visitors in the Health Sciences and in fields such as History and the Medical Humanities. The interdisciplinarity of this project was crucial to its impact because it appealed to students, scholars, and members of the community interested in various aspects of dentistry, medicine, doctor/patient relationships, material culture, and the history of advertising.

Poster Number: 93

Time: Tuesday, May 7, 3:30 p.m.–4:25 p.m.

Incorporating Alumni Feedback into Nursing Information Literacy Sessions: Time Constraints, Free Resources, and Clinical Cases

Nena Schvaneveldt, AHIP, Education Librarian, University of Utah–Salt Lake City; **Anna Ferri**, Research and Learning Librarian, Roseman University of Health Sciences, Henderson, NV; **Anne R. Diekema**, Assistant Professor / Librarian, Southern Utah University, Gerald R. Sherratt Library, Cedar City, UT; **Elizabeth (Betsy) S. Hopkins**, Nursing and Communication Disorders Librarian, Brigham Young University, Harold B. Lee Library, Provo, UT; **Brandon Patterson**, Technology Engagement Librarian, Eccles Health Sciences Library, Salt Lake City, UT

Background: After conducting research on the information-seeking behavior of practicing nurses who graduated from their universities, librarians at four Intermountain West universities sought to improve information literacy instruction to nursing students based on the research findings. Through collaborations with nursing faculty, librarians have implemented a number of strategies to make information literacy instruction in nursing more authentic, engaging, and effective.

Description: Results from the research provided a number of recommendations for improving information literacy instruction, including introducing time-limited searches with real-life scenarios in information literacy sessions. Online evidence-based practice modules are being developed to replace the ubiquitous research paper. Also, conversations about the study results gave rise to greater understanding and partnership between librarians and nursing faculty. Implementation of concepts from the ACRL Framework for Information Literacy, such as investigations of authority, have been newly introduced so that nursing students can better evaluate a variety of sources they will encounter throughout their careers. To prepare students for a change in access to resources after graduation, librarians have encouraged students to look for available resources in their workplaces. To alleviate concerns about losing access, freely available resources, such as Google Scholar and PubMed, are being taught alongside subscription resources, such as CINAHL.

Conclusion: Initial response to the preliminary implementations has been overwhelmingly positive. Nursing students as well as subject faculty have reported greater engagement with information and increased understanding of the material. Further collaborations are being explored and a follow-up study testing the effectiveness of new instructional approaches is currently in the works.

Poster Number: 94

Time: Sunday, May 5, 3:30 p.m.–4:25 p.m.

Infobuttons in the Electronic Health Record (EHR): Improving Usage and Visibility

Sarah Cantrell, Associate Director for Research & Education, Duke University, Durham, NC; **Megan von Isenburg, AHIP**, Associate Dean, Library Services and Archives, Duke University, Durham, NC; **Beverly Murphy, AHIP, FMLA**, Asstistant Director, Communications and Web Content, Hospital Nursing Liaison, Duke University Medical Center LIbrary & Archives, Durham, NC

Background: Infobuttons are context-specific links from one information system, such as an electronic health record (EHR) to some other resource, such as library resources. Over five years ago, the Library integrated infobuttons into the health system's EHR. The infobuttons primarily link to UpToDate, but a custom configuration enabled providers to access six additional information resources and library services through a small navigation bar. Through Google Analytics, we determined that primarily only UpToDate was being utilized, with minimal hits to other resources. As such, the team hypothesized that the providers were potentially unaware of the other resources and services provided.

Description: Library staff partnered with a team from the health system that included a hospitalist/EHR champion and two MD bioinformatics fellows. Based on the analysis of infobutton tool usage data, the team developed a plan to redesign the results display. This redesign was scoped to include changing the navigation bar behavior, labels and naming, and colors. Librarians on the team developed mockups of potential revisions and authored a usability script to step usability participants through various actions and display options. Usability testing was scheduled to include various provider types, including representatives from nursing, residency programs, medical school, and physician groups. Usability tests were captured through Morae software and extensive notes.

Conclusion: Conclusions will be shared after usability testing is completed and results are analyzed.

Poster Number: 95

Time: Monday, May 6, 3:30 p.m.–4:25 p.m.

Integrated Research Data Management Training for Medical School Faculty and Residents: A Program Evaluation

Joanna Thielen, Research Data and Science Librarian, Oakland University Libraries, Rochester, MI; **Stephanie M. Swanberg, AHIP**, Associate Professor, Information Literacy & eLearning Librarian, Oakland University William Beaumont School of Medicine, Rochester, MI

Background: Research data management (RDM) is a combination of practices that make data easier to locate, use, analyze, and share. However, RDM is not often taught in undergraduate and graduate education programs. Many health sciences libraries have started to offer RDM as part of their services, including education, but it remains a challenge to expand library services in already busy libraries. In a collaboration between a medical librarian and a research data librarian at the university library, an introductory training session on RDM was integrated into the Fellowship in Medical Education (FME) program sponsored by the medical school.

Description: The FME program is a year-long faculty development program focused on medical education research and provides monthly instruction sessions and a small stipend to each participant to develop an education research project. The integrated RDM workshop developed by the authors combined didactic lecture with small group discussion and active learning exercises. A pre- and post-workshop evaluation was administered to participants to gauge their RDM training experience and comfort level in RDM principles and practices as well as evaluate the workshop. The RDM session was offered in 2017 - 2018 in the FME program and 2018 - 2019 as part of a combined FME and resident teaching and research program. Due to changes in the program structure between the two years, the session was reduced from a three-hour interactive workshop to a one-hour primarily didactic lecture with homework the second year.

Conclusion: The results of the post-session assessment (n = 5 in 2017 - 2018; n = 3 in 2018 - 2019) indicated that attending the workshop increased participants' comfort with organizing data, storing data, and writing data management plans. However, the reduction in time impacted participants' ability to apply the information. This collaboration between the medical and university libraries has proven effective in offering RDM education opportunities without significantly increasing workload. This poster will review the design, implementation, and assessment for this workshop and conclude with practical suggestions for offering this type of training to researchers at other institutions or hospitals.

Poster Number: 96

Time: Tuesday, May 7, 3:30 p.m.–4:25 p.m.

Involvement of Information Professionals in Patient- and Family-Centered Care Initiatives: A Systematic Review

Antonio P. DeRosa, AHIP, Oncology Consumer Health Librarian, Weill Cornell Medicine, New York, NY; **Becky Baltich Nelson**, Clinical and Systems Librarian, Weill Cornell Medicine, Brooklyn, NY; **Diana Delgado, AHIP**, Associate Director, Information, Education and Clinical Services, Weill Cornell Medicine, New York, NY; **Keith C. Mages, AHIP**, Clinical Medical Librarian, Weill Cornell Medicine, New York, NY; **Lily Martin**, Health Sciences Librarian, North Shore University Hospital, Brooklyn, NY; **Judy Carol Stribling, AHIP**, Assistant Director, Clinical Services, Weill Cornell Medical College, New York, NY

Objectives: The goal of this systematic review was to collect data on patient- and family-centered care (PFCC) programs and initiatives that have included the direct participation of a clinical medical librarian or other information professional.

Methods: Systematic literature searches were conducted in seven scholarly databases in the information, medical, and social sciences. The criteria for inclusion of studies contained two elements: (1) description of patient-centered initiatives or projects presented, explicitly, as PFCC programs; and (2) information professional/librarian involvement in the PFCC initiative or program. Based on the definition of PFCC provided by the Institute for Patient- and Family-Centered Care (IPFCC), we developed a custom code sheet to organize data elements into PFCC Categories and Outcomes. Other extracted data elements included how the information professional became involved in the program and a narrative description of the interventions/process presented.

Results: Consistent with the values of PFCC, each included study (n=12) identified patient education and/or information sharing as an integral component of their patient-centered initiatives. Librarians were noted to contribute towards shared decision-making through direct patient consultation, the provision of education on health literacy, and through information delivery to both provider and patient with the goal of fostering collaborative communication.

Conclusions: The synthesis of available evidence to date suggests that information professionals should focus on patient education and information sharing to support both patients/caregivers and clinical staff. The burgeoning efforts in participatory care and including patients in the decision-making process pose a unique opportunity for information professionals to offer more personalized and value-based information services.

Poster Number: 97

Time: Sunday, May 5, 3:30 p.m.–4:25 p.m.

Is Learning Actually Happening? Investigating Course Outcomes beyond the Traditional Evaluation

Bobbi Newman, Community Outreach & Engagement Specialist, National Network of Libraries of Medicine - GMR, Iowa City, IA; **Rachel Gatewood**, Instructional Designer, Greater Midwest Regional Library & NNLM Training Office

Background: A consumer health continuing education course for public librarians included an evaluation but there were no additional opportunities for follow-up to explore actual impact. To address this, a three-month post-course follow-up was designed to explore the following; was the learning shared with colleagues and or patrons, what new actions had been taken in the library because of the course, and why did some not complete the course? This follow-up questionnaire generated data about practical course value, led to a better understanding of course completion influences, and provides a model for other course developers and continuing education granting organizations.

Description: The questionnaire was developed through collaboratively identifying what information was desired, what was missing in the current course evaluation, and building a process for implementation and review. Through brainstorming, reviewing the current evaluation data, and processing what could realistically be explored, a draft version of a follow-up questionnaire was created. The draft was shared with the organization's evaluation team for feedback. A final version was created and emailed to the most recent cohort of course registrants. With no issues detected and responses that proved valuable, the questionnaire was sent to two previous cohorts as well. It continues to be sent to all course registrants, for both online and in-person versions of the course, three months after completion. Responses are regularly explored and analyzed for information that supports course development, delivery, and public librarian health information knowledge.

Conclusion: Currently, the questionnaire has been delivered to 402 individuals with 209 responses (52%). Key findings include:

80% shared a resource with a fellow staff member, almost 14% plan to; 51% developed new programming / outreach; and 100% of respondents who completed at least one week indicated learning about a new resource.

By implementing a delayed follow-up questionnaire, it is possible to explore learners perceptions of the value of a course, the scope of activities attributed to the learning and discover how to better support learners to meet their needs and support course completion.

Poster Number: 98

Time: Sunday, May 5, 3:30 p.m.–4:25 p.m.

Knowing and Doing: Quantitative and Qualitative Exploration of Wellness Behaviors among Health Sciences Librarians

Susan Keller, Librarian, Children's National Medical Center, College Park, MD; **Layla Heimlich**, Medical Librarian, MedStar Washington Hospital Center, Bethesda, MD; **Fred King**, Medical Librarian, MedStar Washington Hospital Center, Silver Spring, MD; **Jory Barone**, Medical Librarian, Medstar Washington Hospital Center, Washington, DC

Objectives: The connection between abundant and easy access to information among health science librarians and the practice of wellness behaviors is unknown. This projects looks at the behaviors of both health science and non-health science librarians in order to answer the question: "Is having access to high-quality health information associated with a high level of wellness behaviors among health science librarians?"

Methods: After consultation with a biostatistician and approval by the Institutional Review Board, we designed a survey consisting of seven demographic items, two items on workplace support for wellness behavior (e.g., vaccines, wellness programs), three items to assess access to health information and awareness of healthy behavior recommendations, seven specific healthy behavior practice items, and three open ended questions.

We contacted 88 library organizations and 49 agreed to help us distribute our survey. These organizations represented public, school, academic, and health science libraries. We also posted the survey on the MEDLIB-L (Medical Library Association Listserv), Special Library Association Listserv, as well as seven SLA and MLA special interest groups. We opened the survey on March 7, 2018 and closed it on April 25, 2018.

Survey analysis tools included RedCap, SAS 9.4, and Dedoose.

Results: Quantitative Results Summary:

1913 librarians responded to the survey: 663 health science librarians (HSL) and 1250 non-health science librarians (Non-HSL).

• We found NO significant demographic differences between the two groups

• HSL are MORE likely than Non-HSL to eat 5 or more fruits/vegetables per day (p value 0.031)

• HSL were MORE likely than Non-HSL to exercise moderately (p value <0.001)

• HSL were LESS likely than Non-HSL to engage in prayer or other spiritual practices (p value 0.009)

Using Dedoose, we are identifying themes concerning the barriers, facilitators, and connections between the profession of librarianship and wellness behaviors.

Conclusions: Overall, health science librarians have more access to quality health information and have healthier eating and exercise behaviors, but spend less time in prayer and other spiritual activity than non-health science librarians.

We expect to gain more insight into the barriers and facilitators, as well as the connections between the profession of librarianship and the practice of wellness behaviors, after analyzing the qualitative results from the survey.

Poster Number: 99

Time: Tuesday, May 7, 3:30 p.m.–4:25 p.m.

Learning from Lates!

Patti E. Biggs, Information Services Specialist, Library and Information Services, London, England, United Kingdom

Background: Research institutions can promote science to the public through Lates. These are evening events where current research is presented using fun activities. Our library team has participated in two such events and learnt from them.

Description: Lates events present science in a fun and non-threatening way through quizzes, games, crafts, demonstrations and lightening talks. Researchers and support teams interact directly with the public. Our preparation for the events involved discussions with our public engagement team who helped us frame our ideas into activities. The public engagement team challenged us to think differently about presenting information to people, giving feedback on our rehearsals and lots of encouragement as this was very new to the library team. We found that the audience was receptive to information on scientific publishing and open access.

After the event we decided to use some of the same techniques to promote the library and its services to our clientele. Using theatrical flourishes has given our information greater impact.

Conclusion: Using theatrical props has been a great way to start conversations, both with individuals and groups. We have learnt not to be afraid of trying different techniques to get the messages we want to share across to users. Developing props takes time, but does make us focus on the information we wish to convey. We now make more use of give-away items to keep our brand in peoples' minds. This has made promoting library services more interesting and rewarding for the staff involved.

Poster Number: 100

Time: Sunday, May 5, 3:30 p.m.–4:25 p.m.

Learning Process of Medical Informatics Assisted by Electronic Evidence-Based Medicine Resources

Hui-Chin Chang, Director / lecturer, Library, Chung Shan Medical University Hospital / School of Medicine, Chung Shan Medical University, Taichung, Taiwan (Republic of China); **Kuo-Shu Huang**, Associate Professor, Chung Shan Medical University / Institute of Medicine, Taichung, Taiwan (Republic of China); **Hui-Ying Low**, Department of Obstetrics and gynecology, New Far Eastern OB/GYN Hospital, Taichung, Taiwan (Republic of China); **Tzu-heng Chiu**, University Librarian, Taipei Medical University, Taipei, Taipei, Taiwan (Republic of China); **Kevin Ma**, Researcher, University of Pennsylvania

Objectives: The innovative basic medical curriculum for 2nd year medical students has been developed and given by a qualified faculty-clinical medical librarian who has professional knowledge and practical experience in PBL (Problem-based learning) and EBM (Evidence-based medicine). The librarian is responsible for the teaching of the entire semester course and designs a systematic medical information application course.

Methods: The 2nd year preclinical medical students (N = 111) consists of 16 sessions, with 2 hours for each session. In the beginning of the semester, oral presentation of clinical scenario was required, following 11 sessions of introduction of professional search strategy and library resources. After the lectures, the students were required to present the scenario again, applying the tough resources.

The objectives were to assess participant performance outcomes involving capability to utilize the class-mentioned resources to describe assigned clinical scenarios in terms of lesion anatomy and disease pathogenesis, and then, present the above contents consisting of organized structure, correct citation of the references in the form of case reports. This task, along with questionnaires focusing on perceptions and practices of applying these resources in real-life, was issued before and after the course to observe the helpfulness of the course.

Results: The enrolled medical students acquired improved abilities ($P < 0.05$) in that before they could only search Google for medical information, now they can exploit EBM database and filter the searching results. Also the students reported higher frequency of referring to journal articles in their work ($P < 0.05$).

Instead of the elevated frequency of electronic resources utilizing habits, significantly, the improvement of capability outcomes is associated with acquired cognitions regarding usable electronic academic resources in libraries ($P < 0.05$), and the need of taking training courses to utilize which ($P < 0.05$).

Conclusions: Basic training to utilize medical information for pre-clinical students is necessary for scenario-based medical education, establishing literacy and cognition of searching high level journal articles and EBM database. The key regulator in the helpful mechanism of which may be induced individual eager to apply library electronic resources. Notably, taking lecture notes or volunteering as teaching assistant (TA) itself wouldn't cultivate EBM awareness, or further facilitate utilization of related resources in clinical scenarios. Awareness and use of medical library in PBL and EBM is an asset not a burden.

Poster Number: 101

Time: Monday, May 6, 3:30 p.m.–4:25 p.m.

Librarian Feedback Loops Improve Medical Student Self-Directed Learning

Elizabeth Suelzer, AHIP, User Education and Reference Librarian, Medical College of Wisconsin, Milwaukee, WI; **Johnathon Neist**, Medical College of Wisconsin, WI; **Robert Treat**, Associate Professor and Director of Measurement and Evaluation, Medical College of Wisconsin, Milwaukee, WI

Background: Self-Directed Learning (SDL) opportunities are being integrated into the curriculum at the Medical College of Wisconsin. One component of the SDL process, as defined by the Liaison Committee on Medical Education (LCME), is for students to receive feedback from faculty/supervisors on their information-seeking skills. Librarians, being information experts, are well equipped to provide feedback to students and were tasked with doing so through a workgroup early in the curriculum process. Librarians are currently providing feedback for students on their SDL projects and we want to determine if this feedback leads to improvements in students' information seeking behavior skills.

Description: All students in MCW's medical school participates in at least two SDL projects per year in their M1 and M2 school years. Some of the SDL projects involve librarian feedback that aim to improve student resource gathering on different topics of their choosing.. Librarians provide feedback with a rubric that can be used for any SDL project. Not all the SDL projects involve librarian feedback, so some students have yet to receive librarian feedback, while others have received feedback several times over their first 2 years. Librarian feedback is informational only and does not count toward the final grade of the project. One early trend, backed by a large effect size, shows that students receiving feedback on a 1st-year SDL project tended to improve in a 2nd-year SDL project.

Conclusion: Now in its 2nd year, this quality improvement program is improving how students engage with the use of medical literature. Early assessment of the SDL program shows an improvement in student skills, particularly as students re-evaluate what resources to use in a given project, in the progress made from 1st year to 2nd-year. The measurement and evaluation team is aiding the library and instructional design teams to MCW in hopes of improving the SDL activities as they evolve.

Poster Number: 102

Time: Tuesday, May 7, 3:30 p.m.–4:25 p.m.

Library Activity Dashboard Linked to Missions: Using Data to Communicate with Staff and Leadership about How the Library Supports Education, Research, and Clinical Care

Gretchen Naisawald Arnold, Director, University of Virginia–Charlottesville; **Abbey Elizabeth Heflin**, Analytics and Discovery Services Manager, University of Virginia Health Sciences Library, Charlottesville, VA

Background: With the launch of a new university budget model, it became important that the Health Sciences Library be able to capture not only who was using its resources but to also be able to better align use with the institutional missions - education, research and clinical care. Demonstrating how the Library directly aligns to the work of the Health System creates a better understanding of the Library's impact.

Description: All major units within the Health System have been asked to develop operational dashboards that can display current significant metrics as well as document historical trends. To the extent possible, these dashboards should approximate real time activity which in most cases will be daily counts. The HSL took an additional step in attempting to capture how services and resources were used to support large institutional missions. While research and education are traditionally areas of heavy library use, the dashboard also illustrates that use of the library by the clinical operation is on the increase. To heighten staff communication and understanding of the work, regular internal infographics called Weekly Impact Reports are sent to all staff so that everyone has an easy snapshot of what is happening in the Library.

Conclusion: The dashboard, which is publically available from the Library's website, continues to be a work in progress. All ready it has been useful in helping leadership gain an appreciation of what a modern academic medical library does. It helps build the story that the library brings value in ways that are not immediately apparent yet have a direct bearing on the success of all the missions of the institution.

Poster Number: 103

Time: Sunday, May 5, 3:30 p.m.–4:25 p.m.

Library Lockdown: Elevating Instruction through an "Escape Room" Experience

Rachel Helbing, AHIP, Director of Library Services for the Health Sciences; **Stefanie Lapka**, Health Sciences Librarian; University of Houston Health Sciences Library, Houston, TX

Background: An escape room is a live-action game which consists of a series of puzzles. Participants follow clues and progress through a predefined scenario in order to achieve their goal and 'escape.' Conducting an escape room activity in the learning environment is a newer strategy in the health sciences. This escape room activity was unique in that it was facilitated in a large optometry community health course, utilizing existing space and personnel, and minimal supplies.

Description: This learning activity was designed to reinforce information literacy and research skills, and took place over two sessions. In the first, students participated in one hour of in-class instruction. This prepared them with the necessary background information to enable successful completion of the game. In the second session held three days later, students participated in the escape room activity. This consisted of working in teams to complete a variety of tasks, including both paper and web-based puzzles, that tested knowledge and skills related to evidence-based practice. Two librarians and one optometry faculty member served as game facilitators. Teams that completed all game tasks in sequence and obtained the code to unlock a lock box within 45 minutes were considered to have successfully 'escaped.'

Results: A total of 16 groups (95 students) took part in this activity, and all were able to finish within the allotted time. Mean time to 'escape': 32 min 57 s; First place: 19 min 57 s; Last place: 44 min 0 s.

Discussion:

- Allow sufficient time to plan all of the activities - Six weeks for the first event; One week for subsequent events.
- Utilize favorite tools for web-based clues - Google Forms, LibWizard, Qualtrics, etc.
- Test locks and clues for accuracy.
- Start with a simple task to build students’ confidence.
- Plan for potential crowding and noise.
- Allot enough time for the event - 30 minutes for setup; 60 minutes for gameplay.
- Provide assistance as needed when groups get stuck - Add time penalties for hints given.
- Give curricular incentives for thoughtful participation - Performance-based grades are ideal.

Poster Number: 104

Time: Monday, May 6, 3:30 p.m.–4:25 p.m.

Library Collaboration with Faculty Members for Creating ORCID Profiles

Sachie Shishido, AHIP, Reference Librarian, Massachusetts Eye and Ear, Boston, MA

Background: ORCID (Open Researcher and Contributor ID) is a registry of unique identifiers for researchers, analysts, and scholars. It can be linked up to and used by publishers, databases, and organizations. Today, many publishers and funders are integrating ORCID iD into their application processes and reporting workflows. The Department of Ophthalmology of a medical school has decided to require faculty members to have an ORCID profile when they are going through the promotions or reappointments process. This article describes how the library collaborated with the department to facilitate the creation of ORCID profiles for faculty members.

Description: The library created a tutorial that explains step-by-step instructions to create an ORCID account, add references, and grant permission to trusted individuals. An email with the tutorial link along with the librarian's contact information was sent to all faculty members on November 15, 2018. Twenty-two members contacted the librarian regarding their ORCID profiles. Another email was sent to each of them with four questions in order to determine the best way to complete their profiles. Eighteen members requested to add their references and granted permission to the librarian to edit their profiles. The ways to complete their list of works in their ORCID profiles included exporting a list of references from their My Bibliography or SciENcv on their My NCBI accounts, importing the results from Author Search on Web of Science, and using Crossref through ORICD's Search & Link feature.

Conclusion: The process of completing faculty members' ORCID profiles varied depending on the uniqueness of their last name, length of their career, and the way they had been organizing their own references. The librarian was able to deepen the knowledge of author identification systems, develop a better tutorial based on the questions that were asked during this project, and improve customer services by understanding the needs and challenges faculty members may have. Moreover, this collaboration reaffirmed to the community the value of partnering with the library, the library's commitment to service, and its resources that can be widely utilized.

Poster Number: 105

Time: Tuesday, May 7, 3:30 p.m.–4:25 p.m.

Long Night against Procrastination

Alyssa Grimshaw, Access Services/Clinical Librarian, Cushing/Whitney Medical Library, West Haven, CT

Background: To create a fun and productive atmosphere to help students fight procrastination and get work done before finals week.

Description: Each semester, the library hosts the Long Night Against Procrastination (LNAP) the week before finals. The event runs from 7pm-12am and students are asked to check their cell phones at the door. Every 90 minutes, we interrupt the students and serve them snacks and offer wellness activities, this helps break up the night and helps recharge them to stay productive. The evening librarian is available for last minute consultations to assist the students finishing last minute assignments.

Conclusion: Each semester offered, LNAP has had a full registration and has been met with enthusiasm from our students. It has lead to many discoveries in study trends of our different schools. LNAP has helped us directly assist the students, both be productive and study and relax and have fun! We look forward to continuing the event in the upcoming years!

Poster Number: 106

Time: Tuesday, May 7, 3:30 p.m.–4:25 p.m.

Looking Back to Move Forward: A Reflection of a Section's History

Nicole Theis-Mahon, AHIP, Liaison to the School of Dentistry & Collections Coordinator, University of Minnesota–Minneapolis; **Nena Schvaneveldt, AHIP**, Education Librarian, University of Utah–Salt Lake City; **Amanda Nevius**, Research & Instruction Librarian - Dental Liaison, Tufts University, Boston, MA

Background: The history of the Dental Section illuminates our own history as communities of librarians with the Medical Library Association (MLA). Reflections on a community's history can reveal growth, development and changes, or shed light on similarities between the past and present. Knowledge and awareness of a community's past can be used to guide members through change.

Description: We examined historical documents from the Dental Section and conducted interviews with community members to generate a history of the community within the broader context of medical librarianship. To add context, a general history of dentistry and its relationship to medicine was also conducted. Themes between dental and medical librarianship and between dentistry and medicine were identified and described.

Conclusion: The history of a community of librarians is important to understand future directions of MLA, and is informed by the history of the practitioners the librarians serve.

Poster Number: 107

Time: Monday, May 6, 3:30 p.m.–4:25 p.m.

Managing Multiple Systematic Reviews: Recommended Practices and the Systematic Review Tracking Checklist

Q Eileen Wafford, Research Librarian, Galter Health Sciences Library and Learning Center, Northwestern University, Chicago, IL; **Peggy Murphy**, Librarian, Lurie Children's Hospital, Chicago, IL; **Pamela L. Shaw**, Biosciences & Bioinformatics Librarian, Northwestern University, Chicago, IL; **Jonna Peterson**, Senior Clinical Informationist, Northwestern University, Chicago, IL

Background: As more and more researchers incorporate librarians and informationists in the systematic review process, librarians find themselves working on multiple reviews while performing the wide range of other duties related to their positions. This project aims to test, assess, and refine recommended practices and the Systematic Review Tracking Checklist as a system for managing multiple systematic reviews.

Description: We will evaluate our systematic review workflow and identify content shared in each review. We will establish templates for frequently communicated messages. We will solicit advice from information professionals with expertise in data management to establish best practices for naming and organizing files and folders. We will develop and incorporate the Systematic Review Tracking Checklist into our workflow. We will recruit 3-5 librarians from academic and hospital libraries and learning centers who work on multiple systematic reviews. Each librarian will employ the Systematic Review Tracking Checklist and implement the recommended best practices into their workflow. We will survey each librarian after three, six, and nine months. We will assess the feedback and refine the tracking form and recommended practices as needed.

Conclusion: By incorporating our system of recommended practices and the Systematic Review Tracking Checklist, librarians working on multiple reviews should become more efficient and effective at organizing information as well as in planning and controlling the workload from systematic reviews. As a result, librarians will be better able to manage reviews while accomplishing other work-related tasks.

Poster Number: 108

Time: Tuesday, May 7, 3:30 p.m.–4:25 p.m.

Measuring the Impact of a Systematic Review Service

Jennifer DeBerg, User Services Librarian, Hardin Library for the Health Sciences, Iowa City, IA; **Heather Healy**, Clinical Education Librarian, University of Iowa Libraries, Iowa City, IA; **Matt Regan**, Clinical Education Librarian, University of Iowa–Iowa City; **Chris Childs**, Clinical Education Librarian, University of Iowa–Iowa City

Background: In 2010, a systematic review service was launched at a health sciences library to support improvement of adherence to standards. This service, though popular and in a state of continual development, has not been formally evaluated at our institution. The intent of this project is to share our process for measuring the value of our program. Specifically, we will determine whether the establishment of this service has increased librarian authorship or acknowledgement in systematic reviews or meta-analyses published by affiliated authors at this institution. Another objective is to evaluate impact on the quality of reporting.

Description: CINAHL, Embase, Scopus, and PubMed searches will be conducted for the past 10 years to ensure capture of systematic reviews or meta-analyses published while this service has been available. Publications will be evaluated for local librarian author inclusion or acknowledgement, adherence to PRISMA reporting standards, and quality of the search methodology. Results will be examined by subject specialty in order to learn more about needs of researchers and to assist with focusing outreach efforts.

Conclusion: Assessment of the quality of the methods of published systematic reviews at an institution with a well-established systematic review program will facilitate librarians' and administrators' decision-making about future priorities for systematic review services.

Poster Number: 109

Time: Sunday, May 5, 3:30 p.m.–4:25 p.m.

Medical Librarians and Faculty Collaborate to Develop a Dissemination Tool for Precision Medicine

Reina Williams, AHIP, Reference Librarian and Education Coordinator; **Raj C. Shah**, Associate Professor, Family Medicine and Rush Alzheimer's Disease Center; **David J. Sedillo**, Project Manager; Rush University Medical Center, Chicago, IL

Background: Purpose: Dissemination science focuses on ways to narrow the delay between the elucidation of new health concepts through research and use in learning health systems. Precision medicine (defined as an emerging approach for disease treatment and prevention that takes into account individual variability in genes, environment, and lifestyle for each person) is one such innovative concept that is diffusing into daily education curricula and clinical practice. This poster examines the partnership between medical librarians and faculty to develop a dissemination tool for precision medicine that can be utilized for student and clinicians.

Description: Setting/Participants/Resources: The librarians of the Library of Rush University Medical Center have developed a partnership with its faculty and staff at the Rush Alzheimer’s Disease Center.

Brief Description: The Rush University Medical Center is a member of the Illinois Precision Medicine Consortium, which is working with the National Institutes of Health (NIH) and the All of Us Research Program. The Rush University librarians, and faculty and staff of the Rush Alzheimer’s Disease Center developed a precision medicine online resource guide using LibGuides, a content management system, to support student and faculty learning at Rush University Medical Center. This poster describes the content and development of the precision medicine guide, the delivery of this content, and preliminary uptake.

Conclusion: Results/Outcomes: Fourteen topics related to research and precision medicine research were combined to create an online library guide. After launching the resource in October, 2018, 185 users have accessed the resource with the most common pages examined being ‘What is precision medicine?,’ ‘All of Us Program,’ ‘Precision Medicine and Genetic Testing,’ and ‘Precision Medicine Websites.’

Discussion/Conclusion: The LibGuides platform provides an unique opportunity for medical librarians and faculty to collaborate to promote rapid dissemination of new health concepts to faculty and students at an academic medical center. Further work to optimize the resource is ongoing.

Poster Number: 110

Time: Monday, May 6, 3:30 p.m.–4:25 p.m.

Moving Beyond: How the Health Information Center Evolved to New Marketing Trends

Kelsey Grabeel, AHIP, Assistant Director of the Health Information Center, University of Tennessee Graduate School of Medicine / University of Tennessee Medical Center, Knoxville, TN; **Rachel E. Roberts**, Administrative Coordinator, University of Tennessee–Knoxville; **Jennifer Luhrs**, Library Supervisor, UT Graduate School of Medicine, Knoxville, TN; **Martha Earl, AHIP**, Director/Associate Professor, University of Tennessee–Knoxville

Background: To demonstrate the changes implemented in the Health Information Center's (HIC) marketing plan to further engage patients, family members, and the community at an academic medical center. After being open for four years, the HIC elevated their marketing strategy further to bring the community into the library as well as take the library into the hospital community.

Description: The Assistant Director and outreach staff formed the HIC Engagement Task Force to focus specifically on the new direction and changes made to the marketing strategy. One initial step involved the task force meeting with the hospital marketing department to change the HIC's website design. Another step centered on improving the library's physical atmosphere by adding a display table at the entrance, a new display chalkboard outside the door, and seasonal decorations. The team received permission from hospital marketing to use a video monitor to advertise library services and engage patrons with healthy tips related to monthly health observances. In addition, the team developed the Traveling Health Information Center to bring the HIC to waiting rooms throughout the hospital.

Conclusion: The marketing changes increased HIC visibility both physically and virtually. The website changes increased the average number of website users per month from 283 to 849. The book display, chalkboard, and slides prompted positive informal feedback. Circulation of HIC books increased 41%. The Traveling HIC netted 25 requests from Cancer Institute's waiting room clientele. In Day Surgery, Surgery, and NICU waiting rooms, the staff answered 50 questions. The Traveling HIC helped 70 patrons at the local Farmers Market. The updated marketing plan integrated the HIC even more firmly within the hospital's culture and furthered the library's reach within the community.

Poster Number: 111

Time: Monday, May 6, 3:30 p.m.–4:25 p.m.

Multi-State Salary Survey 2019

Stephanie Friree Ford, Manager, Library Resources, McLean Hospital, Belmont, MA; **Meredith I. Solomon, AHIP**, Outreach Officer, Harvard Medical School, Boston, MA; **A'Llyn Ettien, AHIP**, Collections Management Librarian, Boston University Medical Library, Malden, MA; **Lisa A. Adriani, AHIP**, Research and Instruction Librarian, Edward & Barbara Netter Library, Hamden, CT; **Karen Sue Alcorn**, Reference and Instruction Librarian, MCPHS University, Worcester, MA; **Jessica Kilham, AHIP**, Manager, Library Education and Clinical Services, University of Massachusetts Medical School, Worcester, MA; **Lisa Liang Philpotts**, Knowledge Specialist for Reseach & Instruction, Massachusetts General Hospital, Boston, MA

Objectives: This survey examined health science librarian salaries within a six-state region. Survey results were compared to previous state and national data.

Methods: A survey tool adapted from a 2013/2014 survey was distributed through regional librarian listservs.

Results: 106 librarians from the six states in the target region responded to the survey: a 42.91% response rate. The majority came from Massachusetts and Connecticut. These two states have the most health sciences libraries, meaning that averages for other states are based on more limited data. Responses came almost equally from academic(54) and hospital(45) librarians, with 7 "other" institutions. AHIP membership was relatively low, 27.36%, and strongly correlated with the perception that institutions valued it. Most institutions offer some support for professional development: partial support has decreased compared to the 2014 survey, while full support and no support have increased. We inquired about hours worked weekly and results were divided into two categories: full-time and part-time. 84.91% of respondents reported working full-time. Part-time positions were largely solo librarians (7/16) with the rest varying: 1 systems librarian, 4 reference librarians, 4 director/manager-level. The average salary for full-time librarians was $75,944 and $67,685 for part-time librarians. Part-time positions are most common in hospitals (13/16). Average salary for the entire region across all library types and positions was $71,519.

Conclusions: Position and location are both significant. There is a large difference between the average salary of department heads and systems librarians, respectively making the highest and lowest annual salaries. At the same time, average salaries for each position type vary by state, and don't necessarily reflect trends for overall average salary. For example, although Massachusetts medical librarian salaries ranked second highest overall among states in the New England region, director salaries ranked only fourth highest, and it's possible that these relatively low director salaries may have skewed the total average salary for medical librarians in Massachusetts. Comparing librarians to all occupations nationally, the profession is not keeping pace with the average in terms of annual salary increases in CT or nationally and is almost stagnant in MA, while the rest of New England is doing even less well. In the six-state region surveyed, MA is still doing slightly better than the national average for librarians, while CT is doing significantly better. Limited responses from other states means that the apparent poor results may or may not reflect actual average salary increases for these librarians.

Poster Number: 112

Time: Sunday, May 5, 3:30 p.m.–4:25 p.m.

Nursing Students' Experience in Acquiring and Using Information Literacy Skills

Darlene Parker-Kelly, Director of Library/LRC, Charles R. Drew University Health Sciences Library, Los Angeles, CA

Background: Information literacy skills are adopted by library associations, librarians, and many societies/associations. The teaching of information literacy skills is an integral part of library instruction and yet there are gaps in the literature on how students learn these skills. Recent studies have indicated that nursing students have difficulty acquiring and applying information literacy skills in their courses and subsequently in practice. The purpose of this study was to gain insight through the nursing student's perspectives on how they experience information literacy skills.

Description: A qualitative research design was employed and a purposeful sample of 9 students and 3 faculty in a nursing educational program participated in the study. The study sought to answer the following research questions: (1) How are information literacy skills acquired and applied through library instruction in accelerated nursing program? (2) How do nursing students describe their level of confidence in transferring information skills they obtain during library instruction? and (3) What aspects of library instruction were helpful or problematic in gaining and applying information literacy skills?

Conclusion: Students indicated library instruction was critical to learning how to use information literacy skills. In addition, students described real life experiences in adopting information literacy skills including: (1) a time lapse in instruction and assignments; (2) the need for refreshers, either in person or virtually; and (3) challenges locating articles. These insights will assist in improving the method of teaching information literacy skills in the future.

Poster Number: 113

Time: Monday, May 6, 3:30 p.m.–4:25 p.m.

Outreach Strategies and Researchers' Motivations for Sharing Data through a Data Catalog

Melissa Ratajeski, AHIP, Coordinator of Data Management Services, University of Pittsburgh, Pittsburgh, PA; **Katherine Goold Akers**, Biomedical Research & Data Specialist, Wayne State University, Detroit, MI; **Nicole Contaxis**, Data Catalog Coordinator, NYU Health Sciences Library, New York, NY; **Megan Del Baglivo**, Metadata Librarian, Univ. of Maryland, Baltimore, Health Sciences & Human Services Library, Baltimroe, MD; **Na Lin**, Head of Resource Development and Sharing, University of Maryland Baltimore, Baltimore, MD; **Helenmary Sheridan**, Data Services Librarian, University of Pittsburgh, Pittsburgh, PA; **Kevin Read**, Lead of Data Discovery/Data Services Librarian, NYU School of Medicine, New York, NY

Background: The Data Catalog Collaboration Project's (DCCP) mission is to facilitate the discovery of hard-to-find biomedical research data and to reduce perceived barriers and burdens of data sharing. The DCCP currently consists of eight academic health sciences libraries, five of which have active local instances of the open source data catalog. Together, DCCP institutions communicate and share strategies for outreach initiatives and establishing partnerships to populate our individual data catalogs. Through these efforts the DCCP has identified several reasons why researchers opt to use the data catalog model to share their data.

Description: Some successful outreach strategies DCCP members use to populate their individual data catalogs include: (1) Searching PMC for articles with data availability statements; (2) Communicating with deans/department heads; (3) Giving presentations or hosting informational tables at campus data-related events; and (4) Partnering with other research data stakeholders on campus (e.g., Institutional Review Board). As each DCCP institution engages with researchers and other stakeholders, they document comments from researchers on why they may or may not choose, to index their research data in the data catalog. These interactions are shared across the DCCP to identify themes for more meaningful and persuasive outreach messaging and to develop new strategies to strengthen DCCP efforts.

Conclusion: To date, over 70 interactions have been documented by DCCP members. For researchers who agree to have their datasets described in their institution's data catalog, emerging themes include: to find collaborators, to increase visibility of their research, to promote data re-use, and because the data catalog is a low-burden way to share their data. Moving forward, these themes will be used to improve outreach strategies, evaluate changes to data catalog functionality, and identify methods for the DCCP to seamlessly integrate data sharing via the data catalog into existing researcher workflows

Poster Number: 114

Time: Sunday, May 5, 3:30 p.m.–4:25 p.m.

Passing the Test: Collecting Constructive and Implementable Patient Feedback About Patient Education

Ruti Volk, AHIP, Lead, Patient Education and Health Literacy Program, Michigan Medicine, ann arbor, MI; **Karelyn Munro**, Patient Education Resources Coordinator, Michigan Medicine, Ann Arbor, MI; **Amy Hyde**; **Yvette Salamey**; **Julie Wietzke**, Administrative Manager, Office of Patient Experience, Michigan Medicine, Ann Arbor, MI; **Quinlan Davis**

Background: Testing materials with patients is an important step in the process of creating patient education materials. CMS states that "feedback from readers is the gold standard of evidence on how well your written material is working". Testing allows identifying areas of potential confusion which may lead to mistakes, complications and bad outcomes. Common methods for collecting feedback include individual interviews or focus groups, but these methods require a substantial cost and time investment for both staff and participants. This poster presents an efficient, streamlined and cost-effective way to get constructive patient feedback that leads to improvement of educational materials

Description: The team created online surveys based on the Patient Education Assessment Tool (PEMAT), a standardized national tool published by the Agency for Healthcare Research and Quality (AHRQ). This tool was developed to help clinicians determine the quality of patient education materials and has Yes/No questions to assess the clarity and intelligibility of the materials. The team adapted the PEMAT questions to address patients rather than clinicians and added an option for respondents to write comments on each of the questions. In addition, 3-4 comprehension questions to assess if the reader gained the knowledge they were intended to learn from the material were added to each survey. The survey along with a link to the material to be evaluated were sent via email to eAdvisors: patients who volunteered to advise the health system about their experiences virtually.

Conclusion: Collecting patient feedback via an online survey is highly beneficial. Participation is high because eAdvisors can do the surveys at their convenience, without having to travel. Staff appreciates not having to spend time on coordinating and managing individual interviews or focus groups and getting the feedback tallied in an Excel spreadsheet or a summary report. While the PEMAT scores obtained via the Yes/No questions were high, and responses to comprehension questions proved materials were understandable, responders' comments provided constructive, useful and implementable suggestions that led to significant improvements.

Poster Number: 115

Time: Sunday, May 5, 3:30 p.m.–4:25 p.m.

Personalizing Book Collection Development in a Small Clinical Library through Individualized Core Lists

Erin D. Nunley, Graduate Research Assistant, Preston Medical Library, Murfreesboro, TN; **J. Michael Lindsay, AHIP**, Head of Collections and Access Services, Preston Medical Library, University of Tennessee Graduate School of Medicine, Knoxville, TN; **Martha Earl, AHIP**, Director/Associate Professor, University of Tennessee–Knoxville; **Cameron Watson**, Library Associate III, Preston Medical Library, Knoxville, TN; **Rebecca Harrington, AHIP**, Assistant Professor, Research Services Librarian, University of Tennessee Graduate School of Medicine, Knoxville, TN

Background: Preston Medical Library staff sought to enhance the value and awareness of our electronic and print book collections by improving how they gathered input from clinical faculty. In seeking to facilitate faculty selection of core title selections, the library staff revised the format of title selection lists, providing more information on titles and ratings while streamlining the process for faculty to communicate their needs. The aims of our project were to elicit more faculty feedback in title choices through individualized communication, while providing comprehensive bibliographic information on formats and editions for staff use.

Description: Two types of collection development tools were created, one for use by librarians and staff, and a second type distributed to clinical faculty members. Relevant subject lists were downloaded from the Doody's Core Titles List and incorporated into the tools. Each book listed included information on the current availability of the work in the library's collections. Library staff were essential in updating the lists to show where the item was available, editions available, format, and year. Completed spreadsheets were distributed to liaison librarians. Subject lists in html format were transferred into Adobe Acrobat and redesigned as modifiable pdf forms. The relevant pdfs were distributed directly to each faculty member in a department. Faculty could check a box next to the titles they wanted added to the library's collection for research or teaching. The forms were returned to their library liaison.

Conclusion: Librarians gained access to a comprehensive list of core titles, with formats and editions in our collections noted. Once faculty returned their selection lists, the library director used these selections as justification for more funding to update and bridge gaps in the collection. Further, we created a feedback mechanism for collection development, increasing faculty awareness of library collections.

Poster Number: 116

Time: Monday, May 6, 3:30 p.m.–4:25 p.m.

Pilot: Elevating the Decision-Making of African-American Veteran Women during Pregnancy and Childbirth

Charlene Finley, PhD Student, University of North Carolina at Chapel Hll, Raleigh, NC

Objectives: Given the historical context of the integration of African-American women in the military and the Department of Veteran Affairs (VA) health care system, how does the availability (or lack thereof) of women's health services at the VA influence the health information needs and health information seeking behavior and decision-making during pregnancy and childbirth?

Methods: Potential candidates will be recruited by fliers posted in the Women's Health clinic at the Durham VA Health Care System in Durham, North Carolina. Eligibility of participation in the study will be based on the following criteria: 1) Identify as African-American woman, 2) between 18-44 years of age, 3) currently pregnant or was pregnant within the last 12 months, 4) and eligible for care within the VA health care system. In-person, semi-structured interviews will be used to explore the context of health information and decision-making for African-American women during pregnancy and childbirth. The questions will consist of a demographic survey and open-ended questions to capture the unique experiences and insights of veteran women. All study procedures were reviewed and approved by the University of North Carolina at Chapel Hill review board, and the Durham VA Office for Research.

Poster Number: 117

Time: Monday, May 6, 3:30 p.m.–4:25 p.m.

Plays Nice with Others: Blending Thematic Analysis with Elements of Systematic Review Methods to Identify Librarian Research Roles

Patricia F. Anderson, Emerging Technologies Informationist, University of Michigan-Ann Arbor, Ann Arbor, MI; **Emily C. Ginier**, Informationist, University of MI, Ann Arbor, MI

Objectives: In consideration of developing a systematic review (SR) service, we sought to identify librarian roles on SR teams. Guidelines and consensus works describe standard roles for SR librarians, however they lack unusual, innovative, or unique roles. To fill this gap, combining a systematic search with thematic analysis allows a more nuanced interrogation of the data, to discover the unknown unknown.

Methods: A systematic search of the literature identified 177 publications on librarian and informationist tasks and roles in systematic review teams. Title/abstract screening eliminated those off topic, and the remaining articles were retrieved in full. Thematic analysis (TA) of the fulltext articles followed the standard iterative analytic phases: familiarization with the data, creating initial data coding standards, coding, identifying themes, reviewing themes, and defining and naming themes. During analysis, additional articles were identified through citation mining. TA is typically bookended by two phases of dataset identification or creation at the beginning and report generation or writing at the end. It is common in healthcare research use of TA to combine it with a systematized search process. The search created the dataset of articles we used to identify both consensus areas and emerging opportunities regarding librarian engagement on systematic review teams.

Results: Beginning with 10 librarian roles identified in the first article analysed, by the time the first seven articles had been analysed, roles had expanded to 48 roles and seven clusters had begun to emerge. By May 2018, 69 roles and 8 clusters had been identified through analysis of 24 articles. At this point in the process, we sought external validation and confirmation of the structures through a collaborative process engaging other librarians in an exercise to confirm, clarify, and revise clusters and themes. This revision resulted in the current state of themes and clusters with 77 roles, 8 clusters, and 10 subclusters extracted from the analysis of 34 articles. This process illustrates the iterative nature of thematic analysis.

Conclusions: This process allowed us to confirm the central role of librarians in literature searching and document delivery for systematic review projects, while eliciting the novel roles of methodology experts, data management consultants, and engagement with publication and post-publication processes such as facilitating access, marketing, and promotion of findings for facilitating adoption and supporting translational processes. TA frees analysis from assumptions, allowing greater flexibility of analysis, both confirming existing constructs, and facilitating novel discoveries.

Poster Number: 118

Time: Sunday, May 5, 3:30 p.m.–4:25 p.m.

Polishing Medical Students' Presentation Skills: A Curriculum

Alexandra Gomes, AHIP, Associate Director, Himmelfarb Health Sciences Library, Vienna, VA; **Tom Harrod**, Librarian, Himmelfarb Health Sciences Library / George Washington University, Washington, DC; **Anne M. Linton, AHIP**, Director, The George Washington University, Washington, DC

Background: Presenting information to others is a large part of the medical profession, yet these skills are rarely taught in medical school. In response to a curriculum committee's statement of need, Himmelfarb librarians developed a curriculum that introduces specific presentation skills over eighteen months. Students have the opportunity to practice each set of skills and receive feedback. Each skill set builds on the prior set and all earlier skills are included as the presentations progress. The presentation curriculum is integrated with a required course in which students deliver formal presentations on topics of their choice, related to the overall block content.

Description: The overall curriculum was introduced to students near the beginning of their first year, and was imparted to students in small groups by their assigned librarian, in five modules. Topics ranged from including introductions and conclusions to using (and citing) professional level sources to incorporating interactivity in order to engage the audience. The librarian provided oral and/or written feedback to each student based on the criteria for that module. The final presentation in each semester was evaluated for a formal grade. A wrap-up session was held near the end of the 18 months to allow students to reflect on their own improvement, recognize the importance of the content, share their successes, and apply their presentation evaluation skills.

Conclusion: While some students began medical school with strong presentation skills, most others benefitted from the focus on specific presentation elements. The curriculum has been in place for 3.5 years, and clerkship directors remarked on the students' improved presentation skills once students reach rotations. Students also developed an appreciation for well-organized and delivered presentations when they encounter them at conferences or in other environments.

Poster Number: 119

Time: Monday, May 6, 3:30 p.m.–4:25 p.m.

Pop Up Library: Bringing Your Library to Your Patrons

Alyssa Grimshaw, Access Services/Clinical Librarian, Cushing/Whitney Medical Library, West Haven, CT

Background: The School of Nursing is located 8 miles away from our medical campus, where our library is located. There is a learning commons located in the School of Nursing with Course Reserve material, computers, and a space to study. The books located here do not circulate.

Description: Faculty and students mentioned that they would love to be able to check out books over break as a way to de-stress before the upcoming semester. In an effort to encourage reading during breaks, we decided to host a Pop-Up Library. Our student workers pulled over 180 books from the Biography, Historical, and Humanities in Medicine collections. The student workers were tasked with locating books that they thought were interesting and might want to read for fun. Access Services staff manned a desk to check out the books to patrons at the Learning Commons.

Conclusion: Lack of infrastructure for the pop up circulation desk led to creation of due date bookmarks. In the future, we would like to offer more leisure reading selection as we think our nursing students would like a break from medical texts while on vacation. The Pop Up Library was just a fun way of building relationships with the School of Nursing. We extended the event to two days due to interest. We hope to host future Pop Up Libraries.

Poster Number: 120

Time: Tuesday, May 7, 3:30 p.m.–4:25 p.m.

Poster Party: Building a Researcher Community in the Library

Kelli Hines, Scholarly Communications Librarian, Western University of Health Sciences, Harriet K. & Philip Pumerantz Library, Pomona, CA; **Rudy R. Barreras**, Marketing and Outreach Librarian, Harriet K. and Philip Pumerantz Library, Western University of Health Sciences, Pomona, CA

Background: Our health sciences university wants to increase its research output and profile. While the university values interprofessional collaboration, in practice, the colleges and departments are often siloed, with researchers unaware of research conducted elsewhere within the university. Furthermore, researchers often do not avail themselves of available support services. To combat these issues, we began hosting an annual "research mixer" in the library in 2014. We aimed to promote university research by providing a neutral location for researchers to discuss their research, mingle with potential collaborators from other disciplines, and learn about services available to assist them in their research.

Description: We structured the event as a poster session with light refreshments and tables staffed by librarians and representatives from other university departments that support research (Office of Research and Biotechnology, IRB, IT, etc.). This format allowed for a mix of formal presentation and informal conversation. The process of planning the research mixer took six months and included: setting the date; securing funding from the Office of Research and Biotechnology; advertising the event over email, social media, newsletter, and digital screens (including save the date, call for posters, and RSVP); setup (including posters, tables, and food); and takedown. We assessed the effectiveness of the research mixer by tracking attendance and the number of posters, sending a post-event survey, and seeing if our internal statistics showed an increase in use of research support services.

Conclusion: The research mixer increased awareness of research from all colleges and facilitated interdisciplinary collaboration, but it is inconclusive whether it increased awareness or use of library services. The event itself was very popular. Attendance more than tripled (from 23 attendees in 2014 to 72 in 2018) and is expected to continue to grow. The number of posters increased from 5 to 19 and represented students and faculty from all colleges. At least two different interdisciplinary research projects started because of conversations at the research mixer. Furthermore, the library benefited from a fruitful partnership with the Office of Research and Biotechnology.

Poster Number: 121

Time: Tuesday, May 7, 3:30 p.m.–4:25 p.m.

Where Do We Stand? Using Exit Surveys to Determine Impact and Use of Library Services

Annie M. Thompson, Director, USC Wilson Dental Library, Los Angeles, CA

Background: The purpose of this study is to determine the impact and use of library services by collecting data from exiting students. Three years (2016-2018) of data were collected from exiting Doctor of Dental Surgery (DDS), Dental Hygiene (DH), ASPID (Advanced Standing Program for International Dentists) and Advanced Specialty students in an effort to evaluate the quality of service, use of space and resources as well as opening hours.

Description: All students at the School are required to complete an exit survey prior to signing out of the library. In order to gain an overall view of their experience with the library, we ask: how important were our services to their success; how often they utilized other libraries on campus; ask for assessment on personnel, books, collections, and facility; how often they search for information for certain purposes; the importance of particular resources (books, journals, group study rooms); and how often they utilized our services. We also asked if hours were adequate and asked them to provide comments. The data have been used to demonstrate value of the library to higher administration and make adjustments to services offered, however, we have not presented a 3 year window and analyzed changes or trends.

Conclusion: Cumulative data from 2016-2018 show that students consider the library 'Important' to their success. The services and resources most important to the success of all classes are remote access to electronic resources, the dental school intranet, and the library website. The least important services and resources to their success are print materials and interlibrary loan. Services and resources most utilized are the space for studying, printing, and electronic collections. Comments were coded to discover that our students value the staff and library resources, but that they would like more space with more individual study. They also stated that hours are not long enough and they would like us to regulate reading room noise more frequently. The data show that there have been no large changes in responses over a period of three years. We have created a 'text a noise complaint' system, usage statistics show that there is no justification to lengthen hours, and a 3 year plan is being developed to offer a space more conducive to individual study with more group study options. Overall, the responses reflect that we continue to provide excellent service, but have some areas to improve.

Poster Number: 122

Time: Monday, May 6, 3:30 p.m.–4:25 p.m.

PRISMA-S: Developing a New Reporting Guideline Extension for Literature Searches

Melissa L. Rethlefsen, AHIP, Associate Dean and Fackler Director, University of Florida–Gainesville; **Jonathan Koffel**, Emerging Technology and Innovation Strategist, University of Minnesota Health Sciences Library, Minneapolis, MN; **Shona Kirtley**, Senior Research Information Specialist, University of Oxford, Oxford, None; **Siw Waffenschmidt**, , Cologne, Germany; **Ana Patricia Ayala, AHIP**, Research Services Librarian, University of Toronto, Toronto, ON, Canada

Objectives: Despite wide usage of the PRISMA Statement reporting guideline by systematic review authors, compliance with its items regarding literature search reporting is suboptimal. We sought to develop an international standard for literature search reporting aligned with the PRISMA Statement to improve the quality and reproducibility of reported literature searches.

Methods: We formed an executive committee to lead the PRISMA-S extension development. The study protocol was published prior to study inception. To identify potential items for inclusion, we performed a literature search. Identified items were reviewed for overlap and consolidated. We then used a three-step Delphi survey process to assess the items. The first survey asked 163 international experts to rate each item, and the second and third rounds asked respondents to select the 25 most necessary items for a checklist. Potential items moved to rounds 2 and 3 based on pre-specified criteria. Remaining items were discussed at an in-person consensus conference. After the consensus conference, the remaining items were consolidated into a checklist. Executive committee members developed an accompanying explanation and elaboration document. The checklist and documentation were distributed for pilot testing.

Results: We identified 405 potential items from 61 sources located through the literature search process. Sources included both explicit reporting guidelines and studies assessing reproducibility of search strategies. These were consolidated into 123 potential items for the Delphi survey. We received 52 responses (32% response rate) to the first survey, and 35 (67% response rate) to both surveys two and three. The results of the Delphi process were reported at the consensus conference meeting in May 2016. Post-consensus conference, 34 items remained. The checklist was finalized into 13 items and 10 sub-items. Pilot testing is underway.

Conclusions: The PRISMA-S extension for the PReferred Reporting Items for Systematic reviews and Meta-Analyses (PRISMA) reporting guideline will provide a consensus-driven international standard for literature search reporting. Using this new reporting guideline may enable librarians and information specialists to produce higher quality, more reproducible, transparent search strategies for systematic reviews and other literature review-based publications.

Poster Number: 123

Time: Tuesday, May 7, 3:30 p.m.–4:25 p.m.

Program Planning and Learning Outcomes of the First Systematic Review Advanced Searching Workshop in Taipei Medical University (TMU), Taiwan

Tzu-heng Chiu, University Librarian, Taipei Medical University, Taipei, Taipei, Taiwan (Republic of China); **Shu-Yuan Siao**, Head, Division of Knowledge Services, Taipei Medical University Library, Taipei, Taiwan (Republic of China); **Jui-Wen Chen**, Head, Division of Public Services, Taipei Medical University, Taipei Medical University Library, Taipei, Taipei, Taiwan (Republic of China)

Objectives: The clinical librarian team of TMU library has been participating weekly meetings of Cochrane Taiwan (CT) since Spring of 2018. As a result, TMUL and CT together hold a workshop of SR advanced searching on Nov. 2018. This poster reports implementation and participants' learning outcomes, hoping to serve as reference for other medical libraries.

Methods: To measure scales of the achievement of teaching objectives, authors administrated a two-stage questionnaire survey to collect data of learning outcomes. Printed questionnaires of pre-test and post-test were delivered and collected during the workshop. The questionnaire included six statements to exam attendants' degree of understanding of the skills taught by the workshop in a 5-poist scale, which are: Q1. formulating a PICO question, Q2. finding appropriate searching keywords, Q3. searching Pubmed and export results to Endnote, Q4. searching Cochrane Library and export results to EndNote, Q5. searching Embase and then export results to Endnote, and Q6. producing the PRISMA FlowChart. The number of participants are around 50. Authors utilized SPSS statistics software to analyze the collected data.

Results: The clinical question of this workshop is "Can fish oil prevent cardiovascular diseases among healthy adults?" 3 librarians from TMUL was responsible for teaching the search strategies and skills of Pubmed, Cochrane library, Embase and export search results using Endnote, and 2 lecturers invited by CT taught the PICO and flowchart drawing. Directors of TMUL and CT then moderated the discussion and feedback session. 31 valid questionnaires were collected (response ratio is 62%) and 3/4 respondents were female. Among them, 41.9% are nurses, 9.7% are physicians and 2.9% are pharmacist; 54.8% have bachelor's degrees, 29% have master degrees and 13% have a PhD. 32.3% have been attended EBM related workshop and 25.8% have been in SR workshops. As to self-evaluated scores of above 6 skills before and after this workshop: Q1 is 3.61to 4.45, Q2 is 3.71to4.39, Q3 is 3.48to4.45, Q4 is 2.94to4.19, Q5 is 2.87to4.26, Q6 is 2.19to3.45.

Conclusions: To sum up, the learning outcomes is quite good. Before taking the workshop, respondents thought their 6 skills are generally low (2.19-3.71), and after the workshop, all scores are higher (3.45-4.45). Skills of searching Cochrane Library, Embase, and producing the PRISMA FlowChart are much more improved. Due to the good result, the workshop will become a regular training program and open to attendants outside TMU campus from 2019.

Poster Number: 124

Time: Sunday, May 5, 3:30 p.m.–4:25 p.m.

PubMed as a Public Interface for MEDLINE: Assessing Whether PMC Has Changed PubMed's Composition

Peace Ossom Williamson, AHIP, Director of Research Data Services, The University of Texas at Arlington, Arlington, TX; **Christian Minter**, Community Engagement and Health Literacy Librarian, University of Nebraska Medical Center, Omaha, NE

Objectives: Questions have arisen about the quality of PubMed due to PubMed Central (PMC) as a growing component of PubMed. We tested claims that content in PMC is of low quality and affecting PubMed's reliability by evaluating the proportion of PubMed records indexed in MEDLINE over time and evaluating whether this aligns with the proportion of PMC records indexed in MEDLINE.

Methods: The authors retrieved the following via the National Center for Biotechnology Information (NCBI) interface: the percentage of MEDLINE-indexed records added to PubMed in 1990 and in the years between and including 2000 and 2017, and the percentage of MEDLINE-indexed records added to PMC between 2000 and 2017.

Results: Over 90% of PubMed records are indexed in MEDLINE; however, since the launch of PMC, the percentage of MEDLINE-indexed records added to PubMed each year has slowly decreased. This trend aligns with an increase in PMC content, which differs significantly from PubMed content in regards to MEDLINE composition. The largest impact comes from PMC full participation journals not indexed in MEDLINE, not from author manuscripts submitted to PMC in compliance with public access policies. Author manuscripts in PMC continue to be published in MEDLINE-indexed journals at a high rate (85%), but only comprise 2% of PubMed.

Conclusions: The differing scopes of PMC and MEDLINE will likely continue to affect their overlap, and further emphasis by the National Library of Medicine on PubMed's components will make PubMed users more clear about its scope. According to NLM, the aim is not to maintain a certain proportion of MEDLINE records in PubMed or PMC; the hope is that more MEDLINE-indexed journals will be deposited in PMC for long-term preservation and broader access. The authors also conclude that quality control exists in the maintenance and facilitation of both resources, but further research assessing article quality using critical appraisal methodology is needed.

Poster Number: 125

Time: Monday, May 6, 3:30 p.m.–4:25 p.m.

Putting the National Network of Libraries of Medicine (NNLM) on the Map: A Visualization Approach to Making Data-Driven Decisions for NNLM

Nicole Strayhorn, NLM Associate Fellow, American Dental Association Library & Archives, Chicago, IL

Objectives: The goal of this project was to transform an abundance of data from the National Network of Libraries of Medicine (NNLM) into insights through visualization to enable the NNLM to make data-driven decisions and enhance reporting to stakeholders.

Methods: Data related to outreach projects, exhibits, and their members was extracted from internal NNLM systems, the NNLM Network Members Directory, and the NLM Exhibit System. In addition, seven National Network Performance Measures & Evidence were implemented in November 2017 to demonstrate how the NNLM promotes access to biomedical information to improve the public's health. This data was compiled from the eight Regional Medical Libraries and five National Offices Year 2 Research Performance Progress Report (RPPR) then cleaned and normalized using MS Excel, Tableau Prep, and ArcGIS. Finally, the data was analyzed and visualized using Tableau Desktop by designing multiple dashboards.

Results: Approximately 60+ dashboards with maps, bar charts, bubble charts, and tree maps were created analyzing data related to membership, member engagement, partnerships, exhibits, outreach projects, and outreach activities.

Conclusions: By creating graphical representations of data, the NNLM has a fresh way of looking at their data and analyzing it for planning future projects, activities, exhibits, and members based on locations and special populations they want to reach. The National Evaluation Office (NEO) will continue building and expanding the visualizations and data analysis within the NNLM, creating a sustainable model for data-driven decisions.

Poster Number: 126

Time: Tuesday, May 7, 3:30 p.m.–4:25 p.m.

Quick, Cheap, Fun: Activities Designed to Gather Feedback and Engage with Patrons

Helenmary Sheridan, Data Services Librarian; **Melissa Ratajeski, AHIP**, Coordinator of Data Management Services; **Carrie L. Iwema, AHIP**, Coordinator of Basic Science Services; University of Pittsburgh, Pittsburgh, PA

Background: In order to tailor classes, services, and software offerings to patron needs, librarians at an academic health sciences library created several activities to capture user feedback in multiple contexts. Primary design objectives were that the activities be inexpensive to produce; quick for users to complete; and engaging, to pull users into a conversation. In addition, all activities were designed to be movable, so they could be brought to departments and events. Any questions that were asked were purposely not specific to any one discipline/area of research so they could easily be adapted and reused.

Description: To be portable and low-cost, activities used resources that were on-hand and easily transportable such as: dry-erase boards, paper, stickers, or glass containers. Two successful uses of created activities include: 1). During an annual research event where visitors would walk past tables without engaging, librarians drew them in with a hands-on activity around the question, "How can we help you with your data?" Users dropped glass "gems" into containers labeled with their answer ("Organization," etc.), whereupon the librarians followed up. 2). To gather feedback from students and researchers in the library, the question "What software do you need/want to learn?" was posted on two portable whiteboards placed in high-traffic areas in and directly outside the library. This format allowed patrons to reply at length or with a simple tally mark, and many responded to others' suggestions in a robust discussion.

Conclusion: These activities succeeded in being inexpensive to build, easy to complete, and engaging for patrons. They also produced actionable results; for example, data from the whiteboard activity supported the decision to add advanced EndNote classes and caused librarians to realize that increasing advertisement may be needed for already-available software. Because designing and implementing these activities cost little time or money, making changes is relatively easy. Activities are created and tested in an iterative design process, incorporating evaluation into the development cycle as librarians modify each setup after observing its performance.

Poster Number: 127

Time: Sunday, May 5, 3:30 p.m.–4:25 p.m.

Ranking and Visualizing Articles to Facilitate Article Screening in Systematic Reviews

Michael Eliot Bales, Research Impact and Evaluation Informationist; **Peter Robert Oxley**, Associate Director of Research Services; **Terrie R. Wheeler**, Director; Weill Cornell Medicine, New York, NY

Objectives: Systematic reviews summarize evidence and help improve the quality and safety of health care. To ensure that evidence is understood and used in a timely fashion, more efficient methods are needed to support time-consuming aspects of systematic review development. Here we describe a method designed to accelerate article screening during the selection phase of systematic review development.

Methods: We will compile sets of articles retrieved in response to queries developed by an expert systematic review librarian for three systematic reviews. Each article will be assigned a code with respect to its eventual inclusion or exclusion in the review. For each systematic review, we will then employ three methods for projecting the articles into a multidimensional space (latent Dirichlet allocation, doc2vec/FastText, and GloVe), such that articles are arranged based on similarity with respect to their underlying semantic features. We will also arrange the articles in rank order based on similarity to the articles that were eventually selected for inclusion, and will use this ordering to determine the most effective article ranking approach.

Results: After selecting the most effective article ranking approach, we will use various projection techniques to produce visual representations of articles, wherein similar articles will appear spatially proximal to one another. The interface will allow users to move the image (pan), zoom, click for details, and select or reject articles. These visual representations of ranked articles will assist in the development of search queries, aid expert searchers in describing the relationships between search queries and the results returned, and color-code articles based on predicted likelihood of inclusion in the final screening.

Conclusions: We expect that this article ranking and visualization approach will significantly reduce the time required to build systematic review protocols and to screen references, thereby increasing the speed and comprehensiveness of systematic reviews.

Poster Number: 128

Time: Monday, May 6, 3:30 p.m.–4:25 p.m.

Redesign of a Mobile Applications Lecture Using Gamification and Pop Culture

Emily P. Jones, AHIP, Research and Education Informationist, Medical University of South Carolina, Charleston, SC

Background: A mobile applications lecture was redesigned in Fall 2019 as part of a required, 1-credit hour Introduction to Drug Information class for first year pharmacy students. Previously, the class was taught in traditional lecture style with active learning incorporated via TopHat, an audience response system. For the Fall 2019 class, the librarian chose to model a popular culture trivia game, HQ Trivia, for the in-person class titled “Mobile Apps Trivia”.

Description: Mobile app instruction occurred via a flipped classroom using recorded videos that students watched one week prior to an in-person class. Class time was devoted to application of content learned in the previous week. Students were asked drug information questions over 3 rounds, with questions becoming increasingly difficult each round. Students were given a range of 0-2 minutes to look up answers to questions using drug information mobile apps. Then, students were prompted to provide an answer within an assigned time frame of 10-15 seconds. The librarian used an institutional subscription to Poll Everywhere, an audience response system, to create the game using the competition mode. Respondents were ranked on a point system based on correct responses and amount of time used to answer questions. A leaderboard displayed after each question closed indicating the students’ rank in the competition.

Conclusion: Overall, the response from the students was very positive. Students enjoyed the gamification of the class and felt that the competition mode added an extra layer of competitiveness and engagement. Students responded positively to the extra incentive of prizes which were awarded for both participation and performance during trivia. Poll Everywhere has released updates to competition mode since the lecture that allow for more customization. The course coordinator for the course has expressed interest in working with the librarian to potentially develop a research study on the effects of competition mode on student engagement and satisfaction.

Poster Number: 129

Time: Tuesday, May 7, 3:30 p.m.–4:25 p.m.

rELEVATE: A Model for Expanding Resident Outreach and Services

Tiffany A. Follin, AHIP, Medical Liaison and Outreach Librarian, Medical and Health Sciences Collections & User Services, Deerfield Bch, FL; **Michael DeDonno**, Assistant Professor, Florida Atlantic University, Boca Raton, FL

Background: We are a small two-person department integrated into an academic college, comprised of approximately 2,429 individuals. The Liaison and Outreach Librarian position has undergone many evolutions in the past; the most recent of which has been to focus on residents as an outreach population. The goal is to create a model increasing physical access to a librarian, advocate library resources, and streamline information available to residents to facilitate self-directed learning.

Description: Incoming residents receive a concise library session as part of their first year orientation. Last year for the first time, Internal Medicine residents also received an in-depth library resources and services session. Anticipating an increased need for services, five goals were created in collaboration with a colleague in Educational Leadership & Research Methodology: 1) create a Medical Literature Review Request Form to give residents structure and provide faculty with organized distribution when approached, 2) craft materials and guides of library research strategies, 3) provide videos on YouTube, allowing hospital-based residents to circumnavigate firewalls, 4) prepare small “pearl” sessions for select didactic days, and 5) Establish an on-site presence at the hospital and in the CoM Hub for increased access. Program evaluation will include desk tracker statistics, faculty feedback, and a program survey.

Conclusion: We expect to measure residents’ satisfaction with this new model for library outreach and services, and improved skill level and self-efficacy with using library resources for scholarship, research, and clinical applications. It is anticipated there will be a difference in services usage based on residents’ individual areas of research or clinical interest; however, we are hopeful that tailoring program content will result in an overall, high level of satisfaction, skill development, and confidence in using library resources and services.

Poster Number: 130

Time: Sunday, May 5, 3:30 p.m.–4:25 p.m.

Research Data Management Librarian Academy

Elaine Russo Martin, FMLA, Director of Library Services, Harvard Medical School, Boston, MA; **Jean P. Shipman, AHIP, FMLA**, VP, Global Library Relations, Elsevier, Cottonwood Heights, UT

Background: Many librarians have become active participants in their institutions' research lifecycles. Librarians have the expertise to capture scientific knowledge as it is being created so it is managed and recorded for later dissemination, but their skills with managing research data vary. For librarians to effectively lead the application of research data management (RDM) solutions, training may be needed.

o Aim

• Several librarians studied the need for an online Research Data Management (RDM) Librarian Academy. The team includes Harvard Medical School, Tufts Health Sciences, MCPHS University, Boston University School of Medicine, Northeastern University, Elsevier, and Simmons University.

Description: The RDM Librarian Academy project team compiled an inventory of existing courses for academic librarians and conducted a needs assessment through interviews, surveys and focus groups to identify gaps in current training offerings and to identify what librarians and researchers need to contribute to their success. Library school involvement was critical to the project, and the team also interviewed library and i-school educators as well. Simmons Online will be partnering with the project team to develop the online training modules. Credit will be granted for those desiring such. The modules will be available to any one across the globe as they will be posted on a website.

Conclusion: The needs assessment indicated librarians feel they want to be part of research teams to assist with managing research data, but they often feel they don't have the needed skills or confidence to do so. They prefer to learn through online modules and at their own pace. The training program under development addresses these expressed needs. The training inventory guided the development of the 6-7 modules to address gaps in current offerings available to practicing librarians. This poster will review components of the online training and certification program, as well as share the key results of the needs assessment.

Poster Number: 131

Time: Monday, May 6, 3:30 p.m.–4:25 p.m.

Research Development Webinar Series: A Collaboration amongst Touro College and University System Libraries

Joanne M. Muellenbach, AHIP, Director, Touro University Nevada, Henderson, NV; **Sara Tabaei**, Library information Literacy Director, Touro College & University System, New York, NY; **Jason Fetty**, Reference and Instruction Librarian, Touro University Nevada, Henderson, NV; **Deborah A. Crooke**, Associate Director, New York Medical College, Health Sciences Library, Valhalla, NY; **Rhonda L. Altonen**, Director, Touro College of Osteopathic Medicine, New York, NY; **Amy Mikala Castro**, Access Services & Instruction Librarian, Touro University California, Martinez, CA; **Julie Breanna Horwath**

Background: This collaboration amongst Touro College and University System (TCUS) libraries began as an initiative of the College Research Council to increase TCUS's research footprint. Specifically, faculty and students needed to develop greater research knowledge and skills. The Library Advisory Committee, one of four subcommittees of the Research Council, recognized the wealth of research taught across the system by individual libraries, and saw this as an opportunity for collaboration. Program objectives include:

* develop faculty and student research skills

* collaborate with librarians to offer a variety of research topics

* increase the value and visibility of TCUS libraries

Description: The Library Information Literacy Director created a research guide, and TCUS librarians promoted the research webinars to their faculty and students. Group viewing rooms were scheduled, in addition to the option to participate in Zoom conferences from any location. The response was overwhelmingly favorable. The initial, spring 2018 series consisted of seven webinar topics between two TCUS locations, in New York and Nevada. It was then decided to invite TCUS libraries in California and at other New York locations to participate and to expand the range of research topics. Thus, in fall 2018, the research development series included 18 webinars, contributed by five TCUS libraries. Topics included: altmetrics; data management; open educational resources; ORCID; and systematic reviews. Webinar times were adjusted for the various time zones, and sessions were recorded and linked from the workshop guide, whenever possible.

Conclusion: The TCUS libraries collaboration has been a huge success. It has opened up new venues for librarians to work with faculty and to teach new scholarly communications topics. The webinars have also prompted future collaborations, such as embedded librarian projects. Going forward, we hope to offer recorded sessions, adapt session topics for course-integrated instruction, and offer research snippets at selected committee and departmental meetings. In addition, we would like to conduct pre-test and post-test assessments in order to determine the impact that this research webinar series has on faculty and student participants, and their library user satisfaction, overall.

Poster Number: 132

Time: Tuesday, May 7, 3:30 p.m.–4:25 p.m.

Restructuring a Literature Search Service: Preliminary Data and Feedback

Lilian Hoffecker, Research Librarian, Univ. of Colorado Anschutz Medical Campus, Aurora, CO; **Kristen DeSanto, AHIP**, Clinical Librarian, University of Colorado Anschutz Medical Campus, Aurora, CO; **Ben Harnke**, University of Colorado–Aurora; **Christi Piper**, Reference Librarian, University of Colorado Anschutz Medical Campus, Aurora, CO; **Nina L. McHale**, Head, Education & Reference, University of Colorado Strauss Health Sciences Library, Aurora, CO

Background: Literature searches at the authors' library were conducted by one or two librarians until recently, each of whom approached their searches individually. They conducted their searches with little consistency in their approaches or systematic record-keeping. But as requests for searches increased, we made the decision to transform the service so that multiple searchers can participate while producing consistent results. At the 2018 MLA conference, the authors reported on building a literature search service at an academic health sciences library. Here, we describe how it has progressed and what we've learned about the service as we expand data collection.

Description: An online database was developed with the help of an IT professional for patrons to make requests and for searchers to enter detailed data for each search request. For example, the database collects information on the amount of time spent for each project, nature of the search topic, details of the collaborators involved, co-authorship for the librarian, the status of reports and publications, and payment information for paid searches. The database went live in mid-October 2018 and we can now report with specificity on the search service.

Conclusion: Since October 2018 and as of early January 2019, the database shows a total of 42 searches, 22 that are in progress, 19 completed, and 1 suspended (searches for which we have not received any recent communication). Analyzing the data of the in-progress searches, librarians have devoted roughly 311 hours or 39 work days. Of the total search requests, 14 involved the librarian as a co-author. These data are valuable for adapting our literature search service to meet needs of our users and for making appropriate decisions about library service priorities.

Poster Number: 133

Time: Sunday, May 5, 3:30 p.m.–4:25 p.m.

Riding the "L" to Open Educational Resources (OER) Initiatives: New Destinations for Academic Librarians

Virginia F. Desouky, AHIP, Scholarly Engagement Librarian, West Virginia University Health Sciences Library, Morgantown, WV

Background: According to a recent Babson Survey Research Group study, recognition of the use of OERs (Open Educational Resources) for instruction is growing among faculty and department heads. This rise in interest in open educational resources has meant new roles for librarians in academic libraries. Among these are those of advocate, advisor, author, materials reviewer, and publisher. This poster will describe these new roles and will show how librarians can successfully collaborate with campus leaders to meet the challenges of implementing a campus-wide Open Education Initiative. It will also examine what skills or technical competencies are needed in these new roles.

Description: This poster will examine new roles of academic librarians participating in OER initiatives and will discuss challenges of these roles. Examples of success stories will be given as well as suggested steps for implementing a campus-wide initiative. Academic librarians involved in campus OER initiatives throughout the U.S. will be surveyed about their experiences with OERs in general and OER initiatives in particular, if and how their positions have changed, what challenges they face with implementing an OER program at their institutions, and their strategies for coping with the challenges. By the end of this session, participants will be able to describe the effect that OER initiatives have had on academic librarians' positions and will be familiar with the process of starting a campus-wide OER initiative.

Conclusion: Measurable outcomes of the survey will be changes in job descriptions of academic librarians working with OER initiatives and additional skills or competencies needed.

Poster Number: 134

Time: Monday, May 6, 3:30 p.m.–4:25 p.m.

Rightsizing with Real-Time Data=More Bang for Your Buck

Lisa M. Carter, Director of Library Services, Hartford Healthcare Library Services, Hartford, CT

Background: With so many changes happening at Hartford Healthcare, including partnerships and the streamlining of services it became apparent that we were wasting money by providing resources for patrons who didn't need them. What we needed was a business model of resource acquisitions based on need rather than all for one and one for all.

A major deterrent in changing to a needs based solution was that all departments and all locations shared the same IP addresses. Furthermore, we didn't have reliable usage statistics on which to base our renewal decisions and contract negotiations.

Description: We realized that to change to a needs based solution that it was necessary to know about changes before they happened. Newsletters and PR announcements provide only OLD news prohibiting us from being proactive which is necessary to base our budget on actual needs. It was necessary to access people in the know. We realized that we had to start being nosey. So we joined committees, meetings, and huddles - we became regular participants to get the scoop and perceive the need.

In addition to obtaining information about changes within the institution, we needed a consistent and reliable method to measure usage. We needed to know which user groups and locations were using resources and the frequency of access. This information was essential in order to rightsize our resources, negotiate renewals and new purchases.

Conclusion: Obtaining the knowledge needed to proactively respond to changes within the institution allows us to not only purchase resources based on need it also provides us with other valuable benefits. It promotes the library - we now have advocates and power users that help market specific resources and provide valuable feedback. We get actual fan mail.

Obtaining early knowledge of corporate plans and having access to reliable usage stats has enabled the Hartford Healthcare medical libraries to save hundreds of thousands of dollars in resource subscription costs.

Poster Number: 135

Time: Tuesday, May 7, 3:30 p.m.–4:25 p.m.

Rising above the Competition: The Group Interview Experience

Christy Jarvis, AHIP, Associate Director, University of Utah., Salt Lake City, UT; **Tisha Mentnech**, Research Librarian for Life Sciences & Research Metrics, North Carolina State University Libraries, Salt Lake City, UT; **Nena Schvaneveldt, AHIP**, Education Librarian, University of Utah–Salt Lake City; **Shawn Steidinger, AHIP**, Assistant Librarian for Clinical Services, University of Utah–Salt Lake City

Background: Faced with two vacant liaison librarian positions to fill and concerned about overburdening existing library staff, an academic health sciences library experimented with an interview approach seldom used in academia. Instead of scheduling individual interviews on separate days, the search committee arranged onsite group interview opportunities that hosted multiple candidates simultaneously. The committee's objectives were to: (1) Shorten the timeline between posting the open positions and extending final job offers; (2) Avoid staff burnout during the search process; (3) Ensure each candidate received sufficient undivided time and attention to allow for a thorough evaluation.

Description: After initial screening interviews, the search committee invited six candidates onsite for a full day of activities, including a presentation, interviews with the library director and the search committee and various interactions with library faculty and staff. The Chair designed a schedule that hosted three candidates on two separate days and set up a rotation of activities throughout each day. Some activities occurred with the candidates together in a group, such as a meet and greet with staff. Other time slots allowed candidates to engage in solo activities such as giving their presentation and meeting with library faculty. The time lapse from job posting to hire decision was compared to previous faculty hires to determine if this condensed interview schedule resulted in faster hires. Surveys distributed to job candidates and existing library staff revealed attitudes about participating in the process.

Conclusion: Search committee activity – from the time of job postings to the time of hire decisions – was shortened in comparison to similar faculty hires in the previous five years. Candidate feedback was overwhelmingly positive and indicated that each candidate felt there was sufficient time and attention devoted to each individual. Feedback from library staff revealed a high level of satisfaction with the group interview process. The Library will consider employing a group interview approach for future hiring opportunities.

Poster Number: 136

Time: Sunday, May 5, 3:30 p.m.–4:25 p.m.

Sisters of Charity of Leavenworth (SCL) Health "Nursing Research Fellowship" Program Three-Year Analysis

Karen K. Wells, System Director, SCL Health Library System, Sisters of Charity of Leavenworth Health Care System, Denver, CO; **Harriette Kelly**; **SANDRA VANNICE**, Evidence-Based Practice Specialist, SCL Saint Joseph Hospital, LAFAYETTE, CO

Background: We report on the progress of our Nursing Research Fellowship (NRF) program at SCL Health from 2016- Present. As participant instructors, our objective is to provision nurses with literature review skills. Searching proficiencies enable nurses to gather evidence based practices and guidelines via the medical literature, and perform systematic reviews, research syntheses, and literature appraisals towards those ends. Our ultimate goal is to sanction nurses to impact bedside patient care protocols and procedures and to develop continual traditions of information discovery through the pursuit and examination of the literature.

Description: The NRF Program began in 2016 with multiple subject matter experts teaching nurses, in 10 sequential modules, a systematic approach to nursing research. Librarians are an essential part of the program, providing literature review training to the Fellows. This poster describes the program's essential mentors, quantitative data on participants, list of completed research projects, and challenges and changes implemented to address those challenges, from 2016-present.

Conclusion: The assemblage of evaluative quantitative data, as presented from the NRF Director, on number of program participants, research topics, post-program individual surveys, oral or written comments, and reports on the dissemination of Fellows' research findings, are communicated in this poster.

Program evaluation reveals success, as evidenced by its evolution into a multi-site program, with increased number of participants, and an increase in the formal sharing of research findings both locally and nationally. Successful research queries are noted, as are changes to enhance learning. Further, some Fellows have garnered Nursing awards as a result of their participation in the program.

Poster Number: 137

Time: Monday, May 6, 3:30 p.m.–4:25 p.m.

Separating the Wheat from the Chaff: A Semi-Automated Method for Identifying Publications by Faculty of an Institution Using PubMed, Scopus, RefWorks, and Python

Susan Keller, Librarian, Children's National Medical Center, College Park, MD; **Deborah Y. Shroder**, PhD Candidate, Department of Physiology, University of Pennsylvania

Background: Objective: Produce list of publications by institution's faculty for purposes of internal tracking of publication history in a reproducible, minimally-intensive manner. 1) Identify all publications from an institution within a time frame, excluding e-publications ahead of print, and identify all authors (no "et al"). Ambiguous institution names used in citations can make this an iterative process. 2) Highlight all faculty authors within the list, then produce sub-lists of articles by each faculty member and each department. Additional possible subsets of the list include a list of faculty publications in "high impact" journals. Authors using multiple names can complicate this process.

Description: 1) Find all of publications written by faculty authors affiliated with Children's National Medical Center. This step is complicated by the lack of a standard way of identifying the institution. Synonyms for Children's National in Scopus include are varied and include Children's National Medical Center, Childrens National Health System, and many others.

2) Identify the faculty authors on the list of publications. Some sources of ambiguity include variations in the spelling, marriage associated name changes, inconsistent hyphenation, and inconsistencies between the name the author uses to publish and the name on the reference faculty list.

3) Only the creation of a custom Python program made the creation of the highlighted faculty author list possible.

4) Faculty review the draft list of publications and make corrections and additions before the list is published on the hospital website.

Conclusion: Since 2016, the use of the custom Python program has been successful in the creation of a highlighted list of faculty author publications. The program also creates a draft of the publication list that identifies publications without a faculty member and highlights other errors as well. The program was rewritten in 2018 and can now match faculty authors with high impact publications. Python is an open-source program and the creator will make the code available upon request.

Outcome measures include use of the author list to demonstrate faculty productivity and generation of good will toward the library.

Poster Number: 138

Time: Tuesday, May 7, 3:30 p.m.–4:25 p.m.

Service and Staffing Practices in Academic Libraries Serving College of Osteopathic Medicine Programs: A Mixed Methods Study

Joanne M. Muellenbach, AHIP, Director, Touro University Nevada, Henderson, NV; **Wendy C. Duncan**, Provost, California Health Sciences University, Clovis, CA; **Lisa A. Ennis**, Director of Library & Learning Resources, Alabama College of Osteopathic Medicine, Dothan, AL; **Anna Yang, AHIP**, Health Sciences Librarian, California Health Sciences University, Clovis, CA

Objectives: To conduct a systematic assessment of service and staffing practices within academic libraries serving college of osteopathic medicine (COM) programs. To detect trends in new services being offered through comparisons across COM schools, justify the need for additional services and staffing, and assist leadership within developing medical schools in planning for their future academic health sciences libraries.

Methods: A mixed methods study was conducted in three phases. The first phase involved the identification of COM programs within the United States, and the contact information for the library directors or leaders serving them, via their publicly-facing websites. Phase two consisted of a review of the literature to identify current practices regarding services and staffing, and the design of a survey directed at the library directors or leaders of academic libraries serving COM programs. Following an initial review of the Phase Two survey results, additional questions were developed and answers were provided by a selected group of Phase Two library leaders through in-depth phone interviews, which comprised the third phase of the study.

Results: There were a total of 35 COM libraries identified, with at least four COM libraries having one or more branch locations. While many of the COM library websites provided useful information, some were lacking in detail. In Phase Two of the study, 30 out of a possible 35 completed surveys were returned, for an 86% return rate. Phase Three phone interviews with selected COM library leaders provided more in-depth information related to services that have increased the library's visibility, motivation for staff to offer new services, current staffing levels, and competencies needed for serving in these new roles.

Conclusions: This study provides a systematic comparison of services and staffing within academic libraries serving COMs. Library leaders within new medical schools and those within long-standing, traditional schools, can use this data for budget, and strategic, planning. In addition, medical school administrators may find opportunities in the results for more productive collaborations with the library, in areas such as curriculum-integrated instruction and scholarly communication. Finally, leaders within developing medical schools will become better informed about current library services and staffing, in support of accreditation, and in order to plan for an appropriate budget for their future health sciences libraries.

Poster Number: 139

Time: Sunday, May 5, 3:30 p.m.–4:25 p.m.

Simplifying the Process for Finding Research Funding: A Cross-Campus Collaboration at a Large Academic Institution

Merle Rosenzweig, Informationist, University of Michigan–Ann Arbor; **Judith Smith**, Informationist, tbd, Ann Arbor, MI; **Amy Puffenberger**, Marketing & Communications Manager, University of Michigan–Ann Arbor; **Ann Curtis**, Marketing Director, University of Michigan Medical School, MI

Background: The objective was to create an interdisciplinary cross-unit partnership to develop a comprehensive online portal and consultation service to facilitate funding opportunity success for researchers.

To better understand and meet the needs of our researchers, multiple units within the library worked with campus stakeholders to develop an online survey that was distributed to researchers across our campus that were identified as individuals interested in health sciences research. The emergent themes in the survey highlighted the time needed to search for funding, the difficulty in finding relevant opportunities, and the ability to find precise funding applicable to their particular research project.

Description: In light of the key challenges identified through the survey, the primary goals of the collaborative project were to create a research funding portal and consultation service that could serve all faculty on campus in harvesting resources within our university and beyond, regardless of discipline, to assist in their funding search. The foremost design priority was to make the guide both visually inviting and easy to navigate. The design incorporates intelligent redundancy throughout so that key resources are included on each page of the guide. The guide provides access to a Research Funding Search Consultation form for researches to request a personalized grant-seeking consultation. Since the time that the Guide and consultation service were officially announced there has already been high usage of both the Guide and consultation service.

Conclusion: Researchers have the demanding task of finding funding to support their work, and gaining a better understanding of their needs can provide insight into what services and resources are needed. Informationists and librarians can play a key role in this challenging process. The collaborative effort to design and produce this guide and consultation service can serve as a model for others seeking to partner with stakeholders. Establishing and proactively maintaining a collaborative relationship with units across the research enterprise can build a sustainable system to help researchers navigate the complex funding process.

Poster Number: 140

Time: Monday, May 6, 3:30 p.m.–4:25 p.m.

Small but Mighty: The Role of Letters-to-the-Editor during the Zika Virus Epidemic in the Americas

Frances A. Delwiche, Library Associate Professor/Research & Education Librarian, Dana Medical Library, University of Vermont, Burlington, VT

Objectives: Formally published Letters-to-the-Editor are an important component of scientific communication, and during a rapidly evolving epidemic, can be especially useful to the timely dissemination of information. This study sought to demonstrate the value of this often disregarded publication type through a bibliometric analysis of letters written in response to the 2015-2016 Zika Virus (ZIKV) epidemic in the Americas.

Methods: A PubMed search was conducted on the terms (Zika OR ZIKV), and limited to the years 1952 through 2018 and Publication Type = Letter. After eliminating irrelevant results, the results were exported into an EndNote library. The full-text (PDF) of each letter was manually examined, and each item assigned to one of five categories: Response, Author Reply, Observation, Case Report, or Research. Several incorrectly indexed items were excluded from the dataset, and a number of letters discovered serendipitously were added. The number of authors of each letter was documented, as was the number of references cited, the presence or absence of tables, charts, or other graphics, and whether the work was supported by external funding. The data was then exported into an EXCEL spreadsheet, and analyzed.

Results: Of 486 letters in the study, 244 (50.2%) were published in 2016, dropping to 145 (29.8%) in 2017, and 82 (16.9%) in 2018. Contrary to conventional wisdom, only 30.5 % of letters were reader responses to a previously published article, and 10.7% were author replies. The remaining letters were categorized as observations (23.0%), case reports (14.4%), and research (21.4%). The letters were written by 1-35 authors, and included 0-62 references. Nearly 15% reported external funding, and 38% contained graphics. An interesting anomaly were the 103 letters authored or co-authored by one particular individual (VW), which constituted 21.2% of the total.

Conclusions: Formally published Letters to the Editor have traditionally been thought of as a tool for brief, asynchronous, conversations between readers and authors. In addition, during an emerging public health crisis such as the recent Zika Virus epidemic, they can play an important role in the dissemination of vital information. Owing to their relatively short turn-around time, they serve as a vehicle through which early clinical and research findings can be rapidly pushed out to the scientific community. However, as this study demonstrates, the very characteristics that describe this unique publication type may make it simultaneously subject to various publication anomalies.

Poster Number: 141

Time: Tuesday, May 7, 3:30 p.m.–4:25 p.m.

Stand Up for Health: Health and Wellness Services for Your Community

Margot G. Malachowski, AHIP, Education & Outreach Coordinator, National Network of Libraries of Medicine, New England Region, Worcester, MA; **Bobbi Newman**, Community Outreach & Engagement Specialist, National Network of Libraries of Medicine - GMR, Iowa City, IA; **Carolyn Martin, AHIP**, Consumer Health Coordinator, National Network of Libraries of Medicine, Pacific Northwest Region, Seattle, WA

Background: Research shows that consumers turn to public libraries when seeking health information. Public library staff report apprehension when asked health questions. Public librarians explicitly express interest in: learning about reliable sources of health information; matching patron needs with appropriate resources; ethical issues related to health information reference; teaching patrons how to conduct searches; and privacy and confidentiality during reference interviews. The National Network of Libraries of Medicine (NNLM) provides free educational opportunities to public library staff through webinars, in-person instruction and asynchronous courses. Earning library-related continuing education credits is required in some states. Research supports the need for increased staff knowledge and confidence in handling health-related questions.

Description: The National Network of Libraries of Medicine Greater Midwest Region (NNLM GMR) received funding to support the evaluation and development of an asynchronous consumer health information course. Requirements of this project: incorporate recommendations from NNLM instructors, National Library of Medicine staff and public library staff; pilot the revised course with a nationwide cohort; incorporate feedback from the pilot; and deliver a second instance of the revised course. The revised course must meet existing requirements for public library certification and for Level 1 certification of Medical Library Association's Consumer Health Information Specialization. NNLM GMR partnered with the Public Library Association (PLA) and OCLC's WebJunction to evaluate the course to meet the needs of public library staff. With input from WebJunction, NNLM staff streamlined the course before enrolling 64 library staff into the first cohort. PLA and WebJunction assisted with the call for participation in the second cohort of 75 library staff. NNLM revised the course after each cohort, using evaluation data, instructional design principles and learner-centered approaches.

Conclusion: The University of Iowa's Center for Evaluation and Assessment provided evaluation of the project. Course participants gave overwhelmingly positive feedback. Participants reported sharing resources with directors and colleagues, incorporating newly acquired knowledge at the reference desk, and utilizing new collection development strategies. Both cohorts experienced a 47% attrition rate. Library staff reported lack of time as the most common reason for discontinuing. Participants received a follow-up questionnaire 3 months after completing the course. Fifty percent reported developing new programming or outreach. Almost 100% reported continued use of a resource taught in the course. Course meets public library certification requirements and requirements for Level 1 certification of Medical Library Association's Consumer Health Information Specialization.

Poster Number: 142

Time: Sunday, May 5, 3:30 p.m.–4:25 p.m.

Strengths, Weakness, and Opportunities: An Assessment of Kaiser Permanente Libraries' Core E-Book and Print Titles

Loree Hyde, Regional Manager, kpLibraries NW, Kaiser Permanente NW, Clackamas, OR; **Sara Pimental, AHIP**, Senior Consultant, Kaiser Permanente, Oakland, CA; **Eve Melton, AHIP**, Regional Director Library Services NCAL, Kaiser Permanente, Stockton, CA; **Tori Jennell Ward**, Associate Project Department Coordinator, Kaiser Permanente, Portland, OR

Background: The purpose of our project was to assess and enhance Kaiser Permanente's (kpLibraries') national e-book collection and further develop related processes of collection development. The project supports kpLibraries' national Resource management policy that requires maintenance of a "current, authoritative collection of books, multimedia, and electronic materials in the areas of health sciences, clinical, educational, professional development, patient care services, and consumer health information…". Our aim was to gain an understanding of what percentage of our collection can be considered "core", as well highlighting areas of strength and gaps in the collections.

Description: Our dataset (17,694 records) included e-book usage stats from e-book packages, e-book titles purchased individual and print circulation data from our ILS. E-book usage data spanned an 18-month period including 2016 through the second quarter of 2018. Print circulation data started in 2013. To provide scope, we compared our titles owned to the external collection development resource Doody's Core Titles 2017 (DCT). This comparison allowed us to determine the following as related to the DCT: titles not owned in any format; titles not owned in the recommended edition; older editions to be replaced as e-books; high use print titles that could be added as e-books. In addition, we were able to analyze which non-DCT e-book titles had high or low usage and identify strengths in the collection.

Conclusion: We own 69.09% of the titles listed in DCT. Of that, 69.23% are e-book titles. We also found that 53% of our print collection had not circulated, with 3% of the collection circulating 16+ times. Within our e-book collection, 51% of e-books were used between 1 and 100 times, while 5% of the collection was used 1300+ times. We now have useful data and title lists for marketing and building our collections. Our next steps include making informed weeding decisions, as well as identifying high-use subject areas and Doody Core Titles not owned as possible e-book collection development opportunities.

Poster Number: 143

Time: Monday, May 6, 3:30 p.m.–4:25 p.m.

Supporting Evidence-Based Practice in a Competency-Based Nursing Program

Samantha Mosby, Digital Services Librarian, Western Governors University, Salt Lake City , UT; **Casey Swenson**, Digital Services Librarian, Western Governors University, Salt Lake City, UT

Background: Competency-based nursing education is a growing approach within the landscape of health education. Our university provides competency-based education in an online-only environment and with this comes unique challenges and opportunities. An important and often demanding element of nursing education is teaching evidence-based practice. Six of the nursing degrees at our school share a required evidence-based practice course and necessitate robust support from the library. In response to the needs of students, the library developed and implemented multiple avenues for students to receive librarian assistance. By providing the expanded services the library helps students meet the competencies of the course.

Description: In 2018 there were 13,521 students enrolled in the evidence-based practice nursing course. The assessment for this course includes two research tasks, which are best completed using library resources. We have implemented multiple options for students seeking assistance in the course—and the nursing programs in general—including a 24/7 chat service, phone reference appointments, live webinars, email reference, and detailed library guides. The 24/7 chat service is evaluated quarterly using a rubric to determine quality. During phone reference appointments students receive individual guidance on formulating their search strategy and using the databases. Email reference support requests are answered within 24 business hours. Webinars are hosted twice a week and focus on course specific requirements surrounding different types of evidence-based research. Library guides include information from both the students' course textbooks, and general information on using search limiters to locate needed resources. Combined these products and services provide students with a variety of options for meeting their information needs.

Conclusion: When looking at library resource usage for the evidence-based practice course, 73 of 399 or 18.29% of email reference requests, 1,213 of 9,629 or 12.6% number of chat sessions, and 187 out of 475 or 39% of phone appointments were related to this course. By providing multiple options for reference support for students working on their evidence-based practice research performance assessments, and by aligning the delivery of that reference support with the university's model of competency-based education, the WGU Library has seen steady growth in the usage of the library's provided reference services.

Poster Number: 144

Time: Tuesday, May 7, 3:30 p.m.–4:25 p.m.

Teaching Evidence-Based Practice through a Free, Modularized Online Tutorial

Sarah Cantrell, Associate Director for Research & Education; **Megan von Isenburg, AHIP**, Associate Dean, Library Services and Archives; Duke University, Durham, NC

Background: Most health professions learners are required to learn skills related to evidence-based practice (EBP). The "Introduction to Evidence-Based Practice" website tutorial was originally created as a partnership between two institutions over 10 years ago, and the tutorial has been a required component in health professions curricula around the world. An opportunity became available to create a new version of the tutorials as part of a larger health system project. As such, the objective was to evaluate the existing tutorial as well as user feedback to inform the development of revised and expanded modules that promote interactivity and engagement.

Description: To determine current satisfaction with content and delivery, we analyzed 5 years of feedback from learners, representing over 10,000 responses. Analysis showed that 89% of learners completed the existing tutorial as a course requirement. Nearly 75% of learners rated the content as easy to understand, almost 80% rated the tutorial as well-organized, and 81% rated the tutorial as easy to navigate. Strengths of the existing tutorial included the quizzes, the ability to practice with cases, and its clear organization. Weaknesses were that it is too wordy, too long, and presented too much information for one training module. Enhancement requests included more videos and opportunities to practice via questions and examples. To address these concerns, two subject matter experts adapted the content from the original tutorial into a series of seven modules.

Conclusion: The resulting new tutorials are available for free online. One new critical appraisal topic was added. New media types were added through the use of blended animation and voiceovers. Interactivity was added by requiring learners to read articles and answer questions about validity. Engagement was promoted through the use of patient cases relevant across disciplines and settings. The existing tutorials are being maintained for a trial period of 1 year. Satisfaction and usage are being monitored to evaluate acceptance and uptake of the new tutorials. Next steps include integrating the tutorials into curricula of undergraduate and graduate medical education.

Poster Number: 145

Time: Sunday, May 5, 3:30 p.m.–4:25 p.m.

The Adventures of Floridiana Jones: Two Web-Based Games for Diabetics, A Pilot Study

Marilyn G. Teolis, AHIP, Clinical Medical Librarian, James A. Haley Veterans Hospital and Clinics, Tampa, FL; **Shannon Bohle**; **Dorothy Kelly**; **Priscilla L. Stephenson, AHIP, FMLA**, Chief, Library Service, Library Service, James A. Haley Veterans Hospital and Clinics, Tampa, FL, Tampa, FL; **Nona Bramlett**; **Janet Sprehe**; **Carla Brunk,**, Clinical Nurse Educator/ VANASP Faculty, James A Haley VA Hospital/ College of Nursing University of South Florida, Tampa, FL; **Edward J. Poletti, AHIP**, Chief, Learning Resources, Health Sciences Library, Learning Resources, Little Rock, AR; **James L. Combs, III**, Graduate Student, Embry-Riddle Aeronautical University, Cottonwood Heights, UT; **Abigail R. Strickland**, Student, University of Central Florida, Port Orange, FL; **Andrew Todd**

Objectives: We conducted a pilot study engaging patients and healthcare providers in two online educational games to augment their knowledge of diabetes management. We anticipated an increased participation in diabetes education and an increased desire to select healthy behaviors after playing online games compared to a traditional classroom approach..

Methods: We surveyed a convenience sample of patients, staff, and volunteers about their game experiences. In the first game, Floridiana Jones and the Pyramid of Life, players assumed the role of Floridiana "Andy" Jones, who along with his guide, Fadil, are searching for the priceless, Golden Ankh Necklace in an Egyptian pyramid. To stay alive, players traversed three levels of the pyramid, encountering many pitfalls amid their hunt for clues and answers until reaching the necklace.

The second game featured Floridiana "Andy" Jones as he faced perils in Peru while searching for the priceless Necklace of Tobacco Cessation. We chose a mixed quantitative/qualitative data collection approach utilizing questionnaires and interviews. Players were asked about the knowledge they gained, their likeliness to change their behaviors, and their level of technology acceptance and engagement with gaming technology for instruction compared with traditional classroom settings.

Results: A majority of Second Life participants were information professionals or had previous experience with the platform. First time users of Second Life commented that they had to invest time to learn to how to use it. Most respondents said they had fun, they would recommend it, and they preferred learning in a virtual world environment rather than a traditional classroom setting. More than 50% of the Second Life users said a virtual world game could be useful in helping them to manage their health and make healthier lifestyle choices. The second game featured anime (Japanese animation) style characters. Participants were required to download and extract a Zip file. Midway through the study, a registered dietician suggested we include pediatric questions for Type 1 diabetic patients.

Conclusions: Participants accepted using technology and gamification to learn about diabetes. A future area of study would be to determine if patients working with their providers who had played the game were more likely to make healthier lifestyle choices than patients who worked with their providers but didn't play the game. We anticipate the use of games as a teaching method will augment self-knowledge of health topics and increase in popularity.

Poster Number: 146

Time: Monday, May 6, 3:30 p.m.–4:25 p.m.

The Ascendance of Health Sciences Librarians' Interprofessional Partnerships: A Scoping Review of the Literature

Susan C. Steelman, AHIP, Head of Education & Research Services, Univ. of Arkansas for Medical Sciences, Little Rock, AR; **Alice Jean Jaggers**, Outreach Coordinator, UAMS, Little Rock, AR; **Lindsay E. Blake, AHIP**, Clinical Services Librarian, University of Arkansas for the Medical Sciences, Little Rock, AR; **Sheila Thomas**, Reference Librarian, UAMS Library, Little Rock, AR

Objectives: Preliminary searches of the literature have shown increasing librarian involvement in non-traditional library service areas. For example, 70% (80) of the 114 PubMed results for clinical/embedded librarians were published in the last 10 years. This study provides a snapshot of the extant literature showing librarian/informationist involvement and inclusion in clinical services, biomedical systematic reviews, translational research and other interprofessional activities.

Methods: An in-depth search strategy was devised and searching began in mid-January 2019. Expert searching techniques were utilized and included the use of MeSH headings and other thesauri dependent on database. Synonyms, truncation and nesting were utilized where appropriate to the database and terminology. Databases include: MEDLINE (including In Process & Daily Update records) via OVID, Cochrane Collection on EBSCO (Central Register of Controlled Trials, Database of Systematic Reviews, Methodology Register, and Clinical Answers), CINAHL Complete and PsycINFO (both via EBSCO) and Web of Science.

Results: A team of four research librarians following accepted scoping review practices will analyze results for trends and themes. Results to be reported include: citations by year and by type of involvement or activity; subject areas of librarian/IPE intersections; and a list of non-librarian professional journal titles most frequently publishing on these topics.

Conclusions: Conclusions will be discussed in the poster and next steps for the research will be reported.

Poster Number: 147

Time: Tuesday, May 7, 3:30 p.m.–4:25 p.m.

The Effect of Moving to a Closed-Stacks Model on Print Collection Access and Circulation

Scott Thomson, AHIP, Library Director, Rosalind Franklin University of Medicine and Science, North Chicago, IL; **Bart Davis**, Electronic Resources Librarian, Boxer Library, North Chicago, IL; **Charlotte Beyer, AHIP**, Instruction and Reference Librarian, Rosalind Franklin University of Medicine and Science, North Chicago, IL; **Bonnie Watterson**, Administrative Director, Boxer Library; **Chelsea Eidbo**, Library Assistant, Boxer Library, Rosalind Franklin University of Medicine and Science, Waukegan, IL

Background: Many libraries are interested in consolidating print collections and moving to a closed-stacks model of service, but concerns over collection access and use often remain a barrier.

Description: Our library consolidated our print collection and shifted to a closed-stacks model of service in order to facilitate 24 hour access to our library space. The print collection was accessible during staffed hours, and traditional document delivery services were offered. In order to assess the impact on collection circulation and perceptions of accessibility, we tracked circulation for the first calendar year, as well as any comments/complaints related to access/service surrounding the print collection.

Conclusion: Circulation for the print collection did not decrease following the switch to a closed-stacks model of service, and the library did not receive any negative feedback. While this poster only documents the experience of one library with a very focused collection, our experience would seem to indicate that collection circulation and patron perception may not be negatively affected by a move to a close-stacks model.

Poster Number: 148

Time: Monday, May 6, 3:30 p.m.–4:25 p.m.

The Impact of Institutional Repositories: A Systematic Review

Michelle Demetres, Scholarly Communications Librarian, Weill Cornell Medical College, New York, NY; **Diana Delgado, AHIP**, Associate Director, Information, Education and Clinical Services, Weill Cornell Medicine, New York, NY; **Drew Wright**, Research Librarian, Weill Cornell Medical Library, Sunnyside, NY

Objectives: Institutional repositories are an ideal outlet to present and publicize an academic institution's output. However, there are many challenges associated with its startup and upkeep. The objective of this study is to concretely define the various impacts that an institutional repository can provide for an academic institution, thusly justifying its implementation and/or maintenance.

Methods: A comprehensive literature search was performed in March 2018 in the following databases from inception: Ovid MEDLINE, Ovid EMBASE, The Cochrane Library (Wiley), ERIC (ProQuest), Web of Science (Core Collection), Scopus (Elsevier), and Library, Information Science & Technology Abstracts (EBSCO). A total of 6,331 citations were screened against predefined inclusion/exclusion criteria.

Results: The 13 included studies were divided into three areas of impact: citation count; exposure/presence; and administrative. Those focusing on citation count (5) and exposure/presence (7) all saw a positive impact for the institution and/or researchers. The one study focusing on administrative benefit saw a unique tie-in with automated ORCID profile population.

Conclusions: Based on the available literature, institutional repositories do appear to have a positive impact in the following areas: citation count, exposure/presence, and administrative burden. In order to make more concrete conclusions, additional, higher quality studies are needed.

Poster Number: 149

Time: Monday, May 6, 3:30 p.m.–4:25 p.m.

The Implementation of Professional Accreditation of Medical Librarians in Taiwan and Attributes Analysis of Accreditation Exam Candidates in 2017-2018

Tzu-heng Chiu, University Librarian, Taipei Medical University, Taipei, Taipei, Taiwan (Republic of China); **Shu-Yuan Siao**, Head, Division of Knowledge Services, Taipei Medical University Library, Taipei, Taiwan (Republic of China)

Objectives: After one-year planning, the Taiwan Medical Library Association (TMLA) launched its first accreditation exam of medical librarians in Aug 2017. Candidates who pass the exam will receive a "Professional Certificate of Medical Librarianship in Taiwan". This poster introduces the origin, content, and implement of the accreditation exam and analyze attributes of the examination candidates in 2017-2018.

Methods: The exam lasted for 90 minutes with multiple choice and essay questions, candidates who get the score higher than 70 pass the exam and will receive the Certificate issued by TMLA. In 2017, there were 47 medical librarians registered for the exam and 37 pass (78.7%); In 2018, 21 registered and 15 pass it (71.4%). The authors coded the demographic data in registration forms of each candidate with his/her exam score. And then apply the SPSS software to conduct the descriptive statistic, ANOVA and Scheffe's method, trying to investigate the correlation between exam scores and candidates' personal attributes, such as age, sex, education degree, subject background, time of graduation, and type of medical libraries.

Results:

Among the candidates, most of them are in the age of 30-39 (34.3%) and 40-49 (34.3%), and 92.5% are female. Around 3/4 have a bachelor degree and 4/1 have master degrees. 72.4% graduated from LIS related departments. The distribution of their time of graduation are quite even: 21.1% graduated from schools less than 5 years, 19.3% for 6-10 years, 22.8% for 11-15 years, 12.3% for 16-20 years, and 24.6% are more than 21 years. And 41.9% of them work in regional hospital libraries and 29.0% in libraries of medical centers. As to the performance of their accreditation exam, 40.3% of them receive the scores of 80-89 and 31.3% are 70-79. The ANOVA analysis shows that "subject background" significantly influence the "exam score" ($P<0.01$), and "education degree" and "type of medical libraries worked" are also the influential variables for scores ($P< 0.05$). Researchers then apply Scheffe method to test the differences between groups. We found the influence of "subject background" to the "exam score" are significant differences between score groups of 60-69 and 70-79, and 60-69 and 80-89. (see tables)

Conclusions. The accreditation exam of medical librarians had been held for 2 years and the total pass ratio is 77.6%. Research findings could serve as reference for future promotion of this Professional Certification and the planning of related CE courses of TMLA.

Poster Number: 150

Time: Tuesday, May 7, 3:30 p.m.–4:25 p.m.

The Journal of the Medical Library Association's Data Sharing Policy: What, When, Why, and How?

Katherine Goold Akers, Biomedical Research & Data Specialist, Wayne State University, Detroit, MI; **Kevin Read**, Lead of Data Discovery/Data Services Librarian, NYU School of Medicine, New York, NY; **Liz Amos**, Special Assistant to the Chief Health Data Standards Officer, National Library of Medicine, NIH, Bethesda, MD; **Lisa Federer, AHIP**, Data Science and Open Science Librarian, National Library of Medicine, North Bethesda, MD; **Ayaba Logan**, Research and Education Informationist, Medical University of South Carolina, Charleston, SC; **T. Scott Plutchak, AHIP, FMLA**, Retired, University of Alabama at Birmingham, Birmingham, AL

Background: To increase research rigor and reproducibility, allow data re-use, and foster open science, the Journal of the Medical Library Association (JMLA) will enact a Data Sharing Policy beginning October 1, 2019. This poster will provide more information about this policy, including our rationale for its implementation, definition of "data", exceptions to the policy, and acceptable data repositories. The journal editor will be available during the presentation window to answer any questions.

Description: As health sciences librarians and information professionals have long been advocates of data sharing, it is time to practice what we preach and share the data associated with our published research. In 2017, a task force began the process of developing a data sharing policy for the JMLA based on the opinions of recent authors, review of other journals' data sharing policies, and feedback from key MLA groups. Upon policy implementation, authors of Original Investigation and Case Report articles will be required to (1) place the de-identified data associated with the manuscript in a repository and (2) include a Data Availability Statement in the manuscript describing where and how the data can be accessed. Exceptions to this policy will be made in rare cases in which de-identified data cannot be shared due to their proprietary nature or participant privacy concerns.

Conclusion: We hope that health sciences librarians are excited by the prospect of sharing the data associated with their published articles in accordance with the new JMLA Data Sharing Policy and leading by example for their user communities. As the corpus of health sciences library and information research data grows under this policy, so will the integrity of our research processes and findings. The more we share data and encourage others to do the same, the better chance we have of increasing collaboration within and outside our domain, making new discoveries, and becoming a leading field in research data sharing practices.

Poster Number: 151

Time: Sunday, May 5, 3:30 p.m.–4:25 p.m.

The National Network of Libraries of Medicine (NNLM) All of Us Community Engagement Network at the New Orleans Public Library

Elaine R. Hicks, Research, Education, and Public Health Librarian, Tulane University, New Orleans, LA; **Dana Wilkosz**, Health Literacy Educator, New Orleans Public Library, New Orleans, LA

The NIH All of Us Research Program charged the National Library of Medicine with developing a community engagement program with public library partners. Southeast Louisiana, in which New Orleans is located, has a genetically-diverse population living in a medically-under-served area. The National Network of Library of Medicine South Central Region (NNLM SCR) gave an award to the New Orleans Public Library to establish a New Orleans-based All of Us Community Engagement Network. An MPH-trained Health Literacy Educator and Expert-on-Call Health Educator developed local program goals to improve health literacy, engage local communities, and raise awareness about the program among a population underrepresented in biomedical research.

The program is designed to improve health literacy among patrons and train public librarians to identify authoritative online health information. Program activities include:

- Developing and implementing patron health education programs

- Participating in community outreach to promote the All of Us Research Program

- Creating health promotion partnerships among community organizations

- Providing librarian in-service training to increase knowledge and skills about authoritative online health resources.

Program Assessments: Two assessments guide the program: a process assessment and an impact assessment. The Health Literacy Educator conducts a program needs assessment for staff and patrons online and in each branch. Ongoing responses determine which health topics patrons are most interested in at the branch level. The New Orleans Public Library uses the Public Library Association’s Project Outcome Education/Lifelong Learning survey for a process assessment. The NNLM SCR provides evaluation forms to assess training on the use of health resources.

Conclusion: NNLM SCR monthly and activity reports are completed in order to assess the number of health education programs developed and conducted or organized, the number of needs assessments collected, the number of potential partnership meetings attended, the amount of All of Us materials distributed, and the number of All of Us sessions presented. At this early stage in program development, the NNLM SCR has not provided measurable outcomes.

Poster Number: 152

Time: Monday, May 6, 3:30 p.m.–4:25 p.m.

The PULSE of Collaboration: Supporting the Pipeline

Amanda Adams, Reference & Instruction Librarian, Cooper Medical School of Rowan University, Camden, NJ; **Alisha Crawford**, Multimedia Specialist, Cooper Medical School @ Rowan University, Camden, NJ

Background: In 2011, a pipeline program to give underrepresented populations exposure to in healthcare. Initially organizers asked the library to research similar programs to aid in designing courses and experiences aligned with the PULSE mission. The Library also assisted students with capstone posters. Every year, the library has upgraded instruction support beyond poster design into better poster development. This collaboration provides students with research, design, and teamwork skills. The Multimedia Specialist and Reference Librarian are now embedded within CMSRU's summer programs.

Description: PULSE started with one phase of thirty students, during which the Library Multimedia Specialist assisted students with poster design. In the next iteration of library collaboration, the Multimedia Specialist was asked to conduct poster workshops. Each year the number of undergraduates has increased, separated by three phases. Debrief sessions assessed student needs and proposed changes. As a result, library support expanded. The Multimedia Specialist and Reference Librarian partnered to develop and co-teach a series of workshops. Starting with:

•Researching using library databases

•Abstract writing

•Team building

•Poster design and image copyright

As reinforcement, the Multimedia Specialist created video tutorials. Library staff and Office of Diversity staff divide students into groups to lead, facilitate and provide feedback on refining the research topic, abstract text, poster design, and presentation. Using instructional scaffolding, student teams learn to build successful poster capstone projects.

Conclusion: The library supported PULSE since inception, developing start to finish training in poster design that also facilitates mastery of information literacy. Debrief sessions and workshop refinements will based on survey evaluations. In eight years, the poster workshops have engaged more than 360 undergraduate students in library research, abstract writing, and team-based learning. The Multimedia Specialist and Reference Librarian's partnership represents a unique, rewarding collaboration. Their responsibilities have expanded, transforming the library's part in the pipeline. Each part of the capstone project supports student achievement, and equips them with skills necessary for success in higher education and the workforce.

Poster Number: 153

Time: Tuesday, May 7, 3:30 p.m.–4:25 p.m.

Transforming Student Reference Training: Developing Functional Skills Using E-Learning Modules

Antonia Osuna-Garcia, Health and Life Sciences Librarian, UCLA Biomedical Library, Los Angeles, CA; **Bethany Myers, AHIP**, Research Informationist, UCLA, Los Angeles, CA; **Courtney Hoffner**, Librarian, , Los Angeles

Background: Steadily declining patron engagement at the Sciences Libraries reference desk has resulted in a lack of professional experience for student employees, rendering our time-intensive training model inefficient. The UCLA Library recently reorganized into a functional matrix that placed employees into both a division and a functional group. We saw an opportunity to transform our reference student roles into those functional groups and hire and develop students with dynamic skill sets. We decided to critically evaluate our traditional reference program to make the service and training more efficient, provide greater opportunity for student employee skill development, and complement this new organizational structure.

Description: Using our new organizational structure, we conceived a collaborative, functional approach to student employment, one where students can focus on areas of skill and interest and build on their training over time. These new functional roles include: research assistance, outreach, teaching and learning, collections, research partnerships, as well as basic core library services. After developing our model, we used the university's course management system to create interactive training modules, quizzes, thought questions, and collaborative forums to allow the students to complete training through solo work, peer work, and reduced one-on-one time with librarian supervisors. This has significantly reduced staff training time. We also used existing students to assess and contribute to the design. Once students complete basic and low level training, higher level tiers can be built over time.

Conclusion: Our main outcomes are a significant decrease in intensive student training for librarians and improved assessment of skills using online quizzes and modules. Our hope is that a student that is hired to work at the UCLA Library will have a meaningful and educational experience they will be able to leverage to expand their professional goals. The UCLA Library librarians will help them achieve this by developing a student-centered employment program, mentoring and training the students, and providing them with feedback. The result will be a formalized approach to collaborative student employment that will benefit both the Library and students.

Poster Number: 154

Time: Sunday, May 5, 3:30 p.m.–4:25 p.m.

Transitioning to Single Use Gender Neutral Restrooms: Elevating Our Facilities

Victoria Rossetti, Education and Clinical Services Librarian, University of Massachusetts Medical School, Worcester, MA; **Mary Piorun, AHIP**, Director, UMass Medical School, Worcester, MA

Background: The Lamar Soutter Library serves the University of Massachusetts Medical School. Existing facilities in the library include 6 single use restrooms spread out over 3 floors, with 2 on each floor (1 for men, 1 for women). The library averages 500 visitors a day, and our 1st floor area is the most highly trafficked. Admission trends indicate that class sizes are increasing, and the number of women students is trending up; we decided to look at ways to increase the number of facilities that could be used by anyone regardless of gender by making some of the restrooms gender neutral.

Description: Library management were early and necessary champions of this project, taking the idea to school administration and getting approval for changes and expenditures. We also consulted the Diversity and Inclusion Office and the LGBTQ Resource group lent support and offered opinions on signage choice and goals for implementation. The MLA LGBTQIA SIG also offered information on how their libraries managed this transition, providing examples of signage that conveyed the message that the restroom was for anyone without also enforcing the gender binary. The building manager led discussions on what was possible with the existing space and building codes, and worked with us to preserve handicap access.

Conclusion: While our renovation is not complete at this time, when it is we will assess user satisfaction with the space in general, as well as whether there is an increase in how many users we average daily. Another measure of success would be whether other areas of the school follow suit and offer gender neutral restrooms. We would also be interested in converting the remaining 4 restrooms in the library. We hope to create an environment that is flexible and welcoming, and accommodates all of our users.

Poster Number: 155

Time: Monday, May 6, 3:30 p.m.–4:25 p.m.

Understanding Our Impact: Analyzing Librarian Involvement with Systematic Reviews

Hannah J. Craven, Liaison/Research Librarian, Indiana University School of Medicine, Indianapolis, IN; **Christi Piper**, Reference Librarian, University of Colorado Anschutz Medical Campus, Aurora, CO; **Kristina Palmer**, , Arvada, CO

Objectives: On a medical campus, systematic reviews with librarian co-authors compared to reviews without librarians were published in journals with lower impact factors, although still within the comparative range. To try to determine why, discipline and authors' publishing experience were examined. The bibliographic analysis was also expanded to see if there is a difference in the journal ranks by discipline.

Methods: Search strategies were created to pull systematic reviews published in the last five years by campus authors from PubMed, Ovid MEDLINE, and Web of Science. Citations were exported and deduped using EndNote X8. The systematic reviews were grouped by whether a librarian from our campus assisted with the search or not by searching for librarians' names in the author field. A statistically appropriate number of articles without a librarian author were randomly selected for comparison with articles that had librarian assistance. Selected articles were analyzed based on Journal Impact Factor for the year of publication, journal rank by discipline, authors' discipline(s), and years of authors' publication experience. Authors' years of experience are determined by the date of their first published article.

Results: Systematic reviews with the assistance of a librarian were statistically no different from those without a librarian in terms of the Journal Impact Factor or journal rank by discipline where systematic reviews were published. Years of experience significantly differed between groups, with librarians assisting most authors with 5 years or fewer of experience. The departments who utilized librarians for systematic searching the most were: General Medicine, Orthopedics, and Gastroenterology.

Conclusions: This exploratory research helped evaluate who our librarians are primarily working with on systematic reviews. It also informed us that we do not have an impact on the systematic review being published in a higher impact journal based on Journal Impact Factor or rank by discipline. Our liaison efforts will focus less on the three departments listed above as they already utilize our service. Since most of the authors we assisted had 5 years of experience, we will target the campus faculty onboarding orientation.

Poster Number: 156

Time: Tuesday, May 7, 3:30 p.m.–4:25 p.m.

Use of Professional Statistician Positively Impacts Library Physical Space Usage Study

Lori A. Fitterling, University Library Director, Kansas City University of Medicine & Biosciences, Kansas City, MO; **Nora Franco**, Consumer Health Librarian, National Network of Libraries of Medicine Pacific Southwest Region, Los Angeles, CA; **Kristy E. Steigerwalt**, Head of Clinical & Research Support, University of Missouri-Kansas City, Kansas City, MO; **Sarah McQueeny Kartsonis**, User Services Manager, A.R. Dykes Library of the Health Sciences, Kansas City; **Marie A. Thompson**, Dental Librarian, University of Missouri-Kansas City Health Sciences Library, Kansas City, MO; **Mariah L. Harvey**, Head of Electronic Resources & Acquisitions, University of Missouri-Kansas City, Kansas City, MO

Background: In the study of space usage and allocation in medical libraries, few studies have employed the use of professional statisticians to assess and evaluate their data. Though statistics are a necessary element for improving evidence-based research, the use of professional statisticians to ensure quality study design and analysis is scant in library studies. This lack of professional expertise for data assessment can produce incorrect application of statistical methods, data analytics, and false outliers, which may invalidate research.

Description: This poster chronicles a recent methodological study from four libraries concerning physical space usage and their experience of first submitting for publication without consulting a professional statistician. The researchers subsequently consulted a statistician, who re-analyzed and formatted the data using predicted probability ratings with identified outliers factored into final statistical results. Data was then visually displayed using graphs and images to illustrate specific research results such as the percentages of total patron usage of library spaces compared across four libraries. Odds ratios identified the effects of allocated seating and time of day with the likelihood of a patron using a particular space in a library. Final results provided a predicted probability ratio of patron space usage per library.

Conclusion: We showcase here the data management processes offered as an example of the positive impact of retaining a professional statistician. The consultation and subsequent data analysis contributed to the acceptance of our publication and to the pool of evidence-based library research. Libraries interested in improving their publications and focusing on reproducibility of results may consider adding the services of a professional statistician to their library research projects.

Poster Number: 157

Time: Sunday, May 5, 3:30 p.m.–4:25 p.m.

Use Trends by Medical Students at an Academic Medical Center in the Third and Fourth Clerkship Years

Lindsay E. Blake, AHIP, Clinical Services Librarian, University of Arkansas for the Medical Sciences, Little Rock, AR; **Pamela Fall**, Professor Emeritus, Medical College of Georgia, Augusta University, Augusta, GA; **Ashley Saucier**, Associate Director of Medical Student Education, Family Medicine; **Shilpa Brown**; **Erin Z. Latif**, Associate Professor, Medical College of Georgia at Augusta University, Augusta, GA; **Elena A. Wood**, Associate Professor, Medical College of Georgia at Augusta University, Medicine/Academic Affairs, Augusta, GA; **Thaddeus Carson**, Clerkship Director, Augusta University, AUGUSTA, GA; **David L. Kriegel, II**, Associate Professor Director of Student Education, Augusta University, Department of Family Medicine, Augusta, GA; **Matthew Diamond**

Objectives: The objective of this project is to review how 3rd and 4th year medical students use clinical medical literature to practice evidence-based care as they move through their clerkship years.

Methods: A Clinical Librarian and Medical Educator polled Clerkship Directors in required and elective medical rotations. Four required clerkships and one elective rotation had assignments which required the use of literature resources as well as the listing of consulted sources. The Directors of Family Medicine (FM), Internal Medicine (IM), Obstetrics and Gynecology (OB/GYN), Ambulatory Medicine (AM), and Nephrology provided these assignments throughout the 2016-2017 academic year. The Clinical Librarian, Medical Educator, and research assistant pulled the citation information using a modified scoring sheet from Vanderbilt University. The data was then sent to a statistician for analysis.

Results: Comparison across the 3rd year clerkships showed the highest mean use of citations in FM at 5.21 and lowest in OB/GYN at 3.63. In the 4th year, the Nephrology elective had the highest mean number of citations at 3.01 with AM at 0.66. Comparing the 3rd and 4th years, the 3rd year had significantly more citations and cited significantly higher numbers of systematic reviews, guidelines, and journal articles.

Conclusions: This study currently has only local applicability, but shows little consistency among the clerkships or between the 3rd and 4th years. The high use of websites and textbooks shows a need for more training on accessing and using clinically oriented databases. Creation of standard requirements and librarian involvement in creating curriculum for searching and application of information could greatly improve student ability to use resources. The study could be expanded to explore information literacy curricula in other health sciences colleges and medical schools.

Poster Number: 158

Time: Monday, May 6, 3:30 p.m.–4:25 p.m.

Using Bibliometric Analysis and Visualization to Identify Current and Potential Collaborators

Jordan Wrigley, Graduate Student/Intern, University of North Carolina - Chapel Hill, Carrboro, NC; **Megan von Isenburg, AHIP**, Associate Dean, Library Services and Archives, Duke University, Durham, NC; **Virginia Carden, AHIP**, Administrative Research Librarian, Duke University, Durham, NC

Background: To facilitate enhanced collaboration and team science, various methods to detect and analyze current and potential collaborative research communities have been developed. This project characterized current research and collaboration patterns in pain research at one institution after researchers working on a grant application approached the library to better understand current institutional research and publishing on that topic. To address this question, library staff developed a collaborative, multi-tool process for bibliometric analysis and network visualization.

Description: No specific research impact department exists within the library; therefore, staff worked collaboratively to identify existing data and tools to address the research questions. The primary data source used was a pre-existing, curated EndNote library of institutional publications. This library was searched using keywords relevant to the topic to create two sub-libraries: one on pain and one specifically on musculoskeletal pain. Article data from each library were exported into InCites to create benchmarking analysis. Results were ranked by selected variables to highlight existing institutional successes in pain research and publishing. In addition, article data were imported into VOSviewer to visualize collaboration networks by author and by topic. Researchers were consulted to identify and label resulting clusters within the VOSviewer visualizations.

Conclusion: Data visualization is an invaluable tool in detecting and analyzing current and potential collaborative research. This project successfully generated a number of useful visualizations that characterized current and potential pain research at this institution. The analysis was included in a grant proposal for funding a center for pain research and for catalyzing further collaborative research.

Poster Number: 159

Time: Tuesday, May 7, 3:30 p.m.–4:25 p.m.

Using Branding and Design Concepts to Elevate the Library on Campus

Meredith I. Solomon, AHIP, Outreach Officer, Harvard Medical School, Boston, MA; **Robin J. Horst**, Consultant, Ms., MAINEVILLE, OH; **Stephanie Worrell**

Background: Countway Library of Medicine sits in the middle of a large, busy medical campus. To elevate its presence beyond that of a library known for its resources and research/study space, the library engaged brand strategists to help change its users' perception. The overall goal of this program is to build awareness of the library as a nurturing access point for knowledge and growth.

Description: Countway Library of Medicine used brand architecture and strategic messaging to discover its most promotable strengths, identify how to best serve users and redefine its brand positioning as the essential campus resource that connects everyone through learning, wellness and enrichment. Using strategic brand building tools the library articulated its brand 'positioning' (this is a strategic statement that defines what a brand stands for, why it's different from its competitors and why users should care about it) and enhanced its image as a caring provider of skill-building, wellness and enrichment focused classes and events.

Conclusion: To gain a deeper understanding of the library as a brand, enhance its offerings and identify its strengths/weaknesses

To elevate classes/events by organizing them under a strategic platform that supports brand positioning

To create strategic messaging to influence attitudes, share information and promote community

Poster Number: 160

Time: Sunday, May 5, 3:30 p.m.–4:25 p.m.

Using Excel Formulas for Systematic Review Search: Time Savings, Error Reduction, and Carpal Tunnel Relief

Amelia Brunskill, Visiting Assistant Professor & Information Services and Liaison Librarian, Library of the Health Sciences, University of Illinois at Chicago, Chicago, IL

Background: Developing a search strategy for a systematic review is a time consuming and highly iterative process in which small errors can have large consequences. The process can also involve very repetitive hand motions which may exacerbate existing physical issues such as carpal tunnel. As such, mechanisms that would reduce the amount of time and physical repetition associated with compiling this search strategy, while also constraining the possibilities for manual errors, seemed highly advantageous.

Description: Since Excel is part of the standard Office suite and provides a rich array of tools for automating the formatting of data, it seemed like a natural fit for simplifying the formatting and compilation of terms for a systematic search strategy. Ultimately, Excel formulas were created to address the following issues: insertion of desired search field information, introduction of needed Boolean operators between terms and concepts, and correction placement of quotation marks and parentheses. Using these formulas, a spreadsheet was produced to compile search terms and controlled vocabulary terms for three concepts, resulting in a full search string for five different databases.

Conclusion: While only recently created, this spreadsheet has already proven useful for working with graduate students and faculty. Although its approach may not be an optimal fit for all search strategies, it could be helpful for users ranging from experienced searchers to interested novices, and to this end a video was created demonstrating how to use the spreadsheet.

Poster Number: 161

Time: Monday, May 6, 3:30 p.m.–4:25 p.m.

Using LibGuides to Improve Public Health: The Opioid Crisis

Amy Faltinek, AHIP, Senior Director, Technical Services, TTUHSC Preston Smith Library of the Health Sciences, Lubbock, TX

Background: The purpose of a LibGuide that addresses a public health issue is to provide quality information when the amount of available information is overwhelming. This guide includes academic resources and online resources related to the opioid crisis. It includes the following academic resources: databases, ejournals, and ebooks. These resources are available to faculty, staff, and students, as well as community members who visit the libraries. The online resources, available to all Internet users, cover a variety of groups, from consumers to professionals, in an A to Z list of topics, from apps to the workplace, affected by the crisis.

Description: Library resources were scanned for opioid-related topics and then highlighted under the databases, ejournals, and ebooks tabs. One way ideas for online resources were collected was by emailing a suggestion request to the Substance Abuse Librarians and Information Specialists (SALIS) Listserv. Another way online resources were collected was an Internet search that included other opioid LibGuides, professional organizations, and communities affected by the opioid crisis. Information was collected from reliable sources and evaluated for accuracy, authority, bias, timeliness, and validity. The initial topics chosen were: apps, consumer health, counseling/psychiatry/therapy, dentistry, educational/training resources, guidelines, healthcare providers, libraries, obstetrics, older adults, opioid overdose reversal, rural health, school/education resources, Spanish language resources, Texas, veterans, veterinary, and the workplace.

Conclusion: The Opioid Resources LibGuide was first published November 16, 2018. It will continue to be updated and added to as new resources are collected. The use of the LibGuide is expected to be measured through the statistics feature that measures views. Another possible way of collecting information is by survey and may include how the guide is being used, who is the using the guide, and which sections are being used and/or what sections should be added.

Poster Number: 162

Time: Tuesday, May 7, 3:30 p.m.–4:25 p.m.

Using LibGuides to Support Faculty Development and Promote Library Resources

Jeremy Gunnoe, Assistant Director of Operations, Howard University, Washington, DC; **Fatima M. Mncube-Barnes**, Executive Director, Louis Stokes Health Sciences Library, Arlington, VA

Background: There is a need to centralize guidelines, policies, training materials and general information for both new and existing faculty members of the College of Medicine. In collaboration with the Associate Dean of Faculty Development, a librarian created a LibGuide with multiple tabs and subject headings to upload information, videos and articles that allowed easy access and continual updates. This material is also relevant to faculty members of three other health sciences colleges.

Description: The library's website is currently hosted by Springshare and already contains multiple LibGuides for the health science colleges and individual subject areas. A "College of Medicine – Faculty Development" guide was created and has tabs for new and existing faculty members in both the clinical and basic science areas. These include policy materials, information on library resources, direct links to the most used textbooks, journals and articles, training materials on teaching tools and methods, and links to continuing education resources. Additional tabs are created as more information is made available from faculty orientations, retreats and meetings. This includes presenter videos and materials, as well as, updates and new information shared with faculty at these events. Information about this guide has been shared at faculty orientations, retreats and in workshops where the library has presented to faculty.

Conclusion: This LibGuide has been a successful tool for sharing and improving communication and collaboration with the library and the College of Medicine and their Office of Faculty Development. To date, the faculty development LibGuide is the third most accessed guide on the library website out of 40+ available guides. New information is continually added, and materials continue to be updated to the most recent versions available. This avenue has allowed for the library to perform outreach and market its resources while providing a vital resource to health science faculty members.

Poster Number: 163

Time: Sunday, May 5, 3:30 p.m.–4:25 p.m.

Using REDCap to Collect Library Service Statistics in a Large Health System

Carrie Grinstead, AHIP, Regional Medical Librarian, System Library Services, Providence St. Joseph Health, Burbank, CA

Background: In January 2018, library services at Providence Health/Swedish and St. Joseph Health merged to form a single, unified system, incorporating nine libraries and sixteen full-time staff. We faced several challenges in collecting and reporting service statistics, including the disparate methods of our legacy organizations, the variation in services offered at different libraries, and our large geographic spread. Our merged library serves patrons in seven states, and much of our work, including communication with patrons and each other, is conducted over email.

Description: We chose a tool, REDCap, early in our process. REDCap is easy to use and allowed us to build a web form that would be accessible on site and off, and regardless of legacy organization. REDCap allows for quick and easy reporting, facilitating our annual infographic and allowing us, for the first time, to produce monthly usage reports.

One librarian compiled a list of items that had been tracked at each hospital library. We then convened a small task force to decide what information we would collect and at what level of detail. This group included representatives each legacy organization. We considered issues such as accurate representation of the various services performed at each site and usefulness for reporting out. We then built a sample form and invited all team members to test it for one month.

Conclusion: Our REDCap form for statistics collection went live on April 1, 2018. The form is simple to use, and REDCap's branching logic functions allow us to collect detailed information without making the form unwieldy. Occasional communication and retraining for our team has been necessary, to ensure that fields in the form are understood and used consistently. Finally, we are using our reports to assess and further refine the form for 2019. Some fields, such as the purpose of a request, are rarely used and may be eliminated, while new fields may be added in consultation with the team.

Poster Number: 164

Time: Monday, May 6, 3:30 p.m.–4:25 p.m.

Using Mixed Methods Evaluation Data to Improve an Anesthesiology Resident Research Curriculum

Natalie Tagge, Head, Podiatry Library, Temple University Health Sciences Libraries, Philadelphia, PA; **Jenny Pierce**, Head of Research, Education and Outreach, Temple University, Philadelphia, PA; **Stephanie C. Roth, AHIP**, Biomedical & Research Services Librarian, Temple University Health Sciences Library, Philadelphia, PA

Background: The Library developed a research curriculum in collaboration with the Anesthesiology residency director. Beyond accreditation pressure from ACGME, which mandates research output and quality improvement projects in residency, the director was motivated by a desire to provide residents with an improved research skill set. She saw expertise in the library not found in her department, including, a depth of knowledge in teaching evidence based medicine. The overarching goal of the library curriculum is to teach Anesthesiology residents the skills needed to conduct and present research and quality improvement projects. Each course is created from objectives developed with the director.

Description: The anesthesiology residency program director approached the library to create a curriculum after librarians presented an educational session during grand rounds. The library collaborated with the director to develop courses. The Education Librarian taught courses on topics such as evidence based medicine and brought in other librarian's expertise for topics such as systematic reviews. The first round of the library curriculum was challenging with scheduling issues and residents questioning the curriculum's value. Drawing on librarians' observations, meetings with the residency director and head residents, and resident survey data, improvements were made. These include mindful session scheduling that avoids test preparation periods and adding courses of interest, such as poster presentation best practices. Since the changes have been implemented 92% of the resident evaluations have strongly agreed or agreed that "The content covered in today's workshop will be useful to me."

Conclusion: This Library's successful improvement of a residency curriculum demonstrates that libraries can provide important instruction and expertise to initially resistant residents. Curriculum can be improved by collecting and utilizing evaluation data from a variety of sources including residents, residency directors and librarians. The development and improvement of the resident curriculum is an iterative process, so evaluation will continue and changes will be made based on this data. The Library's development of a successful Anesthesiology residency curriculum has been leveraged for outreach into other residency programs, such as Internal Medicine and Radiology.

Poster Number: 165

Time: Monday, May 6, 3:30 p.m.–4:25 p.m.

Using Screencasting to Capture Metacognitive Processes in Health Sciences Information Literacy Instruction

Shaun R. Adamson, Education Librarian, Head of Teaching & Information Services; **Jason Francis**, Health Science Librarian; Weber State University, Ogden, UT

Background: This exploratory project used screencasting to replace traditional written assignments in several for-credit information literacy courses emphasizing health sciences. These assignments relied on the think aloud protocol (TAP), associated with usability studies, to capture not only the results of their searches, but students' decision-making and selection processes. The advantage to this method is that there are no interruptions, suggestive prompts, or questions as they go through the process. They are just asked to narrate what they are doing as they complete the task. Because their conscious effort is focused on task completion, it does not interfere with task performance.

Description: Screencast assignments replaced traditional written assignments in 12 for-credit university information literacy courses. Students completed three screencasts; each was five minutes or less. Each screencast assignment was designed to mirror assignments that had previously been assigned in print, and included four objectives:

1. Develop and apply search statements
2. Use specific tools to locate specific types of sources
3. Describe/summarize the source
4. Establish the relevance of the source to their topic

While the screencasts followed the typical TAP protocol, they were given a list of required items to address in their narratives. Screencasts were graded using a rubric aligned to the required content list. Effectiveness of screencast vs. print assignments was determined by looking at pre- post-test scores, comparing the type of information each type of assignment provided, and analyzing student comments related to the assignments.

Conclusion: Post test results for students who did screencasts compared to those who did print versions of the assignment showed a higher level of mastery of the course learning outcomes. Student discussions reflected a clear appreciation for the value of screencasting as a learning tool. Unlike print assignments, which show only the end results of the search process, screencasting captured the why and how as students navigated through the process to find relevant resources. Further study might include the development of authentic pre- and post-assessment screencast portfolios to provide a more holistic picture of student knowledge progression in an information literacy course.

Poster Number: 166

Time: Sunday, May 5, 3:30 p.m.–4:25 p.m.

Using Text Mining Tools to Generate Terms for a Systematic Review: A Comparison of Voyant Tools and R

Bethany S. McGowan, Assistant Professor of Library Science and Health Sciences Information Specialist, Library of Engineering and Science, Purdue University, West Lafayette, IN

Objectives: Does the use of text analysis tools, like Voyant Tools and R tm, generate new citations for a systematic review search when compared to traditional techniques? Can librarians who want to use text analysis tools but prefer not to use R, generate the same results with Voyant Tools--a text analysis tool whose use does not require coding knowledge?

Methods: This research project executes the search for a systematic review using three approaches, then discusses and compares the results of each. The first approach uses traditional techniques for building a search, specifically use of MeSH, subheadings, text words, search filters, synonyms/acronyms, generic and brand name use, lay and medical terminology, variant spellings, and truncations. The second approach follows Hausner et al.'s 2012 4-step search strategy process, which uses the R text mining tool. The third approach also follows Hausner's 4-step search strategy but replaces the use of the R text mining tool with the use of Voyant Tools. All approaches include consultations with the research team, disciplinary faculty and doctoral students considered subject experts.

Results: The results of all three searches were very similar, however, there are other outcomes to consider including time invested, opportunities to connect with students and faculty, and opportunities to improve search transparency. The traditional development of the search strategy took the most time as it required multiple, in-person meetings with the research team. These meetings, however, allowed the librarian to build closer relationships with students and faculty that can be leveraged in the future. Use of R tm was also time-consuming, as the text mining application has a steep learning curve. Voyant Tools was easy to learn and generated similar useful results as R tm.

Conclusions: Designing search strategies is part art, part science and it is difficult to develop and describe a completely objective search strategy. Even utilizing Hausner's more objective approach requires subjective decision making, particularly when selecting candidate terms to use to develop final search strategies and in situations where existing related systematic reviews do not exist. Following Hausner's method does, however, help to quickly generate search terms, provides a means to validate search strategies and improves transparency by providing an easy to follow process

Poster Number: 167

Time: Monday, May 6, 3:30 p.m.–4:25 p.m.

Using the PubMed Central Data Availability Statement Search Filter and an Institutional Data Catalog to Make Data More Discoverable

Nicole Contaxis, Data Catalog Coordinator, NYU Health Sciences Library, New York, NY; **Ian Lamb**, Solutions Developer, NYU Health Sciences Library, New York, NY; **Alisa Surkis**, Assistant Director, Research Data and Metrics/Vice Chair for Research, NYU Health Sciences Library, New York, NY; **Kevin Read**, Lead of Data Discovery/Data Services Librarian, NYU School of Medicine, New York, NY

Background: Data Availability Statements (DAS's) are included in journal articles to provide information on the data used, including whether it is available and how to access it if possible. However, in order to locate this information, researchers need an awareness of and the initiative to look for a DAS. To make this data more discoverable, librarians and a developer at an academic medical center leveraged a new search filter from PubMed Central (PMC) to index datasets described in DAS's in their institutional data catalog.

Description: Using the "has data avail" filter in PMC, which allows users to narrow their search to journal articles with data availability statements, we pulled 517 articles from PMC that included DAS's and were written by researchers at our institution. Using this sample, we categorized the different types of DAS, established inclusion criteria for cataloging, created a scheduled search alert to identify newly published articles by our research community that included a DAS, and developed a sustainable workflow for identifying datasets for inclusion, indexing them, and notifying researchers when they are published in the institutional data catalog.

Conclusion: In the pilot phase of this project we grouped the 517 DAS's into five categories: data is available through the author (79 statements); through application to a consortium or committee (13); in a repository (99); in the supporting information files (148); and in the paper itself (170). Inclusion criteria was developed as some statements did not clearly identify the data location or availability. To date, seven datasets have been cataloged. Next steps include continuing to catalog these datasets and expanding the scope of the project beyond PMC.

Poster Number: 168

Time: Tuesday, May 7, 3:30 p.m.–4:25 p.m.

Utilizing a Collaborative Partnership between Health Sciences Faculty and Librarians: Building a Course Introducing the Interdisciplinary Nature of Health Care

Jason Francis, Health Science Librarian; **Shaun R. Adamson**, Education Librarian, Head of Teaching & Information Services; **Kathryn Newton**, Associate Professor; **Aaron L. Ashley**, Professor; Weber State University, Ogden, UT

Background: We developed a course through the collaboration of health science faculty and librarians introducing disease processes and the interdisciplinary nature of health care through case study models. While many collaborative ventures include librarians in their more traditional support functions, this project took advantage of an interdisciplinary team that combined librarians' backgrounds in medical informatics and evidence-based medicine, pedagogy, and educational technology, and health science faculty's content knowledge in allied health. Components of the course include increasing knowledge of the role of health professions, which is key to avoiding stereotyping and thereby increasing the ability to work as a healthcare team.

Description: Twelve medical case studies were built online utilizing resources provided by both our Medical and Education librarians. Health Science faculty built each case around content knowledge of a disease process, while collaborating with medical and education librarians to provide resources hyperlinked within each case. Healthcare professionals are introduced as an interdisciplinary team. Objectives of each case include a review of normal physiology, pathophysiology, medical terminology, diagnostic testing, treatment, and health professions. As an ancillary component of the course, students are required to present peer-reviewed literature supporting medical ethics topics. The course is offered to both university allied health and high school students, enrolling in the course for both university and high school credit. Assessment of knowledge of health profession roles was gathered through a pre-post-test survey, administered to 582 High School students throughout the state of Utah.

Conclusion: Repeated-measures ANOVA suggest that students' knowledge of health professions increased significantly post-instruction ($M = 82.74$, $SEM = 1.52$) compared to pre-instruction ($M = 69.94$, $SEM = 4.03$), $F(1,140) = 12.76$, $p = .003$, $p\eta 2 = .48$. If knowledge of health professions can be introduced before a student begins their individual discipline, they may be less likely to form negative stereotypes and more likely to welcome a team approach to medicine. The collaborative role of librarians and Health Science faculty provides a course rich in resources. Further studies should continue to assess the impact of this joint course.

Poster Number: 169

Time: Sunday, May 5, 3:30 p.m.–4:25 p.m.

Virtual Reality in Medical and Health Education: A Review of the Literature

Elisandro Cabada, Medical and Bioengineering Librarian, University of Illinois at Urbana-Champaign, University Library, Urbana, IL

Background: To support the development of a new engineering-focused medical school, a literature review was performed of research articles on how virtual reality, augmented reality, extended reality, and mixed reality technologies have been utilized in medical and health education. These technologies have the potential to enable new approaches to how physician innovators learn new skills and develop an understanding of medicine within and outside of the clinical setting. Immersive technologies can also serve as a bridge between medical and engineering educators to help foster collaboration and drive innovation to understand and solve the challenges we face with human health.

Description: This study was performed by utilizing a SQL query through the Scopus abstract and indexing database and API, focusing on peer-reviewed literature from 2008 to 2018. Information on how these technologies have been implemented in medical and health education, research, and instruction was reviewed to identify affordances, trends and novel uses. Data on equipment utilized, related software, programming languages, and cost was also included where available.

Conclusion: With a better understanding of how virtual reality and related equipment has been implemented in medical and health education, researchers and instructors can better identify how to incorporate these emerging technologies into their own curricula and research in new and innovative ways. With this understanding, administrators and lab managers are also provided with more information to help with strategic budget planning and to better inform where resources are invested.

Poster Number: 170

Time: Monday, May 6, 3:30 p.m.–4:25 p.m.

Where Do I Find...? An Analysis of Fifteen Medical Library Website Home Pages

Dana Haugh, Web Services Librarian, Yale University, Cushing/Whitney Medical Library, New Haven, CT

Objectives: How are medical libraries presenting information to users on their website homepage? What are the most commonly listed resources? What words are used to categorize items? What content management system is used? How many links are on the homepage? What is the first item you notice when you visit the homepage?

Methods: I am using a descriptive research design method to analyze 15 medical library websites. Each medical library website is from an academic institution with graduate schools of medicine in the United States. I am collecting qualitative and quantitative data based on research questions listed in the 'Objectives' section, with additional followup questions based on findings.

Results: Approximately 60% of the medical library websites are hosted on Drupal (half on version 7, half on version 8); only two of the websites do not feature an embedded search bar on the homepage; 80% of the embedded search bars can be 'changed' depending on the user search needs (to search the catalog, articles, journals, etc.); only 1 website does not have a list of popular databases on the homepage; most refer to this list of databases as 'Resources' with variations including 'Suggested Resources', 'Featured Resources', 'Key Resources' and 'Top Resources'; the number of listed databases ranges from 2 to 21; PubMed is featured on every list; only 1 website does not have a section for 'Upcoming classes/workshops' on the homepage; the most popular way of presenting information about upcoming classes is through a feed, with most websites listing 2-3 upcoming classes and a link to 'more'; a horizontal navigation bar is the most popular orientation, though 4 websites have side navigation, and 3 websites do not have medical library site-specific navigation at all (these feature a larger library or university website navigation menu); the number of menu items ranged from 4 to 11; over half of the website homepages featured a 'live chat' option; 3 of the websites were not responsive (not mobile-friendly); most website homepages feature 50-90% of the content 'above the fold'.

Conclusions: It's no wonder users often have trouble navigating library websites. The variety, styling, format, and language used to describe the same types of content varies dramatically from library website to library website. The findings of this study demonstrate the ways in which medical library websites attempt to mitigate the complexity of library services and resources.

Poster Number: 171

Time: Tuesday, May 7, 3:30 p.m.–4:25 p.m.

Enhancing Patient Experience through Patient-Centered Library Services

Sarah L. Carnes, AHIP, Clinical Librarian, Veterans Health Administration, Bedford, MA

Background: This medical library supports not only staff but also patients who reside at or visit the medical center. Only 15% of inpatients and patient residents were receiving services and only an average of three people per day were visiting the library. Providing high quality library services can support patient care plans and enhance Patient Experience. The library team endeavored to refine their program over the course of one year to increase services and access by more than 400% while exceeding the goal of 70% patient and staff satisfaction.

Description: The library team employed a systematic approach to evaluate and improve services. An assessment of current services, needs and expectations involved baseline measures of library access and deliveries as well as process flow mapping. This identified performance gaps for which the library team used tools such as Cause and Effect and Plan Do Study Act (PDSA) to develop courses of action during Phase I. Extensive improvements were made to services, materials, and the library environment, including the incorporation of assistive technology and increased staffing and hours. During Phase II, the team used library service design heuristics and customer service evaluations to evaluate and refine these improvements. The team continues to use adapted Customer Service Questionnaires (CSQ) and an Inputs, Process, Outputs (IPO) tool to monitor the patient library program.

Conclusion: Library visits per day increased by an average of 9.3 people. The number of inpatients supported increased by an average of 163 people per week. The staff burden goal was exceeded. Staff indicated that the services created a burden of just 1.1 on a scale of 1-4, with lower being better. The patient satisfaction goal was also exceeded. On a scale of 1-4, with higher being optimal, patients rated services a 3.9 and attested that their Patient Experience was markedly improved. The enhanced services created no cost to the medical center and the return on investment is estimated at $90,000.00.

Poster Number: 172

Time: Monday, May 6, 3:30 p.m.–4:25 p.m.

Word Trees for Visualizing PubMed Search Results

Edwin Vincent Sperr Jr., AHIP, Clincial Information Librarian, AU/UGA Medical Partnership, Athens, GA

Background: It can be daunting to begin the search process for a complicated question, particularly if a searcher is new to a topic and not yet familiar with the language used to describe it. As many biomedical searches yield several hundred citations or more, it would be helpful for a user to find some way to summarize her results and see the context into which individual search terms fit. This describes an attempt to meet this need by adapting the Word Trees tool to visualize PubMed search results.

Description: Word Trees were developed by Wattenberg and Viégas, based on their work on IBM's Many Eyes platform. Much like Keyword In Context, Word Trees present terms in the context of the words that adjoin them. Uniquely, Word Trees also show the relative frequency of these combinations in an intuitive way.

The application described here takes a user's input and interactively performs a search against PubMed using NCBI's E-utilities API. It then extracts the titles and abstracts from the first hundred search results and graphs that text using the version of Word Trees that is hosted on Google Charts. This process generates a visualization of the context in which individual terms appear for that set of results. This in turn can provide insight into how those terms are used in the broader biomedical literature.

Conclusion: Simple searches show expected results -- "breast" is commonly followed by "cancer", and those two words are most often succeeded by "cells". However, even simple searches can yield insights if one takes advantage of the interactive nature of the Word Tree interface. A search for "morphine" shows "equivalent" as a common succeeding word, and selecting that quickly leads one to the term of art, "MME". This demonstrates how a searcher using this tool might uncover useful terms for search strategy refinement even before she begins examining her first set of results.

Poster Number: E1

Time: Sunday, May 5, 12:00 p.m.–1:55 p.m.

Online Journal Club Format Conducive to an Interprofessional Team

Helen-Ann Brown Epstein, AHIP, FMLA, Informationist, Virtua Health, Mt Laurel, NJ

Background: This author was a member of the first cohort of the MLA Research Training Institute (RTI) in July 2018. It was a 5-day experience funded by a generous IMLS grant and participants came away with a project topic. As a co-convener of the NY-NJ MLA Chapter Online Journal Club and the Informationist member of interprofessional patient care rounding teams, my RTI project will investigates online journal club formats for interprofessional teams.

Aim: Determine which online journal club format is conducive for learning of an interprofessional team, including the Informationist.

Method: Applying Vygotsky's Theory of Social Constructivism, where new knowledge and wisdom is built through social interaction, an invited group of health professionals will take the COLLES-Constructivist On-Line Learning Environment Survey before the journal clubs to set their expectations, then participate in several journal club formats, take the COLLES after to see if expectations were met and debrief about the interaction in the various formats. The COLLES measures perceptions of professional relevance, reflective thinking, interactivity, cognitive demand, affective support and interpretation of meaning.

Anticipated Results: Based on longstanding research about the value of journal clubs and reports based on the theory of social constructivism to build new knowledge through social interaction, a format will be selected for future journal clubs.

Poster Number: E2

Time: Sunday, May 5, 12:00 p.m.–1:55 p.m.

Medical Students and Wikipedia Editing: Implications for Information Literacy

Melissa Kahili-Heede, Information Services and Instruction Librarian, University of Hawaii, John A. Burns School of Medicine, Honolulu, HI

Objectives: This poster presents the results of a pilot study that examines the use of Wikipedia editing with first-year medical students as an intervention for reinforcing and improving information literacy skills. The study took place at the University of Hawaii at Manoa, John A. Burns School of Medicine with students from the MD class of 2022.

Methods: The students learned to edit and contribute to Wikipedia over a period of three months from August to November 2018. At the conclusion of the editing experience, students were asked to respond to a retrospective pre/post survey to assess the impact of Wikipedia editing on various information literacy skills.

Results: Data gathered from the survey show a statistically significant improvement in the students perceived information literacy skills.

Poster Number: E3

Time: Sunday, May 5, 12:00 p.m.–1:55 p.m.

Factors Affecting Clinical Referrals to the Medical Library

Elizabeth Kellermeyer, Biomedical Research Librarian, National Jewish Health, Denver, CO

Objectives:

The objective of this study is to understand why and when clinical care teams refer patients to the medical library. It provides information about how clinical care teams currently utilize and recommend medical libraries as a patient resource.

Methods:

A census of clinical care teams at a research hospital was taken from October to December 2018. The census population was 482; 95 responses were collected. An anonymous online survey using REDCap was administered, featuring multi-choice questions and Likert-type scales to measure awareness of library services available to patients, facilitators and barriers to referral, and likelihood of future referral. Demographic variables included gender, age, professional role, and years employed at the hospital. Spearman correlations were used to determine the strength of relationships between familiarity with the services and how often respondents referred those services (rs). Referral rate distributions were compared between job type groups using the Kruskal-Wallis test.

Results:

Overall, self-reported referral rates were low. There was a marginally significant relationship between referral rate and job type, with providers having lower referral rates ($p=0.01$). There was a positive correlation between familiarity with the services and service referral frequency ($rs=0.78$ for combined data) and between current referral rates and likelihood of future referral ($rs=0.43$ for combined data). Among respondents who had never referred patients, top reasons were “Not aware library offered has service” and “Don’t know how to make a referral to library;” least selected reasons were “Clinic wants to control information to patient” and “Concerned about quality of information provide by library.” Top referral reasons were “Complements or enhances the patient’s access to health information” and “Adds value to the patient’s visit.”

Conclusions:

The results suggest there is evidence that lack of knowledge, rather than lack of interest and support, results in lower clinic referrals to the library. The correlation between knowledge of services and likelihood of referral demonstrates that when providers are aware of the library, they are referring patients. Similarly, those currently referring are likely to make future referrals, suggesting that the library services are considered valuable. The qualitative responses show agreement, linking the lack of referrals to marketing and procedural insufficiencies (rather than distrust or dislike of the services), which indicates potential for increasing referrals by addressing these deficits. A streamlined patient referral system from clinic to library could be beneficial.

Poster Number: E4

Time: Sunday, May 5, 12:00 p.m.–1:55 p.m.

"I Want to Be Open, But How Can I?": Publication Habits and Perceptions of Open Access Publishing amongst Clinical Fellows

Robin O'Hanlon, Associate Librarian, User Services, Memorial Sloan Kettering Cancer Center Library, New York, NY

Introduction: Open Access (OA) publishing rates have risen dramatically in many disciplines within the past decade, including the biomedical sciences. Previous studies have assessed the OA publication rates of scientific researchers, but few have focused specifically on the publishing activities of early career researchers and trainees. The aim of this study was to examine the current publishing activities of clinical and research fellows at Memorial Sloan Kettering Cancer Center (MSKCC), as well as perceptions of OA publishing amongst this population.

Methods: This study employed a mixed methods approach. Publication (n=1489) data authored by clinical and research MSKCC fellows (n=218) between 2013-2018 were collected via an in-house author profile and publication system (Synapse) and citation indexes (Scopus and Web of Science). Publication data collected included the publication model the journal was published, the current OA availability of the publication, if the fellow was listed as first author, and if the fellow was affiliated with MSKCC at the time of publication. Additionally, interviews were conducted with MSKCC fellows (n=15) to discern their perceptions of OA publishing. Interview responses were transcribed and thematically coded.

Results: The average number of publications per fellow was 6.8. The total number of publications that were currently available OA was 28.6% (n=426), while the average number of publications that were currently available OA per fellow was 1.5. The journal publication model with the most publications represented was Hybrid, at 77.5% (n=1146). On average, fellows had published 5.29 publications in Hybrid journals. The rate of publications that were available OA remained relatively flat between 2013-2018. Fellows cited high Author Processing Charges and perceived lack of journal quality/prestige as barriers to OA publishing. The relationship between first authorship and whether or not a fellow’s publication was currently available OA was not statistically significant, X2(1) 1.941, p=0.184.

Conclusions: While the fellows in this study acknowledged the potential of OA to aid in research dissemination, they also expressed hesitation to publish OA. Despite on-going educational and advocacy efforts of OA proponents, including information professionals, misconceptions regarding OA publishing persists. Specifically, confusion surrounding legitimate OA publications and predatory publications exists. This confusion, coupled with the pressure to publish in well-established, high impact journals, means early career researchers face unique challenges in navigating the scholarly publishing landscape.

Poster Number: E5

Time: Sunday, May 5, 12:00 p.m.–1:55 p.m.

How Can We Help? Information Literacies of Doctoral (PhD) Students in the Health Sciences

Elisabeth Nylander, Liaison, Instruction & Research Librarian for the School of Health and Welfare, Jönköping University, Jönköping, N/A, Sweden

INTRODUCTION

Doctoral studies offer a unique phase in the development and legitimization of researchers, in which PhD students shift from the consumption to the production of knowledge. If librarians are to support this process in an evidence-based manner, it is essential to understand the distinct practices of this population. While literature exists concerning the information behavior of graduate students and researchers, there is little work which focuses specifically on the information literacies of PhD students within the health sciences.

AIM

The aim of this project was: 1) to establish the depth and breadth of evidence describing the information literacies of PhD students within the health sciences, and 2) to explore how Jönköping University Library can support the PhD students and their supervisors at the Research School of Health and Welfare.

METHOD

The project aims suggested a mixed method approach. In order to examine the concept of information literacies among PhD students, a scoping review was performed [1]. General trends within the literature were mapped based on the extraction of the following data: geographic location, population, academic discipline, and method of investigation. To better understand the information practices at our health sciences research school, we interviewed both PhD students and their supervisors. These open-ended interviews were conducted and analyzed according to a hermeneutic dialectic process [2], resulting in synthesized constructions of the study participants' experiences.

RESULTS

Our scoping review revealed that many studies fail to treat doctoral studies as a unique process. The result is that PhD students in the health sciences are underrepresented as a distinct group within the recent literature. We are currently performing a critical analysis of the few studies that focus specifically on health science PhD students. Later this year, we expect to present a discussion of these results as well as the findings from interviews at our own health sciences research school.

CONCLUSION

This project highlights the need for more primary research on the information literacies of PhD students in the health sciences.

REFERENCES

1. Arksey H, O'Malley L. Scoping studies: towards a methodological framework. International Journal of Social Research Methodology. 2005 2005/02/01;8(1):19-32. https://doi.org/10.1080/1364557032000119616.

2. Guba EG, Lincoln YS. Fourth generation evaluation. Newbury Park, Calif: Sage; 1989. ISBN 0-8039-3235-9.

Poster Number: E6

Time: Sunday, May 5, 12:00 p.m.–1:55 p.m.

Understanding the Information Needs of Florida International University-Affiliated Psychiatry Medical Residents: A Case Study

Rebecca Roth, Clinical Engagement Librarian, Florida International University, Miami, FL

OBJECTIVE: The purpose of this project was to assess how well the Florida International University's Herbert Wertheim College of Medicine (FIU HWCOM) Medical Library has been meeting the research, reference, and instruction needs of the medical residents at one of its affiliated clinical sites. The results of this case study will be used to improve the Medical Library's services to this user population. This was also an attempt to pilot a model of assessment that the Medical Library hopes to replicate at other FIU clinically affiliated sites in the future.

METHODS: A brief five-question print survey was administered and a subsequent thirty-minute focus group was conducted. Ten (out of a possible fourteen) medical residents participated. Two of the participants were medical fellows. The focus group was audio-recorded, transcribed, and then analyzed for themes.

RESULTS: One major discovery from the focus group was that the original orientation the Medical Librarians had given to new cohorts of residents when they entered their program had not been sufficient enough to explain the process of acquiring library access. At FIU, clinically affiliated residents do not automatically have library accounts and many were unaware that their library access hinged upon having successfully communicated with the College of Medicine's Human Resources Department. When residents did not know who to contact and for whom getting library access was not a priority (especially at the start of the program), they were disinclined to pursue the issue. Others who initially did attempt to troubleshoot sometimes hit barriers and then decided not to persevere. On the other hand, among the residents interviewed, there were a few for whom library access at some point in their program became a priority. When motivated by need, they figured out how to contact the correct people and achieve library access.

CONCLUSIONS: Suggestions offered by the group included visiting them a second time (after they had gotten through all of the other components of their orientation at the hospital), an invitation for residents to visit the FIU campus, and better documentation of the process on the Library's Website. The researchers assume similar confusion is occurring at other clinical sites affiliated with FIU and will create a more structured procedure for granting new residents library access.

Poster Number: E7

Time: Sunday, May 5, 12:00 p.m.–1:55 p.m.

How Medical Students Discover and Use Medical Information Tools

Margaret A. Hoogland, AHIP, Clinical Medical Librarian, Mulford Health Sciences Library - The University of Toledo, Temperance, MI

Objective

Many studies discuss the use of medical information tools by clinical medical students (i.e. third-and-fourth year students). Few studies examine how preclinical students (i.e. first-and-second year students) discover and use medical information tools. By understanding the needs of preclinical medical students, medical librarians can adjust the content and delivery of information in their training sessions. The purpose of this study is to better understand the medical information needs of preclinical students.

Methods:

Medical students received and email containing the study description, link to an online survey, and an opportunity to answer additional questions about medical information tools. Discussion participants got an Amazon or Starbucks gift card. Survey participants received no compensation.

Results:

Of the 525 students who received an invitation to participate, 122 completed the survey and 18 participated in a discussion. Preclinical students primarily use UptoDate and Epocrates. By contrast, Clinical students use Google, Google Scholar, and UptoDate. During the discussion sessions, most students mention UptoDate first but mention using other medical information tools. Out of 18 students, two students or 11% consulted UptoDate exclusively in a clinical setting.

Conclusion

Students primarily discover medical information tools through conversations, during classes, or one-on-one sessions with faculty and health science librarians. Study results show even a short session with a librarian improves preclinical students' knowledge of available tools and services. Librarians, who adapt sessions and conversations with preclinical students, can impact how students use medical information tools for the remainder of medical school.

Keywords: Medical Information Tools, Medical Students, Information Discovery, and Information Use

Poster Number: E8

Time: Sunday, May 5, 12:00 p.m.–1:55 p.m.

Still Feeling the Effects: A Citation Analysis of the Highly Cited, Retracted Article on Measles, Mumps, and Rubella (MMR) Vaccines and Autism

Elizabeth Suelzer, AHIP, User Education and Reference Librarian, Medical College of Wisconsin, Milwaukee, WI

Introduction: In 1998, Andrew Wakefield published an article that allegedly showed a causal relationship between the measles, mumps and rubella (MMR) vaccination and autism. Although this article was twice retracted (Murch et al., 2004; Editors of the Lancet, 2010), and questions arose about the credibility of Wakefield's data (Deer, 2011) the infamous article continues to be highly cited. This research project analyzed the cited by references of the 1998 Wakefield article to learn more about who cited it and how the article was cited.

Methods: We conducted a cited reference search in Web of Science to identify literature that cited the 1998 Wakefield article. The citations and full text copy for each reference were uploaded into Covidence where each reference was screened to determine the characteristic of the in-text citations using an established taxonomy (Bornmann & Daniel, 2008; Leung et al, 2017) and to see if the retractions of the Wakefield article were referenced. The screening was blinded and conflicts were resolved by group consensus.

Results: A total of 1,153 citations were analyzed. Articles were not included in this study if they were non-English or if we were unable to find the Wakefield reference in the article text. The most common citation characteristics were negational (72%), perfunctory (9%) and affirmational (8%). The Wakefield article was retracted twice: a partial retraction by 10 of the 12 authors in 2004, and a full retraction in 2010. The partial retraction was referenced in 32% of the articles published between 2004-2009. Retractions were referenced in 71% of the articles published after 2010. Since 2013, the percentage of authors who referenced the retraction is continually growing.

Conclusions: Overwhelmingly, scholarly literature negated the findings of the 1998 Wakefield article, even before the article was retracted. Many citing authors pointed out the methodological flaws of the study and the small sample size. Highly cited papers are presumed to hold more scholarly weight than less cited papers, however, this article was cited for all the wrong reasons.

A significant number of articles published after the retraction did not cite the retraction. Although most citation styles have recommendations on how to cite retractions, not all authors follow them. There is a need for greater vigilance in ensuring that retracted articles are referenced properly.

Poster Number: E9

Time: Sunday, May 5, 12:00 p.m.–1:55 p.m.

Fostering Community: Developing an Online Community of Practice for Health Sciences Librarians

Alicia Lillich, Kansas Outreach and Technology Coordinator, National Network of Libraries of Medicine, MidContinental Region, Kansas City, KS

Poster Number: E10

Time: Sunday, May 5, 12:00 p.m.–1:55 p.m.

Librarians as Teachers: Researching an Educational Knowledge Gap

Kathy J. Davies, Interim Director of Libraries, Augusta University, Augusta University, Augusta, GA

Poster Number: E11

Time: Sunday, May 5, 12:00 p.m.–1:55 p.m.

Trends among Personal Librarian Programs in Medical and Academic Health Sciences Libraries

Natasha Williams, AHIP, User Services Librarian, University of Central Florida College of Medicine, Orlando, FL

Background:

Personal librarian programs (PLPs) are an outreach model for delivering library information and instruction that assigns one librarian to a group of students as their “personal librarian”, with the aim of enhancing and personalizing students’ individual library experience. Much of the literature on this topic pertains to applications in undergraduate libraries within academic institutions. This study examines how PLPs are implemented within medical and academic health sciences libraries.

Methods:

A 26-item survey was developed in Qualtrics and sent to the following library email listservs: MEDLIB-L (1734 subscribers), AAHSL-ALL (350 subscribers), AACRL-HSIG (696 subscribers), and PSS-Lists (102 subscribers). Participants were also invited to contact the researcher if they were interested in participating in a follow-up interview. Survey responses were analyzed in Excel; incomplete survey sessions were excluded from analysis.

Results:

Of the 2,882 emails sent, 49 survey sessions were recorded, and a total of 38 survey sessions were completed (1.3% response rate). Of the 38 completed responses, 12 libraries reported that a PLP had been implemented at their institution (31.5%). Four questions provided operational information the researcher felt to be most useful to libraries exploring creating their own PLPs: “How many librarians participate in your personal librarian program”; “Approximately how many students are assigned to each of the library participants in your program”; “What types of students does your program serve”; “Please describe the services your personal librarian program offers to students”. Follow up interviews were not conducted due to lack of interest.

Discussion:

Response rates for the survey were low given the sample size. This could be due to subscriber overlap across the listservs (e.g, a librarian subscribing to multiple listservs, colleagues all subscribing to the same listserv and allowing one colleague to respond on the institutions behalf), or self-selection.

Conclusion:

While personal librarian programs in medical and academic health sciences libraries are implemented differently from institution to institution, general similarities regarding execution do exist. Further research is necessary to determine additional nuances, and to determine the extent to which these programs are successful.

Poster Number: E12

Time: Sunday, May 5, 12:00 p.m.–1:55 p.m.

The Expectations, Priorities, and Preferences of Students with Disabilities When Seeking Accessibility Information on Academic Library Websites

Amelia Brunskill, Visiting Assistant Professor & Information Services and Liaison Librarian, Library of the Health Sciences, University of Illinois at Chicago, Chicago, IL

Many academic library websites have one or more webpages of information specifically for library users with disabilities. However, there is currently no standardization in terms of what information is included on these pages, how they are labeled, or where they are located within the overall structure of the site, and there appears to be no literature documenting the expectations and preferences of users with disabilities for these pages.

This study involved interviewing twelve university students who self-identify as having a disability about their needs and expectations in regards to information about accessibility resources, services, and facilities provided by the library. While data analysis is still underway, the ultimate intention is to highlight the needs and expectations that were voiced in these discussions.

Poster Number: E13

Time: Sunday, May 5, 12:00 p.m.–1:55 p.m.

Measuring the Curiosity Continuum: Oregon Physician Assistant Perspectives on Curricular and Non-Curricular Contributors to Their Lifelong Learning, Critical Thinking, and Evidence-Based Practice

Laura Zeigen, AHIP, Health Sciences Education and Research Librarian, Oregon Health and Science University, Portland, OR

Poster Number: E14

Time: Sunday, May 5, 12:00 p.m.–1:55 p.m.

Frequency and Effects of Search Strategy Characteristics on Relevant Article Retrieval in Systematic Reviews

Whitney A. Townsend, Informationist, University of Michigan–Ann Arbor

Objectives

To identify common characteristics of highly effective search strategies for a clinical systematic review topic.

Methods

Before attending an in-person systematic review workshop, participants asked to draft a reproducible search strategy based on a brief scenario and a research question from a published systematic review related to blood transfusion and radical prostatectomy. Participants are provided with three studies that were included in the published systematic review, but are not given the systematic review itself. The scenario proposes three commonly-requested limits: date range, inclusion of specific outcome, and human studies that participants can choose to apply or not. The submitted strategies are evaluated for reproducibility and effectiveness of retrieval of the 10 studies included in the published systematic review. Strategies were considered ”highly successful” if they returned all 10 included studies. We conducted a thematic analysis on the 14 highly successful strategies to identify common characteristics between them that could guide future searchers. Two studies were disproportionately missed by the other 98 search strategies, and their PubMed records were analyzed to identify what made them particularly challenging to find.

Poster Number: E15

Time: Sunday, May 5, 12:00 p.m.–1:55 p.m.

Writing Papers: Learning about Information Work-Process of Public Health Students

Mary White, Global Public Health Librarian, UNC Chapel Hill, Chapel Hill, NC

Poster Number: E16

Time: Sunday, May 5, 12:00 p.m.–1:55 p.m.

School-Based Health Clinic Nurse Practitioners Use of Evidence-Based Questionnaire: Needs Assessment and Resource Implementation

Janene Batten, Nursing Librarian, Yale University, Cushing/Whitney Medical Library, New Haven, CT

Poster Number: E17

Time: Sunday, May 5, 12:00 p.m.–1:55 p.m.

Survey of Users of Electronic-Only Health Sciences Library (HSL) Users

Mellanye Lackey, AHIP, Associate Director for Education and Research, University of Utah, Spencer S. Eccles Health Sciences Library, Salt Lake City, UT

Poster Number: E18

Time: Sunday, May 5, 12:00 p.m.–1:55 p.m.

Nurses' Experiences and Perceptions of Using Online Resources for Patient and Family Education: A Qualitative Interview Study

Carrie Grinstead, AHIP, Regional Medical Librarian, System Library Services, Providence St. Joseph Health, Burbank, CA
